# Supplementary material for: Nucleophilic aromatization of monoterpenes from isoprene under nickel/iodine cascade catalysis
Source: Nat Commun. 2023 Nov 4;14:7087. doi: 10.1038/s41467-023-42847-6 (PMC10625535; doi:10.1038/s41467-023-42847-6)
Supplement: Supplementary file 1 — Supplementary Information [file 41467_2023_42847_MOESM1_ESM.pdf]

Supplementary Information for

Nucleophilic Aromatization of Monoterpenes from Isoprene under Nickel/Iodine Cascade Catalysis

Wei-Song Zhang,[1,2] Ding-Wei Ji,[1] Yang Yang,[1] Ting-Ting Song,[1] Gong Zhang,[1,2] Xiao-Yu Wang,[1,2] and Qing-An Chen*,[1,2]

[1]Dalian Institute of Chemical Physics, Chinese Academy of Sciences, Dalian 116023, People's Republic of China

[2]University of Chinese Academy of Sciences, Beijing 100049, People's Republic of China

*Corresponding author: E-mail: qachen@dicp.ac.cn, Web: www.lbcs.dicp.ac.cn

Table of Contents

1. Supplementary Note 1 .... S2
2. Supplementary Note 2 .... S2
  2.1. Optimization for nucleophilic aromatization from limonene S2
  2.2. Optimization for nucleophilic aromatization from isoprene S3
3. Supplementary Method 1 .... S4
  3.1 Procedure for the synthesis of substrates S4
  3.2 General procedure A: Ni/I_2 catalyzed nucleophilic aromatization from isoprene S5
  3.3 General procedure B: I_2 catalyzed nucleophilic aromatization from terpenes S12
  3.4 Convergent synthesis strategy S13
  3.5 General procedure C: I_2 catalyzed hydroarylation of olefins S14
4. Supplementary Note 3 .... S15
  4.1 Kinetics experiment for the dimerization of isoprene S15
  4.2 Kinetics experiment for the aromatization of terpenes S16
  4.3 Deuterium labeling experiment S19
  4.4 KIE experiment S22
  4.5 Kinetics experiment for the coupling of indole and limonene S23
  4.6 Control experiments S24
  4.7 The role of KI S25
  4.8 Synthesis and transformation of the possible intermediates S26
  4.9 Capture of iodide intermediates S26
  4.10 Effect of the amount of isoprene S27
5. Supplementary Method 2 .... S27
  5.1 General procedure D: Orthogonal C-H functionalizations S27
  5.2 Transformations S29
  5.3 Scale-up experiment S33
6. Supplementary Note 4 .... S34
7. Supplementary References S91

1. Supplementary Note 1

All the reagents were commercially available and were used without further purification unless otherwise stated. Solvents were treated prior to use according to the standard methods. Unless otherwise stated, all reactions were conducted under inert atmosphere using standard Schlenk techniques or in an argon-filled glove-box. ^{1}H NMR and ^{13}C NMR spectra were recorded at room temperature in $CDCl_3$ on 400 MHz or 700 MHz instrument with tetramethylsilane (TMS) as internal standard. Data are reported as follows: chemical shift in ppm (δ), multiplicity (s = singlet, d = doublet, t = triplet, q = quartet, brs = broad singlet, m = multiplet), coupling constant (Hz), and integration. Flash column chromatography was performed on commercially available silica gel (200-300 mesH); All reactions were monitored by TLC, GC-FID, GC-MS or NMR analysis. HRMS data was obtained with Micromass HPLC-Q-TOF mass spectrometer (ESI) or Agilent 6540 Accurate-MS spectrometer.

2. Supplementary Note 2（yields and selectivities were determined by GC-FID analysis of the crude products mixture using trimethoxybenzene as internal standard）

2.1. Optimization for nucleophilic aromatization from limonene

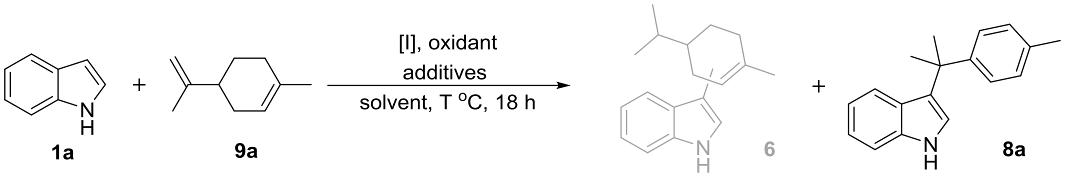

Supplementary Table 1. Effect of oxidants[a]

Entry	Oxidant	Yield of **6** (%)	Yield of **8a** (%)
1[b]	--	16	12
2	NaClO	--	--
3	$NaClO_4$	47	8
4	$^tBuO_2H$	--	--
5	$K_2S_2O_8$	7	60
6[c]	$K_2S_2O_8$	2	5
7[d]	$K_2S_2O_8$	--	--

[a]Conditions: **1a** (0.20 mmol), **9a** (0.30 mmol), I_2 (20 mol%), oxidant (0.30 mmol, 1.5 equiv.), THF (0.50 mL), air atmosphere, 80 °C, 18 h. [b]I_2 (0.30 mmol); [c]I_2 (10 mol%), [d]KI (40 mol%) was used instead of I_2.

Supplementary Table 2. Effect of solvents[a]

Entry	Solvent	Yield of **6** (%)	Yield of **8a** (%)
1	MeCN	5	21
2	TFE	--	--
3	1,4-dioxane	27	25
4	$MeNO_2$	--	--

[a]Conditions: **1a** (0.20 mmol), **9a** (0.30 mmol), I_2 (20 mol%), $K_2S_2O_8$ (0.30 mmol, 1.5 equiv.), solvent (0.50 mL), air atmosphere, 80 °C, 18 h.

Supplementary Table 3. Effect of temperature[a]

Entry	Temperature/°C	Yield of **6** (%)	Yield of **8a** (%)
1	40	3	trace
2	60	4	31
3	80	5	61
4	100	5	66

[a]Conditions: **1a** (0.20 mmol), **9a** (0.30 mmol), I_2 (20 mol%), $K_2S_2O_8$ (0.40 mmol, 2.0 equiv.), THF (0.50 mL), N_2 atmosphere, T °C, 18 h.

Supplementary Table 4. Effect of additives[a]

Entry	Additive	Yield of **6** (%)	Yield of **8a** (%)
1	NaI	6	59
2	KI	7	66
3	ZnI_2	14	50
4	$CrCl_3$	7	54
5	$FeCl_3$	37	19
6	$AlCl_3$	14	3
7	$InBr_3$	15	8
8[b]	KI	5	76

[a]Conditions: **1a** (0.20 mmol), **9a** (0.30 mmol), I_2 (20 mol%), additives (20 mol%), $K_2S_2O_8$ (0.40 mmol, 2.0 equiv.), THF (0.50 mL), N_2 atmosphere, 80 °C, 18 h. [b]KI (10 mol%), 100 °C, 6 h, isolated yield of **8a**.

2.2. Optimization for nucleophilic aromatization from isoprene (two steps in one pot)

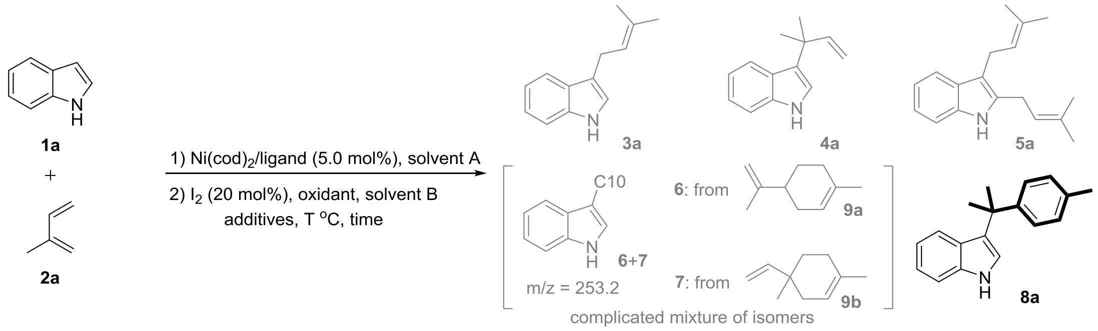

Supplementary Table 5. Effect of bases[a]

Entry	Base	Yield of **3a** and **4a** (%)	Yield of **5a** (%)	Yield of **6** and **7** (%)	Yield of **8a** (%)
1	KO^tBu	--	--	trace	4
2	NaO^tBu	--	--	5	61
3	LiO^tBu	35	7	--	--
4	NaOMe	--	--	4	5
5	$NaOSiMe_3$	--	--	3	34

[a]Conditions: Step I: **2a** (1.2 mmol), $Ni(cod)_2$/IPr·HCl (5.0 mol%), base (10 mol%), *n*-hexane (0.50 mL), 100 °C, 12 h; Step II: **1a** (0.20 mmol), I_2 (20 mol%), $K_2S_2O_8$ (2.0 equiv.), KI (10 mol%), THF (0.50 mL), 100 °C, 24 h.

Supplementary Table 6. Effect of oxidants[a]

Entry	Oxidant	Yield of **6** and **7** (%)	Yield of **8a** (%)
1	$Na_2S_2O_8$	4	64
2	$K_2S_2O_8$	5	61
3	$(NH_4)_2S_2O_8$	4	55

[a]Conditions: Step I: **2a** (1.2 mmol), $Ni(cod)_2$/IPr·HCl (5.0 mol%), NaO^{t}Bu (10 mol%), *n*-hexane (0.50 mL), 100 °C, 12 h; Step II: **1a** (0.20 mmol), I_2 (20 mol%), Oxidant (2.0 equiv.), KI (10 mol%), THF (0.50 mL), 100 °C, 24 h.

Supplementary Table 7. Effect of solvent A[a]

Entry	Solvent A	Yield of **6** and **7** (%)	Yield of **8a** (%)
1	Cyclohexane	4	62
2	CME	21	8
3	EA	--	trace
4	NMP	--	14
5	MeCN	--	--
6	1,4-dioxane	4	48
7	DCE	--	--
8	EtOH	--	trace

[a]Conditions: Steps I: **2a** (1.2 mmol), $Ni(cod)_2$/IPr·HCl (5.0 mol%), NaO^{t}Bu (10 mol%), solvent A (0.50 mL), 100 °C, 12 h; Step II: **1a** (0.20 mmol), I_2 (20 mol%), $Na_2S_2O_8$ (2.0 equiv.), KI (10 mol%), THF (0.50 mL), 100 °C, 18 h.

Supplementary Table 8. Effect of acids[a]

Entry	Acid	Yield of **6** and **7** (%)	Yield of **8a** (%)
1	$CH_3CH_2CO_2H$	6	66
2	TFA	6	59
3	$PhSO_3H$	5	64
4	$AdCO_2H$	7	68
5	$(PhO)_2PO_2H$	6	72
6[b]	$(PhO)_2PO_2H$	5	82

[a]Conditions: Step I: **2a** (1.2 mmol), $Ni(cod)_2$/IPr·HCl (5.0 mol%), NaO^{t}Bu (10 mol%), solvent A (0.50 mL), 100 °C, 12 h; Step II: **1a** (0.20 mmol), I_2 (20 mol%), $Na_2S_2O_8$ (2.0 equiv.), KI/acid (10 mol%), THF (0.50 mL), 100 °C, 18 h. [b]12 + 24 h.

3. Supplementary Method 1

3.1 Procedure for the synthesis of substrates

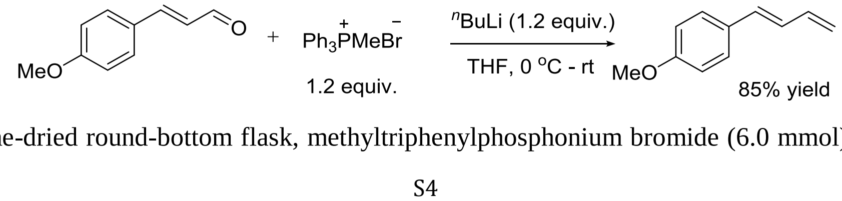

To a flame-dried round-bottom flask, methyltriphenylphosphonium bromide (6.0 mmol) in THF (40

mL) was added ^{n}BuLi (2.4 mL, 2.5 M in *n*-hexane, 6.0 mmol) slowly at 0 °C under N_2. After stirring for 20 min, a cinnamaldehyde (5.0 mmol) was added. The reaction mixture was then warmed to room temperature and stirred for another 10 hours. After the starting material was consumed completely which was detected by TLC, the reaction mixture was quenched with sat. NH_4Cl aq. (15 mL) and extracted with diethyl ether (20 mL × 3). The combined organic layers were dried over Na_2SO_4, concentrated in vacuo and purified by flash chromatography on silica gel with *n*-pentene or *n*-hexane to afford the diene product (682.4 mg, 85% yield).

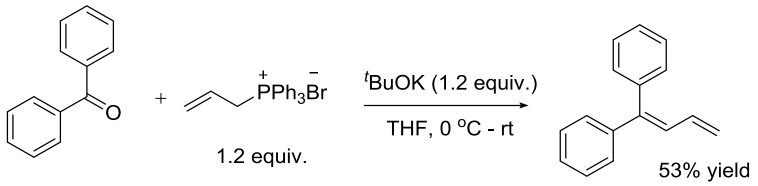

To a flame-dried round-bottom flask, allyltriphenylphosphonium bromide (6.0 mmol) in THF (40 mL) was added potassium *tert*-butoxide (6.0 mmol) at 0 °C under N_2. After stirring for 20 min, a diphenyl ketone (5.0 mmol) was added. The reaction mixture was then warmed to room temperature and stirred for another 10 hours. After the starting material was consumed completely which was detected by TLC, the reaction mixture was quenched with sat. NH_4Cl aq. (15 mL) and extracted with diethyl ether (20 mL × 3). The combined organic layers were dried over Na_2SO_4, concentrated in vacuo and purified by flash chromatography on silica gel with *n*-hexane to afford the diene product (545.1 mg, 53% yield).

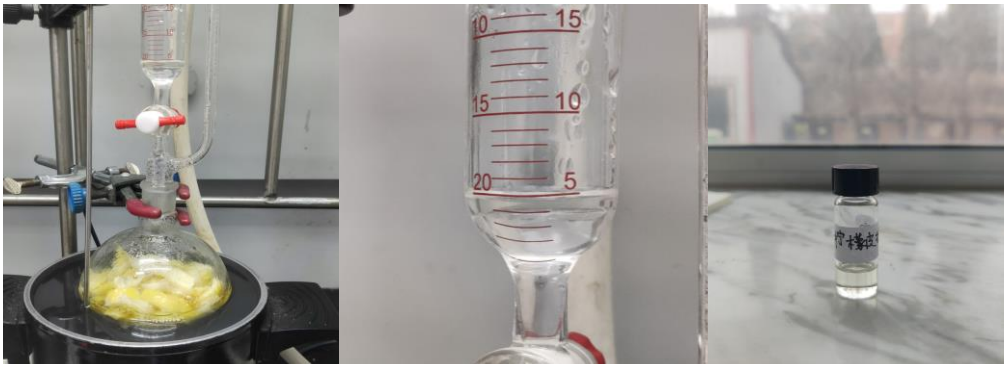

The fresh lemon peel (500 g) was cut small pieces into a round-bottom flask and 250 mL water was added to soak for one hour. The mixture was heated to reflux and the distillate was collected. After 4 h, the mixture was cooled to rt and obvious stratification was observed. The oil on top was collected (1.2 mL) and no further purification was needed. The oil mixture was analyzed by GC-FID (69% of limonene, 9% of β-pinene, 10% of γ-terpinene and 12% of others).

3.2 General procedure A: Ni/I_2 catalyzed nucleophilic aromatization from isoprene

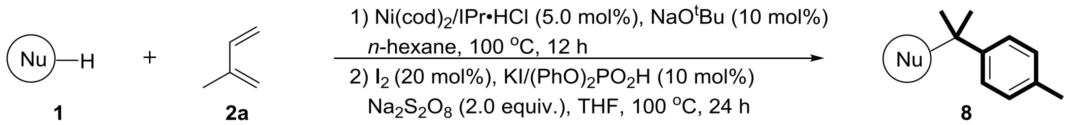

1 **2a** **8**

Step I: In a glove box, a sealed tube was charged with $Ni(cod)_2$ (0.01 mmol, 5.0 mol%), IPr·HCl (0.01 mmol, 5.0 mol%), NaO^{t}Bu (0.02 mmol, 10 mol%), isoprene **2a** (1.2 mmol), *n*-hexane (0.50 mL) at room temperature. The reaction tube was sealed with a Teflon screw cap, removed from the glove box. Then, the reaction mixture was stirred at 100 °C for 12 hours. Step II: As the reaction mixture was cooled to room temperature, $(PhO)_2PO_2H$ (0.02 mmol, 10 mol%) was added to the reaction tube and

stirred for 5 minutes. Then KI (0.02 mmol, 10 mol%), $K_2S_2O_8$ (0.40 mmol, 2.0 equiv.), nucleophile **1** (0.20 mmol), I_2 (0.04 mmol, 20 mol%) and THF (0.50 mL) were added into the reaction mixture and stirred at 100 ℃ for additional 24 hours. Direct purification by column chromatography on silica gel using petroleum ether and ethyl acetate afforded the corresponding product **8**.

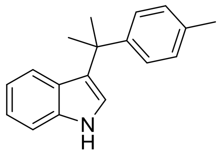

3-(2-(*p*-Tolyl)propan-2-yl)-1H-indole (8a): Prepared according to the general procedure A, yellow oil, 39.2 mg, 79% yield, R_f = 0.3 (PE/EA = 20/1), ^{1}**H NMR** (400 MHz, Chloroform-*d*) δ 7.87 (s, 1H), 7.33 (d, *J* = 8.1 Hz, 1H), 7.25 (d, *J* = 7.7 Hz, 2H), 7.15-7.04 (m, 5H), 6.91 (t, *J* = 7.7 Hz, 1H), 2.32 (s, 3H), 1.78 (s, 6H); ^{13}**C NMR** (100 MHz, Chloroform-*d*) δ 147.02, 137.21, 134.99, 128.83, 126.38, 126.32, 126.16, 121.70, 121.50, 120.64, 118.95, 111.14, 38.69, 30.82, 21.07. **HRMS** calculated for $C_{18}H_{20}N$ $[M+H]^+$ 250.1590, found 250.1596.

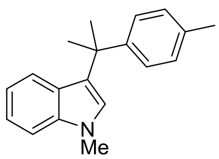

1-Methyl-3-(2-(*p*-tolyl)propan-2-yl)-1H-indole (8b): Prepared according to the general procedure A, colorless oil, 39.9 mg, 76% yield, R_f = 0.8 (PE/EA = 20/1), ^{1}**H NMR** (700 MHz, Chloroform-*d*) δ 7.31 (d, *J* = 8.2 Hz, 1H), 7.29 (d, *J* = 8.2 Hz, 2H), 7.18 (t, *J* = 7.6 Hz, 1H), 7.13 (d, *J* = 8.0 Hz, 1H), 7.10 (d, *J* = 8.0 Hz, 2H), 6.98 (s, 1H), 6.92 (t, *J* = 7.5 Hz, 1H), 3.80 (s, 3H), 2.35 (s, 3H), 1.80 (s, 6H); ^{13}**C NMR** (175 MHz, Chloroform-*d*) δ 147.19, 137.86, 134.92, 128.80, 126.53, 126.41, 125.60, 124.79, 121.56, 121.24, 118.37, 109.18, 38.67, 32.75, 30.97, 21.07. **HRMS** calculated for $C_{19}H_{22}N$ $[M+H]^+$ 264.1747, found 264.1756.

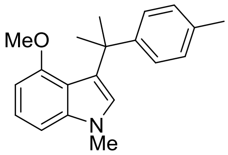

4-Methoxy-1-methyl-3-(2-(*p*-tolyl)propan-2-yl)-1H-indole (8c): Prepared according to the general procedure A, colorless oil, 41.4 mg, 71% yield, R_f = 0.4 (PE/EA = 50/1), ^{1}**H NMR** (700 MHz, Chloroform-*d*) δ 7.18 (d, *J* = 8.2 Hz, 2H), 7.11 (t, *J* = 7.9 Hz, 1H), 7.03 (d, *J* = 8.0 Hz, 2H), 6.91 (d, *J* = 8.2 Hz, 1H), 6.87 (s, 1H), 6.36 (d, *J* = 7.7 Hz, 1H), 3.76 (s, 3H), 3.37 (s, 3H), 2.31 (s, 3H), 1.77 (s, 6H); ^{13}**C NMR** (175 MHz, Chloroform-*d*) δ 154.13, 149.24, 139.84, 133.75, 128.17, 125.98, 125.43, 124.99, 122.48, 117.10, 102.36, 100.10, 54.58, 39.08, 33.01, 31.61, 21.04. **HRMS** calculated for $C_{20}H_{24}NO$ $[M+H]^+$ 294.1852, found 294.1851.

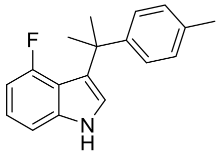

4-Fluoro-3-(2-(*p*-tolyl)propan-2-yl)-1H-indole (8d): Prepared according to the general procedure A, yellow oil, 43.4 mg, 81% yield, R_f = 0.3 (PE/EA = 20/1), ^{1}**H NMR** (400 MHz, Chloroform-*d*) δ 7.99 (s, 1H), 7.21 (d, *J* = 8.2 Hz, 2H), 7.14 (d, *J* = 8.1 Hz, 1H), 7.12-7.01 (m, 4H), 6.61 (dd, *J* = 11.3, 7.7 Hz, 1H), 2.32 (s, 3H), 1.78 (s, 6H); ^{13}**C NMR** (100 MHz, Chloroform-*d*) δ 156.37 (d, *J* = 248.5 Hz), 147.65, 140.16 (d, *J* = 12.0 Hz), 134.71, 128.63, 126.04, 125.61 (d, *J* = 4.5 Hz), 122.69 (d, *J* = 8.1 Hz), 121.10, 114.94 (d, *J* = 20.7 Hz), 107.15 (d, *J* = 3.6 Hz), 105.10 (d, *J* = 21.7 Hz), 38.68, 31.15 (d, *J* = 4.3 Hz), 21.11; ^{19}**F NMR** (376 MHz, Chloroform-*d*) δ -113.32. **HRMS** calculated for $C_{18}H_{19}FN$ $[M+H]^+$ 268.1496, found 268.1493.

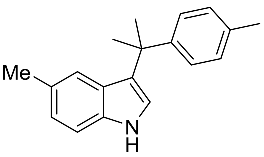

5-Methyl-3-(2-(*p*-tolyl)propan-2-yl)-1H-indole (8e): Prepared according to the general procedure A (48 h for the second step), yellow oil, 37.5 mg, 71% yield, R_f = 0.3 (PE/EA = 20/1), ^{1}**H NMR** (700 MHz, Chloroform-*d*) δ

7.80 (s, 1H), 7.27 (d, *J* = 8.4 Hz, 2H), 7.24 (d, *J* = 8.2 Hz, 1H), 7.09 (d, *J* = 8.0 Hz, 2H), 7.06 (d, *J* = 2.1 Hz, 1H), 6.97 (d, *J* = 8.4 Hz, 1H), 6.94 (s, 1H), 2.34 (s, 3H), 2.32 (s, 3H), 1.78 (s, 6H); **^{13}C NMR** (175 MHz, Chloroform-*d*) δ 147.08, 135.59, 134.91, 128.82, 128.02, 126.39, 126.36, 125.72, 123.35, 121.15, 120.89, 110.81, 38.71, 30.84, 21.71, 21.07. **HRMS** calculated for $C_{19}H_{22}N$ $[M+H]^+$ 264.1747, found 264.1745.

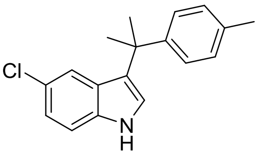

5-Chloro-3-(2-(*p*-tolyl)propan-2-yl)-1H-indole (8f): Prepared according to the general procedure A, yellow oil, 52.2 mg, 92% yield, R_f = 0.2 (PE/EA = 20/1), **^{1}H NMR** (700 MHz, Chloroform-*d*) δ 7.93 (s, 1H), 7.24 (d, *J* = 9.3 Hz, 1H), 7.22 (d, *J* = 8.1 Hz, 2H), 7.13 (d, *J* = 2.3 Hz, 1H), 7.11-7.05 (m, 4H), 2.34 (s, 3H), 1.76 (s, 6H); **^{13}C NMR** (175 MHz, Chloroform-*d*) δ 146.44, 135.57, 135.25, 128.97, 127.21, 126.24, 126.19, 124.61, 122.08, 122.03, 120.77, 112.12, 38.58, 30.73, 21.06. **HRMS** calculated for $C_{18}H_{19}{}^{35}ClN$ $[M+H]^+$ 284.1201, found 284.1199; $C_{18}H_{19}{}^{37}ClN$ $[M+H]^+$ 286.1171, found 286.1167.

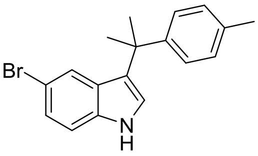

5-Bromo-3-(2-(*p*-tolyl)propan-2-yl)-1H-indole (8g): Prepared according to the general procedure A, yellow oil, 49.5 mg, 75% yield, R_f = 0.2 (PE/EA = 20/1), **^{1}H NMR** (400 MHz, Chloroform-*d*) δ 7.91 (s, 1H), 7.26 (s, 1H), 7.25-7.17 (m, 4H), 7.14-7.05 (m, 3H), 2.35 (s, 3H), 1.77 (s, 6H); **^{13}C NMR** (100 MHz, Chloroform-*d*) δ 146.41, 135.83, 135.25, 128.96, 127.88, 126.22, 126.09, 124.62, 123.80, 121.90, 112.59, 112.29, 38.59, 30.74, 21.05. **HRMS** calculated for $C_{18}H_{19}{}^{79}BrN$ $[M+H]^+$ 328.0695, found 328.0696; $C_{18}H_{19}{}^{81}BrN$ $[M+H]^+$ 330.0675, 330.0677.

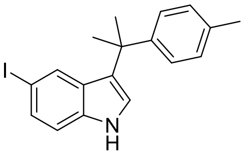

5-Iodo-3-(2-(*p*-tolyl)propan-2-yl)-1H-indole (8h): Prepared according to the general procedure A, yellow oil, 51.1 mg, 68% yield, R_f = 0.2 (PE/EA = 20/1), **^{1}H NMR** (700 MHz, Chloroform-*d*) δ 7.91 (s, 1H), 7.46 (s, 1H), 7.38 (d, *J* = 8.5 Hz, 1H), 7.22 (d, *J* = 8.1 Hz, 2H), 7.10 (t, *J* = 8.2 Hz, 3H), 7.05 (d, *J* = 2.3 Hz, 1H), 2.34 (s, 3H), 1.75 (s, 6H); **^{13}C NMR** (175 MHz, Chloroform-*d*) δ 146.40, 136.25, 135.25, 130.10, 130.09, 128.94, 128.71, 126.22, 125.81, 121.50, 113.15, 82.70, 38.59, 30.77, 21.06. **HRMS** calculated for $C_{18}H_{19}IN$ $[M+H]^+$ 376.0557, found 376.0550.

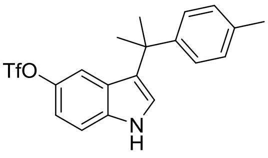

3-(2-(*p*-Tolyl)propan-2-yl)-1H-indol-5-yl trifluoromethanesulfonate (8i): Prepared according to the general procedure A, white solid, melting point: 108-110 °C, 56.8 mg, 72% yield, R_f = 0.4 (PE/EA = 5/1), **^{1}H NMR** (700 MHz, Chloroform-*d*) δ 8.10 (s, 1H), 7.31 (d, *J* = 8.8 Hz, 1H), 7.23 (d, *J* = 1.5 Hz, 1H), 7.20 (d, *J* = 8.0 Hz, 2H), 7.08 (d, *J* = 7.9 Hz, 2H), 7.00 (dd, *J* = 8.8, 1.9 Hz, 1H), 6.84 (s, 1H), 2.32 (s, 3H), 1.74 (s, 6H); **^{13}C NMR** (175 MHz, Chloroform-*d*) δ 146.05, 143.06, 135.92, 135.52, 128.99, 127.50, 126.39, 126.25, 122.94, 118.86 (q, *J* = 321.2 Hz), 115.09, 113.65, 111.98, 38.54, 30.65, 20.98; **^{19}F NMR** (376 MHz, Chloroform-*d*) δ -72.60. **HRMS** calculated for $C_{19}H_{19}F_3SO_3N$ $[M+H]^+$ 398.1032, found 398.1026.

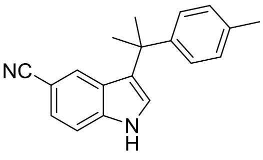

3-(2-(*p*-Tolyl)propan-2-yl)-1H-indole-5-carbonitrile (8j): Prepared according to the general procedure A, colorless oil, 28.1 mg, 51% yield, R_f = 0.3 (PE/EA = 5/1), **^{1}H NMR** (700 MHz, Chloroform-*d*) δ 8.47 (s, 1H),

7.41 (s, 1H), 7.39 (d, J = 8.4 Hz, 1H), 7.33 (d, J = 8.4 Hz, 1H), 7.26 (s, 1H), 7.19 (d, J = 8.1 Hz, 2H), 7.08 (d, J = 8.0 Hz, 2H), 2.33 (s, 3H), 1.75 (s, 6H); **^{13}C NMR** (175 MHz, Chloroform-*d*) δ 146.04, 138.93, 135.53, 129.08, 127.34, 126.99, 126.13, 125.92, 124.64, 122.73, 121.22, 112.12, 101.77, 38.55, 30.76, 21.03. **HRMS** calculated for $C_{19}H_{19}N_2$ [M+H]$^+$ 275.1543, found 275.1545.

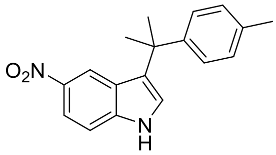

5-Nitro-3-(2-(*p*-tolyl)propan-2-yl)-1H-indole (8k): Prepared according to the general procedure A (48 h for the second step), yellow solid, melting point: 82-84 °C, 47.2 mg, 80% yield, R_f = 0.1 (PE/EA = 5/1), **^{1}H NMR** (700 MHz, DMSO-d_6) δ 11.66 (s, 1H), 7.89 (d, J = 8.9 Hz, 1H), 7.80 (s, 1H), 7.56 (s, 1H), 7.49 (d, J = 9.0 Hz, 1H), 7.17 (d, J = 7.9 Hz, 2H), 7.05 (d, J = 7.7 Hz, 2H), 2.22 (s, 3H), 1.69 (s, 6H); **^{13}C NMR** (175 MHz, DMSO-d_6) δ 146.26, 140.37, 139.65, 134.60, 128.73, 126.94, 125.87, 125.48, 124.65, 116.98, 116.20, 111.99, 38.09, 30.57, 20.50. **HRMS** calculated for $C_{18}H_{19}N_2O_2$ [M+H]$^+$ 295.1441, found 295.1437.

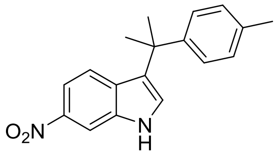

6-Nitro-3-(2-(*p*-tolyl)propan-2-yl)-1H-indole (8l): Prepared according to the general procedure A, yellow solid, melting point: 100-102 °C, 55.5 mg, 94% yield, R_f = 0.3 (PE/EA = 5/1), **^{1}H NMR** (700 MHz, Chloroform-*d*) δ 8.62 (s, 1H), 8.34 (d, J = 1.8 Hz, 1H), 7.77 (dd, J = 8.9, 1.8 Hz, 1H), 7.46 (d, J = 2.4 Hz, 1H), 7.20 (d, J = 8.1 Hz, 2H), 7.12-7.02 (m, 3H), 2.32 (s, 3H), 1.77 (s, 6H); **^{13}C NMR** (175 MHz, Chloroform-*d*) δ 146.16, 142.92, 135.68, 135.52, 130.95, 129.04, 127.52, 126.64, 126.22, 121.15, 114.44, 108.20, 38.60, 30.72, 21.04. **HRMS** calculated for $C_{18}H_{19}N_2O_2$ [M+H]$^+$ 295.1441, found 295.1436.

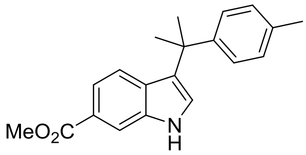

Methyl 3-(2-(*p*-tolyl)propan-2-yl)-1H-indole-6-carboxylate (8m): Prepared according to the general procedure A, white solid, melting point: 165-167 °C, 47.4 mg, 77% yield, R_f = 0.2 (PE/EA = 10/1), **^{1}H NMR** (700 MHz, Chloroform-*d*) δ 8.43 (s, 1H), 8.13 (s, 1H), 7.60 (dd, J = 8.7 Hz, 1.3 Hz, 1H), 7.28 (d, J = 2.4 Hz, 1H), 7.22 (d, J = 8.1 Hz, 2H), 7.12-7.05 (m, 3H), 3.92 (s, 3H), 2.32 (s, 3H), 1.78 (s, 6H); **^{13}C NMR** (175 MHz, Chloroform-*d*) δ 168.43, 146.63, 136.56, 135.21, 129.74, 128.90, 126.73, 126.30, 124.15, 123.25, 120.94, 119.91, 113.64, 52.04, 38.63, 30.76, 21.03. **HRMS** calculated for $C_{20}H_{22}NO_2$ [M+H]$^+$ 308.1645, found 308.1648.

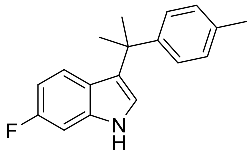

6-Fluoro-3-(2-(*p*-tolyl)propan-2-yl)-1H-indole (8n): Prepared according to the general procedure A, yellow oil, 47.6 mg, 89% yield, R_f = 0.3 (PE/EA = 20/1), **^{1}H NMR** (400 MHz, Chloroform-*d*) δ 7.87 (s, 1H), 7.25 (d, J = 7.6 Hz, 2H), 7.13-7.07 (m, 3H), 7.04-6.96 (m, 2H), 6.68 (td, J = 9.7, 2.3 Hz, 1H), 2.34 (s, 3H), 1.77 (s, 6H); **^{13}C NMR** (100 MHz, Chloroform-*d*) δ 159.70 (d, J = 237.4 Hz), 146.76, 137.09 (d, J = 12.3 Hz), 135.15, 128.88, 126.43, 126.31, 122.71, 122.08 (d, J = 10.0 Hz), 120.85 (d, J = 3.5 Hz), 107.71 (d, J = 24.1 Hz), 97.34 (d, J = 25.8 Hz), 38.59, 30.76, 21.06; **^{19}F NMR** (376 MHz, Chloroform-*d*) δ -121.82. **HRMS** calculated for $C_{18}H_{19}FN$ [M+H]$^+$ 268.1496, found 268.1497.

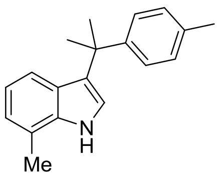

7-Methyl-3-(2-(*p*-tolyl)propan-2-yl)-1H-indole (8o): Prepared according to the general procedure A, yellow oil, 39.2 mg, 74% yield, R_f = 0.3 (PE/EA = 20/1), **^{1}H NMR** (700 MHz, Chloroform-*d*) δ 7.85 (s, 1H), 7.24 (d, J = 8.0 Hz, 2H),

7.13 (d, *J* = 2.1 Hz, 1H), 7.07 (d, *J* = 7.9 Hz, 2H), 6.95 (d, *J* = 8.0 Hz, 1H), 6.93 (d, *J* = 7.0 Hz, 1H), 6.83 (t, *J* = 7.5 Hz, 1H), 2.49 (s, 3H), 2.31 (s, 3H), 1.77 (s, 6H); **^{13}C NMR** (175 MHz, Chloroform-*d*) δ 147.06, 136.76, 134.96, 128.83, 126.89, 126.40, 125.68, 122.30, 120.34, 120.18, 119.31, 119.20, 38.74, 30.85, 21.08, 16.77. **HRMS** calculated for $C_{19}H_{22}N$ $[M+H]^+$ 264.1747, found 264.1749.

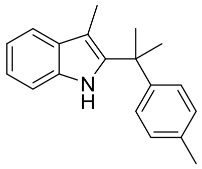

3-Methyl-2-(2-(*p*-tolyl)propan-2-yl)-1H-indole (8p): Prepared according to the general procedure A (80 °C for the second step), yellow oil, 30.4 mg, 58% yield, R_f = 0.5 (PE/EA = 20/1), **^{1}H NMR** (400 MHz, Chloroform-*d*) δ 7.79 (s, 1H), 7.52 (d, *J* = 7.4 Hz, 1H), 7.29 (d, *J* = 7.2 Hz, 1H), 7.22 (d, *J* = 8.2 Hz, 2H), 7.18-7.10 (m, 4H), 2.37 (s, 3H), 2.09 (s, 3H), 1.81 (s, 6H); **^{13}C NMR** (100 MHz, Chloroform-*d*) δ 145.85, 141.39, 135.79, 134.11, 130.44, 129.10, 126.28, 121.17, 119.13, 118.08, 110.41, 106.71, 40.31, 29.79, 21.08, 9.87. **HRMS** calculated for $C_{19}H_{22}N$ $[M+H]^+$ 264.1747, found 264.1754.

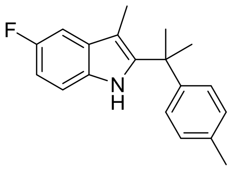

5-Fluoro-3-methyl-2-(2-(*p*-tolyl)propan-2-yl)-1H-indole (8q): Prepared according to the general procedure A (80 °C for the second step), yellow oil, 38.2 mg, 68% yield, R_f = 0.5 (PE/EA = 20/1), **^{1}H NMR** (400 MHz, Chloroform-*d*) δ 7.79 (s, 1H), 7.24-7.10 (m, 6H), 6.89 (td, *J* = 9.0, 2.5 Hz, 1H), 2.37 (s, 3H), 2.02 (s, 3H), 1.80 (s, 6H); **^{13}C NMR** (100 MHz, Chloroform-*d*) δ 157.90 (d, *J* = 233.6 Hz), 145.52, 143.54, 135.93, 130.92 (d, *J* = 9.4 Hz), 130.52, 129.17, 126.22, 110.95 (d, *J* = 9.7 Hz), 109.22 (d, *J* = 26.2 Hz), 106.97 (d, *J* = 4.6 Hz), 103.03 (d, *J* = 23.2 Hz), 40.34, 29.63, 21.08, 9.91; **^{19}F NMR** (376 MHz, Chloroform-*d*) δ -125.21. **HRMS** calculated for $C_{19}H_{21}FN$ $[M+H]^+$ 282.1653, found 282.1656.

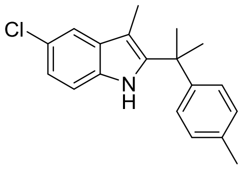

5-Chloro-3-methyl-2-(2-(*p*-tolyl)propan-2-yl)-1H-indole (8r): Prepared according to the general procedure A (80 °C for the second step), yellow oil, 48.1 mg, 81% yield, R_f = 0.5 (PE/EA = 20/1), **^{1}H NMR** (400 MHz, Chloroform-*d*) δ 7.82 (s, 1H), 7.47 (d, *J* = 1.7 Hz, 1H), 7.24-7.17 (m, 3H), 7.14 (d, *J* = 8.2 Hz, 2H), 7.09 (dd, *J* = 8.5, 1.9 Hz, 1H), 2.37 (s, 3H), 2.03 (s, 3H), 1.79 (s, 6H); **^{13}C NMR** (100 MHz, Chloroform-*d*) δ 145.42, 143.09, 135.96, 132.38, 131.62, 129.18, 126.19, 124.88, 121.28, 117.64, 111.39, 106.57, 40.32, 29.63, 21.08, 9.79. **HRMS** calculated for $C_{19}H_{21}{}^{35}ClN$ $[M+H]^+$ 298.1357, found 298.1366; $C_{19}H_{21}{}^{37}ClN$ $[M+H]^+$ 300.1328, found 300.1337.

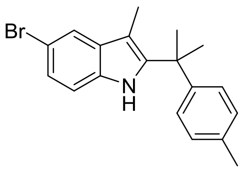

5-Bromo-3-methyl-2-(2-(*p*-tolyl)propan-2-yl)-1H-indole (8s): Prepared according to the general procedure A (80 °C for the second step), yellow oil, 50.1 mg, 73% yield, R_f = 0.5 (PE/EA = 20/1), **^{1}H NMR** (400 MHz, Chloroform-*d*) δ 7.82 (s, 1H), 7.62 (d, *J* = 1.8 Hz, 1H), 7.23-7.17 (m, 3H), 7.16-7.10 (m, 3H), 2.36 (s, 3H), 2.02 (s, 3H), 1.79 (s, 6H); **^{13}C NMR** (100 MHz, Chloroform-*d*) δ 145.40, 142.93, 135.98, 132.65, 132.28, 129.19, 126.19, 123.84, 120.75, 112.43, 111.85, 106.50, 40.31, 29.64, 21.08, 9.79. **HRMS** calculated for $C_{19}H_{21}{}^{79}BrN$ $[M+H]^+$ 342.0852, found 342.0858; $C_{19}H_{21}{}^{81}BrN$ $[M+H]^+$ 344.0831, found 344.0841.

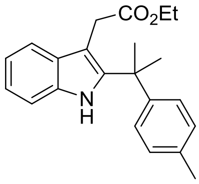

Ethyl 2-(2-(2-(*p*-tolyl)propan-2-yl)-1H-indol-3-yl)acetate (8t): Prepared according to the general procedure A (80 ºC for the second step), colorless oil, 32.6 mg, 49% yield, R_f = 0.5 (PE/EA = 5/1), **^{1}H NMR** (400 MHz, Chloroform-*d*) δ 7.91 (s, 1H), 7.53 (d, *J* = 7.7 Hz, 1H), 7.28 (d, *J* = 7.7 Hz, 1H), 7.21 (d, *J* = 8.2 Hz, 2H), 7.17-7.07 (m, 4H), 4.06 (q, *J* = 7.1 Hz, 2H), 3.48 (s, 2H), 2.34 (s, 3H), 1.80 (s, 6H), 1.19 (t, *J* = 7.1 Hz, 3H); **^{13}C NMR** (100 MHz, Chloroform-*d*) δ 172.16, 145.52, 143.17, 136.02, 134.19, 129.77, 129.20, 126.31, 121.62, 119.68, 118.54, 110.56, 104.27, 60.62, 40.38, 31.03, 30.17, 21.08, 14.31. **HRMS** calculated for $C_{22}H_{25}O_2NNa$ [M+Na]$^+$ 358.1778, found 358.1784.

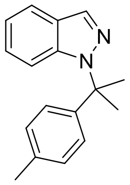

1-(2-(*p*-Tolyl)propan-2-yl)-1H-indazole (8u): Prepared according to the general procedure A, colorless oil, 22.5 mg, 45% yield, R_f = 0.6 (PE/EA = 20/1), **^{1}H NMR** (400 MHz, Chloroform-*d*) δ 8.05 (d, *J* = 0.8 Hz, 1H), 7.75-7.69 (m, 1H), 7.11 (s, 4H), 7.07-7.01 (m, 2H), 6.71-6.62 (m, 1H), 2.34 (s, 3H), 2.03 (s, 6H); **^{13}C NMR** (100 MHz, Chloroform-*d*) δ 143.05, 138.27, 136.65, 131.70, 129.13, 125.29, 125.17, 125.05, 120.71, 120.02, 112.20, 63.44, 29.53, 20.84. **HRMS** calculated for $C_{17}H_{19}N_2$ [M+H]$^+$ 251.1543, found 251.1545.

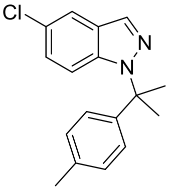

5-Chloro-1-(2-(*p*-tolyl)propan-2-yl)-1H-indazole (8v): Prepared according to the general procedure A, colorless oil, 30.6 mg, 54% yield, R_f = 0.5 (PE/EA = 20/1), **^{1}H NMR** (400 MHz, Chloroform-*d*) δ 7.98 (d, *J* = 0.8 Hz, 1H), 7.67 (d, *J* = 1.5 Hz, 1H), 7.13 (d, *J* = 8.2 Hz, 2H), 7.08 (d, *J* = 8.4 Hz, 2H), 6.98 (dd, *J* = 9.0, 2.0 Hz, 1H), 6.55 (d, *J* = 9.0 Hz, 1H), 2.34 (s, 3H), 2.01 (s, 6H); **^{13}C NMR** (100 MHz, Chloroform-*d*) δ 142.92, 137.25, 137.06, 131.37, 129.54, 126.40, 126.12, 126.05, 125.44, 120.10, 113.46, 64.04, 29.79, 21.15. **HRMS** calculated for $C_{17}H_{18}{}^{35}ClN_2$ [M+H]$^+$ 285.1153, found 285.1143; $C_{17}H_{18}{}^{37}ClN_2$ [M+H]$^+$ 287.1124, found 287.1114.

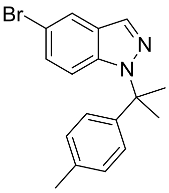

5-Bromo-1-(2-(*p*-tolyl)propan-2-yl)-1H-indazole (8w): Prepared according to the general procedure A, colorless oil, 30.5 mg, 46% yield, R_f = 0.5 (PE/EA = 20/1), **^{1}H NMR** (400 MHz, Chloroform-*d*) δ 7.98 (d, *J* = 0.7 Hz, 1H), 7.84 (d, *J* = 1.4 Hz, 1H), 7.17-7.04 (m, 5H), 6.50 (d, *J* = 9.0 Hz, 1H), 2.34 (s, 3H), 2.00 (s, 6H); **^{13}C NMR** (100 MHz, Chloroform-*d*) δ 142.89, 137.26, 131.25, 129.55, 128.46, 127.11, 125.44, 123.39, 113.82, 113.65, 64.06, 29.79, 21.16. **HRMS** calculated for $C_{17}H_{18}{}^{79}BrN_2$ [M+H]$^+$ 329.0648, found 329.0657; $C_{17}H_{18}{}^{81}BrN_2$ [M+H]$^+$ 331.0627, found 331.0625.

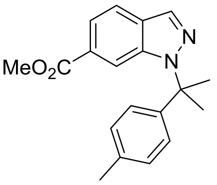

Methyl 1-(2-(*p*-tolyl)propan-2-yl)-1H-indazole-6-carboxylate (8x): Prepared according to the general procedure A (N^1/N^2 = 13:1), colorless oil, 31.9 mg, 52% yield, R_f = 0.4 (PE/EA = 5/1), **^{1}H NMR** (400 MHz, Chloroform-*d*) δ 8.08 (s, 1H), 7.75-7.68 (m, 2H), 7.46 (s, 1H), 7.17-7.04 (m, 4H), 3.83 (s, 3H), 2.33 (s, 3H), 2.06 (s, 6H); **^{13}C NMR** (100 MHz, Chloroform-*d*) δ 167.44, 142.86, 138.08, 137.26, 131.94, 129.53, 128.12, 127.12, 125.39, 120.93, 120.81, 114.88, 64.34, 52.27, 30.00, 21.16. **HRMS** calculated for $C_{19}H_{21}O_2N_2$ [M+H]$^+$ 309.1598, found 309.1594.

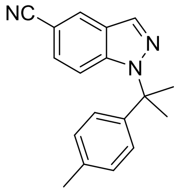

1-(2-(*p*-Tolyl)propan-2-yl)-1H-indazole-5-carbonitrile (8y): Prepared according to the general procedure A (N^1/N^2 = 3:1), white solid, melting point: 107-109 °C, 31.5 mg, 57% yield, R_f = 0.4 (PE/EA = 5/1), **^{1}H NMR** (400 MHz, Chloroform-*d*) δ 8.14 (d, *J* = 0.8 Hz, 1H), 8.12-8.07 (m, 1H), 7.21 (dd, *J* = 8.9, 1.5 Hz, 1H), 7.14 (d, *J* = 8.1 Hz, 2H), 7.08 (d, *J* = 8.3 Hz, 2H), 6.67 (d, *J* = 8.9 Hz, 1H), 2.34 (s, 3H), 2.02 (s, 6H); **^{13}C NMR** (100 MHz, Chloroform-*d*) δ 142.36, 139.34, 137.58, 132.95, 129.68, 127.47, 127.40, 125.37, 125.08, 119.81, 113.42, 103.89, 64.57, 29.81, 21.15. **HRMS** calculated for $C_{18}H_{18}N_3$ $[M+H]^+$ 276.1495, found 276.1501.

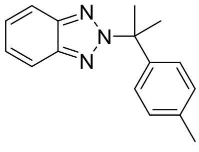

2-(2-(*p*-Tolyl)propan-2-yl)-2H-benzo[d][1,2,3]triazole (8z): Prepared according to the general procedure A (N^2/N^1 = 3:1), white solid, melting point: 99-101 °C, 18.7 mg, 37% yield, R_f = 0.6 (PE/EA = 20/1), **^{1}H NMR** (700 MHz, Chloroform-*d*) δ 7.91-7.86 (m, 2H), 7.40-7.35 (m, 2H), 7.09 (d, *J* = 8.1 Hz, 2H), 7.01 (d, *J* = 8.2 Hz, 2H), 2.30 (s, 3H), 2.24 (s, 6H); **^{13}C NMR** (100 MHz, Chloroform-*d*) δ 144.08, 142.59, 137.38, 129.31, 126.22, 124.84, 118.45, 69.43, 29.89, 21.10. **HRMS** calculated for $C_{16}H_{18}N_3$ $[M+H]^+$ 252.1495, found 252.1498.

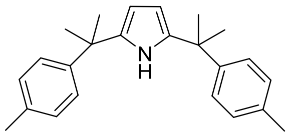

2,5-Bis(2-(*p*-tolyl)propan-2-yl)-1H-pyrrole (8aa): Prepared according to the general procedure A (**1aa** (0.10 mmol), filtering was required after the first step), colorless oil, 20.5 mg, 62% yield, R_f = 0.6 (PE/EA = 20/1), **^{1}H NMR** (400 MHz, Chloroform-*d*) δ 7.17 (s, 1H), 7.05 (s, 8H), 6.01 (d, *J* = 2.7 Hz, 2H), 2.30 (s, 6H), 1.59 (s, 12H); **^{13}C NMR** (100 MHz, Chloroform-*d*) δ 146.67, 139.77, 135.52, 128.88, 126.19, 103.65, 39.18, 30.30, 20.99. **HRMS** calculated for $C_{24}H_{30}N$ $[M+H]^+$ 332.2373, found 332.2378.

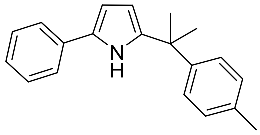

2-Phenyl-5-(2-(*p*-tolyl)propan-2-yl)-1H-pyrrole (8bb): Prepared according to the general procedure A (Filtering was required after the first step), colorless oil, 30.4 mg, 55% yield, R_f = 0.5 (PE/EA = 20/1), **^{1}H NMR** (700 MHz, Chloroform-*d*) δ 7.84 (s, 1H), 7.39-7.34 (m, 2H), 7.32-7.27 (m, 2H), 7.19 (d, *J* = 8.3 Hz, 2H), 7.17-7.09 (m, 3H), 6.45 (dd, *J* = 3.4, 2.7 Hz, 1H), 6.17 (dd, *J* = 3.4, 2.7 Hz, 1H), 2.33 (s, 3H), 1.70 (s, 6H); **^{13}C NMR** (100 MHz, Chloroform-*d*) δ 145.95, 142.15, 135.92, 133.01, 131.25, 129.18, 128.87, 126.32, 125.88, 123.58, 106.43, 105.53, 39.27, 30.30, 21.04. **HRMS** calculated for $C_{20}H_{22}N$ $[M+H]^+$ 276.1747, found 276.1742.

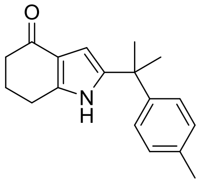

2-(2-(*p*-Tolyl)propan-2-yl)-1,5,6,7-tetrahydro-4H-indol-4-one (8cc): Prepared according to the general procedure A, white solid, melting point: 240-242 °C, 37.6 mg, 70% yield, R_f = 0.2 (PE/EA = 2/1), **^{1}H NMR** (700 MHz, Chloroform-*d*) δ 8.01 (s, 1H), 7.15 (d, *J* = 8.3 Hz, 2H), 7.10 (d, *J* = 8.2 Hz, 2H), 6.42 (d, *J* = 2.5 Hz, 1H), 2.68 (t, *J* = 6.2 Hz, 2H), 2.41 (t, *J* = 6.1 Hz, 2H), 2.32 (s, 3H), 2.09 (p, *J* = 6.3 Hz, 2H), 1.63 (s, 6H); **^{13}C NMR** (100 MHz, Chloroform-*d*) δ 194.75, 144.88, 143.70, 142.08, 136.08, 129.21, 126.28, 120.01, 101.30, 38.91, 37.75, 29.79, 24.02, 22.86, 21.02. **HRMS** calculated for $C_{18}H_{22}NO$ $[M+H]^+$ 268.1696, found 268.1698.

3.3 General procedure B: I_2 catalyzed nucleophilic aromatization from terpenes

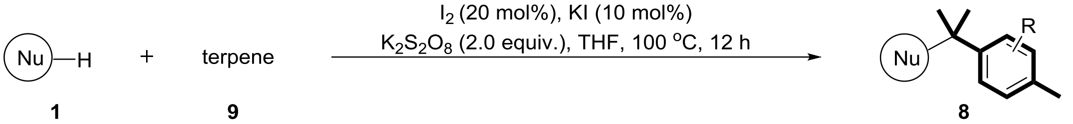

In a glove box, a sealed tube was charged with KI (0.02 mmol, 10 mol%), $K_2S_2O_8$ (0.40 mmol, 2.0 equiv.), nucleophile **1** (0.20 mmol), I_2 (0.04 mmol, 20 mol%), **9** (0.30 mmol) and THF (0.50 mL) at room temperature. The reaction tube was sealed with a Teflon screw cap, removed from the glove box. Then, the reaction mixture was stirred at 100 ℃ for 12 hours. Direct purification by column chromatography on silica gel using petroleum ether and ethyl acetate afforded the corresponding product **8**.

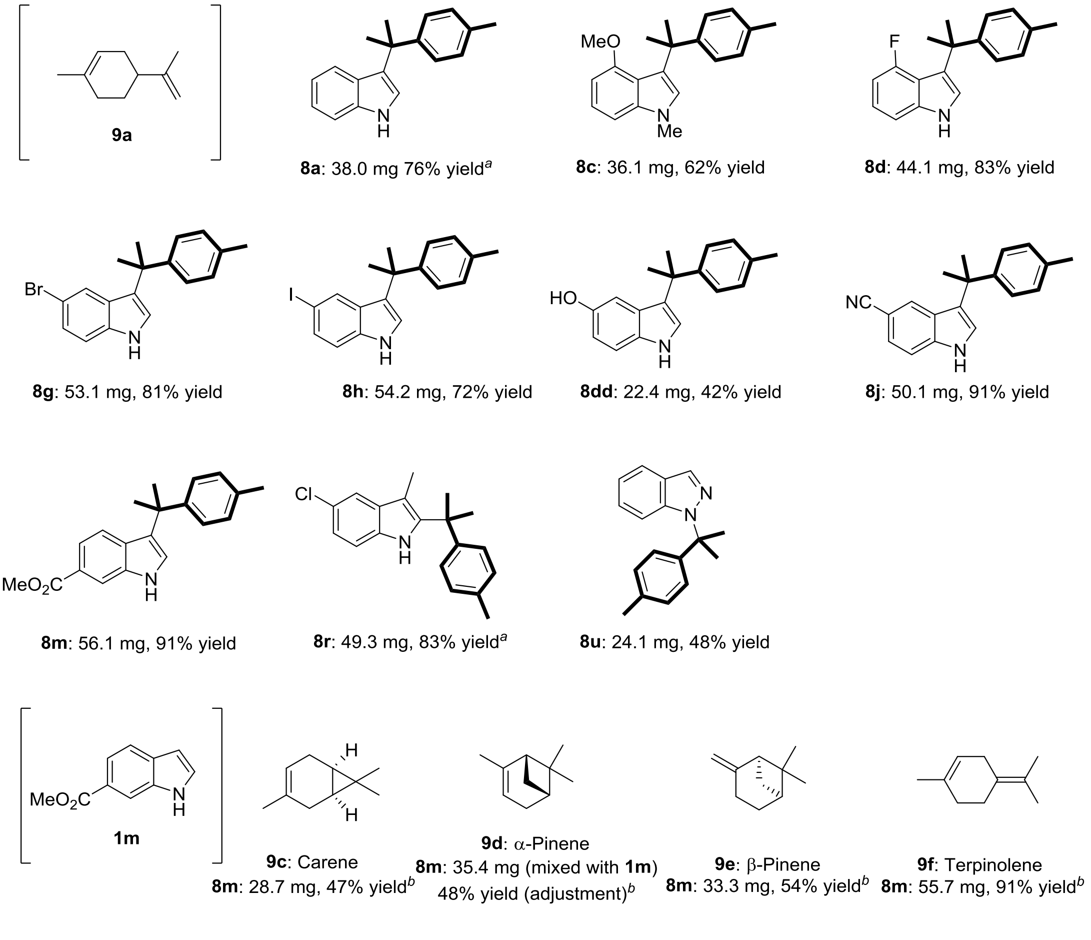

Conditions: **1** (0.20 mmol), **9** (0.30 mmol), I_2 (20 mol%), KI (10 mol%), $K_2S_2O_8$ (2.0 equiv.), THF (0.5 mL), 100 °C, 12 h. [a]6 h; [a]80 °C, 18 h; [b]**9** (0.40 mmol), $K_2S_2O_8$ (2.5 equiv.), 24 h.

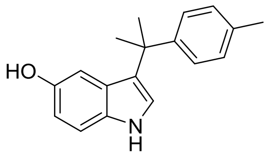

3-(2-(*p*-Tolyl)propan-2-yl)-1H-indol-5-ol (8dd): Prepared according to the general procedure B (limonene as **9**), yellow oil, 22.4 mg, 42% yield, R_f = 0.2 (PE/EA = 5/1), **^{1}H NMR** (700 MHz, Chloroform-*d*) δ 7.81 (s, 1H), 7.23 (d, *J* = 7.9 Hz, 2H), 7.18 (d, *J* = 8.6 Hz, 1H), 7.10 (s, 1H), 7.07 (d, *J* = 7.8 Hz, 2H), 6.69 (d, *J* = 8.6 Hz, 1H), 6.46 (s, 1H), 4.34 (s, 1H), 2.31 (s, 3H), 1.73 (s, 6H); **^{13}C NMR** (175 MHz, Chloroform-*d*) δ 148.60, 146.82, 135.05, 132.55, 128.90, 126.77, 126.38, 125.66, 121.83, 111.74,

111.49, 106.00, 38.60, 30.68, 21.06. **HRMS** calculated for $C_{18}H_{20}NO$ $[M+H]^+$ 266.1539, found 266.1538.

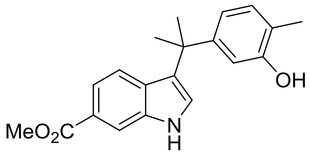

Methyl 3-(2-(3-hydroxy-4-methylphenyl)propan-2-yl)-1H-indole-6-carboxylate (8ee): Prepared according to the general procedure B (DMSO (0.40 mmol, 2.0 equiv.) was used instead of $K_2S_2O_8$, THF/$MeNO_2$ (0.5/0.5 mL), 24 h), pink oil, 45.7 mg, 71% yield from **9f**, 28.1 mg, 43% yield from **9g**, R_f = 0.3 (PE/EA = 3/1). **^{1}H NMR** (400 MHz, Chloroform-*d*) δ 8.24 (s, 1H), 8.02 (s, 1H), 7.55 (dd, *J* = 8.5, 1.3 Hz, 1H), 7.22 (d, *J* = 2.4 Hz, 1H), 7.11 (d, *J* = 8.5 Hz, 1H), 7.02 (d, *J* = 7.8 Hz, 1H), 6.84 (dd, *J* = 7.8, 1.7 Hz, 1H), 6.69 (d, *J* = 1.6 Hz, 1H), 5.13 (s, 1H), 3.87 (s, 3H), 2.20 (s, 3H), 1.71 (s, 6H); **^{13}C NMR** (100 MHz, Chloroform-*d*) δ 168.57, 153.72, 149.15, 136.46, 130.71, 129.74, 126.54, 124.14, 123.14, 121.19, 120.95, 119.96, 118.41, 113.64, 113.51, 52.13, 38.64, 30.67, 15.50. **HRMS** calculated for $C_{20}H_{22}NO_3$ $[M+H]^+$ 324.1594, found 324.1598.

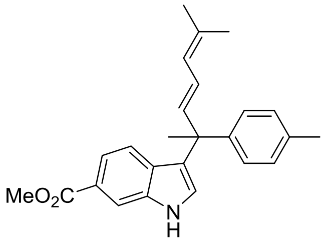

Methyl (*E*)-3-(6-methyl-2-(*p*-tolyl)hepta-3,5-dien-2-yl)-1H-indole-6-carboxylate (8ff): Prepared according to the general procedure B (18 h), an inseparable mixture of **8ff**, **1m** and **8m**, 32.5 mg in total, 31% yield of **8ff**, R_f = 0.4 (PE/EA = 5/1). NMR data for **8ff** was provided. **^{1}H NMR** (700 MHz, Chloroform-*d*) δ 8.23 (s, 1H), 8.12 (s, 1H), 7.76 (d, *J* = 8.5 Hz, 1H), 7.74 (d, *J* = 8.5 Hz, 1H), 7.32 (d, *J* = 8.0 Hz, 2H), 7.18 (d, *J* = 2.1 Hz, 1H), 7.12 (d, *J* = 7.9 Hz, 2H), 6.50-6.43 (m, 2H), 6.15-6.06 (m, 1H), 3.93 (s, 3H), 2.33 (s, 3H), 2.07 (s, 3H), 1.59 (s, 6H); **^{13}C NMR** (175 MHz, Chloroform-*d*) δ 168.32, 143.80, 140.47, 136.66, 136.54, 134.65, 129.06, 127.62, 126.61, 125.51, 124.90, 123.84, 123.69, 121.11, 120.44, 120.10, 113.69, 103.18, 52.08, 37.40, 28.85, 21.19, 16.14. **HRMS** calculated for $C_{25}H_{28}NO_2$ $[M]^+$ 374.2115, found 374.2118.

3.4 Convergent synthesis strategy

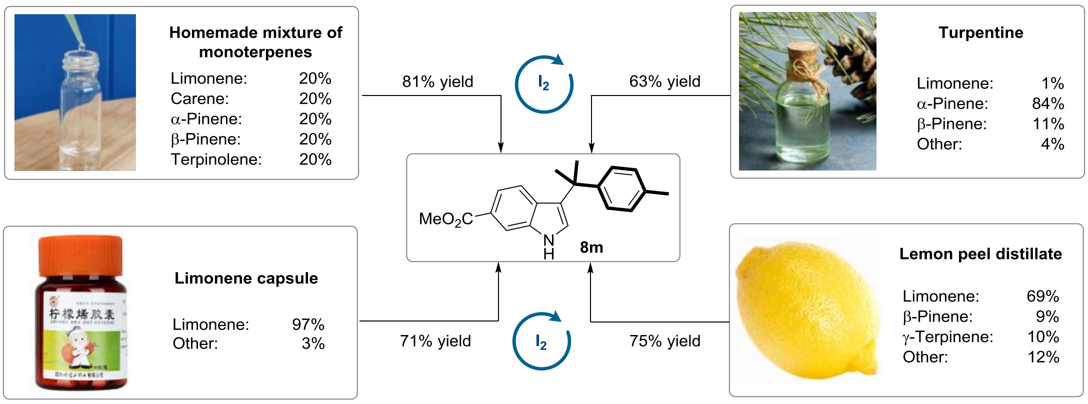

Supplementary Fig. 1. Convergent synthesis strategy

Under the air atmosphere, a sealed tube was charged with KI (0.02 mmol, 10 mol%), $K_2S_2O_8$ (0.40 mmol, 2.0 equiv.), indole compound **1m** (0.20 mmol), I_2 (0.04 mmol, 20 mol%), mixture of terpenes (54.5 mg) or a grain of capsule and THF (0.50 mL) at room temperature. The reaction tube was sealed with a Teflon screw cap, removed from the glove box. Then, the reaction mixture was stirred at 100 ℃ for 18-24 hours. Direct purification by column chromatography on silica gel using petroleum ether and ethyl acetate afforded the corresponding product **8m**.

3.5 General procedure C: I_2 catalyzed hydroarylation of olefins

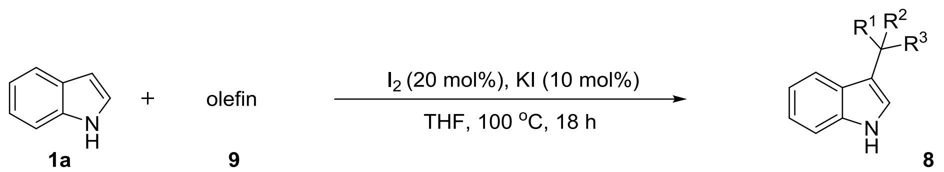

1a **9** **8**

In a glove box, a sealed tube was charged with KI (0.02 mmol, 10 mol%), indole **1a** (0.20 mmol), I_2 (0.04 mmol, 20 mol%), **9** (0.25 mmol) and THF (0.50 mL) at room temperature. The reaction tube was sealed with a Teflon screw cap, removed from the glove box. Then, the reaction mixture was stirred at 100 ℃ for 18 hours. Direct purification by column chromatography on silica gel using petroleum ether and ethyl acetate afforded the corresponding product **8**.

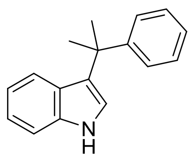

3-(2-Phenylpropan-2-yl)-1H-indole (8gg): Prepared according to the general procedure C, known compound,[1] yellow oil, 41.0 mg, 87% yield, R_f = 0.3 (PE/EA = 20/1). **^{1}H NMR** (400 MHz, Chloroform-*d*) δ 7.87 (s, 1H), 7.46-7.38 (m, 2H), 7.36 (d, *J* = 8.2 Hz, 1H), 7.34-7.28 (m, 2H), 7.25-7.20 (m, 1H), 7.16 (t, *J* = 7.6 Hz, 1H), 7.14-7.09 (m, 2H), 6.94 (ddd, *J* = 8.0, 7.0, 1.0 Hz, 1H), 1.84 (s, 6H); **^{13}C NMR** (100 MHz, Chloroform-*d*) δ 150.02, 137.22, 128.13, 126.50, 126.18, 126.12, 125.66, 121.72, 121.42, 120.70, 118.99, 111.17, 39.05, 30.76.

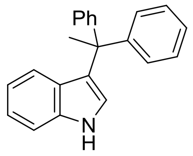

3-(1,1-Diphenylethyl)-1H-indole (8hh): Prepared according to the general procedure C, known compound,[1] yellow oil, 53.7 mg, 90% yield, R_f = 0.2 (PE/EA = 20/1). **^{1}H NMR** (400 MHz, Chloroform-*d*) δ 7.74 (s, 1H), 7.30-7.18 (m, 11H), 7.14-7.08 (m, 2H), 6.91 (td, *J* = 7.6, 0.9 Hz, 1H), 6.37 (d, *J* = 2.5 Hz, 1H), 2.25 (s, 3H); **^{13}C NMR** (100 MHz, Chloroform-*d*) δ 148.59, 137.20, 128.47, 127.96, 126.47, 126.01, 125.57, 123.81, 122.20, 121.80, 119.19, 111.29, 48.13, 29.47.

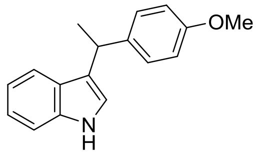

3-(1-(4-Methoxyphenyl)ethyl)-1H-indole (8ii): Prepared according to the general procedure C, known compound,[2] white solid, 47.7 mg, 95% yield, R_f = 0.3 (PE/EA = 20/1). **^{1}H NMR** (400 MHz, Chloroform-*d*) δ 7.85 (s, 1H), 7.35 (d, *J* = 7.9 Hz, 1H), 7.28 (d, *J* = 8.1 Hz, 1H), 7.19 (d, *J* = 8.6 Hz, 2H), 7.15-7.09 (m, 1H), 7.02-6.95 (m, 1H), 6.95-6.87 (m, 1H), 6.80 (d, *J* = 8.7 Hz, 2H), 4.31 (q, *J* = 7.1 Hz, 1H), 3.74 (s, 3H), 1.66 (d, *J* = 7.1 Hz, 3H); **^{13}C NMR** (100 MHz, Chloroform-*d*) δ 157.81, 139.14, 136.73, 128.43, 126.95, 122.01, 121.82, 121.10, 119.84, 119.24, 113.77, 111.13, 55.32, 36.19, 22.69.

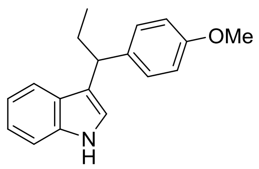

3-(1-(4-Methoxyphenyl)propyl)-1H-indole (8jj): Prepared according to the general procedure C, known compound,[2] colorless oil, 49.5 mg, 93% yield, R_f = 0.2 (PE/EA = 20/1). **^{1}H NMR** (400 MHz, Chloroform-*d*) δ 7.81 (s, 1H), 7.40 (d, *J* = 7.9 Hz, 1H), 7.23 (d, *J* = 8.1 Hz, 1H), 7.17 (d, *J* = 8.6 Hz, 2H), 7.10 (t, *J* = 7.6 Hz, 1H), 6.98 (t, *J* = 7.5 Hz, 1H), 6.91 (d, *J* = 2.1 Hz, 1H), 6.78 (d, *J* = 8.7 Hz, 2H), 4.04-3.90 (m, 1H), 3.71 (s, 3H), 2.24-2.10 (m, 1H), 2.04-1.86 (m, 1H), 0.91 (t, *J* = 7.3 Hz, 3H); **^{13}C NMR** (100 MHz, Chloroform-*d*) δ 157.78, 137.61, 136.60, 128.97, 127.18, 121.95, 120.97, 120.84, 119.68, 119.21, 113.69, 111.11, 55.29, 44.03, 29.25, 12.95.

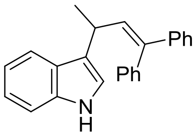

3-(4,4-Diphenylbut-3-en-2-yl)-1H-indole (8kk): Prepared according to the general procedure C, yellow oil, 57.8 mg, 89% yield, R_f = 0.2 (PE/EA = 20/1). **^{1}H NMR** (400 MHz, Chloroform-*d*) δ 7.77 (s, 1H), 7.49 (d, *J* = 7.9 Hz, 1H), 7.41-7.35 (m, 2H), 7.35-7.31 (m, 1H), 7.30-7.25 (m, 3H), 7.23-7.12 (m, 6H), 7.08-7.01 (m, 1H), 6.92 (d, *J* = 1.8 Hz, 1H), 6.24 (d, *J* = 10.3 Hz, 1H), 3.88 (dq, *J* = 10.4, 6.9 Hz, 1H), 1.49 (d, *J* = 6.8 Hz, 3H); **^{13}C NMR** (100 MHz, Chloroform-*d*) δ 142.85, 140.29, 139.84, 136.59, 134.81, 130.02, 128.40, 128.18, 127.56, 127.21, 127.01, 126.80, 122.01, 121.39, 120.07, 119.83, 119.24, 111.19, 31.58, 21.88. **HRMS** calculated for $C_{24}H_{22}N$ [M+H]$^+$ 324.1747, found 324.1741.

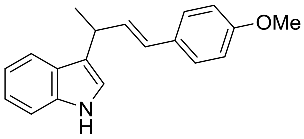

(*E*)-3-(4-(4-Methoxyphenyl)but-3-en-2-yl)-1H-indole (8ll): Prepared according to the general procedure C, known compound,[3] yellow solid, 28.9 mg, 52% yield, R_f = 0.2 (PE/EA = 20/1). **^{1}H NMR** (400 MHz, Chloroform-*d*) δ 7.95 (s, 1H), 7.70 (d, *J* = 7.9 Hz, 1H), 7.37 (d, *J* = 8.1 Hz, 1H), 7.30 (d, *J* = 8.7 Hz, 2H), 7.20 (t, *J* = 7.1 Hz, 1H), 7.10 (t, *J* = 7.1 Hz, 1H), 7.02 (d, *J* = 2.1 Hz, 1H), 6.84 (d, *J* = 8.7 Hz, 2H), 6.47 (d, *J* = 15.9 Hz, 1H), 6.34 (dd, *J* = 15.8, 6.9 Hz, 1H), 3.97-3.85 (m, 1H), 3.80 (s, 3H), 1.57 (d, *J* = 6.9 Hz, 3H); **^{13}C NMR** (100 MHz, Chloroform-*d*) δ 158.80, 136.68, 133.47, 130.73, 127.65, 127.35, 126.95, 122.07, 120.83, 120.49, 119.81, 119.33, 114.00, 111.23, 55.42, 34.36, 20.96.

4. Supplementary Note 3

4.1 Kinetics experiment for the dimerization of isoprene

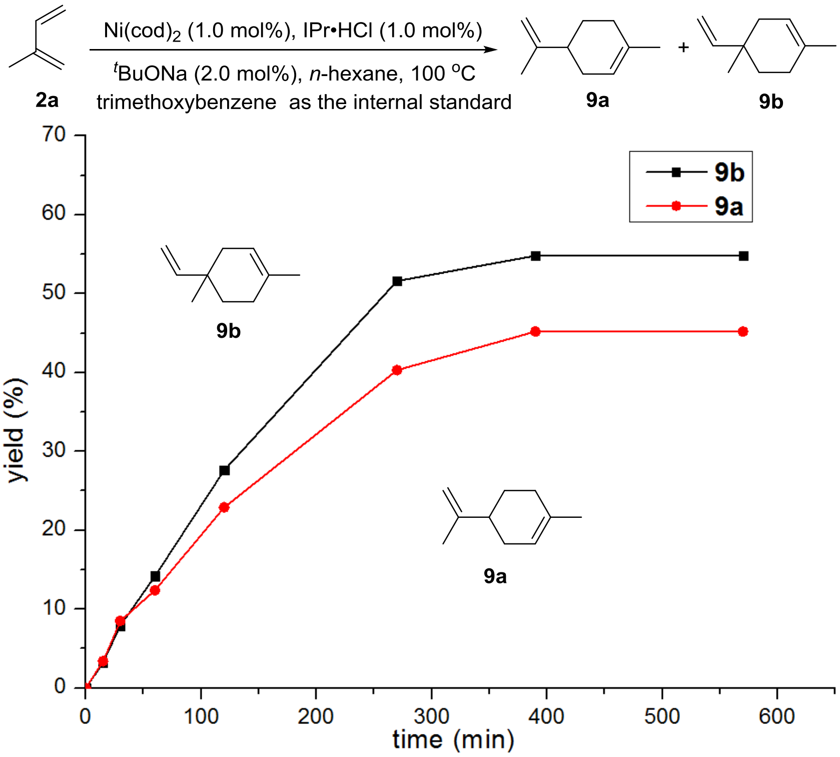

Supplementary Fig. 2. Kinetics experiment for the dimerization of isoprene

In a glove box, a sealed tube was charged with $Ni(cod)_2$ (0.084 mmol, 1.0 mol%), IPr HCl (0.084 mmol, 1.0 mol%), NaO^{t}Bu (0.168 mmol, 2.0 mol%), 1,3,5-trimethoxybenzene (0.10*7 mmol), **2a** (1.2*7 mmol) and *n*-hexane (5.0 mL) at room temperature. Then, the mixture was stirred to dissolve

and divided into 7 sealed tubes equally. These reaction tubes were sealed with a Teflon screw cap, removed from the glove box. The reaction mixture was stirred at 100 °C and detected by GC-FID at 15 min, 30 min, 60 min, 120 min, 270 min, 390 min, 570 min.

4.2 Kinetics experiment for the aromatization of terpenes

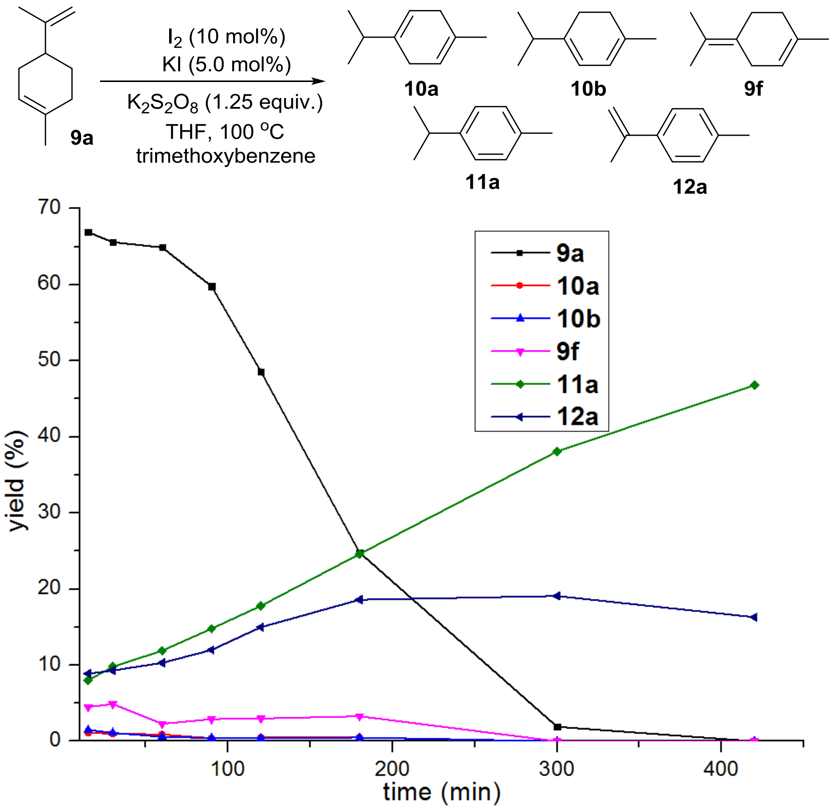

Supplementary Fig. 3. Kinetics experiment for the aromatization of limonene

Under air atmosphere, a sealed tube was charged with I_2 (0.08 mmol, 10 mol%), KI (0.04 mmol, 5.0 mol%), $K_2S_2O_8$ (1.0 mmol, 1.25 equiv.), 1,3,5-trimethoxybenzene (0.30 mmol), **9a** (0.80 mmol) and THF (1.5 mL) at room temperature. Then, the mixture was sealed with a Teflon screw cap, stirred at 100 ℃ and detected by GC-FID at 15 min, 30 min, 60 min, 90 min, 120 min, 180 min, 300 min, 420 min.

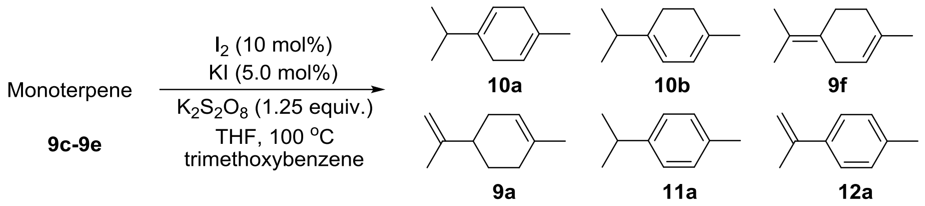

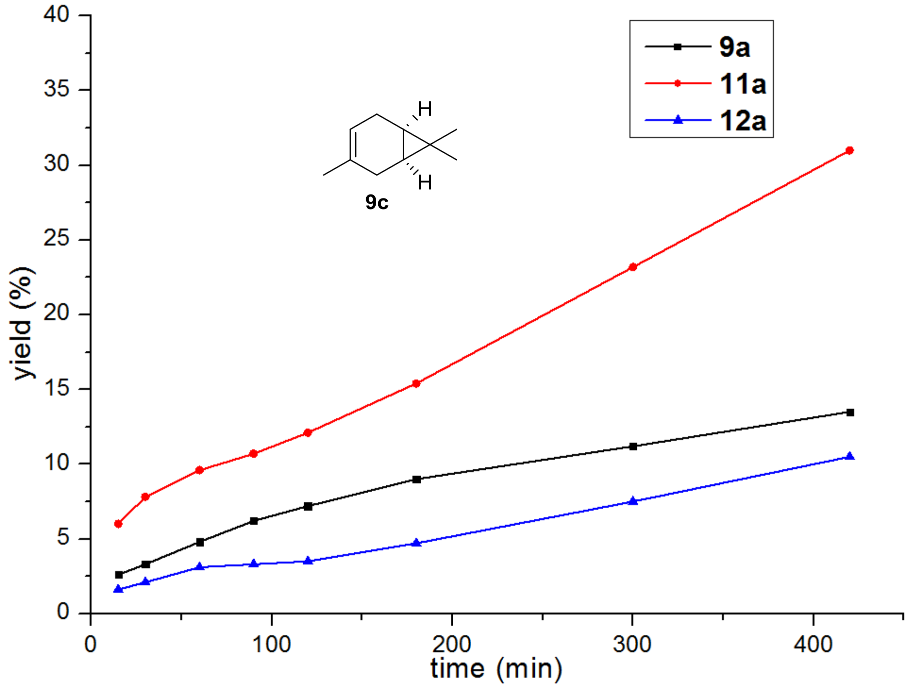

Supplementary Fig. 4. Kinetics experiment for the aromatization of carene

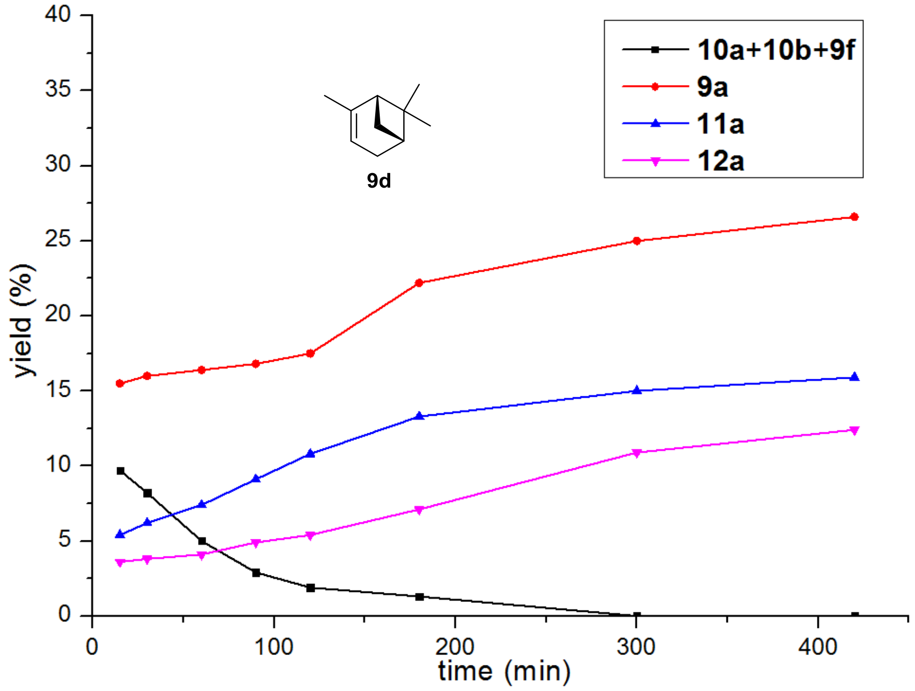

Supplementary Fig. 5. Kinetics experiment for the aromatization of α-pinene

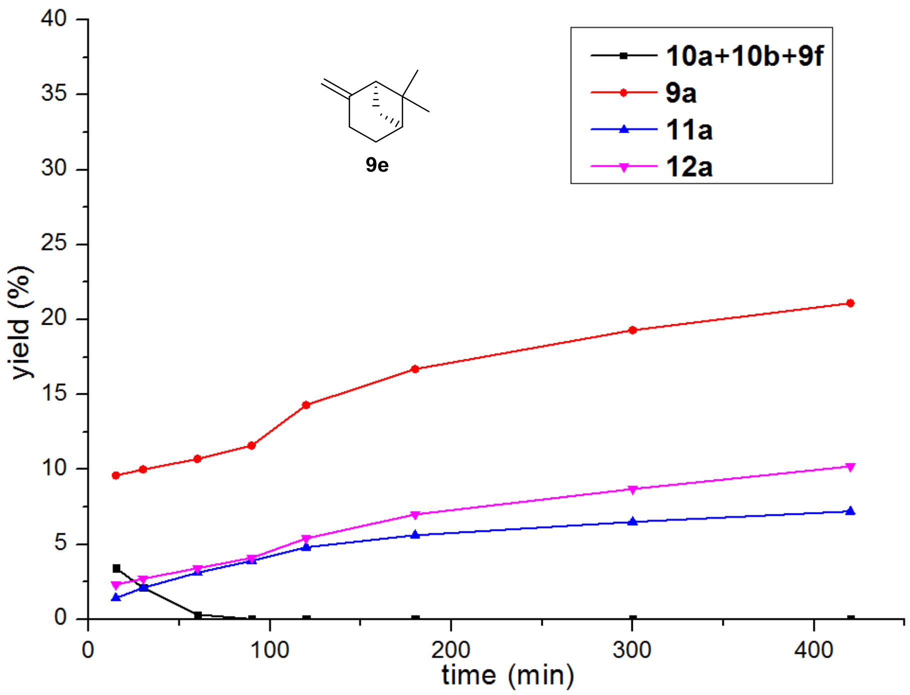

Supplementary Fig. 6. Kinetics experiment for the aromatization of β-pinene

Under air atmosphere, a sealed tube was charged with I_2 (0.08 mmol, 10 mol%), KI (0.04 mmol, 5.0 mol%), $K_2S_2O_8$ (1.0 mmol, 1.25 equiv.), 1,3,5-trimethoxybenzene (0.30 mmol), monoterpene (0.80 mmol) and THF (1.5 mL) at room temperature. Then, the mixture was sealed with a Teflon screw cap, stirred at 100 ℃ and detected by GC-FID at 15 min, 30 min, 60 min, 90 min, 120 min, 180 min, 300 min, 420 min.

On the basis of the above observations and previous work[4-6], plausible mechanisms were proposed. For carene, iodonium species **A'** is initially obtained from carene **9c** with the help of I_2. Through a nucleophilic attack by iodide, vicinal diiodide compound **B'** is generated, which gives the intermediate **C'** smoothly by elimination of H–I. With the iodonium species **D'** generation, a subsequent electron transfer yields a carbocation **E'**, which could be attacked by iodide ion to form species **F'**. Finally, olefin **12a** is formed from species **F'** via double elimination of H–I. The required I_2 is regenerated from the oxidation of H–I by persulfate.

Through the similar process of ring opening and di-iodination/HI elimination, olefin **12a** could obtained from α-pinene **9d** and β-pinene **9e** as well.

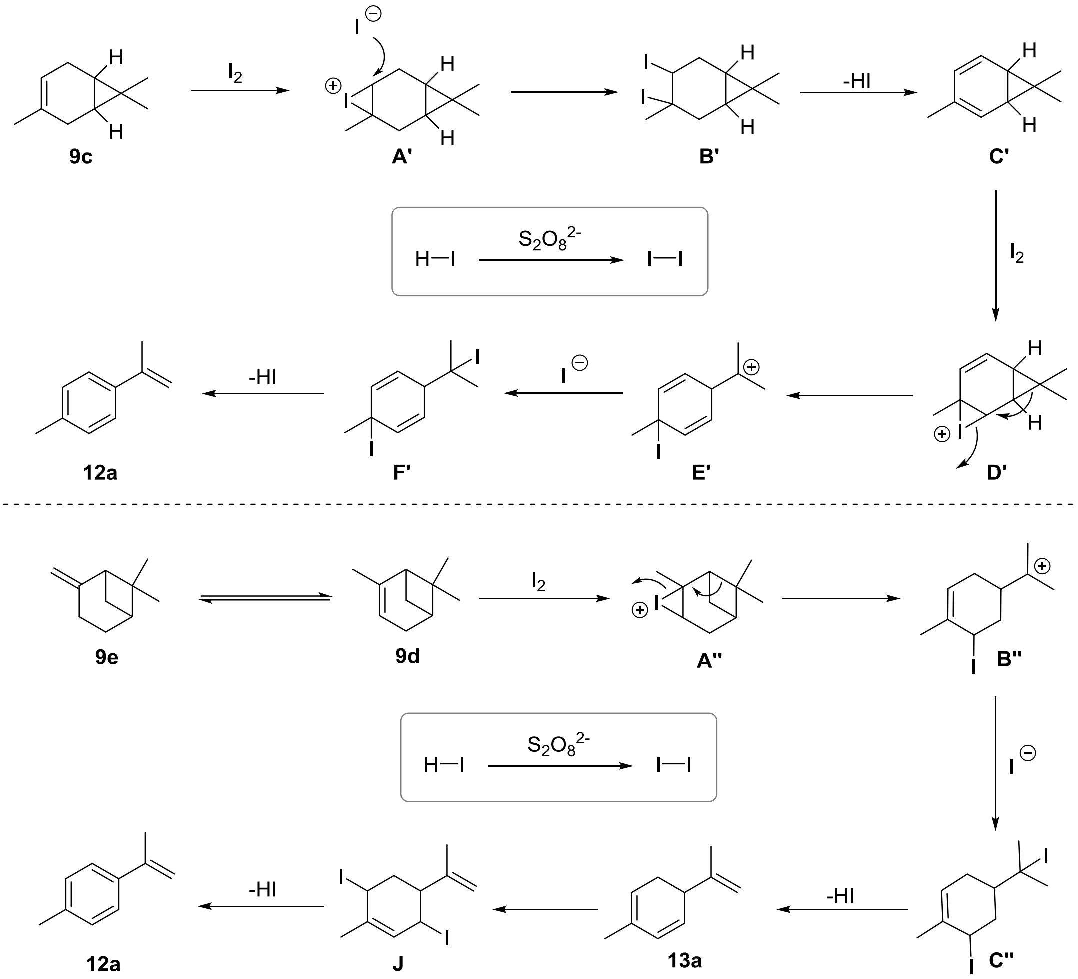

Supplementary Fig. 7. Proposed mechanism for the aromatization of monoterpenes

4.3 Deuterium labeling experiment

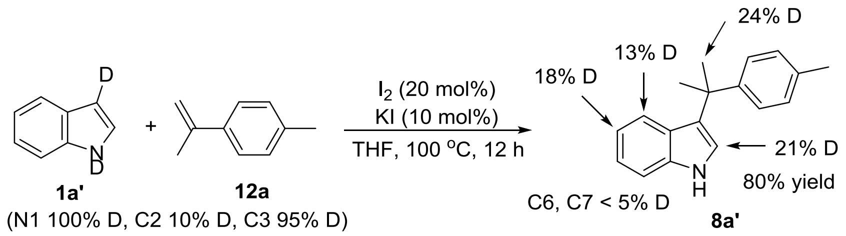

In a glove box, a sealed tube was charged with I_2 (0.04 mmol, 20 mol%), KI (0.02 mmol, 10 mol%), indole **1a’** (0.20 mmol), **12a** (0.30 mmol) and THF (0.50 mL) at room temperature. The reaction tube was sealed with a Teflon screw cap, removed from the glove box. Then, the reaction mixture was stirred at 100 ℃ for 12 hours. Direct purification by column chromatography on silica gel using petroleum ether and ethyl acetate afforded the corresponding product **8a'**.

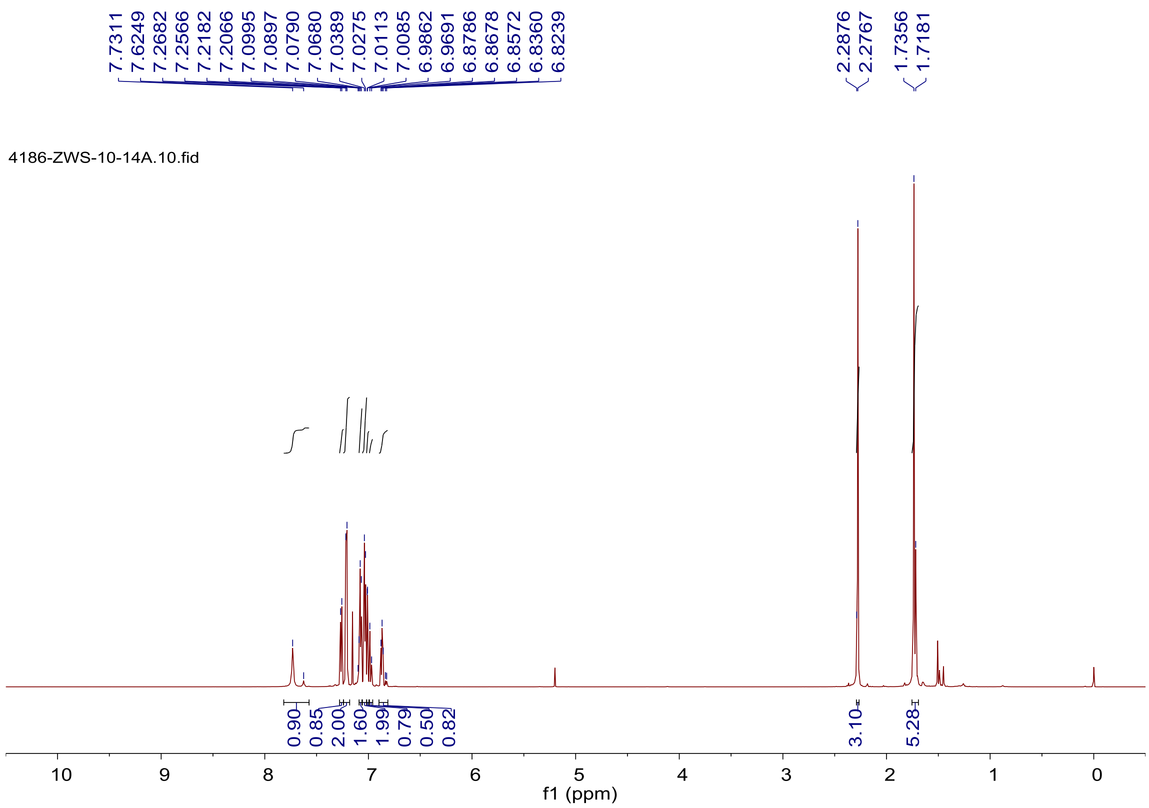
7.7311
7.6249
7.2682
7.2566
7.2182
7.2066
7.0995
7.0897
7.0790
7.0680
7.0389
7.0275
7.0113
7.0085
6.9862
6.9691
6.8786
6.8678
6.8572
6.8360
6.8239
2.2876
2.2767
1.7356
1.7181
4186-ZWS-10-14A.10.fid
0.90
0.85
2.00
1.60
1.99
0.79
0.50
0.82
3.10
5.28
f1 (ppm)

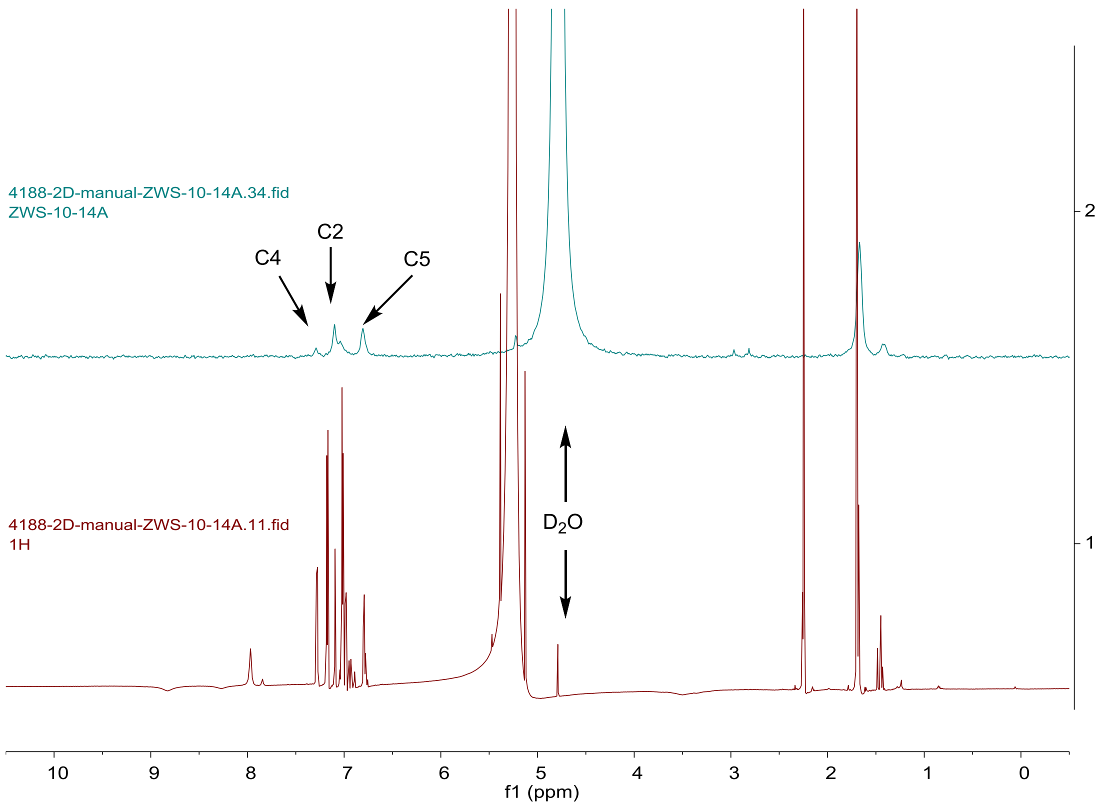
4188-2D-manual-ZWS-10-14A.34.fid
ZWS-10-14A
C4
C2
C5
4188-2D-manual-ZWS-10-14A.11.fid
1H
D2O
f1 (ppm)

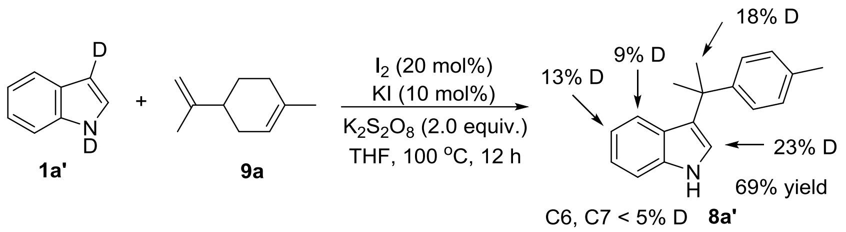

In a glove box, a sealed tube was charged with I_2 (0.04 mmol, 20 mol%), KI (0.02 mmol, 10 mol%), $K_2S_2O_8$ (0.40 mmol, 2.0 equiv.), indole **1a'** (0.20 mmol), **9a** (0.30 mmol) and THF (0.50 mL) at room temperature. The reaction tube was sealed with a Teflon screw cap, removed from the glove box. Then, the reaction mixture was stirred at 100 ℃ for 12 hours. Direct purification by column chromatography on silica gel using petroleum ether and ethyl acetate afforded the corresponding product **8a'**.

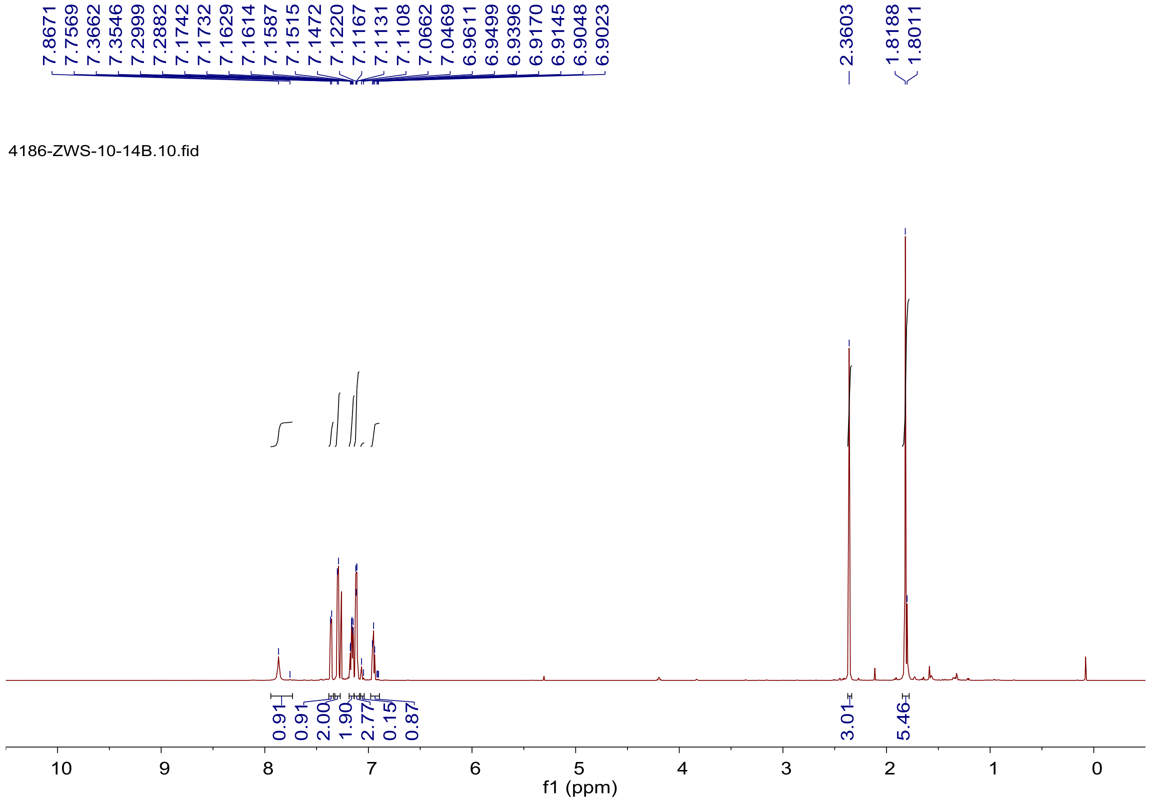

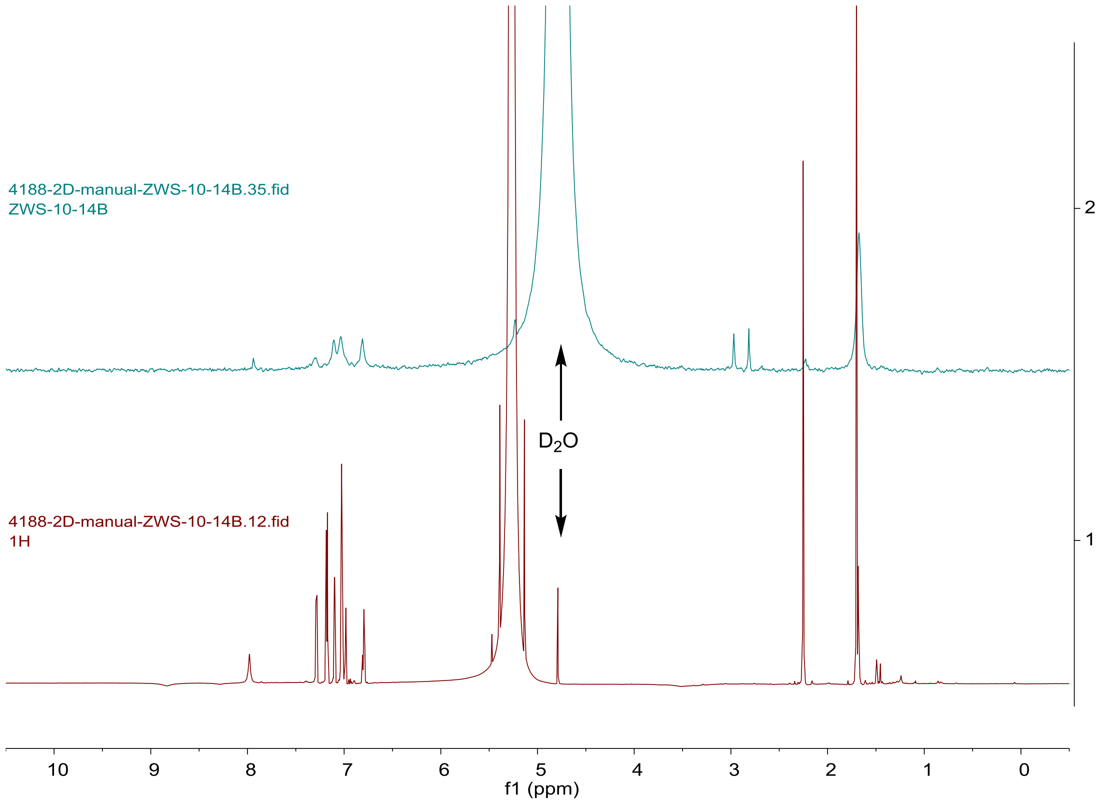

4.4 KIE experiment

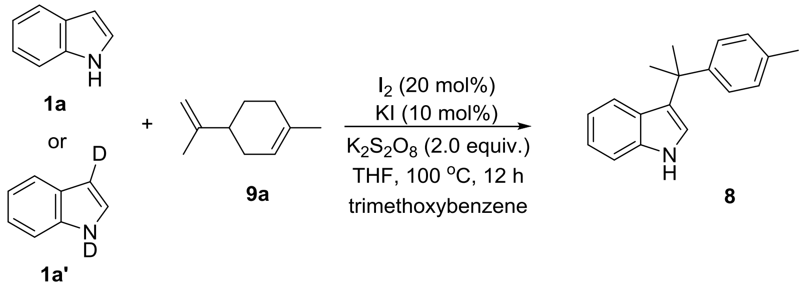

Under air atmosphere, a sealed tube was charged with I_2 (0.04 mmol, 20 mol%), KI (0.02 mmol, 10 mol%), $K_2S_2O_8$ (0.40 mmol, 2.0 equiv.), 1,3,5-trimethoxybenzene (0.10 mmol), indole **1a** or **1a'** (0.20 mmol), **9a** (0.30 mmol) and THF (0.5 mL) at room temperature. Then, the mixture was sealed with a Teflon screw cap, stirred at 100 ℃ and detected by GC-FID at 15 min, 30 min, 60 min, 90 min.

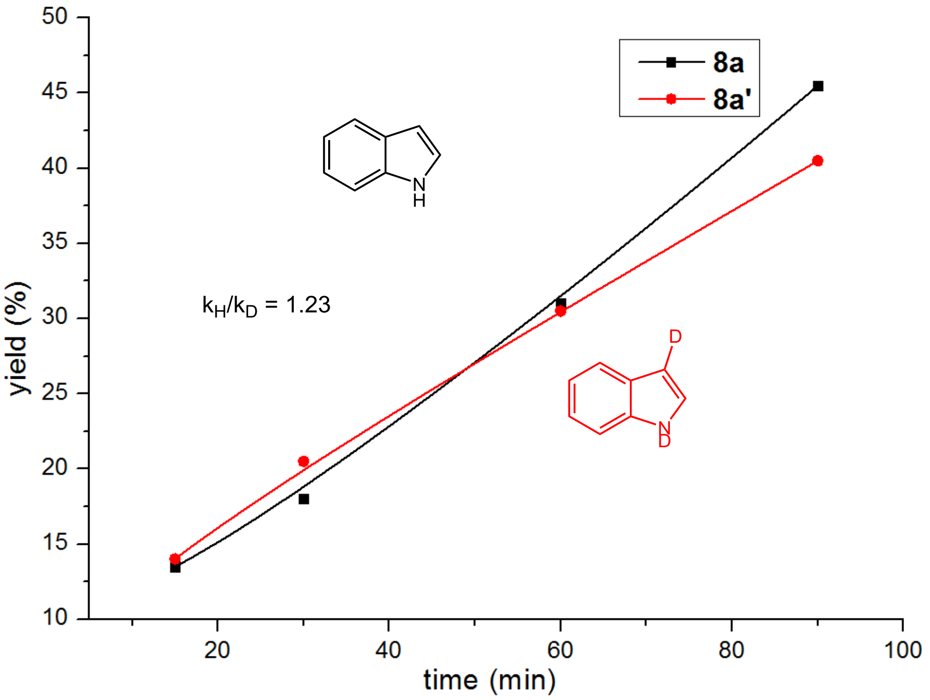

Supplementary Fig. 8. KIE experiment

4.5 Kinetics experiment for the coupling of indole and limonene

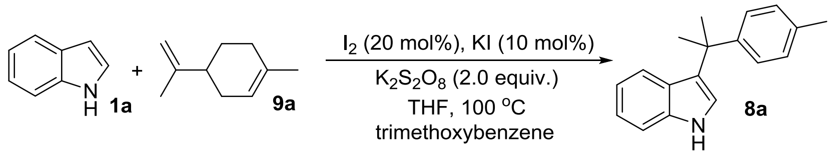

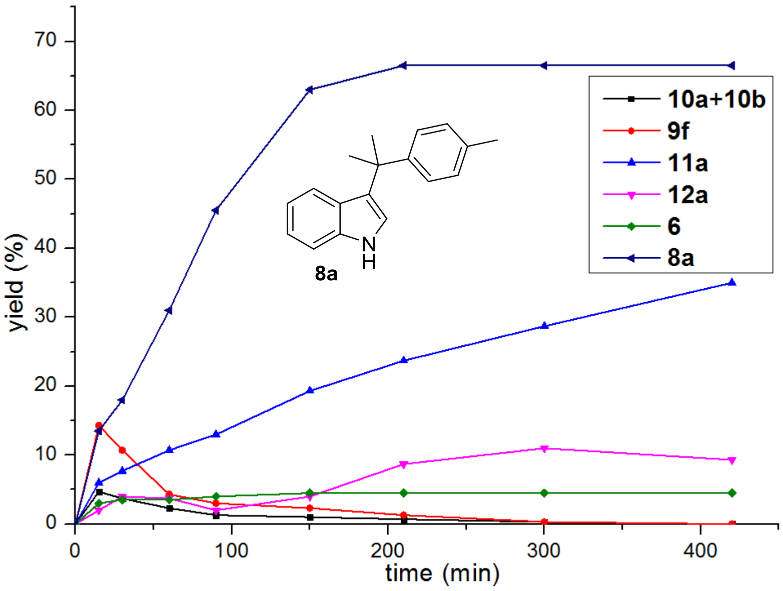

Supplementary Fig. 9. Kinetics experiment for the coupling of indole and limonene

Under air atmosphere, a sealed tube was charged with I_2 (0.04 mmol, 20 mol%), KI (0.02 mmol, 10 mol%), $K_2S_2O_8$ (0.40 mmol, 2.0 equiv.), 1,3,5-trimethoxybenzene (0.10 mmol), indole **1a** (0.20 mmol), **9a** (0.30 mmol) and THF (0.50 mL) at room temperature. Then, the mixture was sealed with a Teflon screw cap, stirred at 100 ℃ and detected by GC-FID at 15 min, 30 min, 60 min, 90 min, 150 min, 210 min, 300 min, 420 min.

4.6 Control experiments

Supplementary Table 9. Control experiment I

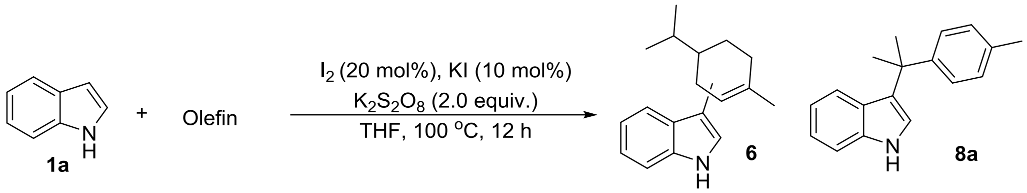

entry	Olefin	Yield of **6**	Yield of **8a**
1	**10a**	43%	7%
2	**10b**	6%	2%
3	**9f**	7%	89%
4	**13a**	0	21%
5	**12a**	0	35% (84%[a])
6	**11a**	0	0

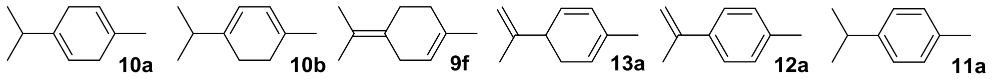

Condition: **1a** (0.20 mmol), olefin (0.30 mmol), I_2 (20 mol%), KI (10 mol%), $K_2S_2O_8$ (2.0 equiv.), THF (0.50 mL), 100 °C, 12 h. [a]HI (20 mol%) was added without I_2, KI and $K_2S_2O_8$.

In a glove box, a sealed tube was charged with I_2 (0.04 mmol, 20 mol%), KI (0.02 mmol, 10 mol%), $K_2S_2O_8$ (0.40 mmol, 2.0 equiv.), indole **1a** (0.20 mmol), olefin (0.30 mmol) and THF (0.50 mL) at room temperature. The reaction tube was sealed with a Teflon screw cap, removed from the glove box. Then, the reaction mixture was stirred at 100 ℃ for 12 hours. Yields were determined by GC-FID.

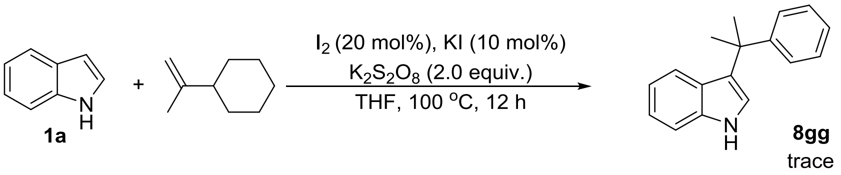

In a glove box, a sealed tube was charged with I_2 (0.04 mmol, 20 mol%), KI (0.02 mmol, 10 mol%), $K_2S_2O_8$ (0.40 mmol, 2.0 equiv.), indole **1a** (0.20 mmol), olefin (0.30 mmol) and THF (0.50 mL) at room temperature. The reaction tube was sealed with a Teflon screw cap, removed from the glove box. Then, the reaction mixture was stirred at 100 ℃ for 12 hours. Only the trace of yield of **8gg** was detected, which suggested that the C=C bond of cycle is necessary for the aromatization.

Supplementary Table 10. Control experiment II

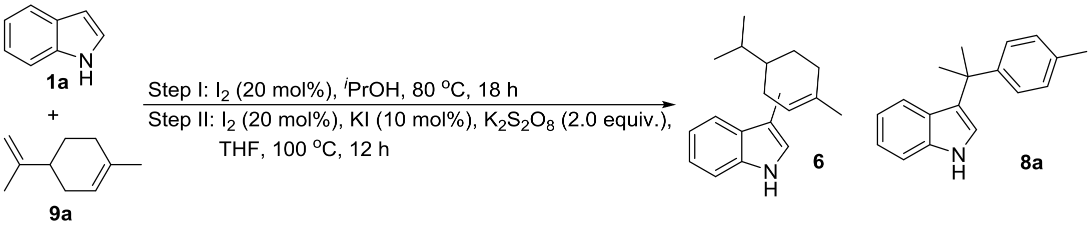

entry	Step	Yield of **6**	Yield of **8a**
1	Step I	61%	3%
2	Step II	26% (43% for step I)	3% (5% for step I)

Step I: In a glove box, a sealed tube was charged with I_2 (0.04 mmol, 20 mol%), indole **1a** (0.20 mmol), olefin (0.30 mmol) and ^{i}PrOH (0.50 mL) at room temperature. The reaction tube was sealed with a Teflon screw cap, removed from the glove box. Then, the reaction mixture was stirred at 80 ℃ for 18 hours. Yields were determined by GC-FID. Step II: The solvent was then evaporated and the residue was added into the mixture with I_2 (0.04 mmol, 20 mol%), KI (0.02 mmol, 10 mol%), $K_2S_2O_8$ (0.40 mmol, 2.0 equiv.), and THF (0.50 mL). Then, the reaction mixture was stirred at 100 ℃ for 12 hours. Yields were determined by GC-FID.

4.7 The role of KI

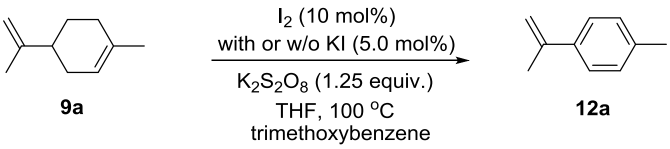

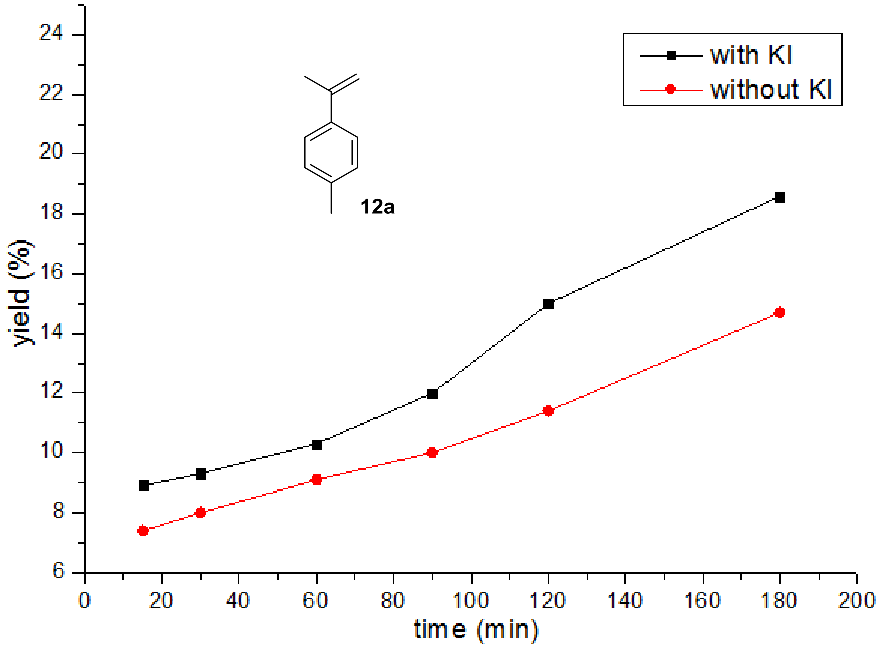

Supplementary Fig. 10. Kinetics experiments for the aromatization of limonene with or w/o KI

Under air atmosphere, a sealed tube was charged with I_2 (0.08 mmol, 10 mol%), KI (0.04 mmol, 5.0 mol%), $K_2S_2O_8$ (1.0 mmol, 1.25 equiv.), 1,3,5-trimethoxybenzene (0.30 mmol), **9a** (0.80 mmol) and THF (1.5 mL) at room temperature. Then, the mixture was sealed with a Teflon screw cap, stirred at

100 ℃ and detected by GC-FID at 15 min, 30 min, 60 min, 90 min, 120 min, 180 min.

The formation rate of **12a** in absence of KI was slower than that with KI. Meanwhile, based on the proposed mechanism (Please see page 9, Fig. 4e), intermediate **H** was formed from iodonium ion **G** and iodide ion. Thus, the addition of KI probably facilitates the generation of **H** or other related intermediates.

4.8 Synthesis and transformation of the possible intermediates

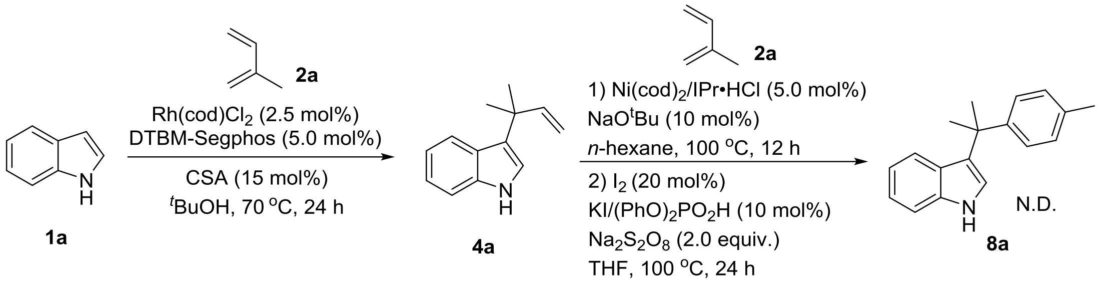

To a sealed tube (4 mL) was sequentially added indole **1a** (1.0 mmol), $[Rh(cod)Cl]_2$ (0.025 mmol, 2.5 mol%), DTBM-Segphos (0.05 mmol, 5.0 mol%), CSA (0.15 mmol, 15 mol%), ^{t}BuOH (2.0 mL), and isoprene **2a** (3.0 mmol). The resulting orange mixture was then stirred at 70 ºC for 24 h. The system was diluted with DCM and washed with water. The organic phase was dried over sodium sulfate and concentrated under reduced pressure. The residue was purified by flash column chromatography (silica gel, petroleum ether/EtOAc = 20:1) to reverse-prenylated indole **4a** (122.8 mg, 66%) as a colorless oil.[7]

Step I: In a glove box, a sealed tube was charged with $Ni(cod)_2$ (0.01 mmol, 5.0 mol%), IPr·HCl (0.01 mmol, 5.0 mol%), NaO^{t}Bu (0.02 mmol, 10 mol%), isoprene **2a** (1.2 mmol), *n*-hexane (0.50 mL) at room temperature. The reaction tube was sealed with a Teflon screw cap, removed from the glove box. Then, the reaction mixture was stirred at 100 ℃ for 12 hours. Step II: As the reaction mixture was cooled to room temperature, $(PhO)_2PO_2H$ (0.02 mmol, 10 mol%) was added to the reaction tube and stirred for 5 minutes. Then KI (0.02 mmol, 10 mol%), $K_2S_2O_8$ (0.40 mmol, 2.0 equiv.), nucleophile **4a** (0.20 mmol), I_2 (0.04 mmol, 20 mol%) and THF (0.50 mL) were added into the reaction mixture and stirred at 100 ℃ for additional 24 hours. Target product **8a** was not be detected finally.

This result indicates that hydroarylation does not proceed before the cyclodimerization of isoprene.

4.9 Capture of iodide intermediates

HRMS calculated for $C_{10}H_{14}I$ $[M+H]^+$ 261.0135, found 261.0152.

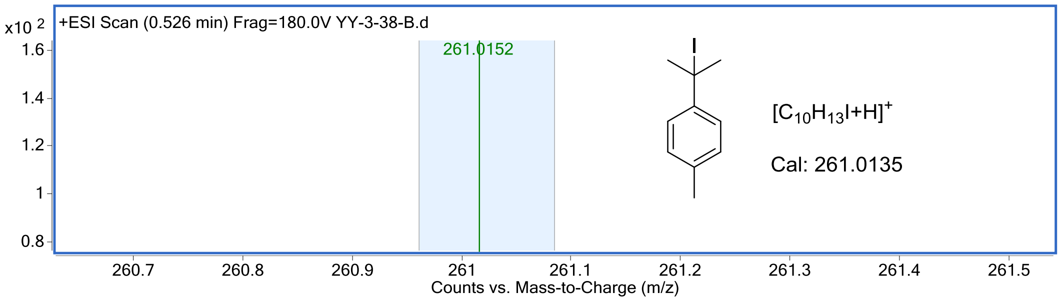

HRMS calculated for $C_{10}H_{13}INa$ $[M+Na]^+$ 282.9954, found 282.9971.

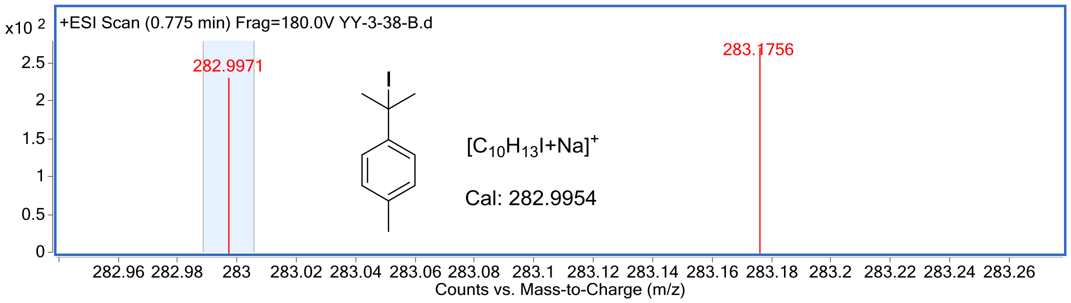

To a sealed tube (4 mL) was sequentially added limonene **9a** (0.20 mmol), I_2 (0.20 mmol), THF (0.50 mL). The resulting mixture was stirred at 60 °C or 40 °C for 30 min and then detected by HRMS. Iodide intermediates could only be detected at 60 °C.

4.10 Effect of the amount of isoprene

Supplementary Table 11. Effect of the amount of isoprene

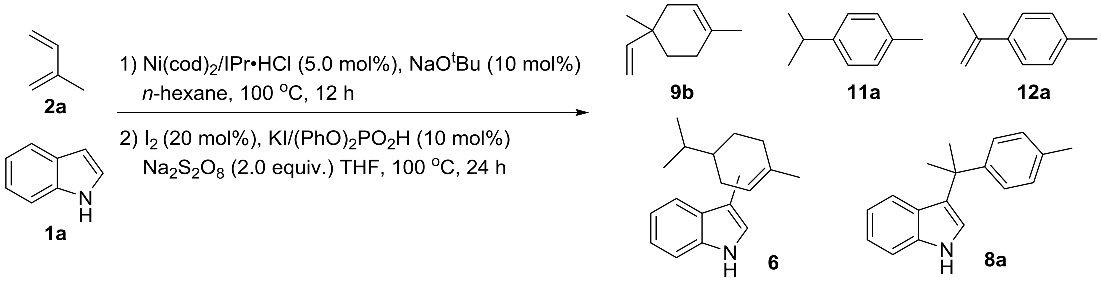

Entry	Isoprene	Yield of **9b** (%)	Yield of **11a** (%)	Yield of **12a** (%)	Yield of **6** (%)	Yield of **8a** (%)
1	2.0 equiv.	58	8	--	1	33
2	6.0 equiv.	54	7	--	5	79

[a]Conditions: Step I: **2a** (0.4 mmol or 1.2 mmol), $Ni(cod)_2$/IPr HCl (5.0 mol%), NaO^{t}Bu (10 mol%), *n*-hexane (0.50 mL), 100 °C, 12 h; Step II: **1a** (0.20 mmol), I_2 (20 mol%), $K_2S_2O_8$ (2.0 equiv.), KI (10 mol%), THF (0.50 mL), 100 °C, 24 h. Yields were determined by GC-FID. The yields of **9b**, **11a** and **12a** were calculated on the basis of isoprene. The yields of **6** and **8a** were calculated on the basis of **1a**

5. Supplementary Method 2

5.1 General procedure D: Orthogonal C-H functionalizations

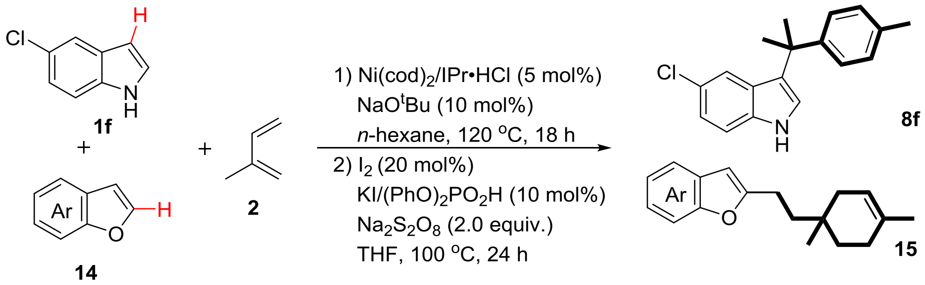

Step I: In a glove box, a sealed tube was charged with $Ni(cod)_2$ (0.01 mmol, 5.0 mol%), IPr HCl (0.01 mmol, 5.0 mol%), NaO^{t}Bu (0.02 mmol, 10 mol%), benzofuran **14** (0.20 mmol), isoprene **2** (1.2 mmol), *n*-hexane (0.50 mL) at room temperature. The reaction tube was sealed with a Teflon screw cap, removed from the glove box. Then, the reaction mixture was stirred at 120 ℃ for 18 hours. Step II: As

the reaction mixture was cooled to room temperature, $(PhO)_2PO_2H$ (0.02 mmol, 10 mol%) was added to the reaction tube and stirred for 5 minutes. Then, KI (0.02 mmol, 10 mol%), $K_2S_2O_8$ (0.40 mmol, 2.0 equiv.), indole compound **1f** (0.20 mmol), I_2 (0.04 mmol, 20 mol%) and THF (0.50 mL) were added into the reaction mixture and stirred at 100 ℃ for additional 24 hours. Direct purification by column chromatography on silica gel using petroleum ether and ethyl acetate afforded the corresponding products **8f** and **15**.

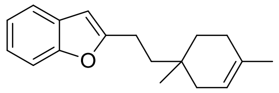

2-(2-(1,4-Dimethylcyclohex-3-en-1-yl)ethyl)benzofuran (15a): Prepared according to the general procedure D, colorless oil, 47.7 mg, 94% yield, R_f = 0.5 (PE). **^{1}H NMR** (400 MHz, Chloroform-*d*) δ 7.53-7.47 (m, 1H), 7.43 (d, *J* = 7.5 Hz, 1H), 7.25-7.17 (m, 2H), 6.40 (s, 1H), 5.34-5.28 (m, 1H), 2.86-2.69 (m, 2H), 2.00-1.66 (m, 9H), 1.56-1.43 (m, 2H), 0.98 (s, 3H); **^{13}C NMR** (100 MHz, Chloroform-*d*) δ 160.52, 154.72, 132.95, 129.16, 123.12, 122.48, 120.24, 119.73, 110.77, 101.50, 39.08, 37.89, 33.87, 31.05, 27.66, 24.34, 23.54, 23.24. **HRMS** calculated for $C_{18}H_{23}O$ $[M+H]^+$ 255.1743, found 255.1744. **8f**, 47.3 mg, 83% yield.

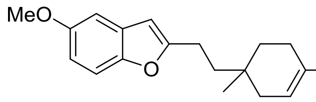

2-(2-(1,4-Dimethylcyclohex-3-en-1-yl)ethyl)-5-methoxybenzofuran (15b): Prepared according to the general procedure D, colorless oil, 52.2 mg, 92% yield, R_f = 0.4 (PE/EA = 50/1). **^{1}H NMR** (400 MHz, Chloroform-*d*) δ 7.31 (d, *J* = 8.9 Hz, 1H), 6.97 (d, *J* = 2.5 Hz, 1H), 6.82 (dd, *J* = 8.8, 2.6 Hz, 1H), 6.33 (s, 1H), 5.34-5.28 (m, 1H), 3.84 (s, 3H), 2.86-2.64 (m, 2H), 1.99-1.64 (m, 9H), 1.54-1.42 (m, 2H), 0.97 (s, 3H); **^{13}C NMR** (100 MHz, Chloroform-*d*) δ 161.41, 155.82, 149.69, 132.92, 129.72, 119.71, 111.36, 111.07, 103.23, 101.70, 56.00, 39.06, 37.87, 33.85, 31.02, 27.65, 24.31, 23.52, 23.33. **HRMS** calculated for $C_{19}H_{25}O_2$ $[M+H]^+$ 285.1849, found 285.1856. **8f**, 48.2 mg, 85% yield.

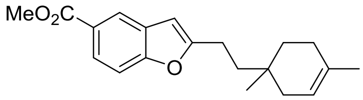

Methyl 2-(2-(1,4-dimethylcyclohex-3-en-1-yl)ethyl)benzofuran-5-carboxylate (15c): Prepared according to the general procedure D, white solid, melting point: 58-60 °C, 41.5 mg, 65% yield, R_f = 0.5 (PE/EA = 20/1). **^{1}H NMR** (400 MHz, Chloroform-*d*) δ 8.20 (d, *J* = 1.4 Hz, 1H), 7.93 (dd, *J* = 8.6, 1.7 Hz, 1H), 7.41 (d, *J* = 8.6 Hz, 1H), 6.49-6.36 (m, 1H), 5.35-5.26 (m, 1H), 3.92 (s, 3H), 2.88-2.64 (m, 2H), 1.96-1.61 (m, 9H), 1.52-1.40 (m, 2H), 0.95 (s, 3H); **^{13}C NMR** (100 MHz, Chloroform-*d*) δ 167.59, 162.09, 157.36, 132.96, 129.15, 125.11, 124.81, 122.66, 119.63, 110.57, 101.95, 52.14, 38.84, 37.84, 33.82, 31.03, 27.62, 24.33, 23.51, 23.24. **HRMS** calculated for $C_{20}H_{25}O_3$ $[M+H]^+$ 313.1798, found 313.1802. **8f**, 45.9 mg, 81% yield.

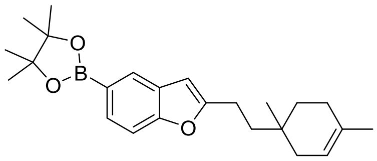

2-(2-(2-(1,4-Dimethylcyclohex-3-en-1-yl)ethyl)benzofuran-5-yl)-4,4,5,5-tetramethyl-1,3,2-dioxaborolane (15d): Prepared according to the general procedure D, colorless oil, 54.6 mg, 72% yield, R_f = 0.6 (PE/EA = 20/1). **^{1}H NMR** (400 MHz, Chloroform-*d*) δ 7.97 (s, 1H), 7.67 (dd, *J* = 8.2, 1.1 Hz, 1H), 7.40 (d, *J* = 8.2 Hz, 1H), 6.41-6.32 (m, 1H), 5.34-5.26 (m, 1H), 2.80-2.68 (m, 2H), 1.99-1.65 (m, 9H), 1.54-1.42 (m, 2H), 1.36 (s, 12H), 0.95 (s, 3H); **^{13}C NMR** (100 MHz, Chloroform-*d*) δ 160.56, 156.88, 132.96, 129.86, 128.88, 127.57, 119.71, 110.28, 101.52, 83.77, 38.96, 37.89, 33.85, 31.05, 27.65, 25.03, 24.33, 23.54, 23.22. **HRMS** calculated for $C_{24}H_{34}BO_3$ $[M+H]^+$ 381.2596, found 381.2602. **8f**, 44.4 mg, 78% yield.

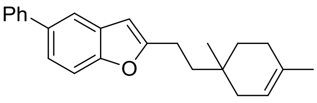

2-(2-(1,4-Dimethylcyclohex-3-en-1-yl)ethyl)-5-phenylbenzofuran (15e): Prepared according to the general procedure D, colorless oil, 63.6 mg, 96% yield, R_f = 0.2 (PE). **^{1}H NMR** (400 MHz, Chloroform-*d*) δ 7.74-7.69 (m, 1H), 7.68-7.62 (m, 2H), 7.52-7.44 (m, 4H), 7.37 (t, *J* = 7.4 Hz, 1H), 6.49-6.38 (m, 1H), 5.42-5.31 (s, 1H), 2.94-2.68 (m, 2H), 2.04-1.69 (m, 9H), 1.59-1.45 (m, 2H), 1.01 (s, 3H); **^{13}C NMR** (100 MHz, Chloroform-*d*) δ 161.27, 154.37, 142.03, 136.19, 132.95, 129.69, 128.80, 127.53, 126.82, 122.82, 119.72, 118.84, 110.85, 101.73, 39.07, 37.88, 33.87, 31.05, 27.66, 24.33, 23.55, 23.32. **HRMS** calculated for $C_{24}H_{27}O$ $[M+H]^+$ 331.2056, found 331.2062. **8f**, 49.3 mg, 87% yield.

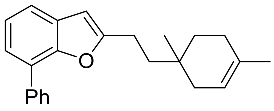

2-(2-(1,4-Dimethylcyclohex-3-en-1-yl)ethyl)-7-phenylbenzofuran (15f): Prepared according to the general procedure D, colorless oil, 59.3 mg, 90% yield, R_f = 0.2 (PE). **^{1}H NMR** (400 MHz, Chloroform-*d*) δ 7.97-7.91 (m, 2H), 7.56 (t, *J* = 7.6 Hz, 2H), 7.51 (dd, *J* = 7.6, 1.1 Hz, 1H), 7.48-7.42 (m, 2H), 7.32 (t, *J* = 7.6 Hz, 1H), 6.49 (s, 1H), 5.48-5.32 (m, 1H), 2.91-2.76 (m, 2H), 2.04-1.70 (m, 9H), 1.59-1.45 (m, 2H), 1.02 (s, 3H); **^{13}C NMR** (100 MHz, Chloroform-*d*) δ 160.77, 151.86, 136.94, 132.92, 129.94, 128.73, 128.65, 127.56, 124.86, 123.03, 122.81, 119.73, 119.49, 101.62, 38.77, 37.95, 33.84, 31.05, 27.65, 24.41, 23.53, 23.27. **HRMS** calculated for $C_{24}H_{27}O$ $[M+H]^+$ 331.2056, found 331.2051. **8f**, 48.7 mg, 86% yield.

5.2 Transformations

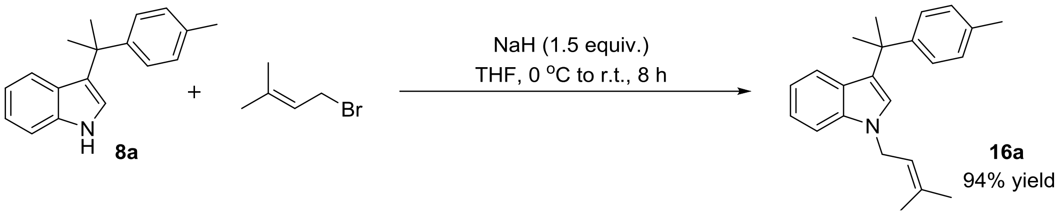

In a Schlenk tube (4.0 mL), indole **8a** (0.20 mmol) was dissolved in THF (0.50 mL). After cooling to 0 °C, NaH (1.5 equiv.) was added, and the mixture was stirred at this temperature for 15 min. Then prenyl bromide (1.5 equiv.) was added dropwise at 0 °C, and the mixture was stirred at room temperature for 8 h. The reaction was quenched by water and extracted with DCM. The organic phase was dried over sodium sulfate and concentrated under reduced pressure. Purification by flash column chromatography on silica gel (PE/EtOAc = 50:1) yielded the desired product **16a**.

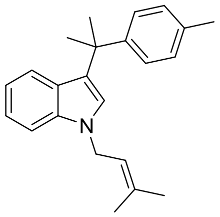

1-(3-Methylbut-2-en-1-yl)-3-(2-(*p*-tolyl)propan-2-yl)-1H-indole (16a): Yellow oil, 59.8 mg, 94% yield, R_f = 0.3 (PE/EA = 50/1). **^{1}H NMR** (400 MHz, Chloroform-*d*) δ 7.31 (d, *J* = 8.3 Hz, 1H), 7.28 (d, *J* = 8.2 Hz, 2H), 7.20-7.06 (m, 4H), 7.04 (s, 1H), 6.94-6.87 (m, 1H), 5.50-5.40 (m, 1H), 4.73 (d, *J* = 6.7 Hz, 2H), 2.34 (s, 3H), 1.89 (s, 3H), 1.83 (s, 3H), 1.79 (s, 6H); **^{13}C NMR** (100 MHz, Chloroform-*d*) δ 147.28, 137.16, 135.75, 134.87, 128.78, 126.78, 126.41, 124.71, 124.18, 121.60, 121.04, 120.57, 118.33, 109.49, 44.26, 38.72, 30.99, 25.83, 21.08, 18.22. **HRMS** calculated for $C_{23}H_{28}N$ $[M+H]^+$ 318.2216, found 318.2217.

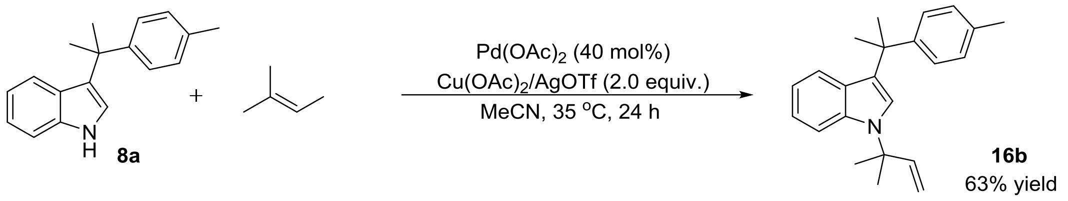

Indole **8a** (0.20 mmol), $Cu(OAc)_2$ (0.40 mmol), AgOTf (0.40 mmol) and CH_3CN (1.0 mL) were sequentially added to a sealed tube (4.0 mL). Then $Pd(OAc)_2$ (10 mol%) and 2-methyl-2-butene (30 equiv.) were introduced. The resulting dark mixture was stirred at 35 °C, followed by three sequential additions of $Pd(OAc)_2$ (10 mol% after each hour). The mixture was allowed to stir for 24 h in total. The solvent was then evaporated and the residue was loaded onto silica gel for purification (PE/EtOAc = 50:1) to give the expected product **16b**.[8]

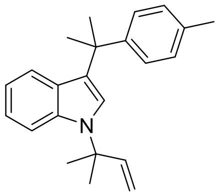

1-(2-Methylbut-3-en-2-yl)-3-(2-(*p*-tolyl)propan-2-yl)-1H-indole (16b): Colorless oil, 39.7 mg, 63% yield, R_f = 0.3 (PE/EA = 50/1). **^{1}H NMR** (400 MHz, Chloroform-*d*) δ 7.51 (d, *J* = 8.4 Hz, 1H), 7.26 (s, 1H), 7.23 (d, *J* = 7.9 Hz, 2H), 7.13-6.98 (m, 4H), 6.85 (t, *J* = 7.5 Hz, 1H), 6.22 (dd, *J* = 17.5, 10.7 Hz, 1H), 5.31-5.15 (m, 2H), 2.32 (s, 3H), 1.81 (s, 6H), 1.77 (s, 6H); **^{13}C NMR** (100 MHz, Chloroform-*d*) δ 147.34, 144.69, 136.55, 134.85, 128.80, 128.03, 126.39, 123.76, 121.66, 121.57, 120.27, 118.10, 113.69, 113.37, 58.93, 38.76, 31.01, 28.10, 21.08. **HRMS** calculated for $C_{23}H_{28}N$ $[M+H]^+$ 318.2216, found 318.2214.

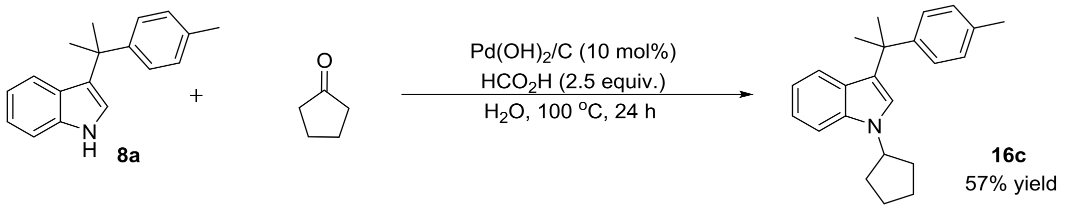

$Pd(OH)_2/C$ (20 wt%, 10 mol%) and indole **8a** (0.20 mmol) were added into a sealed tube (10 mL) charged with a magnetic stir bar. After three cycles of evacuation/backfilling sequence with argon, ketone (0.60 mmol), distilled water (0.50 mL) and formic acid (0.50 mmol, 2.5 equiv.) were added. Then the reaction was stirred in a preheated oil bath at 100 °C for 24 h. After being cooled to room temperature, the mixture was extracted with dichloromethane and the combined organic extracts were washed by brine, dried over, filtered, concentrated, and purified by flash chromatography on silica gel (PE/EtOAc = 50:1) to give product **16c**.[9]

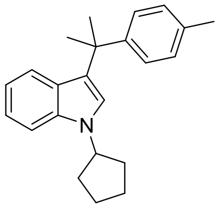

1-Cyclopentyl-3-(2-(*p*-tolyl)propan-2-yl)-1H-indole (16c): Colorless oil, 36.0 mg, 57% yield, R_f = 0.3 (PE/EA = 50/1). **^{1}H NMR** (700 MHz, Chloroform-*d*) δ 7.35 (d, *J* = 8.3 Hz, 1H), 7.23 (d, *J* = 8.1 Hz, 2H), 7.13-7.09 (m, 2H), 7.07-7.03 (m, 3H), 6.86 (t, *J* = 7.4 Hz, 1H), 4.79 (p, *J* = 7.4 Hz, 1H), 2.30 (s, 3H), 2.27-2.20 (m, 2H), 2.00-1.90 (m, 4H), 1.83-1.76 (m, 2H), 1.75 (s, 6H); **^{13}C NMR** (175 MHz, Chloroform-*d*) δ 147.32, 137.41, 134.87, 128.79, 126.75, 126.41, 124.68, 121.61, 121.06, 120.90, 118.31, 109.76, 56.91, 38.79, 32.68, 31.01, 24.28, 21.08. **HRMS** calculated for $C_{23}H_{28}N$ $[M+H]^+$ 318.2216, found 318.2223.

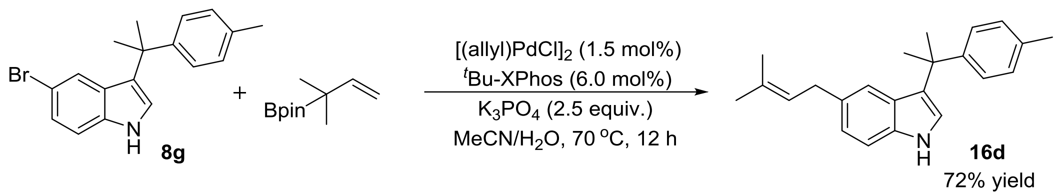

A screw-cap sealed tube, which was equipped with a magnetic stir bar and fitted with a teflon septum, was charged with $[(allyl)PdCl]_2$ (1.5 mol%), ^{t}Bu-XPhos (6.0 mol%), indole **8g** (0.20 mmol) and K_3PO_4 (0.50 mmol, 2.5 equiv.). The sealed tube was evacuated and backfilled with argon and allylboronic acid pinacol ester (0.30 mmol) was added via syringe. The mixture of acetonitrile (0.20 mL) and water (0.20 mL) was added via syringe and the reaction mixture was allowed to stir at 70 °C for 12 h. After cooling to room temperature, the reaction mixture was diluted with ethyl acetate and washed with water. The aqueous layer was extracted with ethyl acetate (2×10 mL). The combined organic layers were dried over Na_2SO_4, concentrated in vacuo, and purified by flash chromatography on silica gel (PE/EtOAc = 20:1) to give product **16d**.[10]

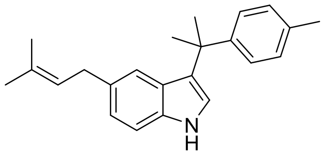

5-(3-Methylbut-2-en-1-yl)-3-(2-(*p*-tolyl)propan-2-yl)-1H-indole (16d): Colorless oil, 45.5 mg, 72% yield, R_f = 0.4 (PE/EA = 20/1). **^{1}H NMR** (700 MHz, Chloroform-*d*) δ 7.80 (s, 1H), 7.26-7.23 (m, 3H), 7.09-7.04 (m, 3H), 6.95 (d, *J* = 8.2 Hz, 1H), 6.86 (s, 1H), 5.33-5.18 (m, 1H), 3.27 (d, *J* = 7.3 Hz, 2H), 2.33 (s, 3H), 1.77 (s, 6H), 1.70 (s, 3H), 1.63 (s, 3H); **^{13}C NMR** (175 MHz, Chloroform-*d*) δ 147.06, 135.77, 134.85, 132.06, 131.73, 128.78, 126.42, 126.40, 126.11, 124.40, 122.59, 120.76, 120.50, 110.92, 38.67, 34.45, 30.79, 25.83, 21.06, 17.77. **HRMS** calculated for $C_{23}H_{28}N$ $[M+H]^+$ 318.2216, found 318.2209.

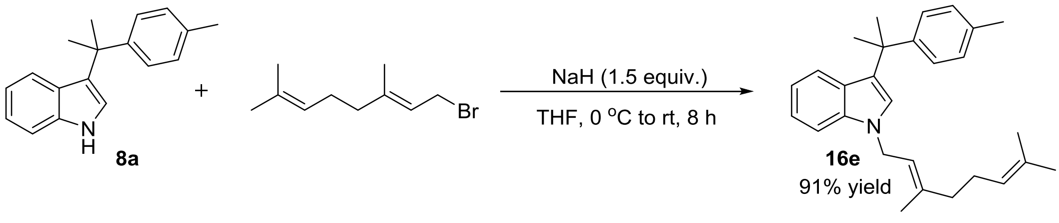

In a Schlenk tube (4.0 mL), indole **8a** (0.20 mmol) was dissolved in THF (0.50 mL). After cooling to 0 °C, NaH (1.5 equiv) was added, and the mixture was stirred at this temperature for 15 min. Then geranyl bromide (1.5 equiv.) was added dropwise at 0 °C, and the mixture was stirred at room temperature for 8 h. The reaction was quenched by water and extracted with DCM. The organic phase was dried over sodium sulfate and concentrated under reduced pressure. Purification by flash column chromatography on silica gel (PE/EtOAc = 50:1) yielded the desired product **16e**.

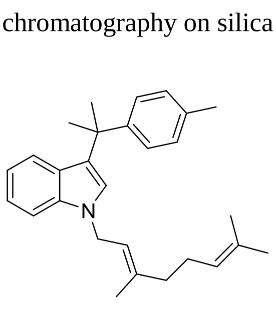

(*E*)-1-(3,7-Dimethylocta-2,6-dien-1-yl)-3-(2-(*p*-tolyl)propan-2-yl)-1H-indole (16e): Yellow oil, 70.0 mg, 91% yield, R_f = 0.3 (PE/EA = 50/1). **^{1}H NMR** (400 MHz, Chloroform-*d*) δ 7.36 (d, *J* = 8.2 Hz, 1H), 7.33 (d, *J* = 8.2 Hz, 2H), 7.22-7.11 (m, 4H), 7.08 (s, 1H), 6.95 (ddd, *J* = 8.0, 7.0, 0.9 Hz, 1H), 5.55-5.47 (m, 1H), 5.23-5.15 (m, 1H), 4.78 (d, *J* = 6.6 Hz, 2H), 2.39 (s, 3H), 2.28-2.11 (m, 4H), 1.92 (s, 3H), 1.84 (s, 6H), 1.79-1.73 (m, 3H), 1.70 (s, 3H); **^{13}C NMR** (100 MHz, Chloroform-*d*) δ 147.28, 139.23, 137.23, 134.85, 131.93, 128.78, 126.80,

126.41, 124.70, 124.18, 123.89, 121.58, 121.05, 120.46, 118.34, 109.55, 44.23, 39.61, 38.72, 30.99, 26.47, 25.85, 21.06, 17.87, 16.57. **HRMS** calculated for $C_{28}H_{36}N$ $[M+H]^+$ 386.2842, found 386.2846.

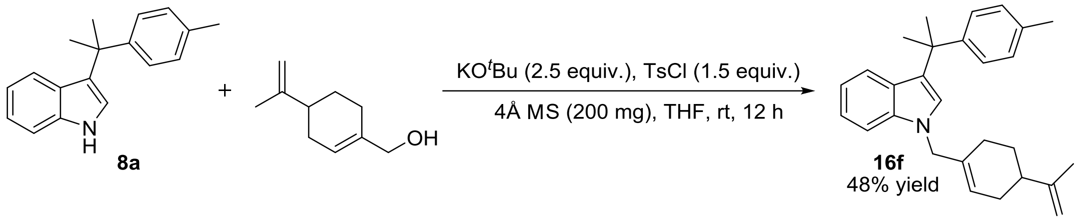

To a Schlenk tube was added indole **8a** (0.20 mmol), perillyl alcohol (0.30 mmol), TsCl (0.30 mmol, 1.5 equiv.), KO^{t}Bu (0.50 mmol, 2.5 equiv.), 4Å MS (200 mg) and THF (1.0 mL) under argon. Then the reaction mixture was stirred at room temperature for 12 h. The reaction was quenched by water and extracted with DCM. The organic phase was dried over sodium sulfate and concentrated under reduced pressure. Purification by flash column chromatography on silica gel (PE/EtOAc = 50:1) yielded the desired product **16f**.[11]

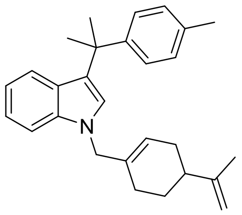

1-((4-(Prop-1-en-2-yl)cyclohex-1-en-1-yl)methyl)-3-(2-(*p*-tolyl)propan-2-yl)-1H-indole (16f): An inseparable mixture of the self-coupling product of perilla alcohol and **16f**, 50.3 mg in total, 48 % yield of **16f**, R_f = 0.2 (PE/EA = 20/1). NMR data for **16f** was provided. **^{1}H NMR** (400 MHz, Chloroform-*d*) δ 7.29 (d, *J* = 8.2 Hz, 1H), 7.24 (d, *J* = 8.3 Hz, 2H), 7.13-7.03 (m, 4H), 6.99 (s, 1H), 6.92-6.84 (m, 1H), 5.64-5.55 (m, 1H), 4.75-4.70 (m, 2H), 4.60 (s, 2H), 2.32 (s, 3H), 2.22-2.09 (m, 3H), 2.04-1.90 (m, 3H), 1.86-1.68 (m, 9H), 1.57-1.44 (m, 1H); **^{13}C NMR** (100 MHz, Chloroform-*d*) δ 149.72, 147.24, 137.60, 134.90, 134.00, 128.81, 126.67, 126.38, 124.99, 124.82, 123.65, 121.55, 121.13, 118.34, 109.74, 108.91, 52.45, 41.02, 38.68, 31.02, 30.98, 30.54, 27.45, 26.69, 21.08, 20.95. **HRMS** calculated for $C_{28}H_{34}N$ $[M+H]^+$ 384.2686, found 384.2691.

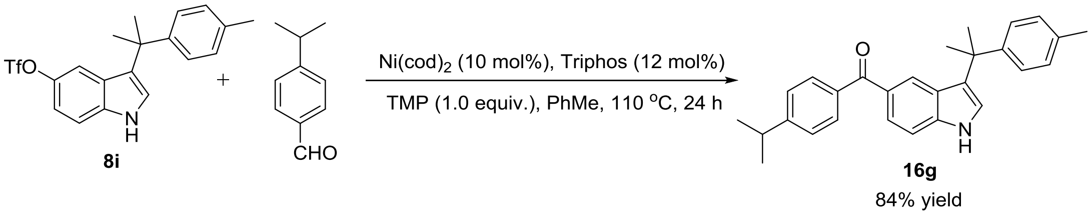

In a glove box, a sealed tube was charged with $Ni(cod)_2$ (0.02 mmol, 10 mol%), Triphos (0.012 mmol, 12 mol%), TMP (0.20 mmol, 1.0 equiv.), indole **8i** (0.20 mmol), cumaldehyde (0.40 mmol) and toluene (1.0 mL) at room temperature. The reaction tube was sealed with a Teflon screw cap, removed from the glove box. Then, the reaction mixture was stirred at 110 ℃ for 24 hours. The reaction was quenched by water and extracted with DCM. The organic phase was dried over sodium sulfate and concentrated under reduced pressure. Purification by flash column chromatography on silica gel (PE/EtOAc = 5:1) yielded the desired product **16g**.[12]

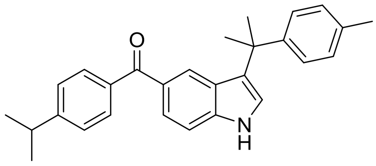

(4-Isopropylphenyl)(3-(2-(*p*-tolyl)propan-2-yl)-1H-indol-5-yl) methanone (16g): Colorless oil, 66.2 mg, 84% yield, R_f = 0.3 (PE/EA = 5/1). **^{1}H NMR** (400 MHz, Chloroform-*d*) δ 8.68 (s, 1H),

7.76 (dd, J = 8.6, 1.5 Hz, 1H), 7.55 (s, 1H), 7.50 (d, J = 8.2 Hz, 2H), 7.36 (d, J = 8.6 Hz, 1H), 7.24-7.15 (m, 5H), 7.05 (d, J = 8.0 Hz, 2H), 3.08-2.89 (m, 1H), 2.39 (s, 3H), 1.72 (s, 6H), 1.35 (s, 3H), 1.33 (s, 3H); **^{13}C NMR** (100 MHz, Chloroform-*d*) δ 196.96, 152.89, 146.66, 139.65, 136.09, 134.83, 130.50, 128.93, 128.53, 127.91, 126.32, 126.28, 126.14, 125.05, 123.69, 121.99, 111.44, 38.57, 34.35, 30.73, 23.95, 21.21. **HRMS** calculated for $C_{28}H_{30}NO$ [M+H]$^+$ 396.2322, found 396.2328.

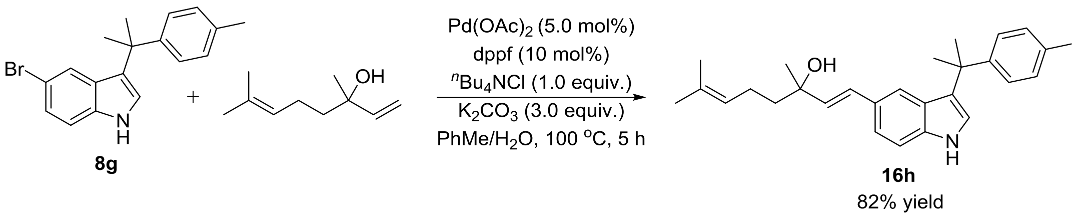

To a Schlenk tube was added $Pd(OAc)_2$ (0.01 mmol, 5.0 mol%), dppf (0.02 mmol, 10 mol%), $^{n}Bu_4NCl$ (0.20 mmol, 1.0 equiv.), indole **8g** (0.20 mmol), K_2CO_3 (0.60 mmol, 3.0 equiv.), linalool (0.30 mmol) and toluene/H_2O (0.50/0.50 mL) under argon. Then, the reaction mixture was stirred at 100 ℃ for 5 hours. The reaction was quenched by water and extracted with DCM. The organic phase was dried over sodium sulfate and concentrated under reduced pressure. Purification by flash column chromatography on silica gel (PE/EtOAc = 5:1) yielded the desired product **16h**.[13]

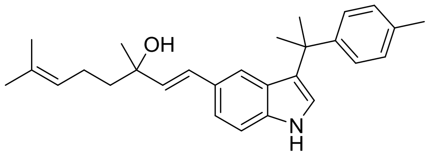

(*E*)-3,7-Dimethyl-1-(3-(2-(*p*-tolyl)propan-2-yl)-1H-indol-5-yl)octa-1,6-dien-3-ol (16h): Colorless oil, 65.7 mg, 82% yield, R_f = 0.1 (PE/EA = 20/1). **^{1}H NMR** (400 MHz, Chloroform-*d*) δ 7.95 (s, 1H), 7.26-7.23 (m, 4H), 7.09-7.03 (m, 4H), 6.52 (d, J = 16.1 Hz, 1H), 6.05 (d, J = 16.1 Hz, 1H), 5.15 (t, J = 7.1 Hz, 1H), 2.32 (s, 3H), 2.14-1.99 (m, 2H), 1.77 (s, 6H), 1.70-1.65 (m, 5H), 1.58 (s, 3H), 1.36 (s, 3H); **^{13}C NMR** (100 MHz, Chloroform-*d*) δ 146.90, 136.85, 135.00, 133.42, 132.07, 128.86, 128.52, 128.14, 126.56, 126.35, 124.58, 121.15, 120.51, 119.65, 111.33, 73.68, 42.79, 38.68, 30.84, 28.49, 25.85, 23.15, 21.04, 17.86. **HRMS** calculated for $C_{28}H_{36}NO$ [M+H]$^+$ 402.2791, found 402.2787.

5.3 Scale-up experiment

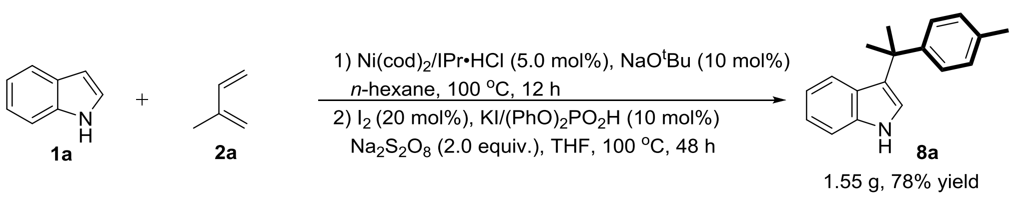

Step I: In a glove box, a sealed tube was charged with $Ni(cod)_2$ (0.40 mmol, 5.0 mol%), IPr HCl (0.40 mmol, 5.0 mol%), NaO^{t}Bu (0.80 mmol, 10 mol%), isoprene **2a** (48 mmol), *n*-hexane (25 mL) at room temperature. The reaction tube was sealed with a Teflon screw cap, removed from the glove box. Then, the reaction mixture was stirred at 100 ℃ for 12 hours. Step II: As the reaction mixture was cooled to room temperature, $(PhO)_2PO_2H$ (0.80 mmol, 10 mol%) was added to the reaction tube and stirred for 5 minutes. Then, KI (0.80 mmol, 10 mol%), $K_2S_2O_8$ (16 mmol, 2.0 equiv.), indole **1a** (8.0 mmol), I_2 (1.6 mmol, 20 mol%) and THF (25 mL) were added into the reaction mixture and stirred at 100 ℃ for additional 24 hours. Direct purification by column chromatography on silica gel using petroleum ether and ethyl acetate afforded the corresponding product **8a**.

6. Supplementary Note 4

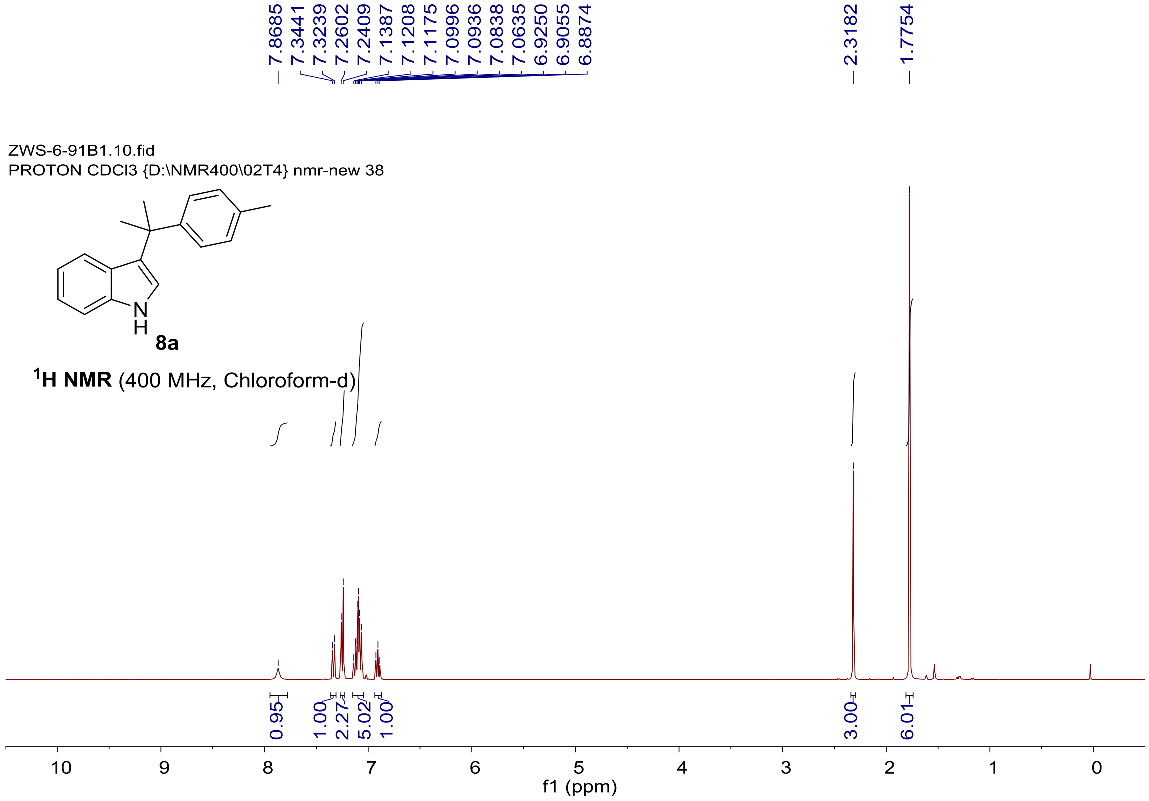

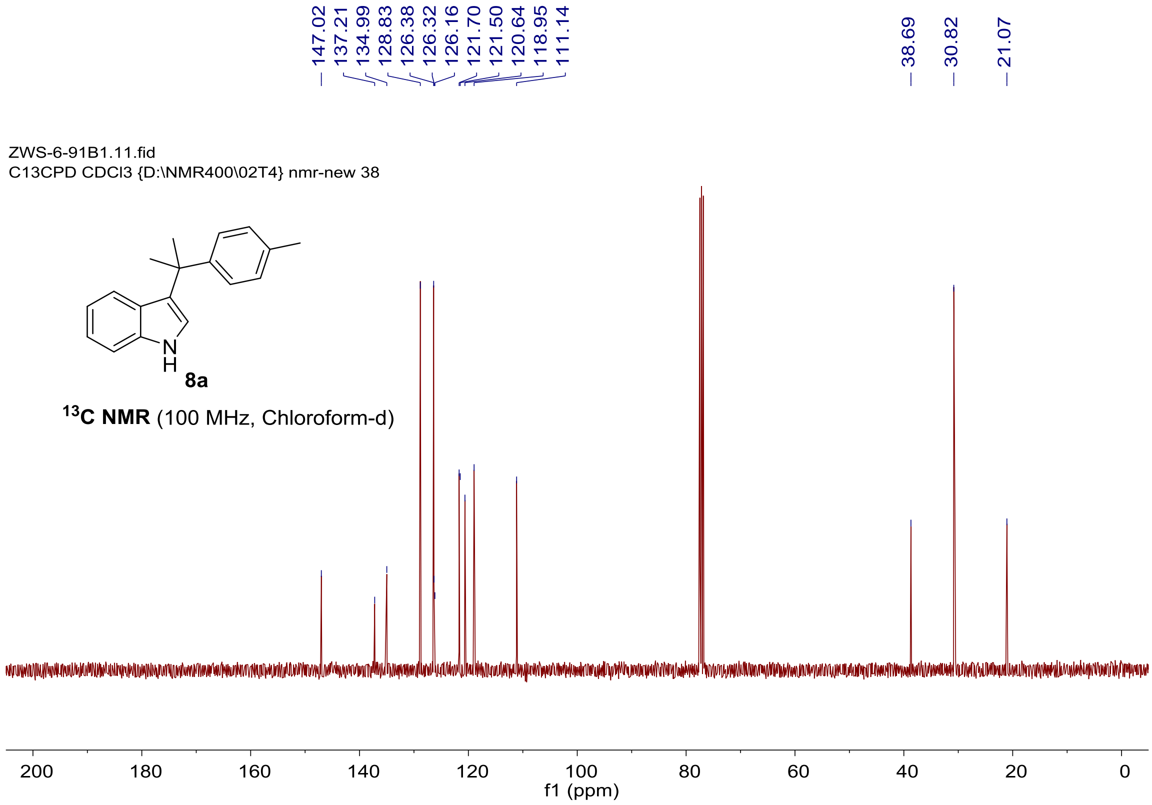

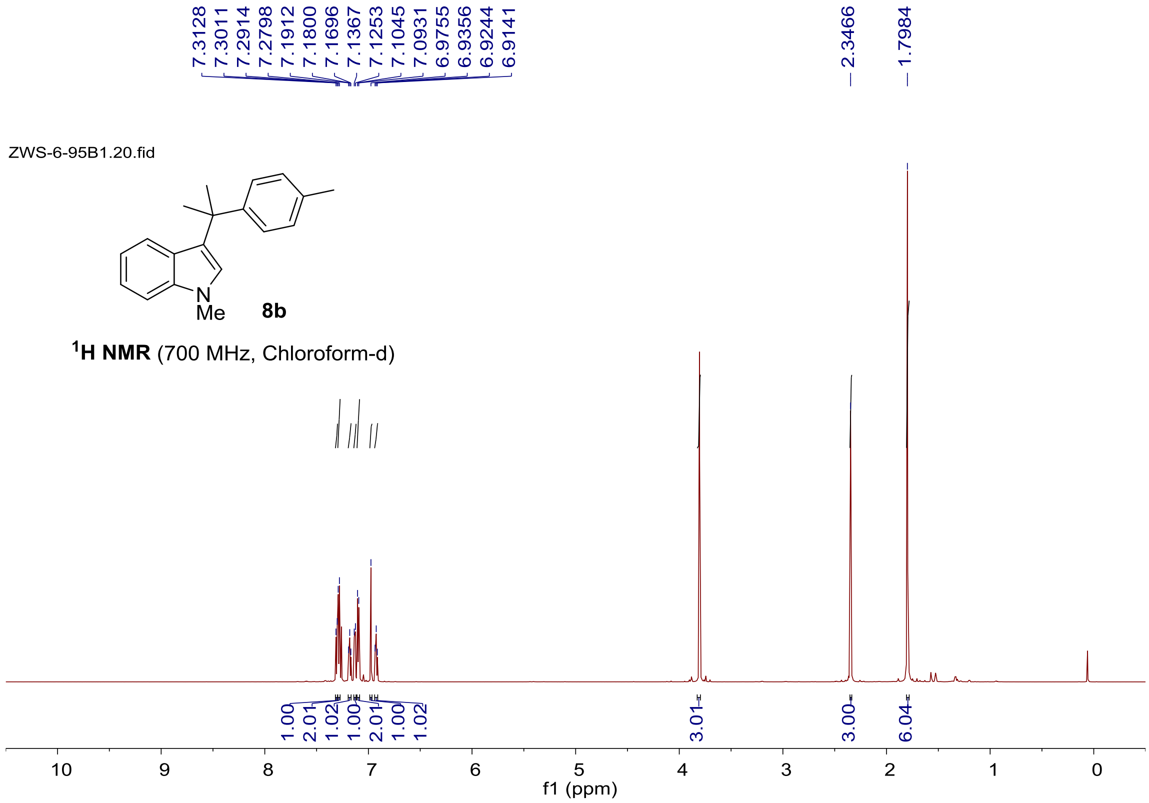
7.3128
7.3011
7.2914
7.2798
7.1912
7.1800
7.1696
7.1367
7.1253
7.1045
7.0931
6.9755
6.9356
6.9244
6.9141
2.3466
1.7984
ZWS-6-95B1.20.fid
N
Me
8b
1H NMR (700 MHz, Chloroform-d)
1.00
2.01
1.02
1.00
2.01
1.00
1.02
3.01
3.00
6.04
10
9
8
7
6
5
4
3
2
1
0
f1 (ppm)

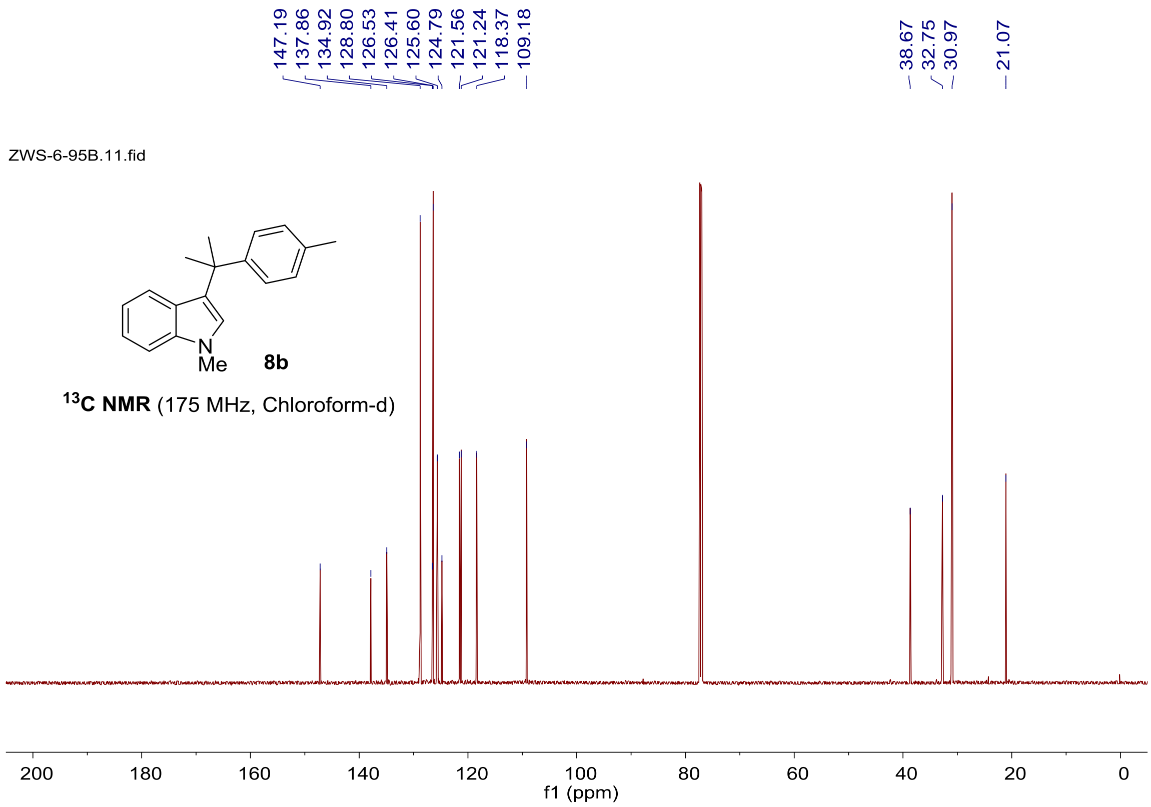
147.19
137.86
134.92
128.80
126.53
126.41
125.60
124.79
121.56
121.24
118.37
109.18
38.67
32.75
30.97
21.07
ZWS-6-95B.11.fid
N
Me
8b
13C NMR (175 MHz, Chloroform-d)
200
180
160
140
120
100
80
60
40
20
0
f1 (ppm)

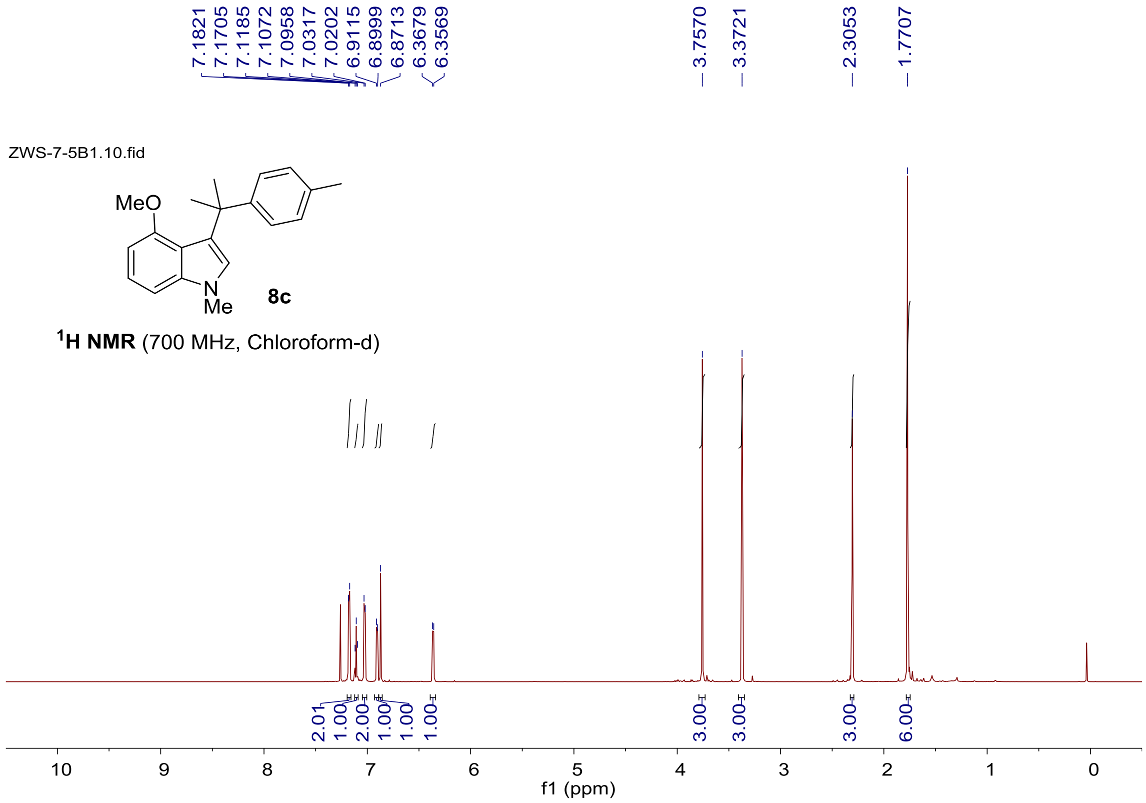
7.1821
7.1705
7.1185
7.1072
7.0958
7.0317
7.0202
6.9115
6.8999
6.8713
6.3679
6.3569
3.7570
3.3721
2.3053
1.7707
ZWS-7-5B1.10.fid
MeO
N
Me
8c
¹H NMR (700 MHz, Chloroform-d)
2.01
1.00
2.00
1.00
1.00
1.00
3.00
3.00
3.00
6.00
f1 (ppm)

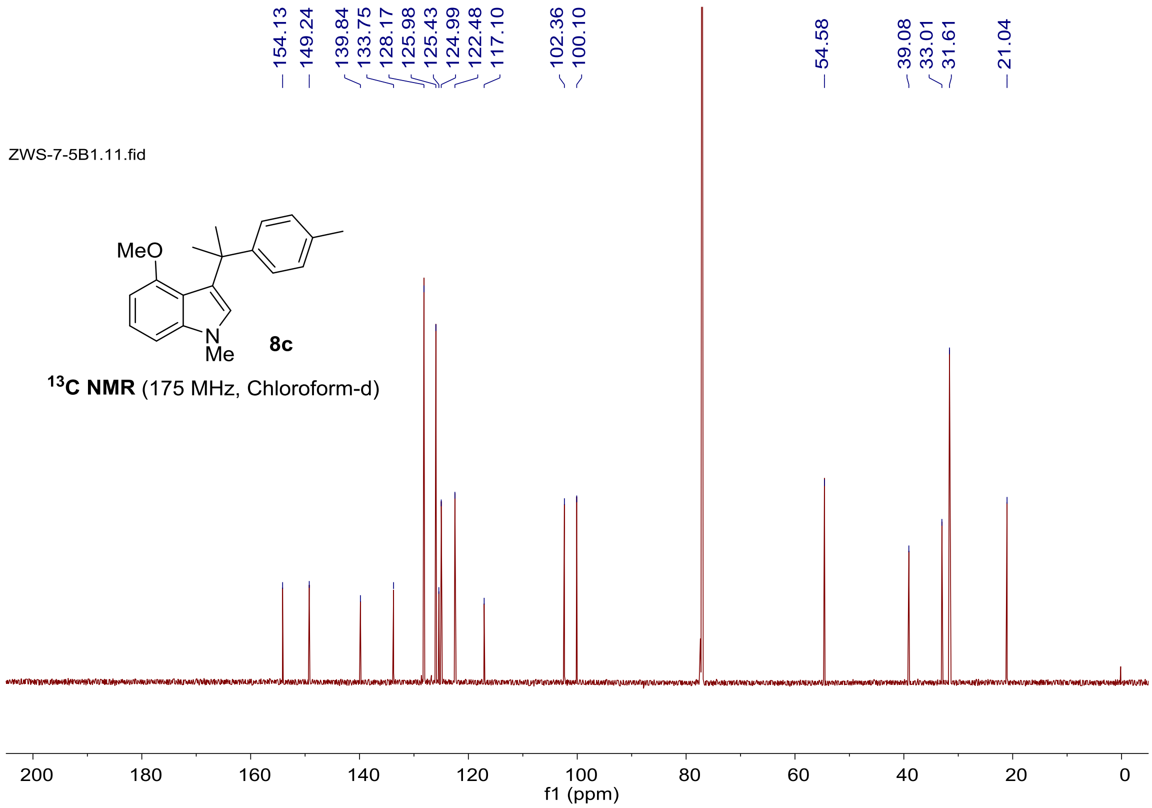
154.13
149.24
139.84
133.75
128.17
125.98
125.43
124.99
122.48
117.10
102.36
100.10
54.58
39.08
33.01
31.61
21.04
ZWS-7-5B1.11.fid
MeO
N
Me
8c
¹³C NMR (175 MHz, Chloroform-d)
f1 (ppm)

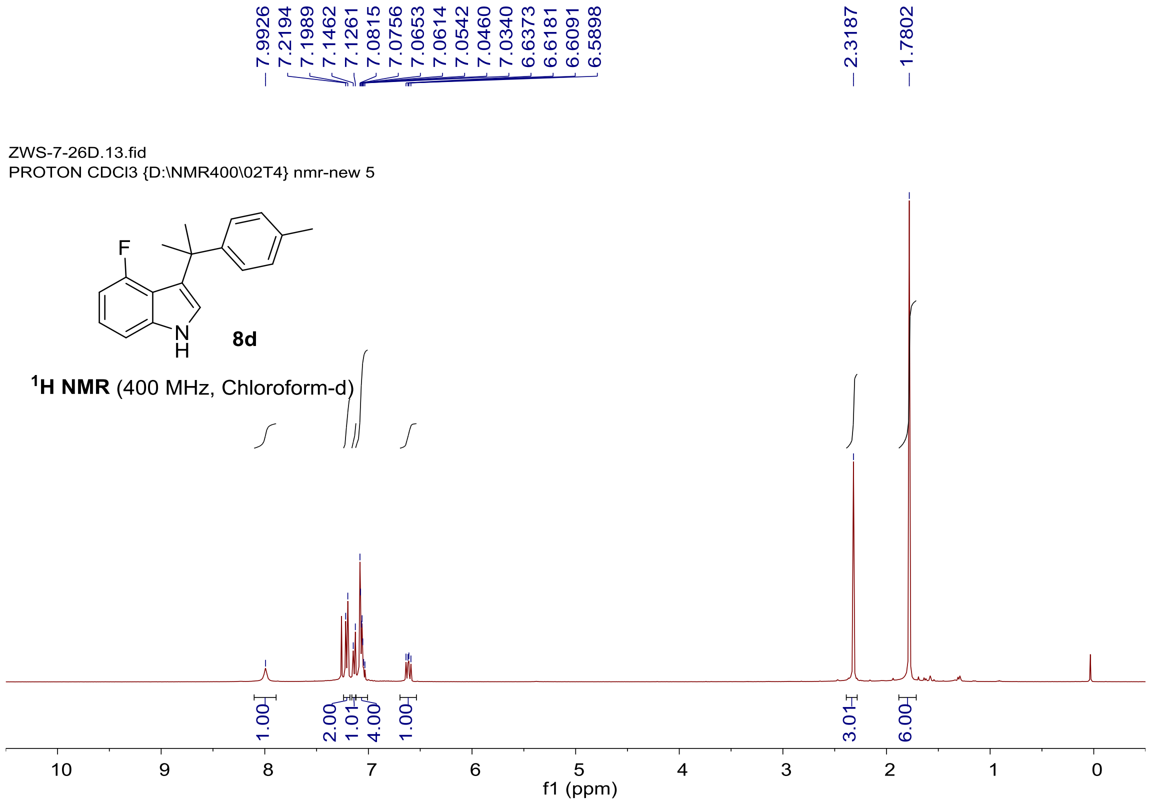
7.9926
7.2194
7.1989
7.1462
7.1261
7.0815
7.0756
7.0653
7.0614
7.0542
7.0460
7.0340
6.6373
6.6181
6.6091
6.5898
2.3187
1.7802
ZWS-7-26D.13.fid
PROTON CDCl3 {D:\NMR400\02T4} nmr-new 5
F
N
H
8d
1H NMR (400 MHz, Chloroform-d)
1.00
2.00
1.01
4.00
1.00
3.01
6.00
f1 (ppm)

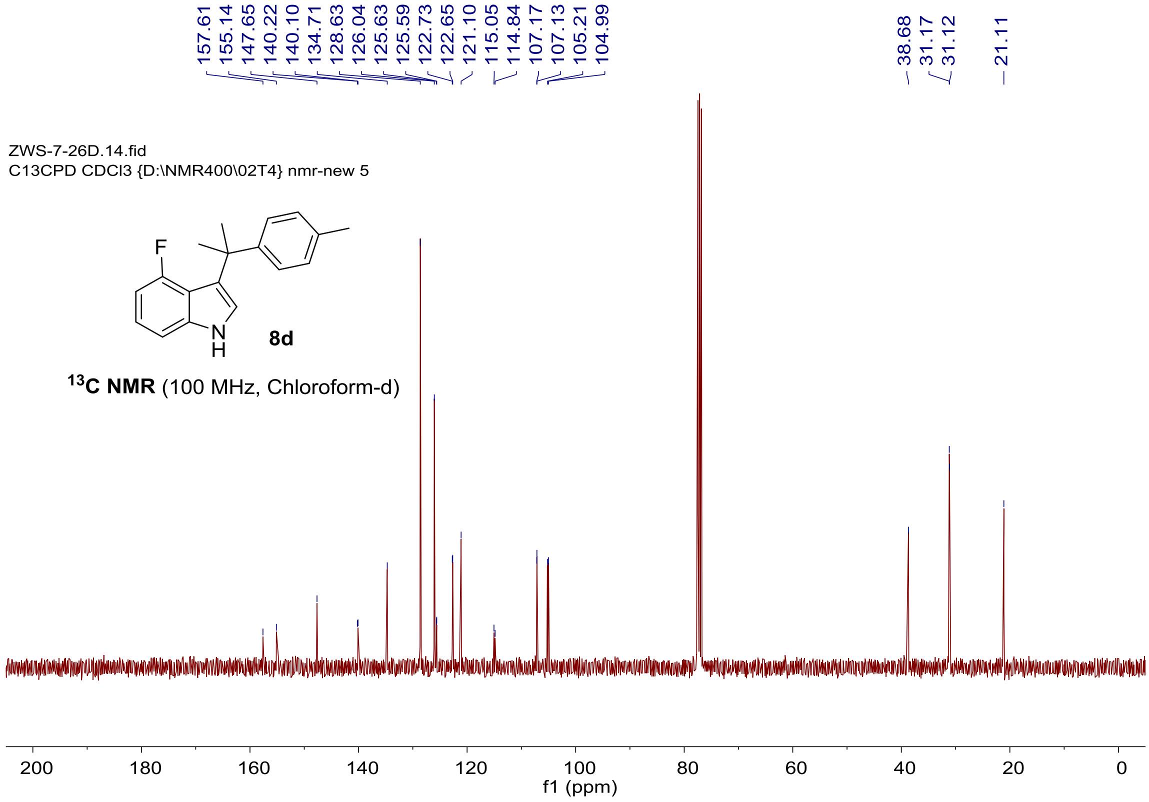
157.61
155.14
147.65
140.22
140.10
134.71
128.63
126.04
125.63
125.59
122.73
122.65
121.10
115.05
114.84
107.17
107.13
105.21
104.99
38.68
31.17
31.12
21.11
ZWS-7-26D.14.fid
C13CPD CDCl3 {D:\NMR400\02T4} nmr-new 5
F
N
H
8d
13C NMR (100 MHz, Chloroform-d)
f1 (ppm)

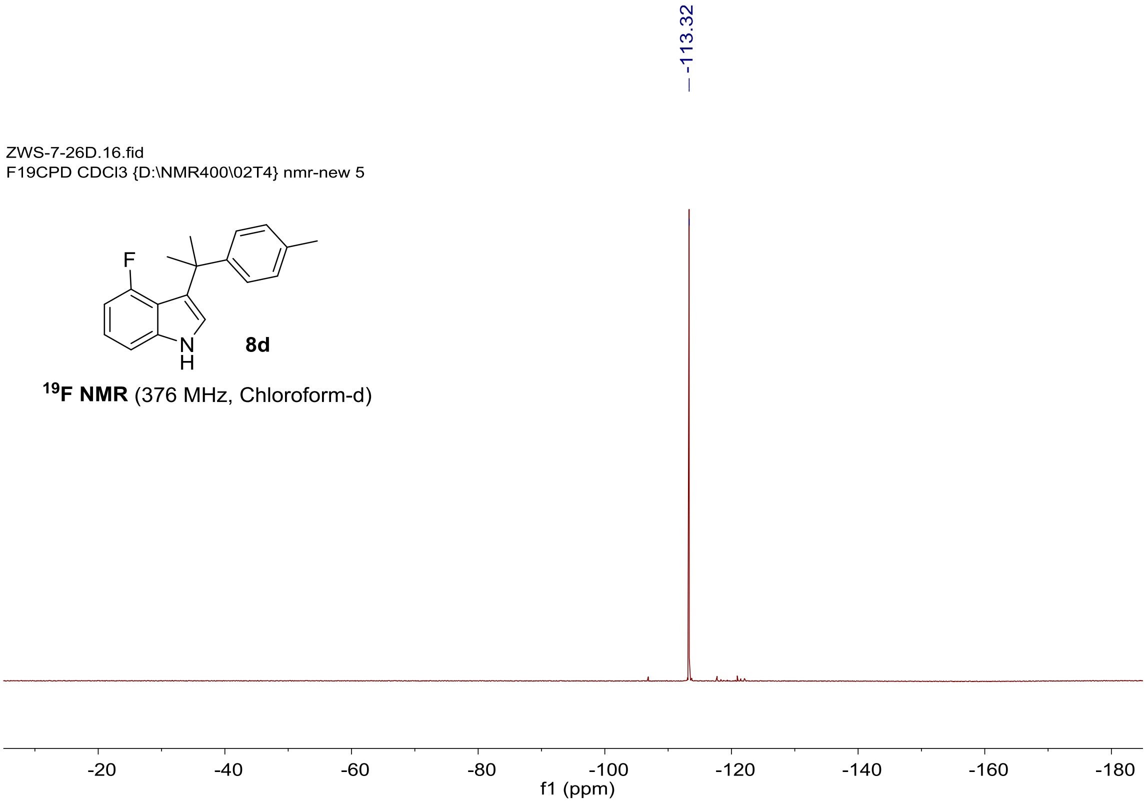
-113.32
ZWS-7-26D.16.fid
F19CPD CDCl3 {D:\NMR400\02T4} nmr-new 5
F
N
H
8d
19F NMR (376 MHz, Chloroform-d)
-20
-40
-60
-80
-100
-120
-140
-160
-180
f1 (ppm)

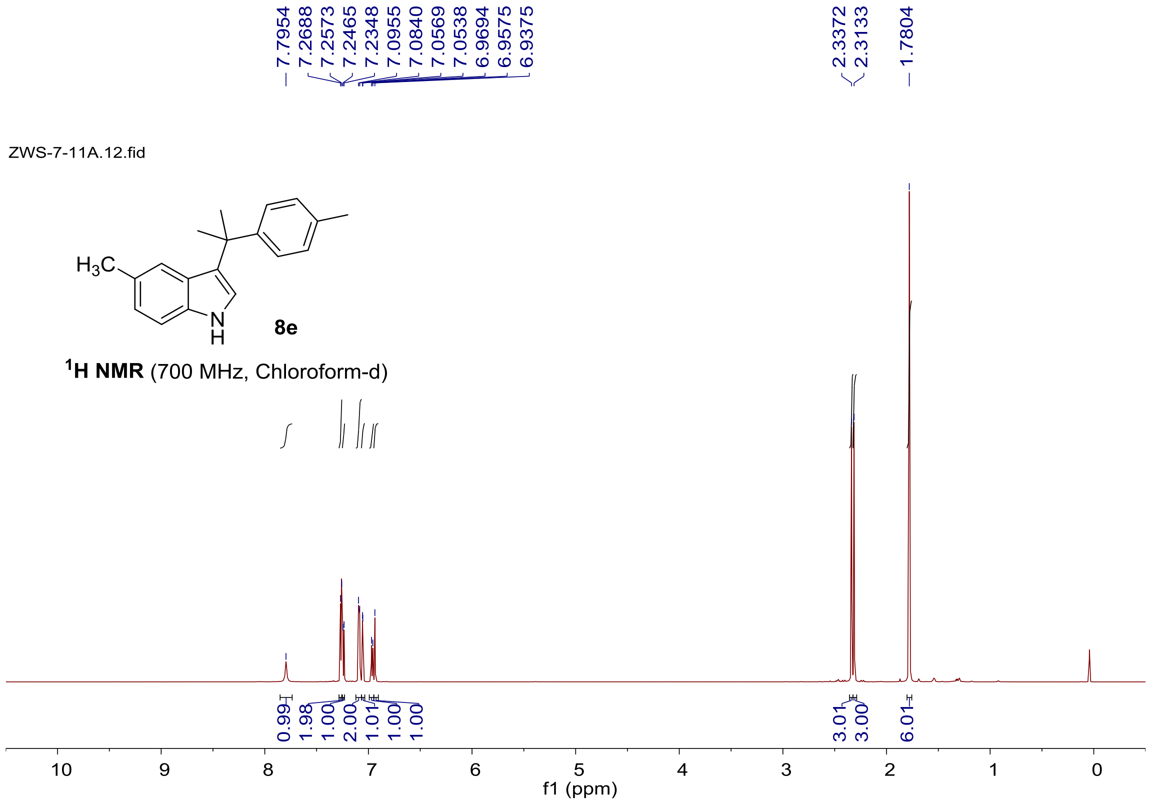
7.7954
7.2688
7.2573
7.2465
7.2348
7.0955
7.0840
7.0569
7.0538
6.9694
6.9575
6.9375
2.3372
2.3133
1.7804
ZWS-7-11A.12.fid
H3C
N
H
8e
1H NMR (700 MHz, Chloroform-d)
0.99
1.98
1.00
2.00
1.01
1.00
1.00
3.01
3.00
6.01
10 9 8 7 6 5 4 3 2 1 0
f1 (ppm)

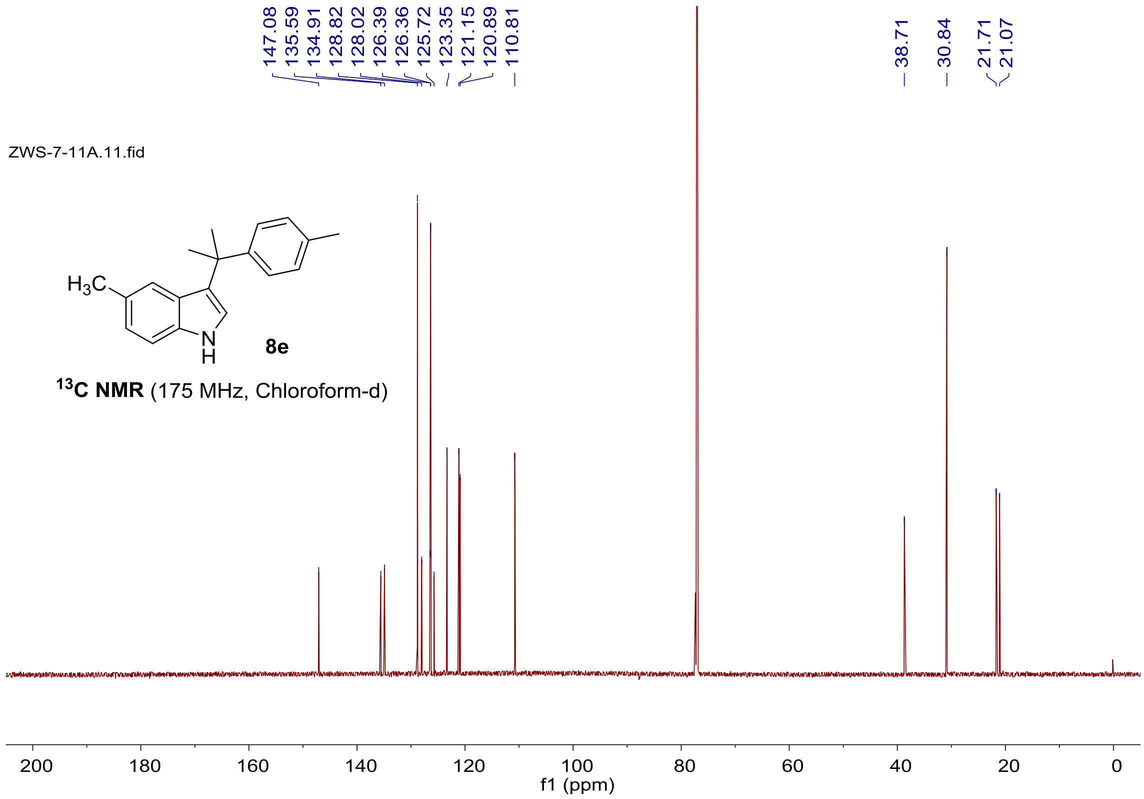
147.08
135.59
134.91
128.82
128.02
126.39
126.36
125.72
123.35
121.15
120.89
110.81
38.71
30.84
21.71
21.07
ZWS-7-11A.11.fid
H3C
N
H
8e
13C NMR (175 MHz, Chloroform-d)
200 180 160 140 120 100 80 60 40 20 0
f1 (ppm)

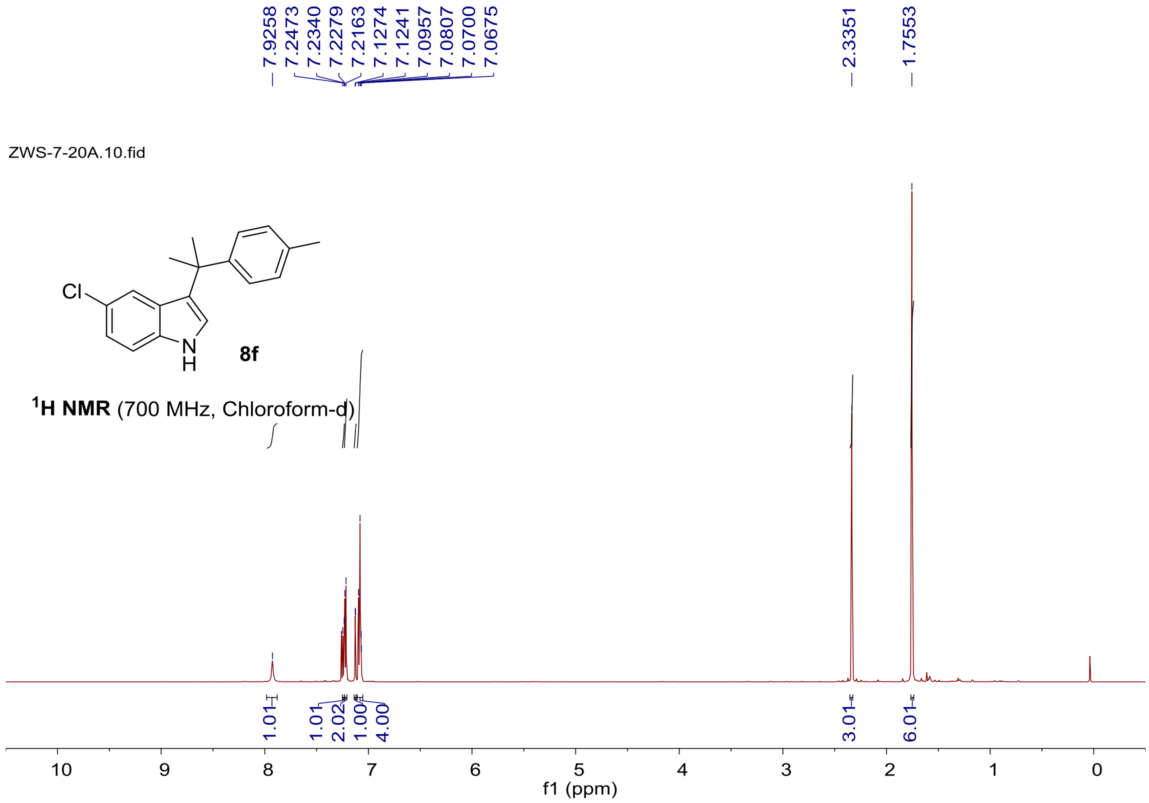
7.9258
7.2473
7.2340
7.2279
7.2163
7.1274
7.1241
7.0957
7.0807
7.0700
7.0675
2.3351
1.7553
ZWS-7-20A.10.fid
Cl
N
H
8f
¹H NMR (700 MHz, Chloroform-d)
1.01
1.01
2.02
1.00
4.00
3.01
6.01
10
9
8
7
6
5
4
3
2
1
0
f1 (ppm)

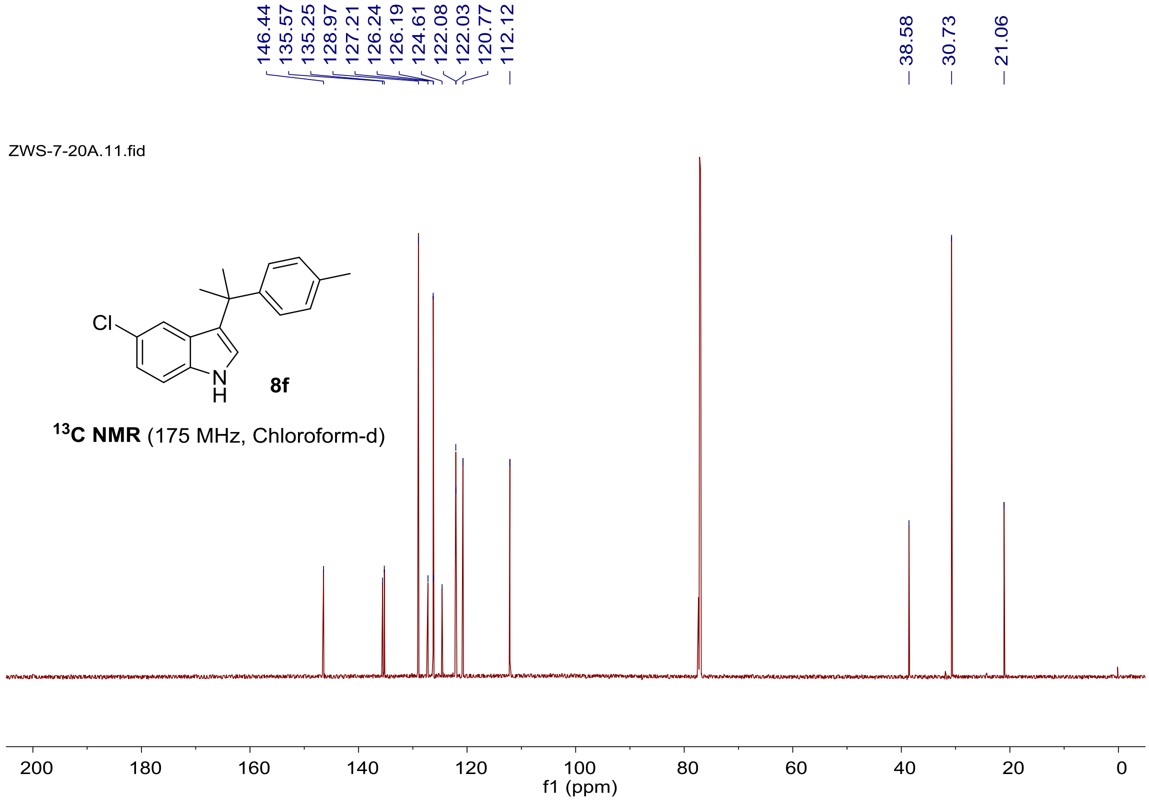
146.44
135.57
135.25
128.97
127.21
126.24
126.19
124.61
122.08
122.03
120.77
112.12
38.58
30.73
21.06
ZWS-7-20A.11.fid
Cl
N
H
8f
¹³C NMR (175 MHz, Chloroform-d)
200
180
160
140
120
100
80
60
40
20
0
f1 (ppm)

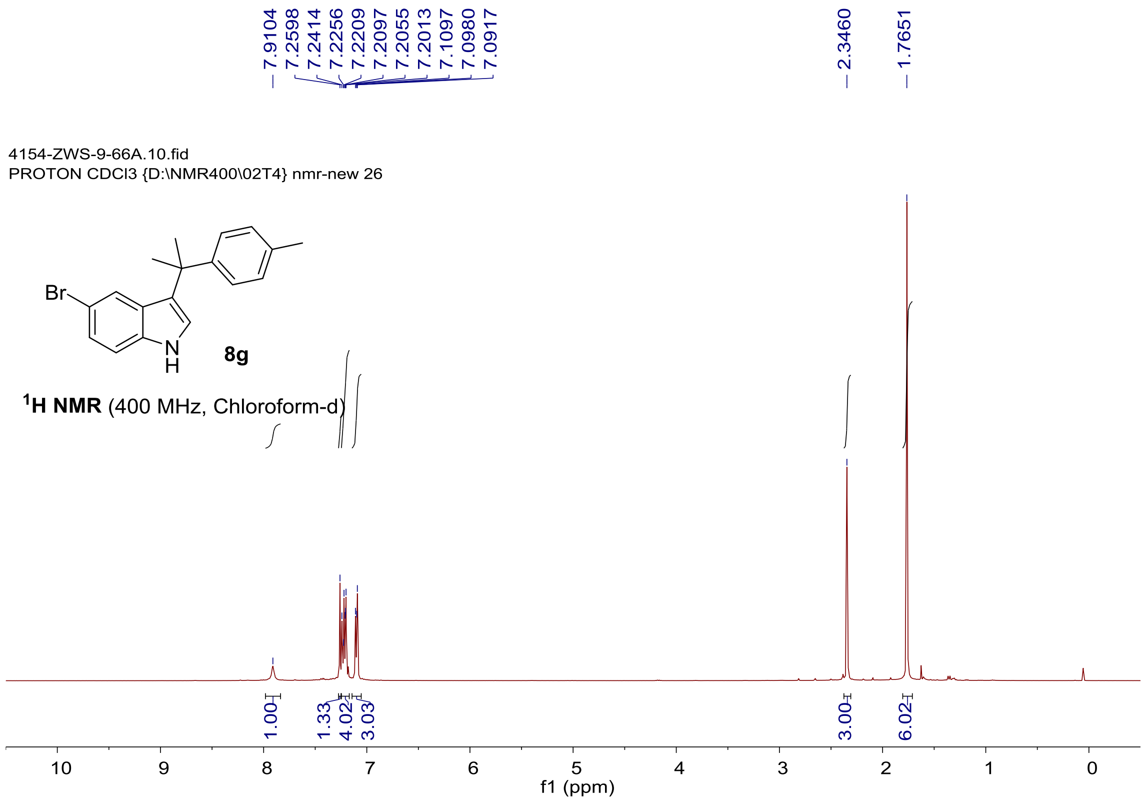
7.9104
7.2598
7.2414
7.2256
7.2209
7.2097
7.2055
7.2013
7.1097
7.0980
7.0917
2.3460
1.7651
4154-ZWS-9-66A.10.fid
PROTON CDCl3 {D:\NMR400\02T4} nmr-new 26
Br
N
H
8g
1H NMR (400 MHz, Chloroform-d)
1.00
1.33
4.02
3.03
3.00
6.02
10
9
8
7
6
5
4
3
2
1
0
f1 (ppm)

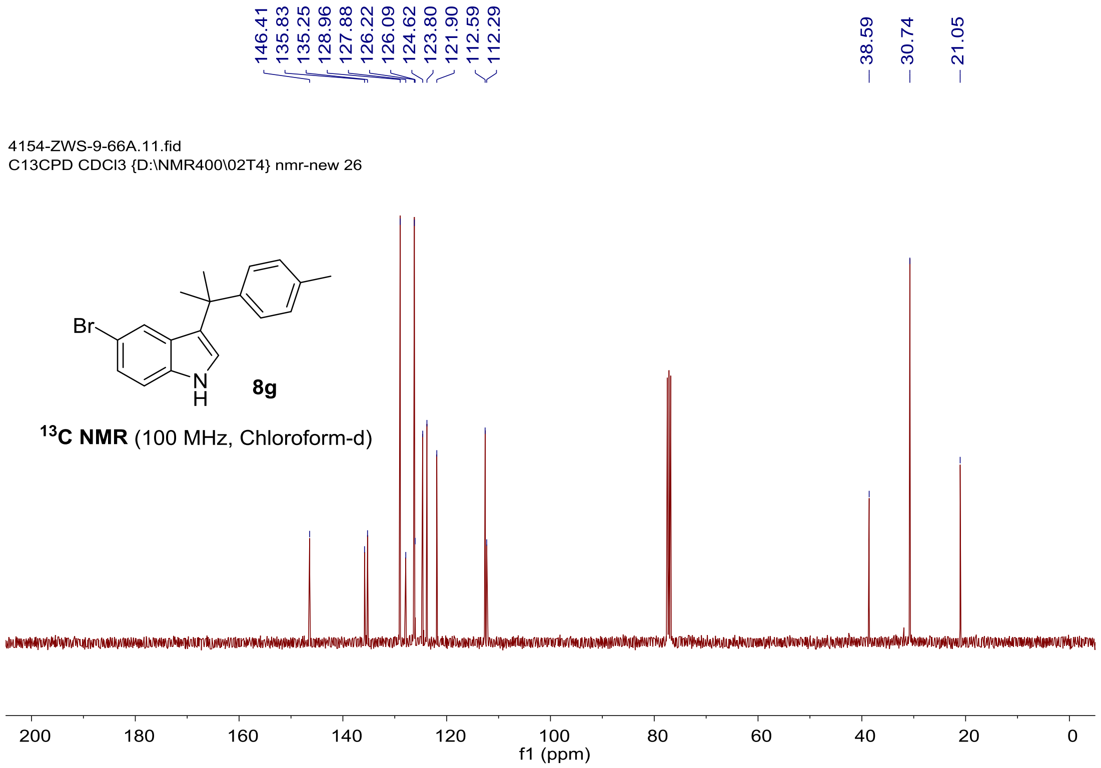
146.41
135.83
135.25
128.96
127.88
126.22
126.09
124.62
123.80
121.90
112.59
112.29
38.59
30.74
21.05
4154-ZWS-9-66A.11.fid
C13CPD CDCl3 {D:\NMR400\02T4} nmr-new 26
Br
N
H
8g
13C NMR (100 MHz, Chloroform-d)
200
180
160
140
120
100
80
60
40
20
0
f1 (ppm)

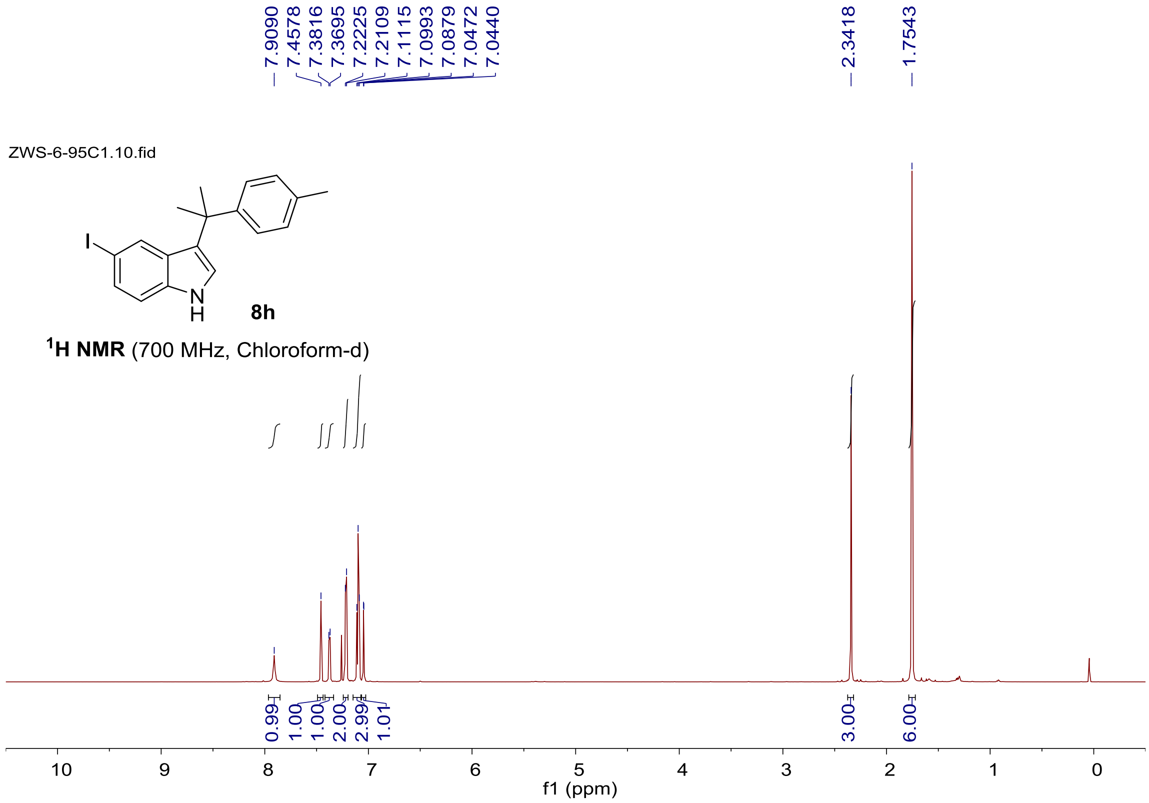
7.9090
7.4578
7.3816
7.3695
7.2225
7.2109
7.1115
7.0993
7.0879
7.0472
7.0440
2.3418
1.7543
ZWS-6-95C1.10.fid
I
N
H
8h
1H NMR (700 MHz, Chloroform-d)
0.99
1.00
1.00
2.00
2.99
1.01
3.00
6.00
10
9
8
7
6
5
4
3
2
1
0
f1 (ppm)

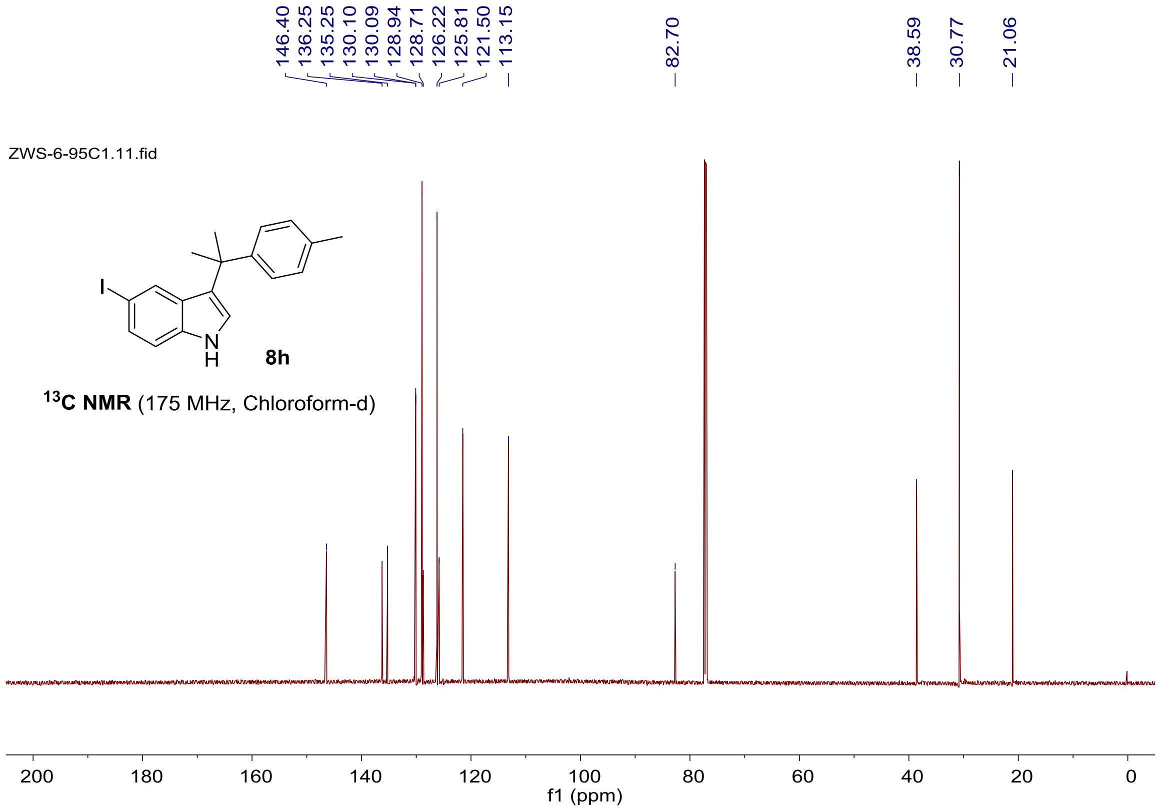
146.40
136.25
135.25
130.10
130.09
128.94
128.71
126.22
125.81
121.50
113.15
82.70
38.59
30.77
21.06
ZWS-6-95C1.11.fid
I
N
H
8h
13C NMR (175 MHz, Chloroform-d)
200
180
160
140
120
100
80
60
40
20
0
f1 (ppm)

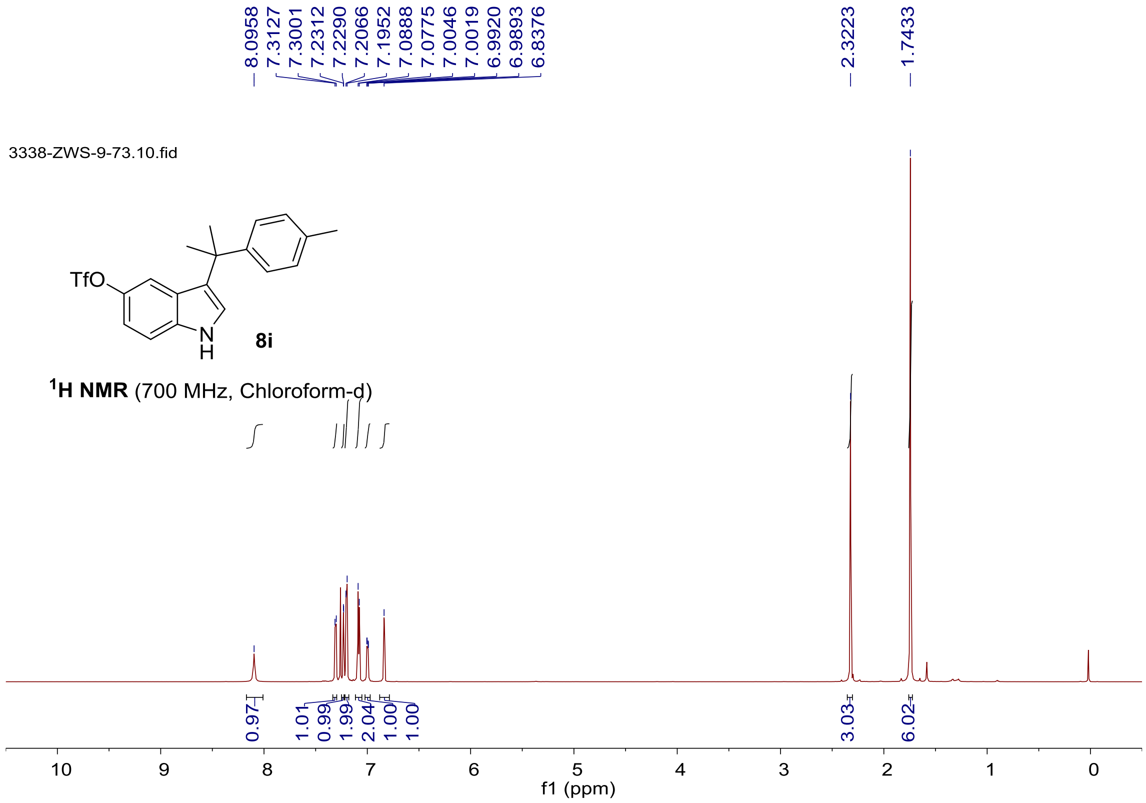
8.0958
7.3127
7.3001
7.2312
7.2290
7.2066
7.1952
7.0888
7.0775
7.0046
7.0019
6.9920
6.9893
6.8376
2.3223
1.7433
3338-ZWS-9-73.10.fid
TfO
N
H
8i
^{1}H NMR (700 MHz, Chloroform-d)
0.97
1.01
0.99
1.99
2.04
1.00
1.00
3.03
6.02
10
9
8
7
6
5
4
3
2
1
0
f1 (ppm)

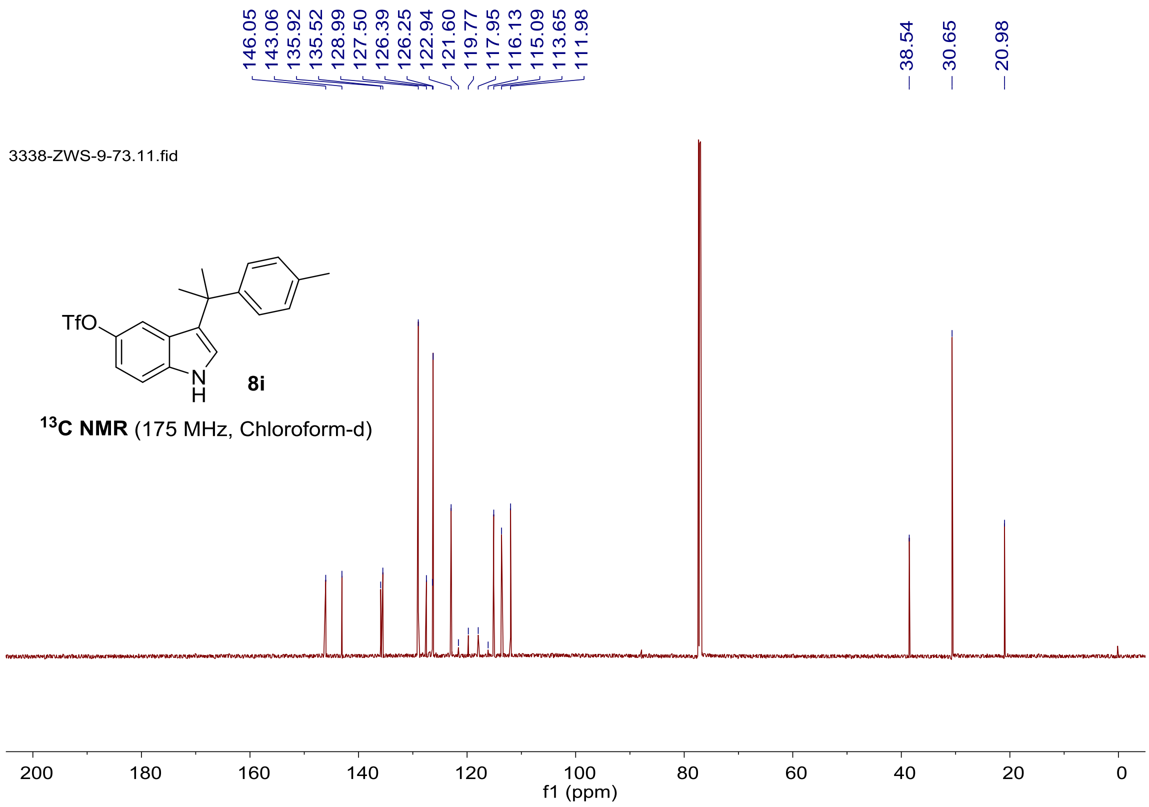
146.05
143.06
135.92
135.52
128.99
127.50
126.39
126.25
122.94
121.60
119.77
117.95
116.13
115.09
113.65
111.98
38.54
30.65
20.98
3338-ZWS-9-73.11.fid
TfO
N
H
8i
^{13}C NMR (175 MHz, Chloroform-d)
200
180
160
140
120
100
80
60
40
20
0
f1 (ppm)

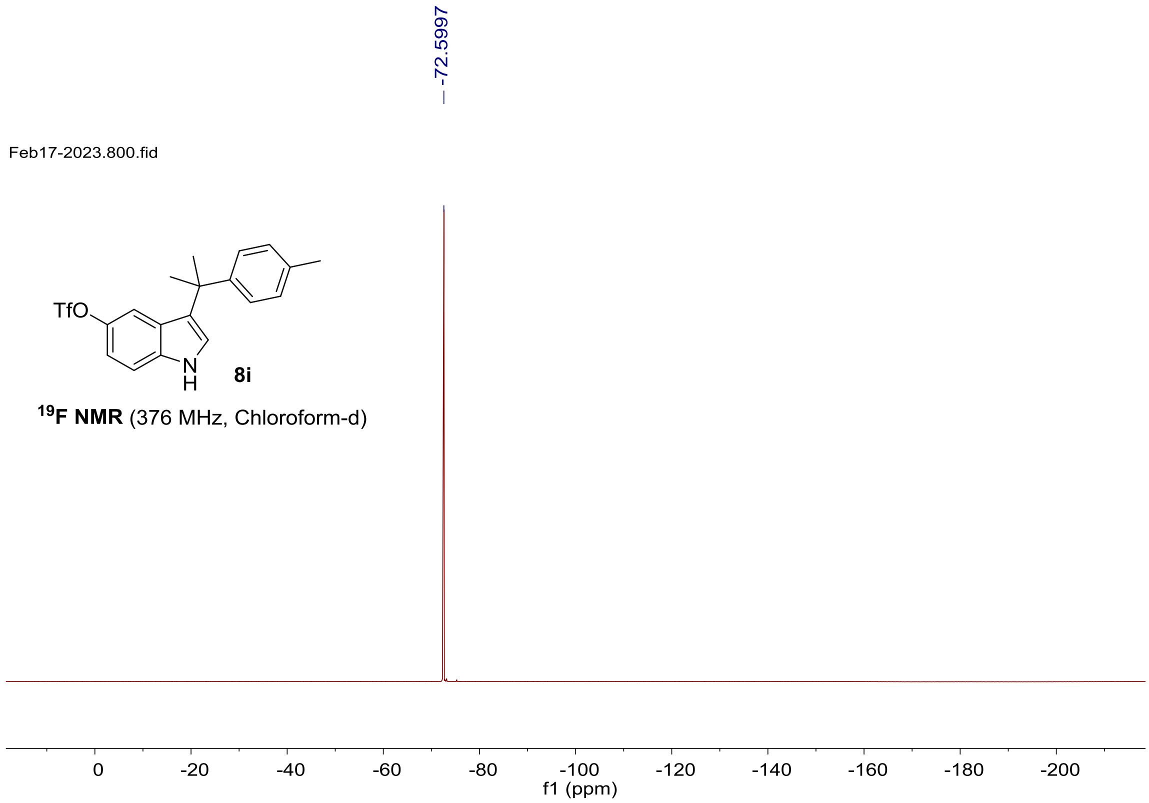

19F NMR (376 MHz, Chloroform-d)

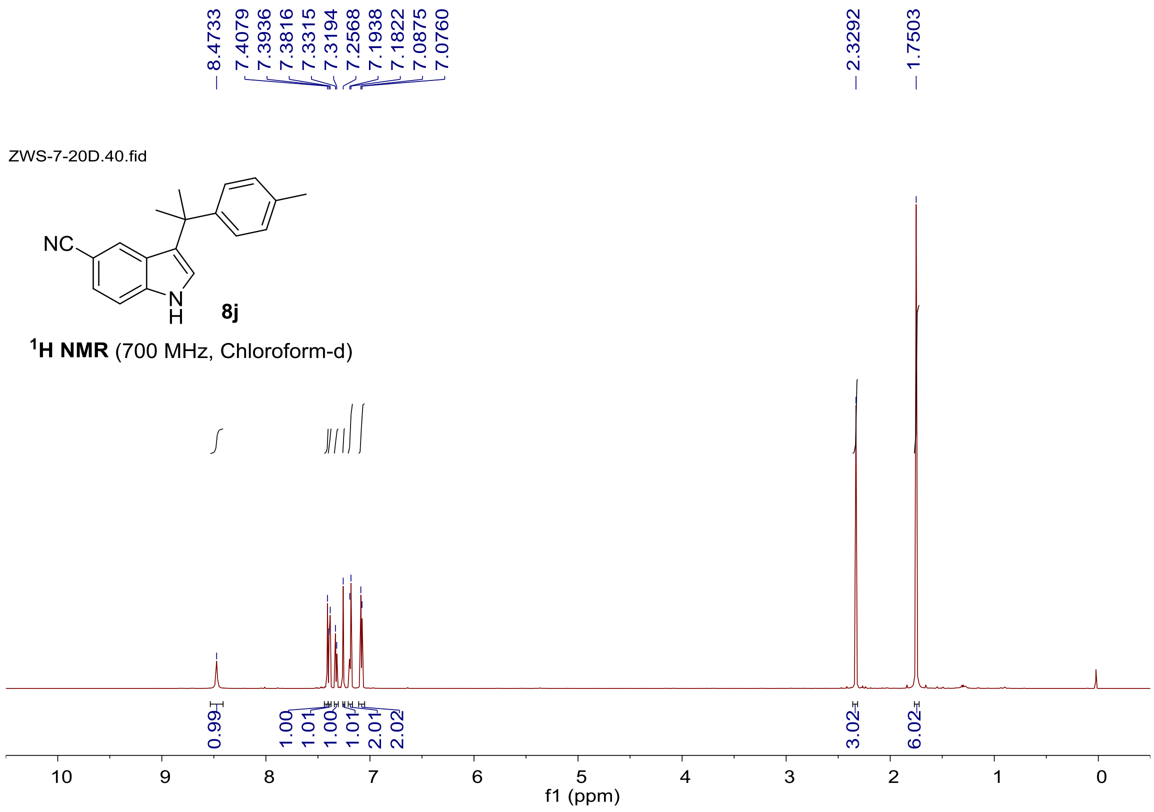

8.4733
7.4079
7.3936
7.3816
7.3315
7.3194
7.2568
7.1938
7.1822
7.0875
7.0760
2.3292
1.7503
ZWS-7-20D.40.fid
NC
N
H
8j
^{1}H NMR (700 MHz, Chloroform-d)
0.99
1.00
1.01
1.00
1.01
2.01
2.02
3.02
6.02
10 9 8 7 6 5 4 3 2 1 0
f1 (ppm)

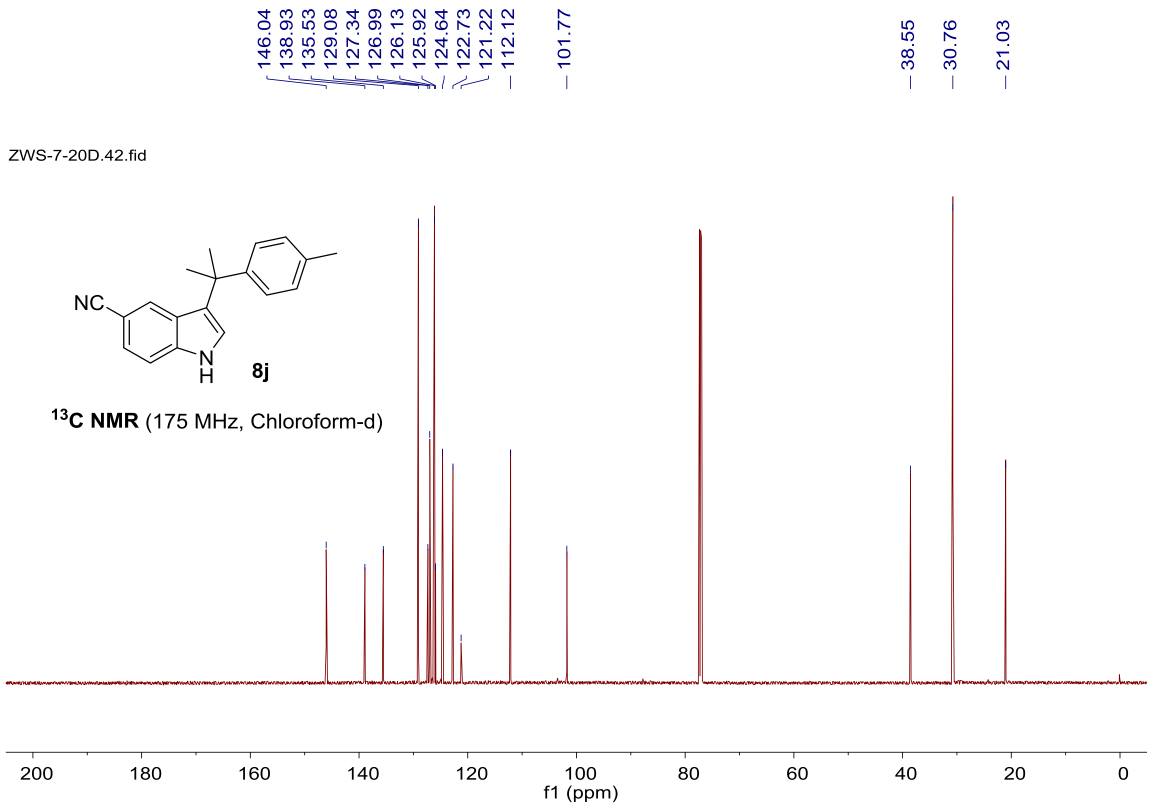

146.04
138.93
135.53
129.08
127.34
126.99
126.13
125.92
124.64
122.73
121.22
112.12
101.77
38.55
30.76
21.03
ZWS-7-20D.42.fid
NC
N
H
8j
^{13}C NMR (175 MHz, Chloroform-d)
200 180 160 140 120 100 80 60 40 20 0
f1 (ppm)

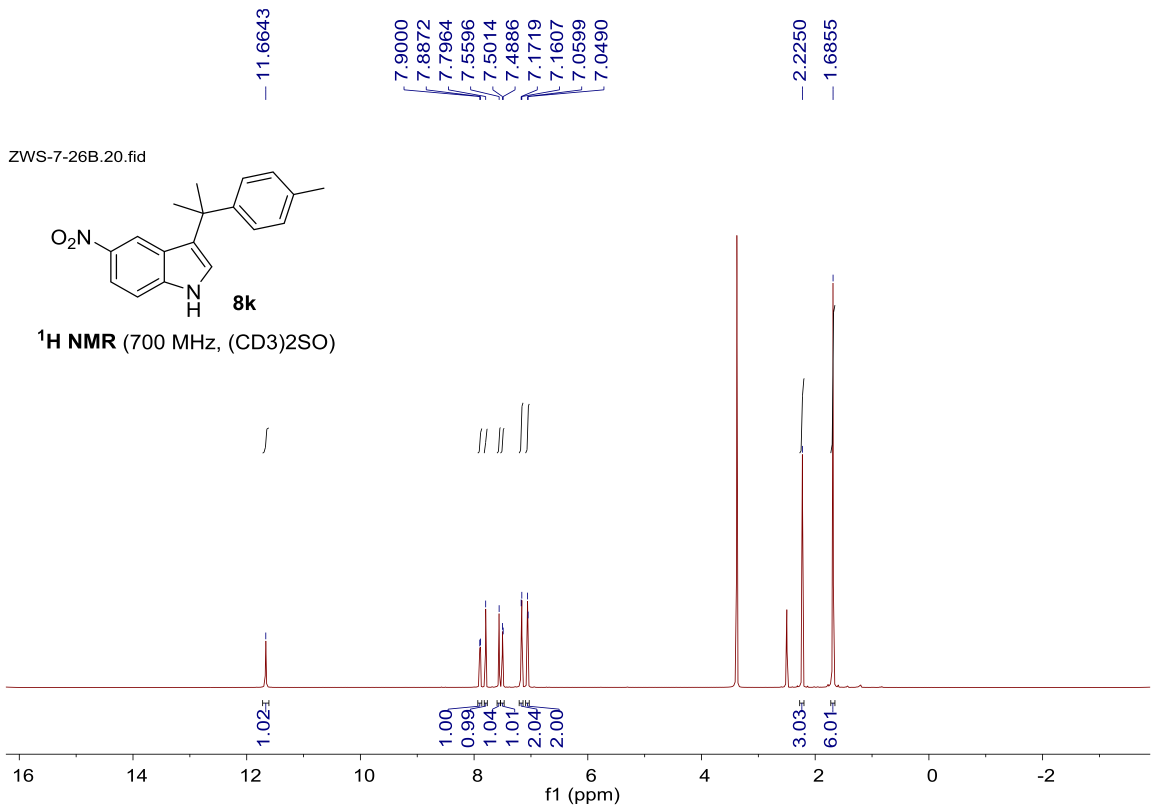

8k

^{1}H NMR (700 MHz, (CD3)2SO)

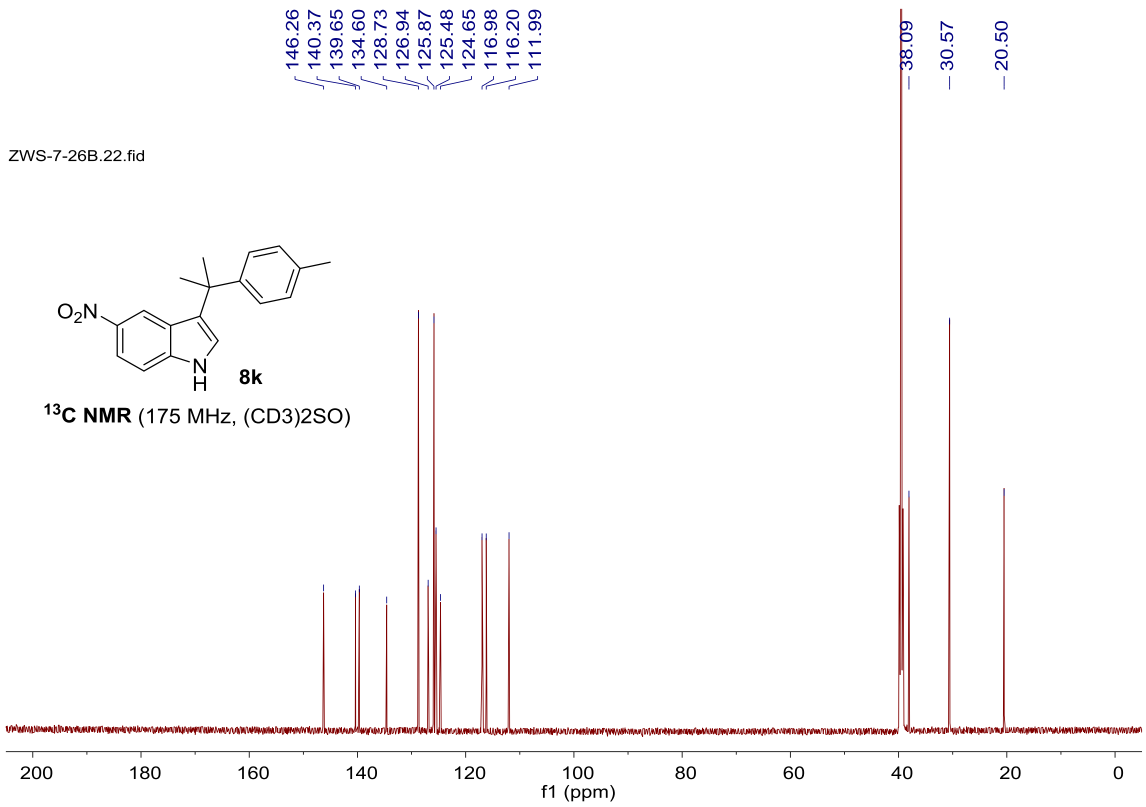

8k

^{13}C NMR (175 MHz, (CD3)2SO)

ZWS-7-26A.10.fid

8l

^{1}H NMR (700 MHz, Chloroform-d)

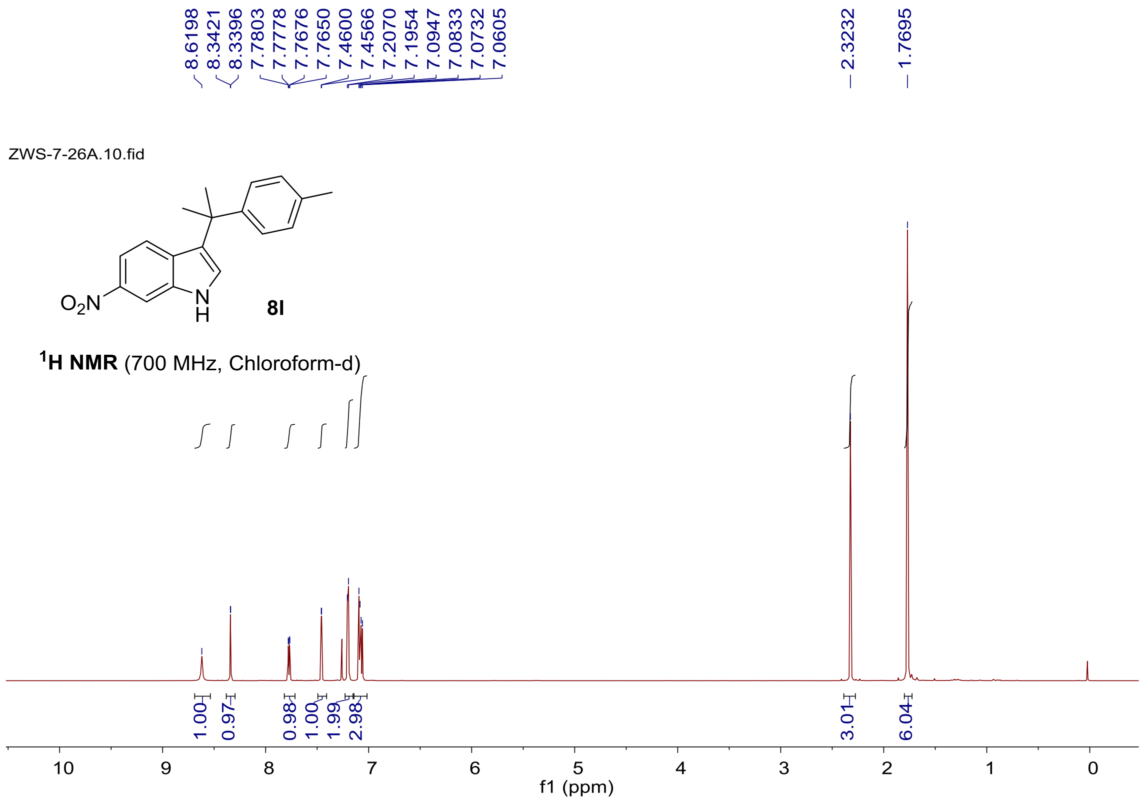

ZWS-7-26A.11.fid

8l

^{13}C NMR (175 MHz, Chloroform-d)

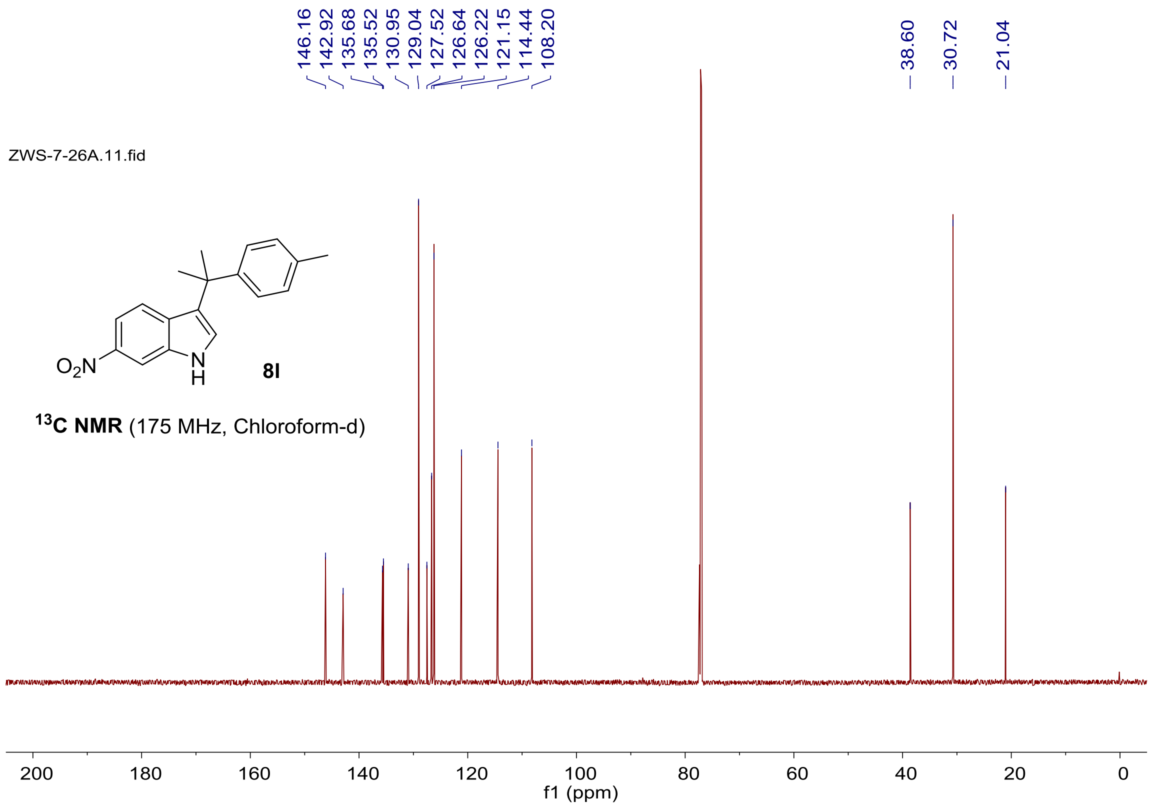

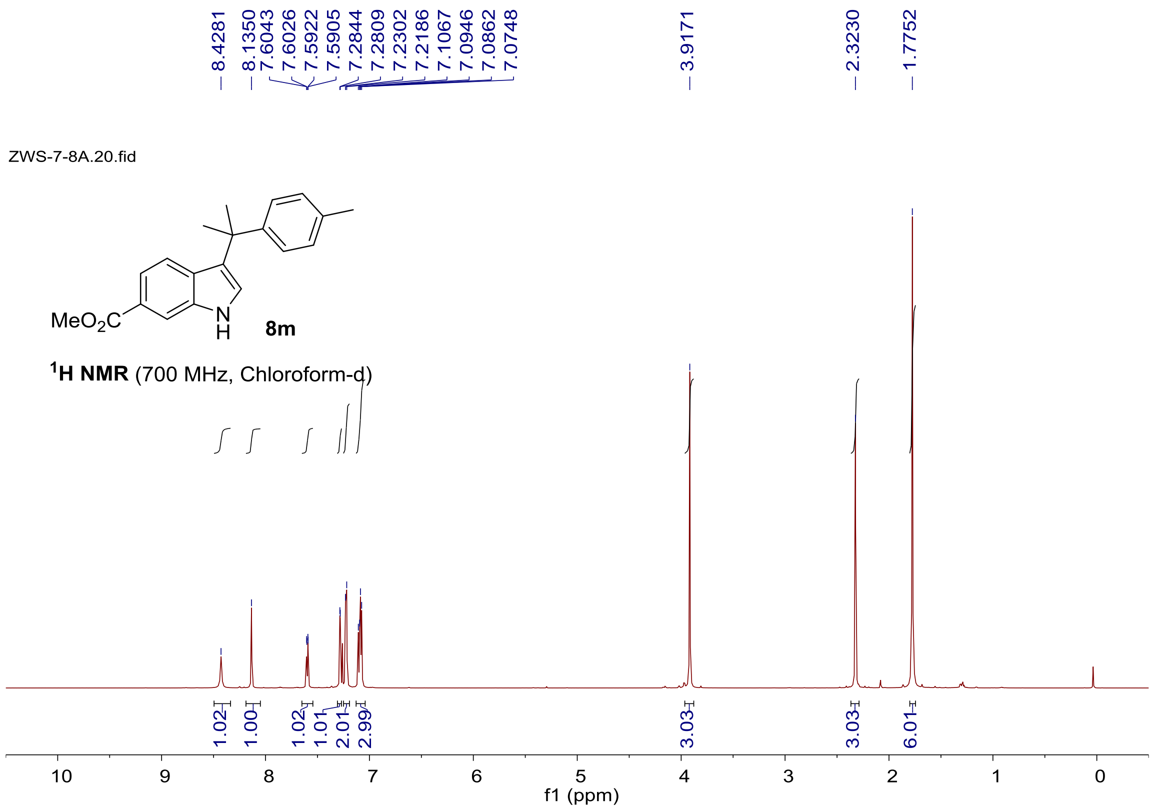
8.4281
8.1350
7.6043
7.6026
7.5922
7.5905
7.2844
7.2809
7.2302
7.2186
7.1067
7.0946
7.0862
7.0748
3.9171
2.3230
1.7752
ZWS-7-8A.20.fid
MeO2C
N
H
8m
1H NMR (700 MHz, Chloroform-d)
1.02
1.00
1.02
1.01
2.01
2.99
3.03
3.03
6.01
f1 (ppm)

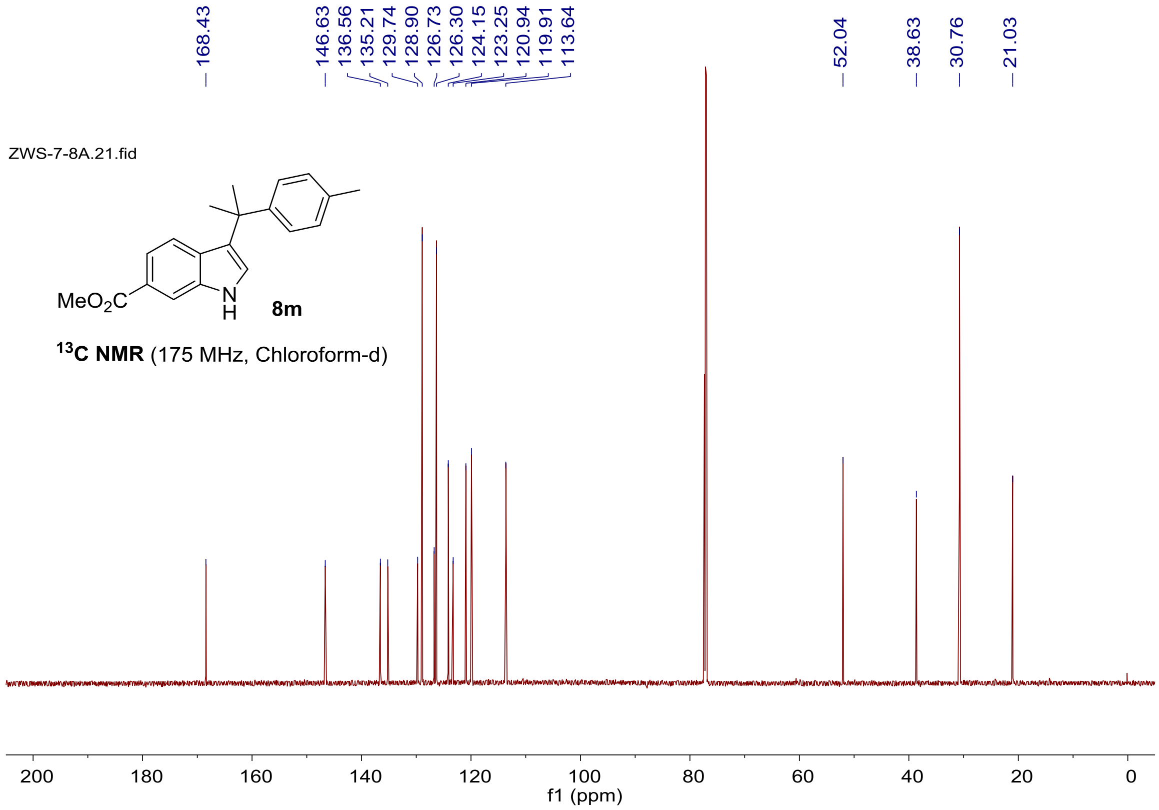
168.43
146.63
136.56
135.21
129.74
128.90
126.73
126.30
124.15
123.25
120.94
119.91
113.64
52.04
38.63
30.76
21.03
ZWS-7-8A.21.fid
MeO2C
N
H
8m
13C NMR (175 MHz, Chloroform-d)
f1 (ppm)

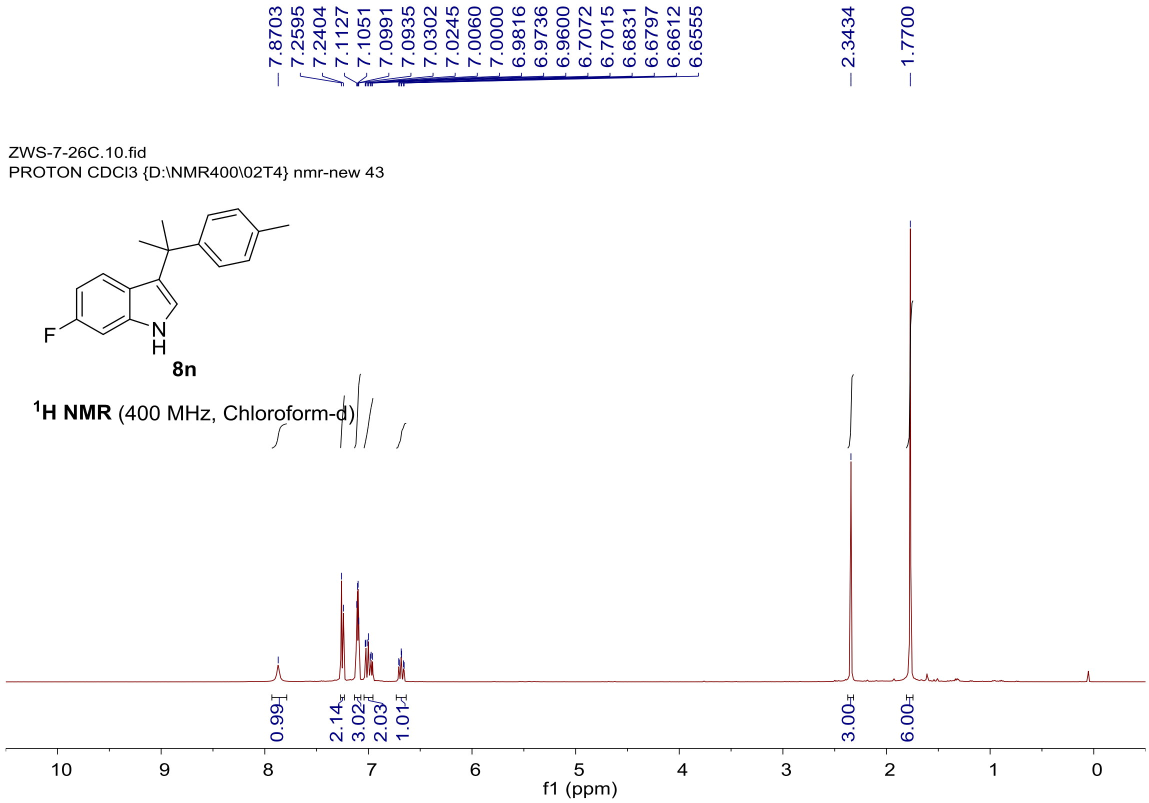
7.8703
7.2595
7.2404
7.1127
7.1051
7.0991
7.0935
7.0302
7.0245
7.0060
7.0000
6.9816
6.9736
6.9600
6.7072
6.7015
6.6831
6.6797
6.6612
6.6555
2.3434
1.7700
ZWS-7-26C.10.fid
PROTON CDCl3 {D:\NMR400\02T4} nmr-new 43
F
N
H
8n
1H NMR (400 MHz, Chloroform-d)
0.99
2.14
3.02
2.03
1.01
3.00
6.00
10
9
8
7
6
5
4
3
2
1
0
f1 (ppm)

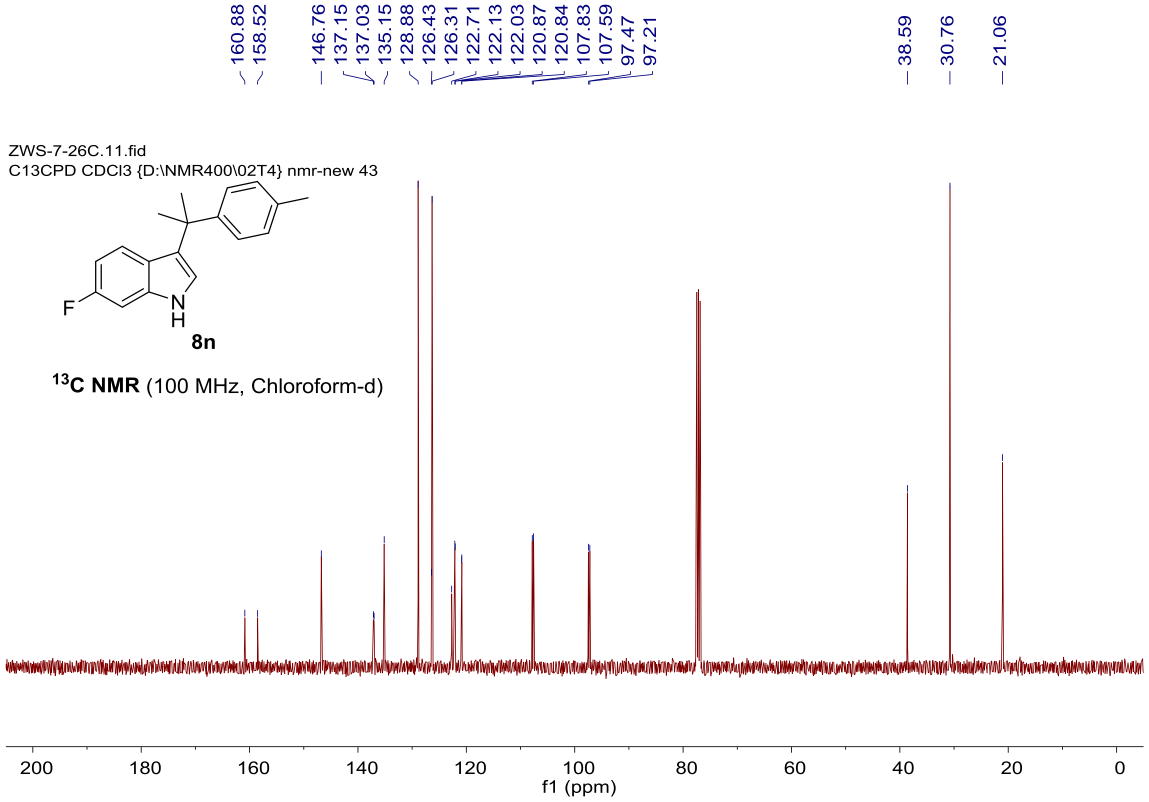
160.88
158.52
146.76
137.15
137.03
135.15
128.88
126.43
126.31
122.71
122.13
122.03
120.87
120.84
107.83
107.59
97.47
97.21
38.59
30.76
21.06
ZWS-7-26C.11.fid
C13CPD CDCl3 {D:\NMR400\02T4} nmr-new 43
F
N
H
8n
13C NMR (100 MHz, Chloroform-d)
200
180
160
140
120
100
80
60
40
20
0
f1 (ppm)

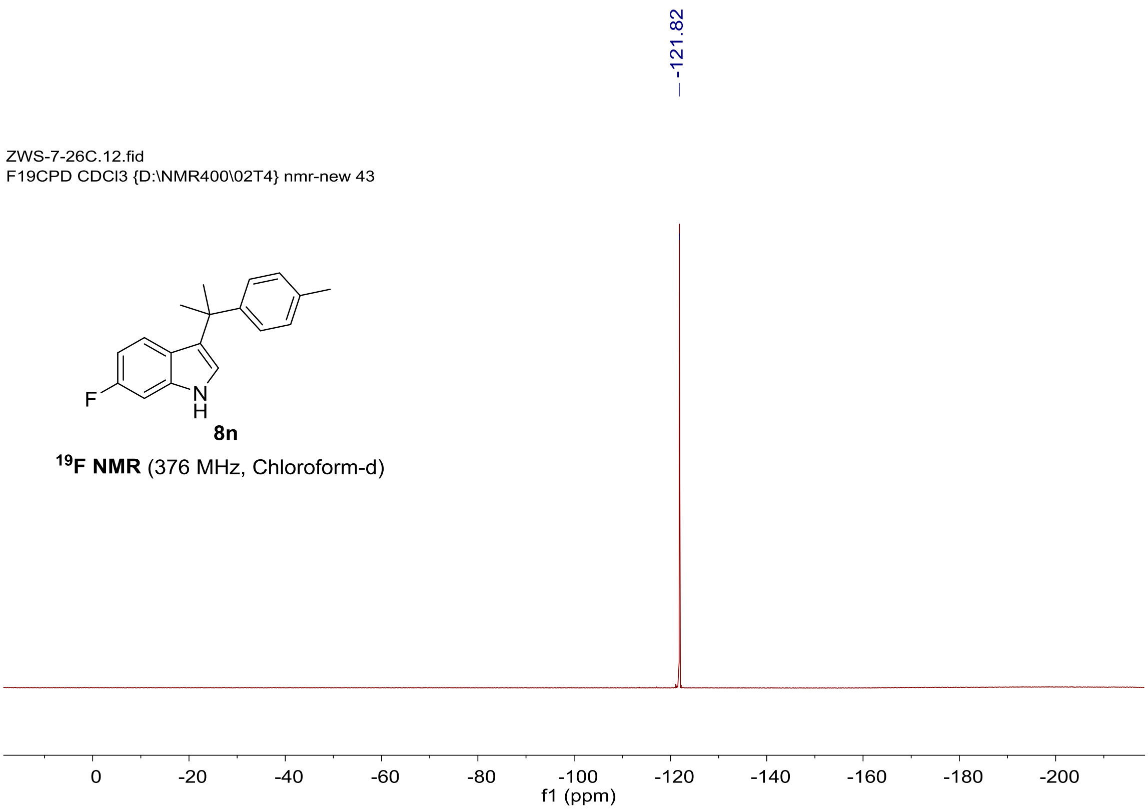

8n

^{19}F NMR (376 MHz, Chloroform-d)

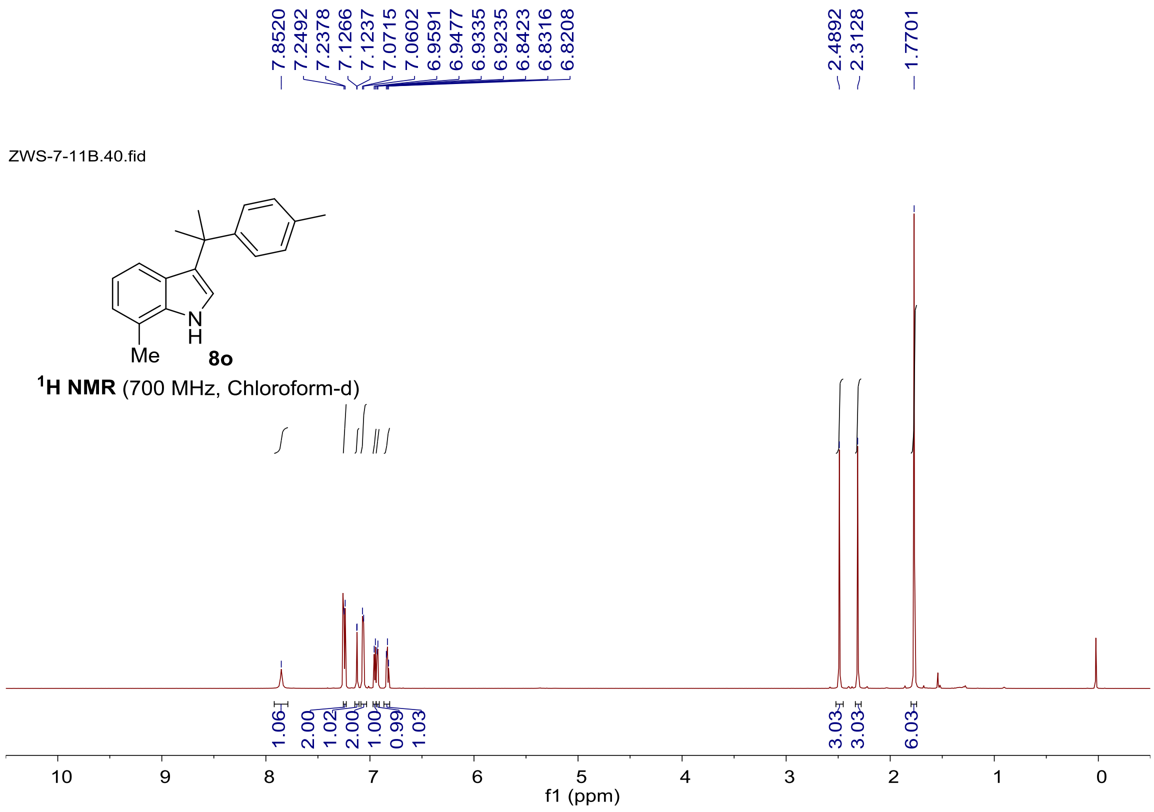

7.8520
7.2492
7.2378
7.1266
7.1237
7.0715
7.0602
6.9591
6.9477
6.9335
6.9235
6.8423
6.8316
6.8208
2.4892
2.3128
1.7701
ZWS-7-11B.40.fid
Me
8o
1H NMR (700 MHz, Chloroform-d)
1.06
2.00
1.02
2.00
1.00
0.99
1.03
3.03
3.03
6.03
f1 (ppm)

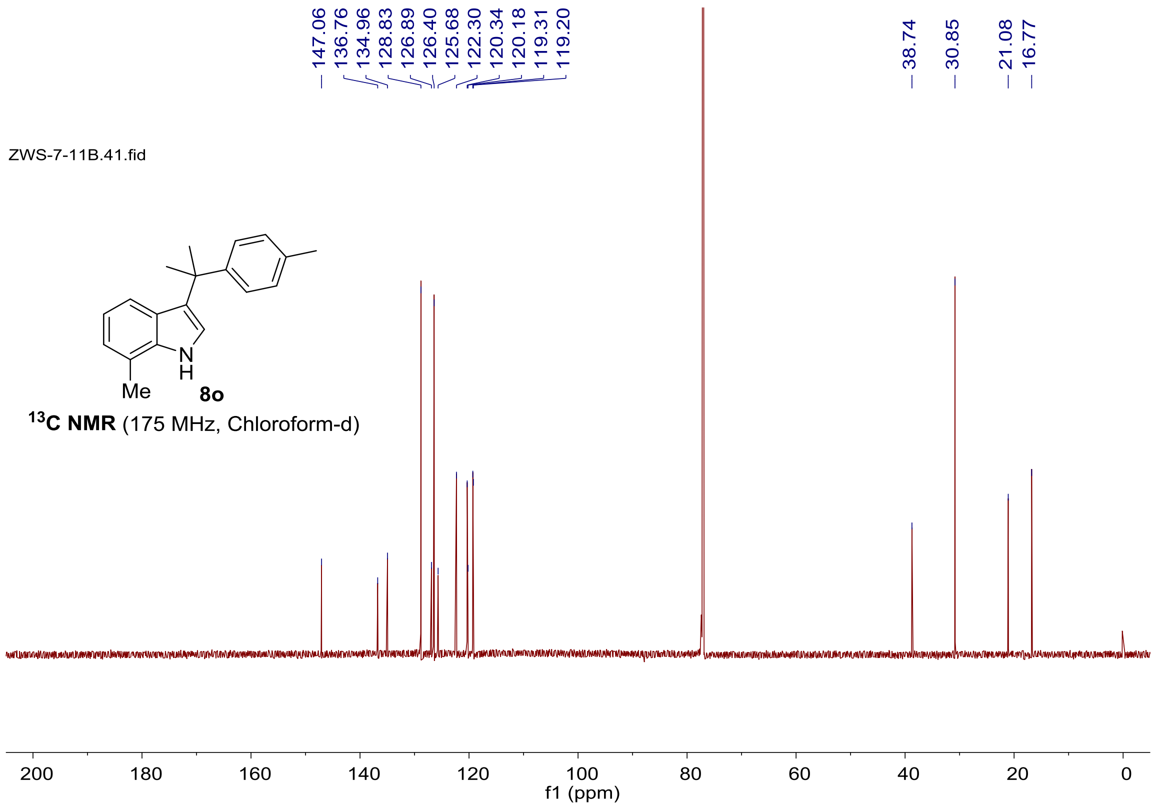

147.06
136.76
134.96
128.83
126.89
126.40
125.68
122.30
120.34
120.18
119.31
119.20
38.74
30.85
21.08
16.77
ZWS-7-11B.41.fid
Me
8o
13C NMR (175 MHz, Chloroform-d)
f1 (ppm)

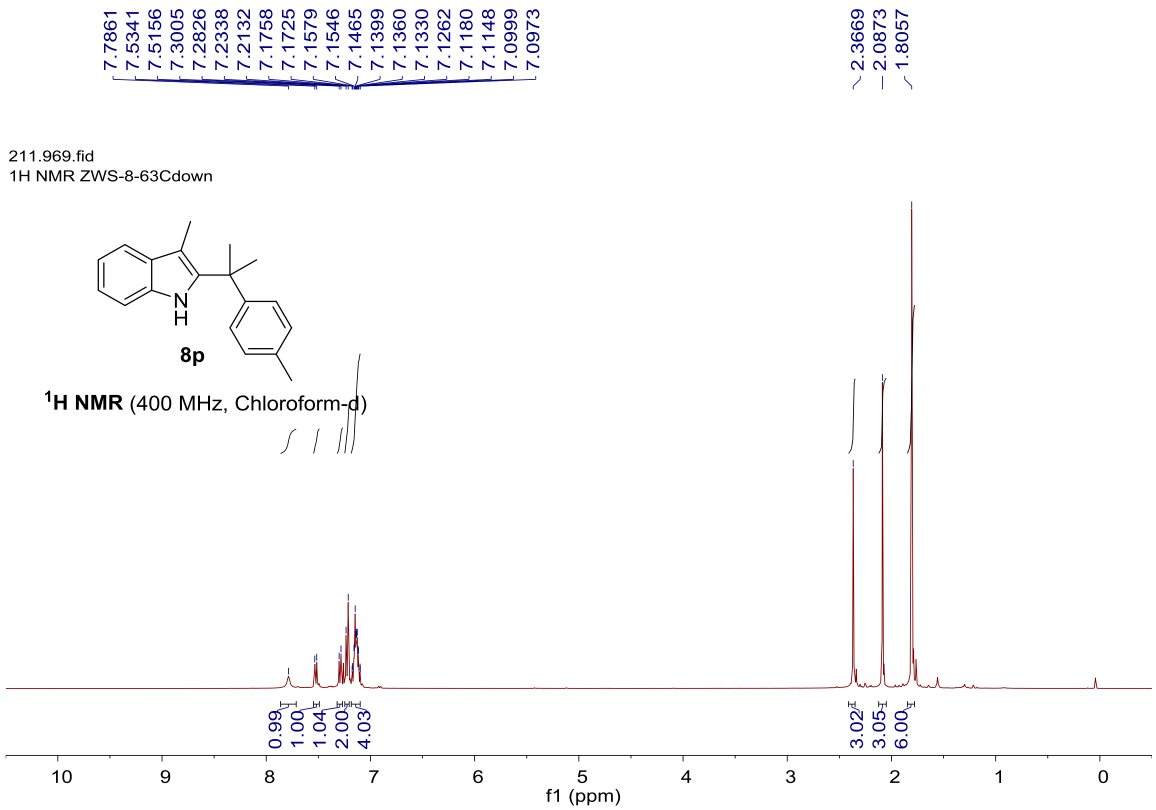
7.7861
7.5341
7.5156
7.3005
7.2826
7.2338
7.2132
7.1758
7.1725
7.1579
7.1546
7.1465
7.1399
7.1360
7.1330
7.1262
7.1180
7.1148
7.0999
7.0973
2.3669
2.0873
1.8057
211.969.fid
1H NMR ZWS-8-63Cdown
N
H
8p
1H NMR (400 MHz, Chloroform-d)
0.99
1.00
1.04
2.00
4.03
3.02
3.05
6.00
10
9
8
7
6
5
4
3
2
1
0
f1 (ppm)

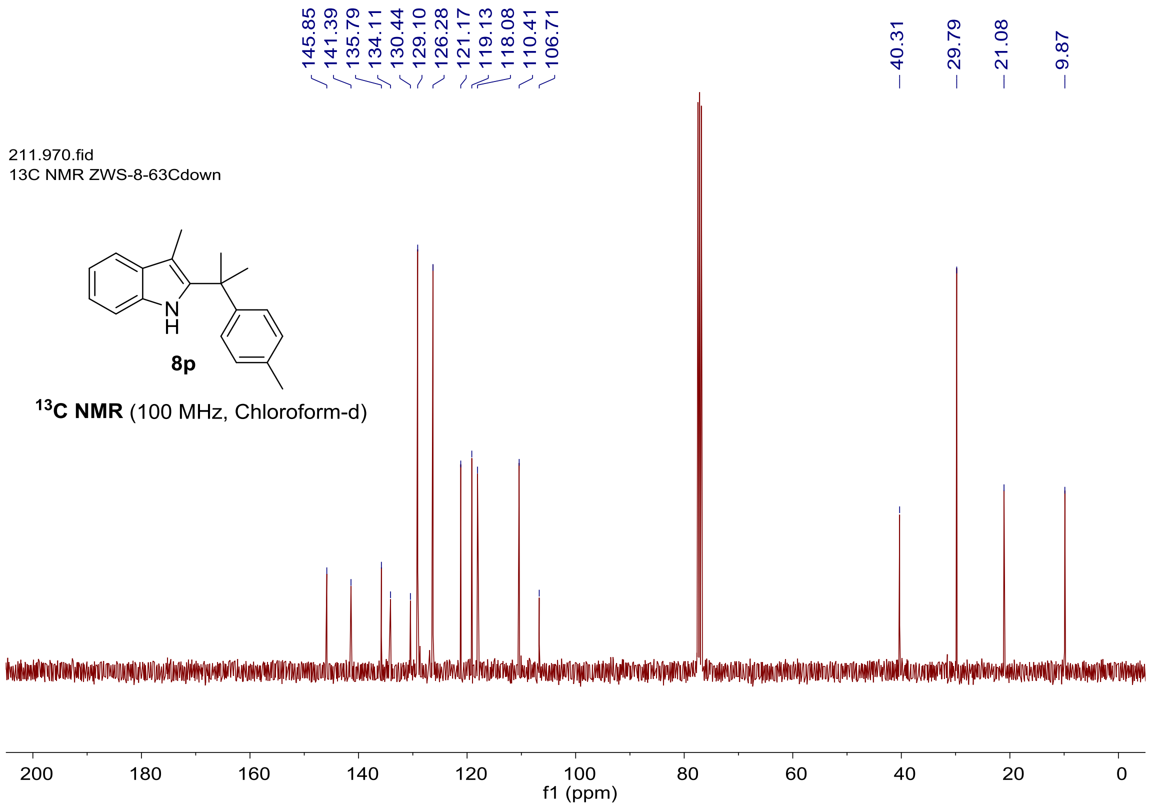
145.85
141.39
135.79
134.11
130.44
129.10
126.28
121.17
119.13
118.08
110.41
106.71
40.31
29.79
21.08
9.87
211.970.fid
13C NMR ZWS-8-63Cdown
N
H
8p
13C NMR (100 MHz, Chloroform-d)
200
180
160
140
120
100
80
60
40
20
0
f1 (ppm)

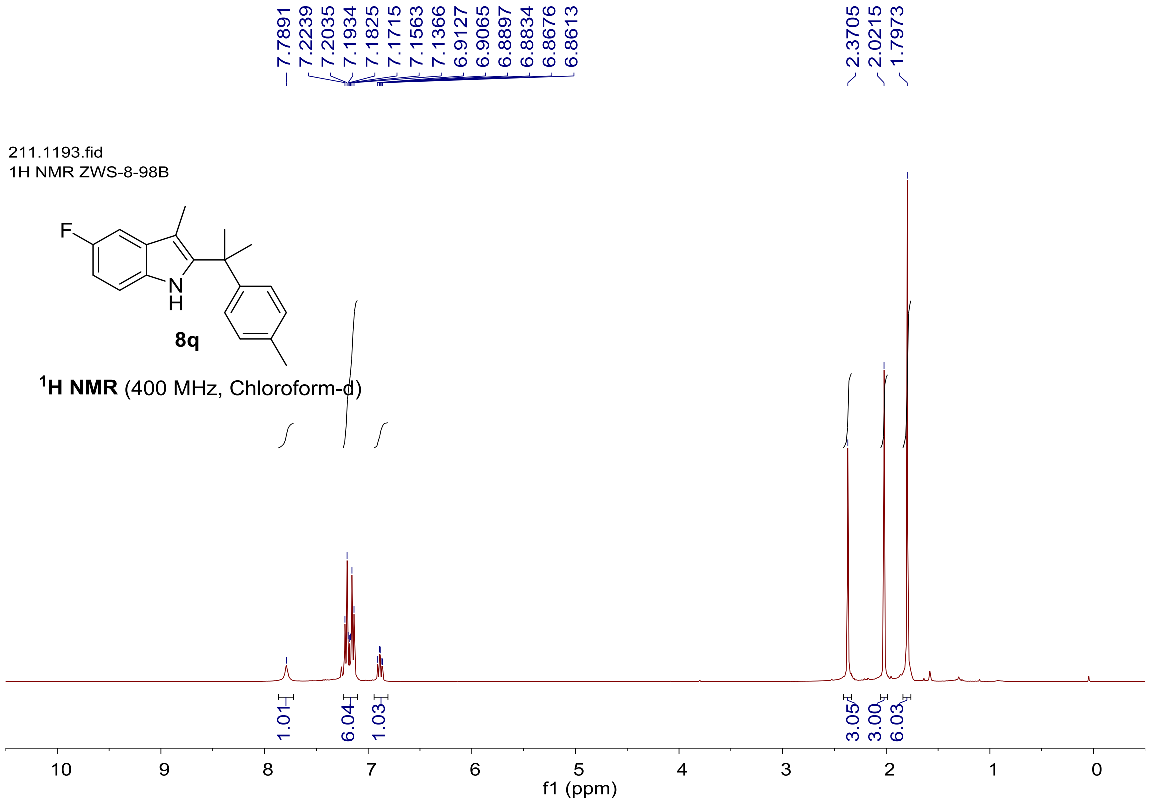
7.7891
7.2239
7.2035
7.1934
7.1825
7.1715
7.1563
7.1366
6.9127
6.9065
6.8897
6.8834
6.8676
6.8613
2.3705
2.0215
1.7973
211.1193.fid
1H NMR ZWS-8-98B
F
N
H
8q
1H NMR (400 MHz, Chloroform-d)
1.01
6.04
1.03
3.05
3.00
6.03
10
9
8
7
6
5
4
3
2
1
0
f1 (ppm)

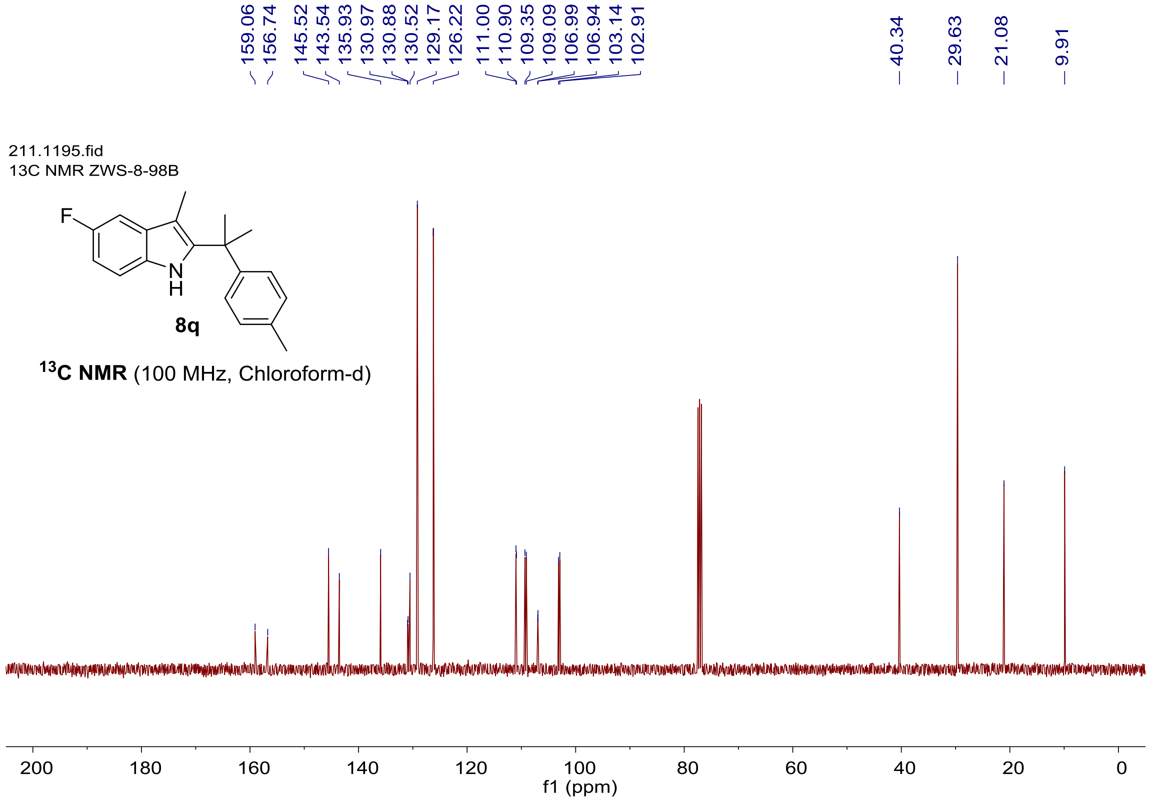
159.06
156.74
145.52
143.54
135.93
130.97
130.88
130.52
129.17
126.22
111.00
110.90
109.35
109.09
106.99
106.94
103.14
102.91
40.34
29.63
21.08
9.91
211.1195.fid
13C NMR ZWS-8-98B
F
N
H
8q
13C NMR (100 MHz, Chloroform-d)
200
180
160
140
120
100
80
60
40
20
0
f1 (ppm)

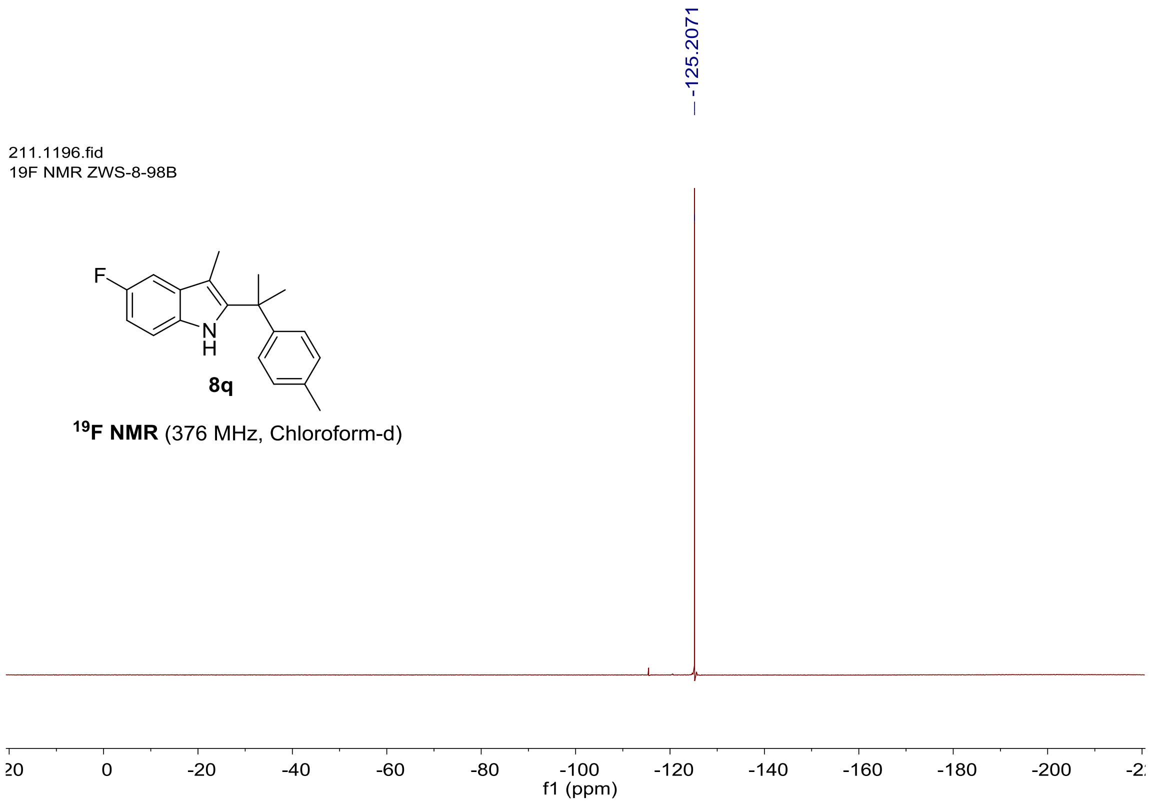
-125.2071
211.1196.fid
19F NMR ZWS-8-98B
F
N
H
8q
19F NMR (376 MHz, Chloroform-d)
20
0
-20
-40
-60
-80
-100
-120
-140
-160
-180
-200
f1 (ppm)

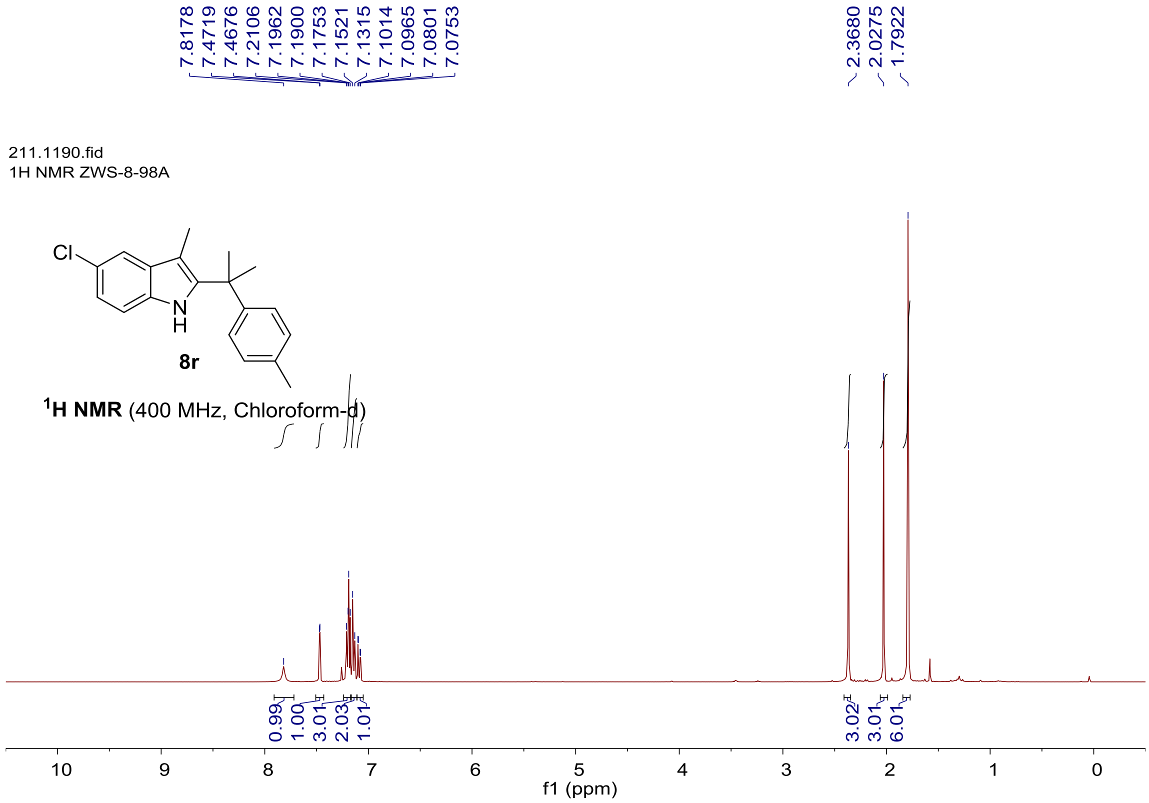
7.8178
7.4719
7.4676
7.2106
7.1962
7.1900
7.1753
7.1521
7.1315
7.1014
7.0965
7.0801
7.0753
2.3680
2.0275
1.7922
211.1190.fid
1H NMR ZWS-8-98A
Cl
N
H
8r
1H NMR (400 MHz, Chloroform-d)
0.99
1.00
3.01
2.03
1.01
3.02
3.01
6.01
10
9
8
7
6
5
4
3
2
1
0
f1 (ppm)

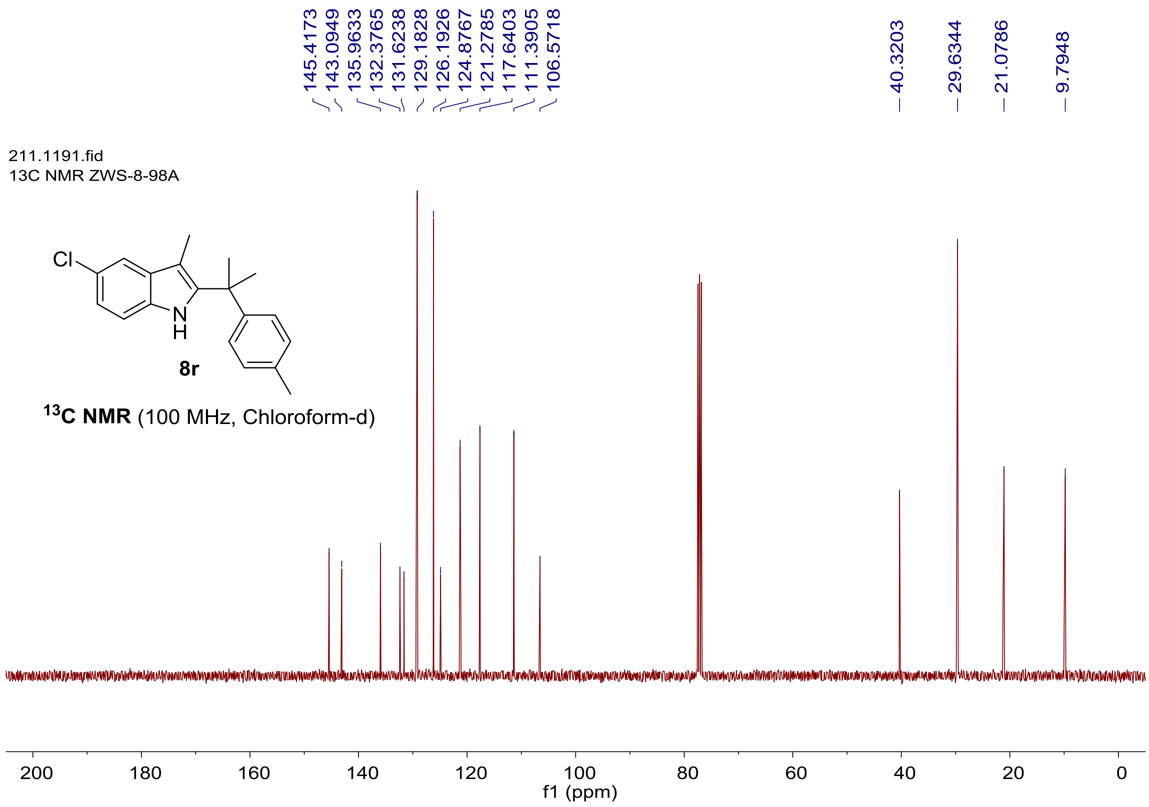
145.4173
143.0949
135.9633
132.3765
131.6238
129.1828
126.1926
124.8767
121.2785
117.6403
111.3905
106.5718
40.3203
29.6344
21.0786
9.7948
211.1191.fid
13C NMR ZWS-8-98A
Cl
N
H
8r
13C NMR (100 MHz, Chloroform-d)
200
180
160
140
120
100
80
60
40
20
0
f1 (ppm)

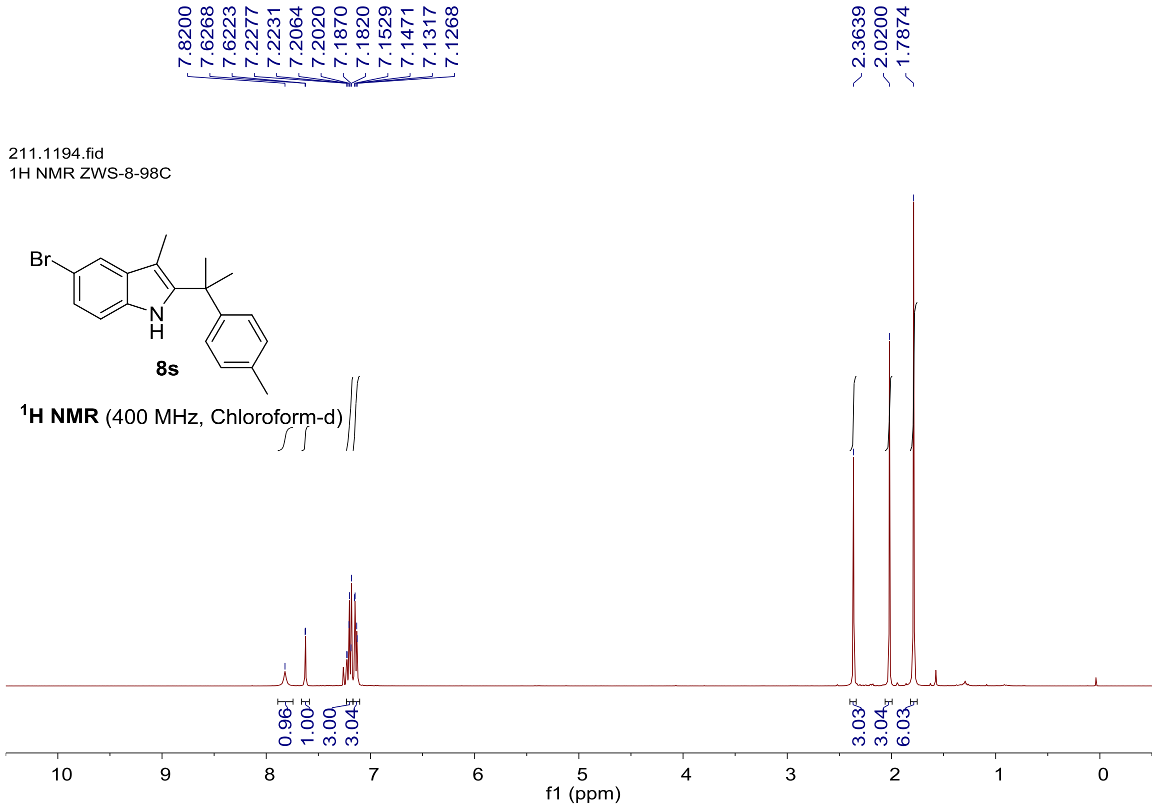
7.8200
7.6268
7.6223
7.2277
7.2231
7.2064
7.2020
7.1870
7.1820
7.1529
7.1471
7.1317
7.1268
2.3639
2.0200
1.7874
211.1194.fid
1H NMR ZWS-8-98C
Br
N
H
8s
1H NMR (400 MHz, Chloroform-d)
0.96
1.00
3.00
3.04
3.03
3.04
6.03
10
9
8
7
6
5
4
3
2
1
0
f1 (ppm)

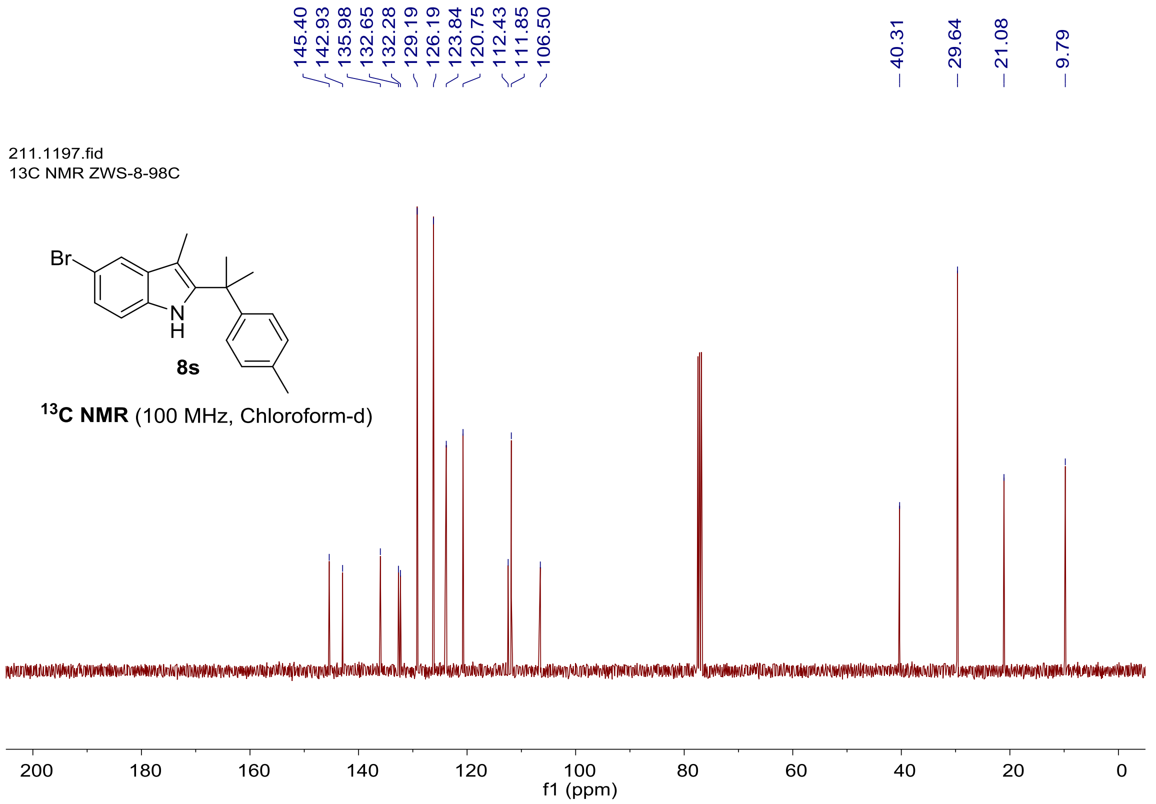
145.40
142.93
135.98
132.65
132.28
129.19
126.19
123.84
120.75
112.43
111.85
106.50
40.31
29.64
21.08
9.79
211.1197.fid
13C NMR ZWS-8-98C
Br
N
H
8s
13C NMR (100 MHz, Chloroform-d)
200
180
160
140
120
100
80
60
40
20
0
f1 (ppm)

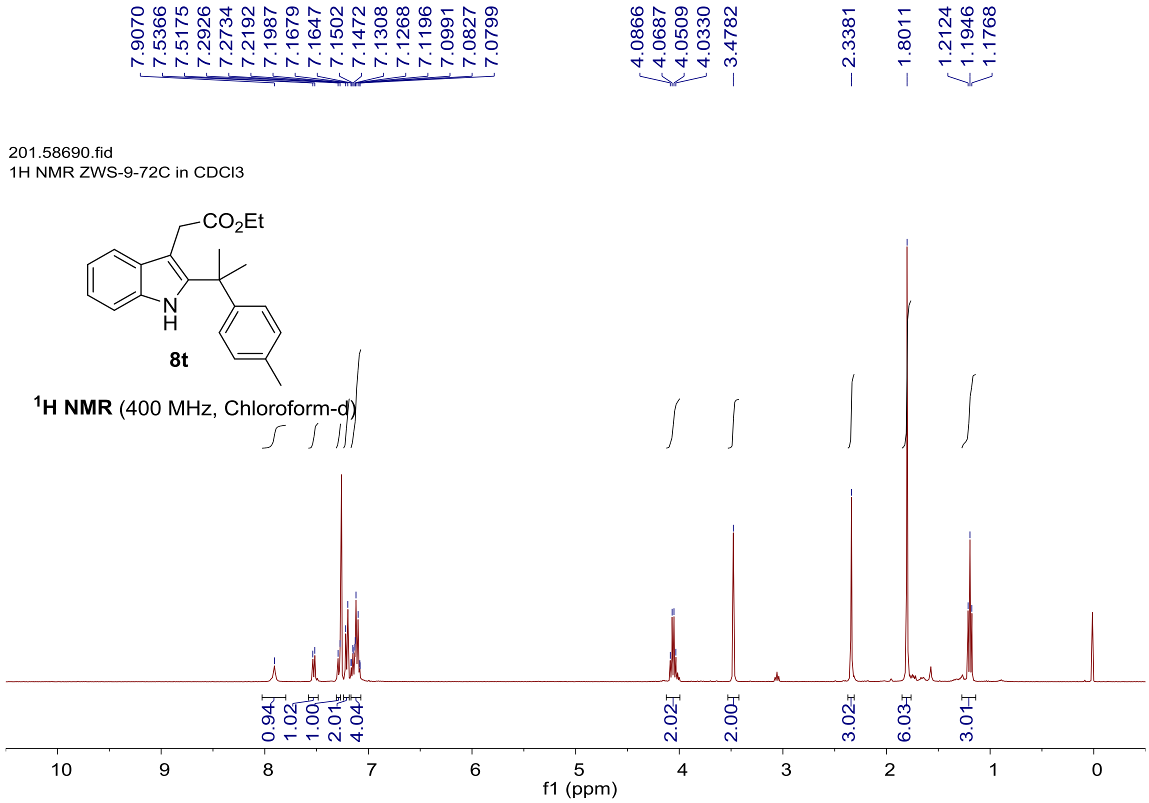
7.9070
7.5366
7.5175
7.2926
7.2734
7.2192
7.1987
7.1679
7.1647
7.1502
7.1472
7.1308
7.1268
7.1196
7.0991
7.0827
7.0799
4.0866
4.0687
4.0509
4.0330
3.4782
2.3381
1.8011
1.2124
1.1946
1.1768
201.58690.fid
1H NMR ZWS-9-72C in CDCl3
CO_2Et
N
H
8t
^{1}H NMR (400 MHz, Chloroform-d)
0.94
1.02
1.00
2.01
4.04
2.02
2.00
3.02
6.03
3.01
10
9
8
7
6
5
4
3
2
1
0
f1 (ppm)

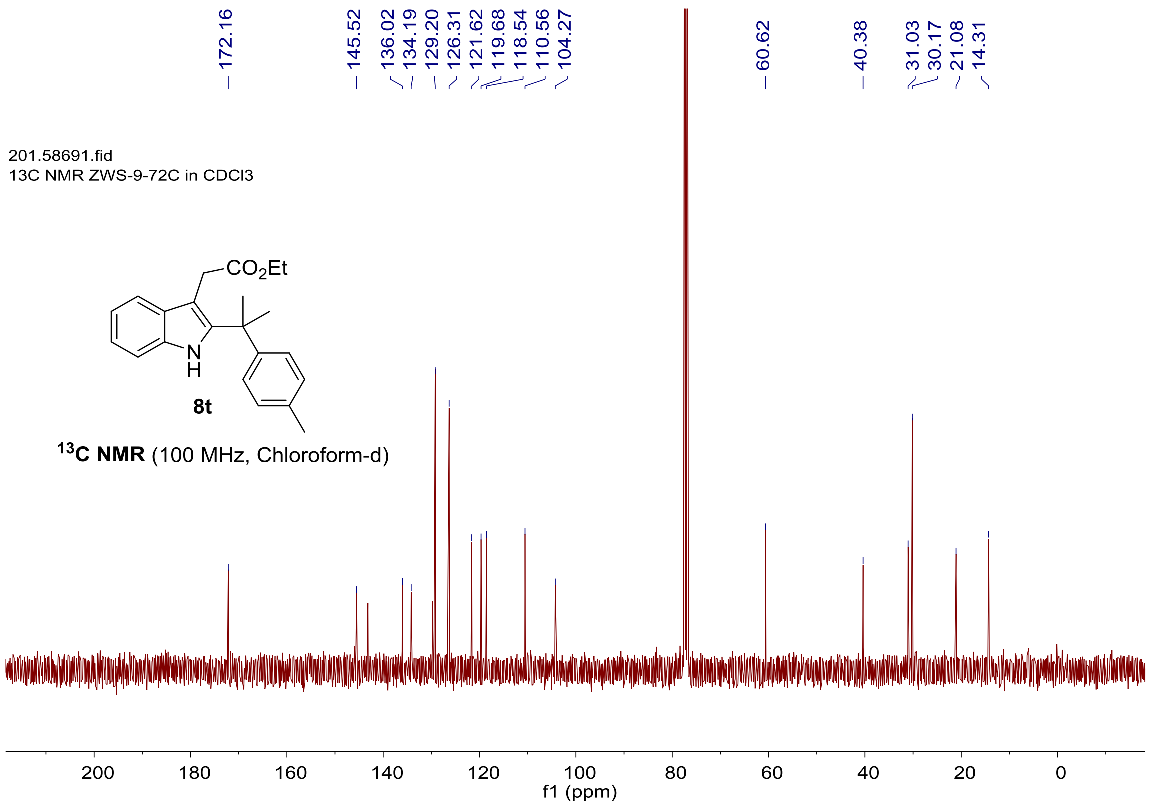
172.16
145.52
136.02
134.19
129.20
126.31
121.62
119.68
118.54
110.56
104.27
60.62
40.38
31.03
30.17
21.08
14.31
201.58691.fid
13C NMR ZWS-9-72C in CDCl3
CO_2Et
N
H
8t
^{13}C NMR (100 MHz, Chloroform-d)
200
180
160
140
120
100
80
60
40
20
0
f1 (ppm)

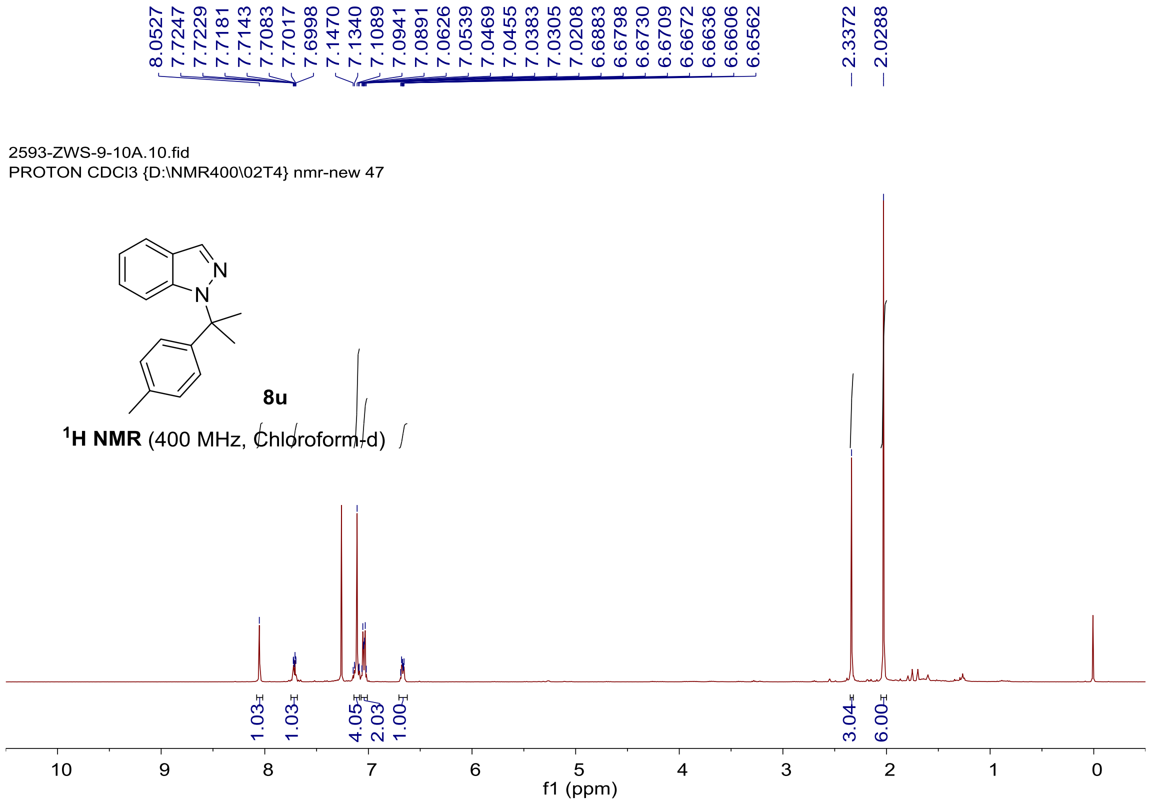
8.0527
7.7247
7.7229
7.7181
7.7143
7.7083
7.7017
7.6998
7.1470
7.1340
7.1089
7.0941
7.0891
7.0626
7.0539
7.0469
7.0455
7.0383
7.0305
7.0208
6.6883
6.6798
6.6730
6.6709
6.6672
6.6636
6.6606
6.6562
2.3372
2.0288
2593-ZWS-9-10A.10.fid
PROTON CDCl3 {D:\NMR400\02T4} nmr-new 47
N
N
8u
1H NMR (400 MHz, Chloroform-d)
1.03
1.03
4.05
2.03
1.00
3.04
6.00
10
9
8
7
6
5
4
3
2
1
0
f1 (ppm)

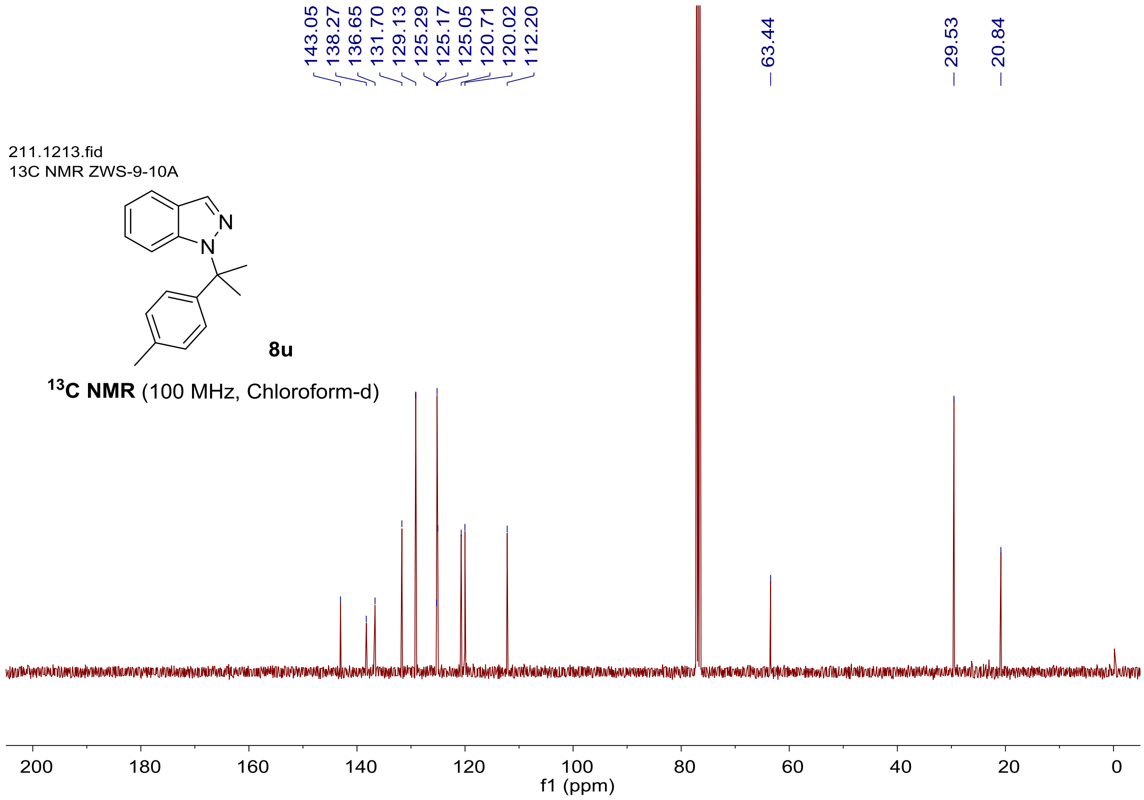
143.05
138.27
136.65
131.70
129.13
125.29
125.17
125.05
120.71
120.02
112.20
63.44
29.53
20.84
211.1213.fid
13C NMR ZWS-9-10A
N
N
8u
13C NMR (100 MHz, Chloroform-d)
200
180
160
140
120
100
80
60
40
20
0
f1 (ppm)

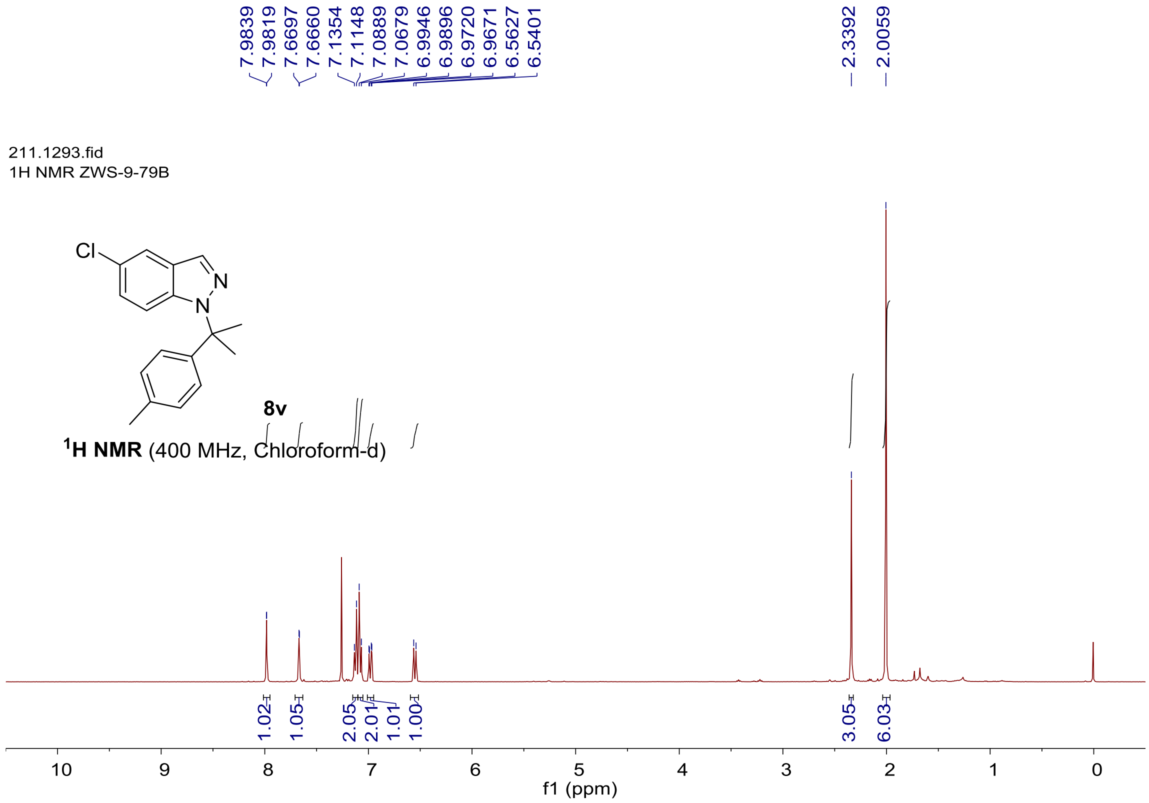
7.9839
7.9819
7.6697
7.6660
7.1354
7.1148
7.0889
7.0679
6.9946
6.9896
6.9720
6.9671
6.5627
6.5401
2.3392
2.0059
211.1293.fid
1H NMR ZWS-9-79B
Cl
N
N
8v
1H NMR (400 MHz, Chloroform-d)
1.02
1.05
2.05
2.01
1.01
1.00
3.05
6.03
10
9
8
7
6
5
4
3
2
1
0
f1 (ppm)

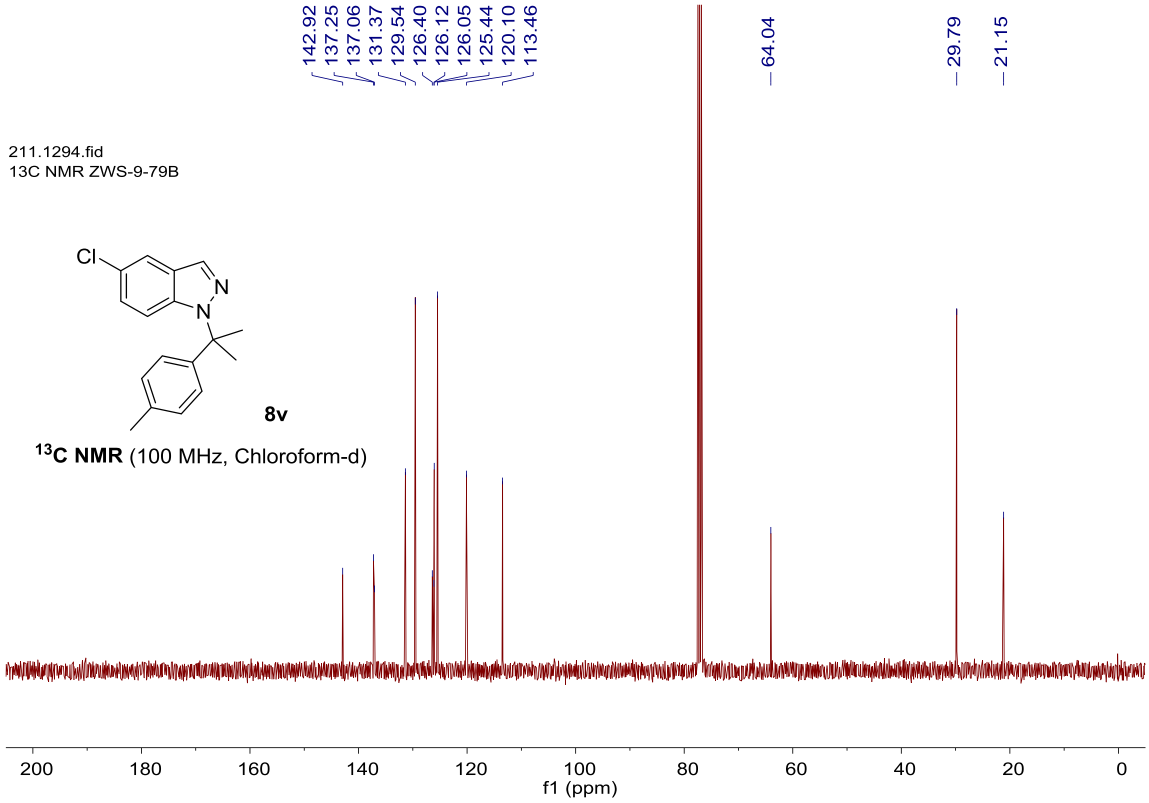
142.92
137.25
137.06
131.37
129.54
126.40
126.12
126.05
125.44
120.10
113.46
64.04
29.79
21.15
211.1294.fid
13C NMR ZWS-9-79B
Cl
N
N
8v
13C NMR (100 MHz, Chloroform-d)
200
180
160
140
120
100
80
60
40
20
0
f1 (ppm)

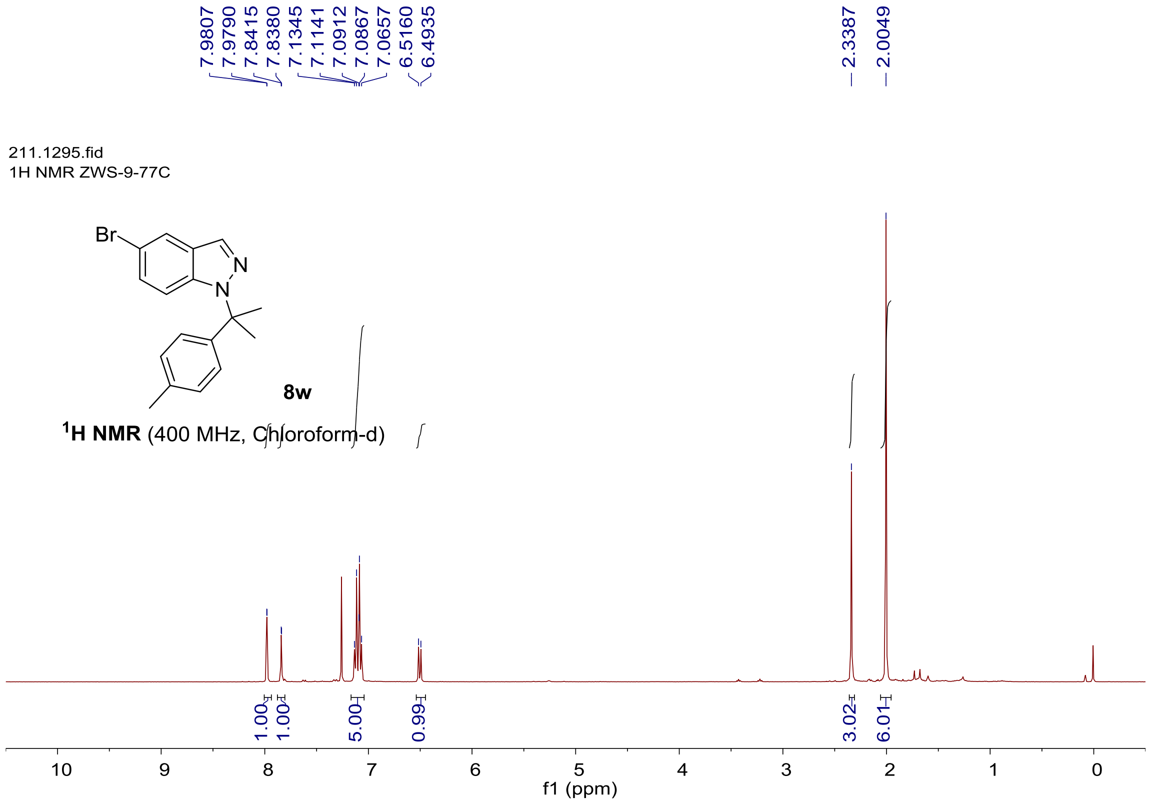
7.9807
7.9790
7.8415
7.8380
7.1345
7.1141
7.0912
7.0867
7.0657
6.5160
6.4935
2.3387
2.0049
211.1295.fid
1H NMR ZWS-9-77C
Br
N
N
8w
1H NMR (400 MHz, Chloroform-d)
1.00
1.00
5.00
0.99
3.02
6.01
10
9
8
7
6
5
4
3
2
1
0
f1 (ppm)

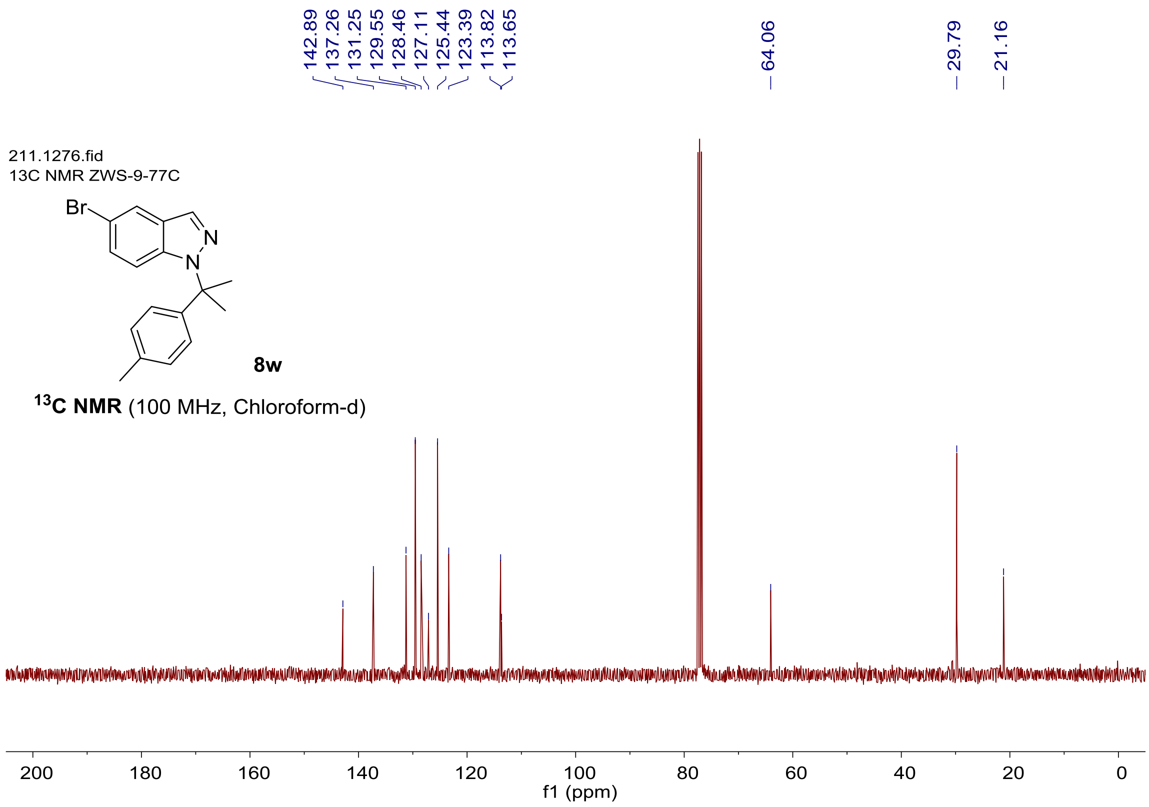
142.89
137.26
131.25
129.55
128.46
127.11
125.44
123.39
113.82
113.65
64.06
29.79
21.16
211.1276.fid
13C NMR ZWS-9-77C
Br
N
N
8w
13C NMR (100 MHz, Chloroform-d)
200
180
160
140
120
100
80
60
40
20
0
f1 (ppm)

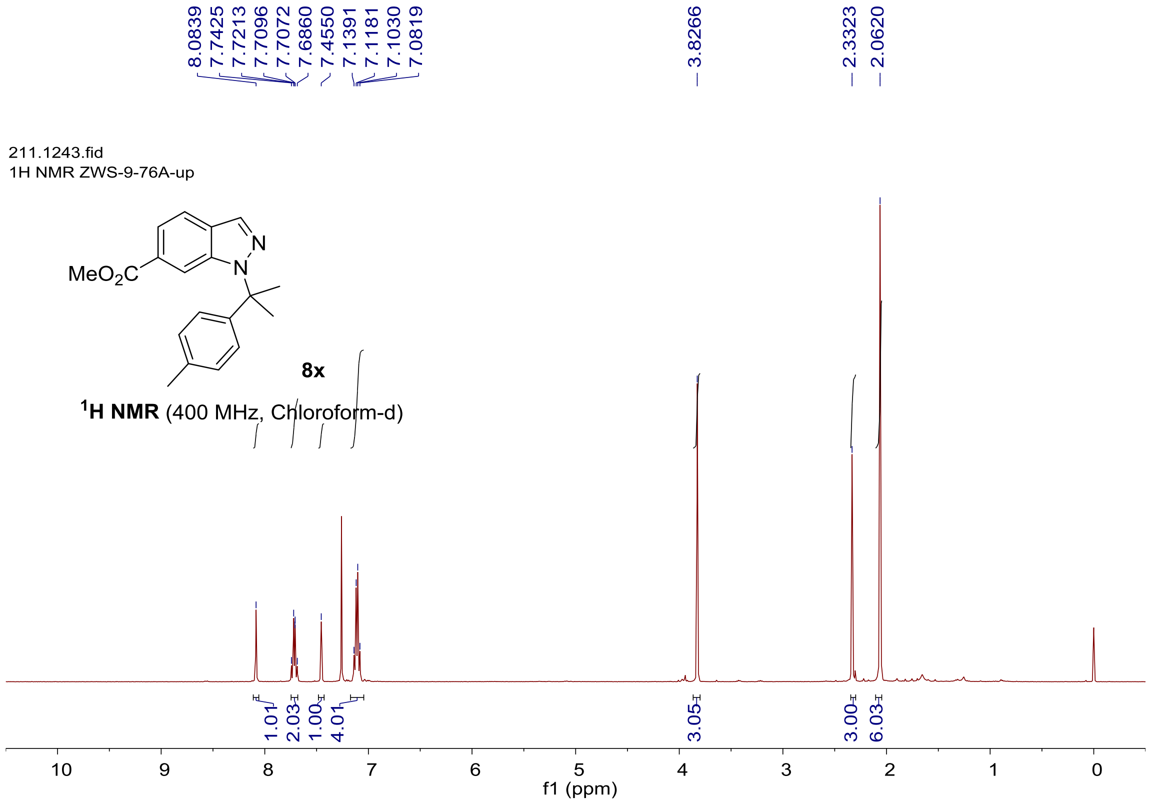

211.1243.fid
1H NMR ZWS-9-76A-up
MeO2C
N
N
8x
1H NMR (400 MHz, Chloroform-d)
8.0839
7.7425
7.7213
7.7096
7.7072
7.6860
7.4550
7.1391
7.1181
7.1030
7.0819
3.8266
2.3323
2.0620
1.01
2.03
1.00
4.01
3.05
3.00
6.03
f1 (ppm)

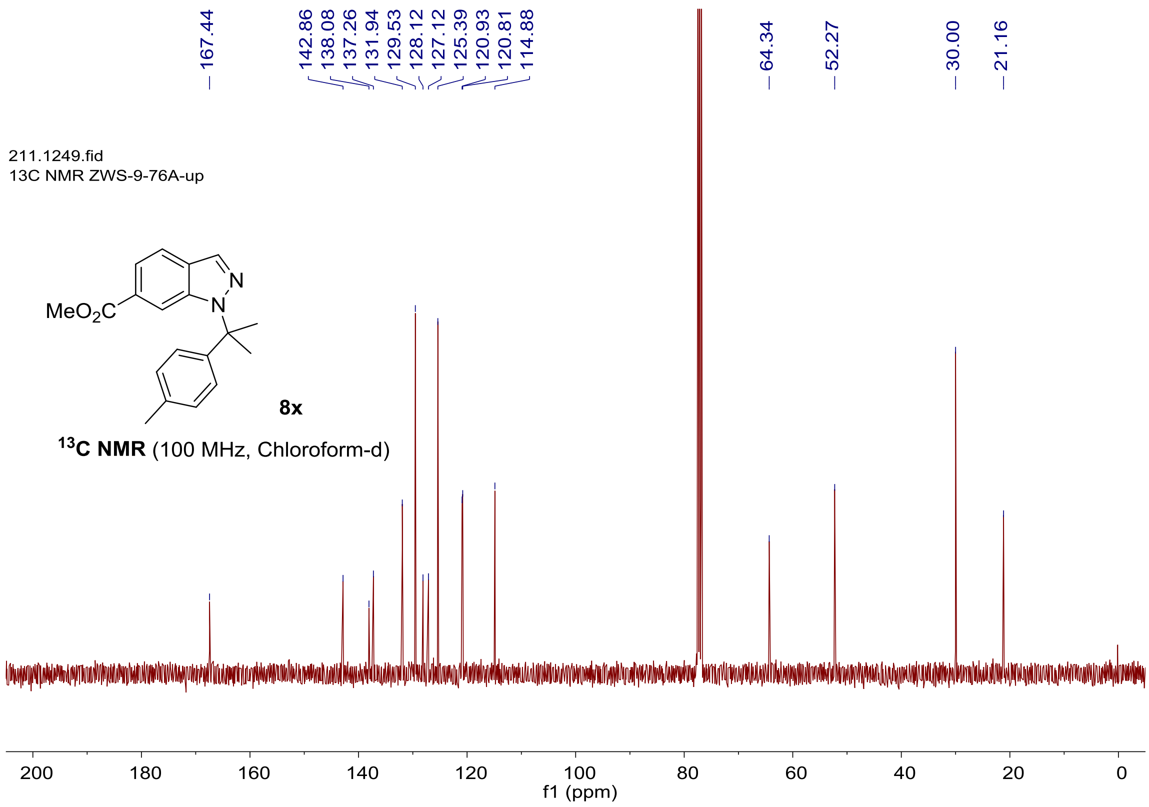

211.1249.fid
13C NMR ZWS-9-76A-up
MeO2C
N
N
8x
13C NMR (100 MHz, Chloroform-d)
167.44
142.86
138.08
137.26
131.94
129.53
128.12
127.12
125.39
120.93
120.81
114.88
64.34
52.27
30.00
21.16
f1 (ppm)

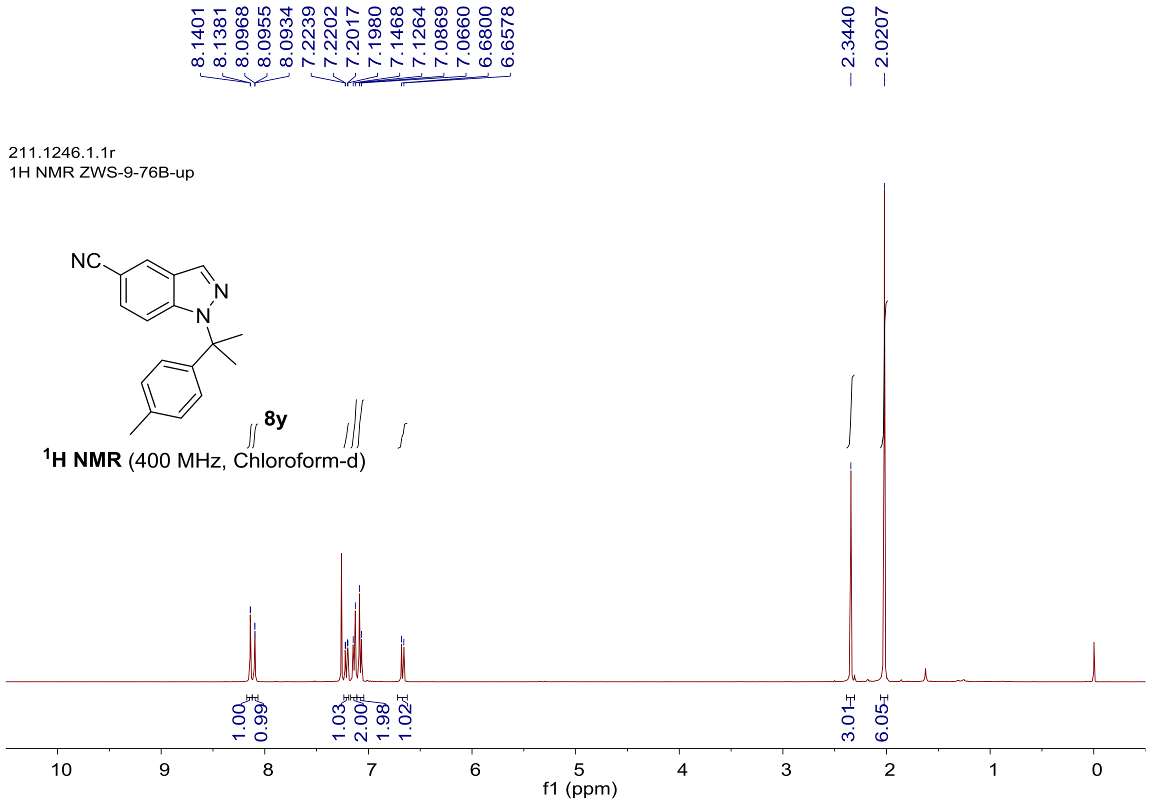
8.1401
8.1381
8.0968
8.0955
8.0934
7.2239
7.2202
7.2017
7.1980
7.1468
7.1264
7.0869
7.0660
6.6800
6.6578
2.3440
2.0207
211.1246.1.1r
1H NMR ZWS-9-76B-up
NC
N
N
8y
1H NMR (400 MHz, Chloroform-d)
1.00
0.99
1.03
2.00
1.98
1.02
3.01
6.05
10
9
8
7
6
5
4
3
2
1
0
f1 (ppm)

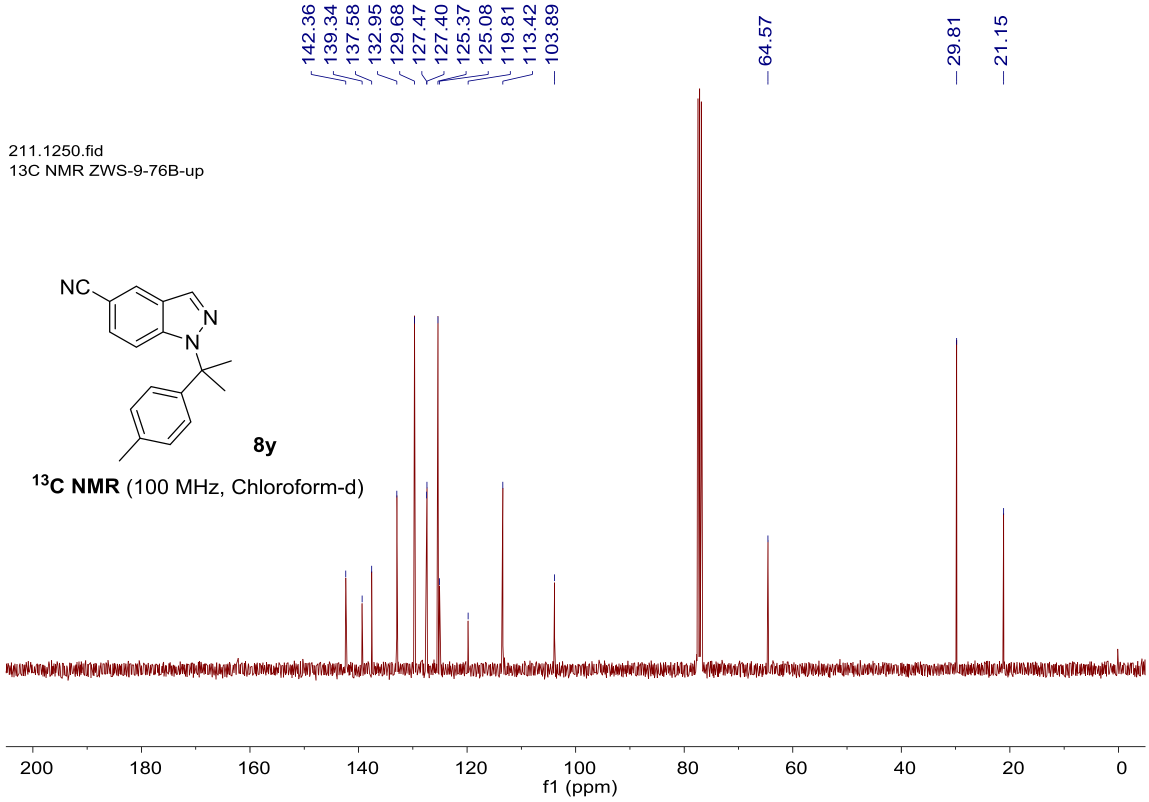
142.36
139.34
137.58
132.95
129.68
127.47
127.40
125.37
125.08
119.81
113.42
103.89
64.57
29.81
21.15
211.1250.fid
13C NMR ZWS-9-76B-up
NC
N
N
8y
13C NMR (100 MHz, Chloroform-d)
200
180
160
140
120
100
80
60
40
20
0
f1 (ppm)

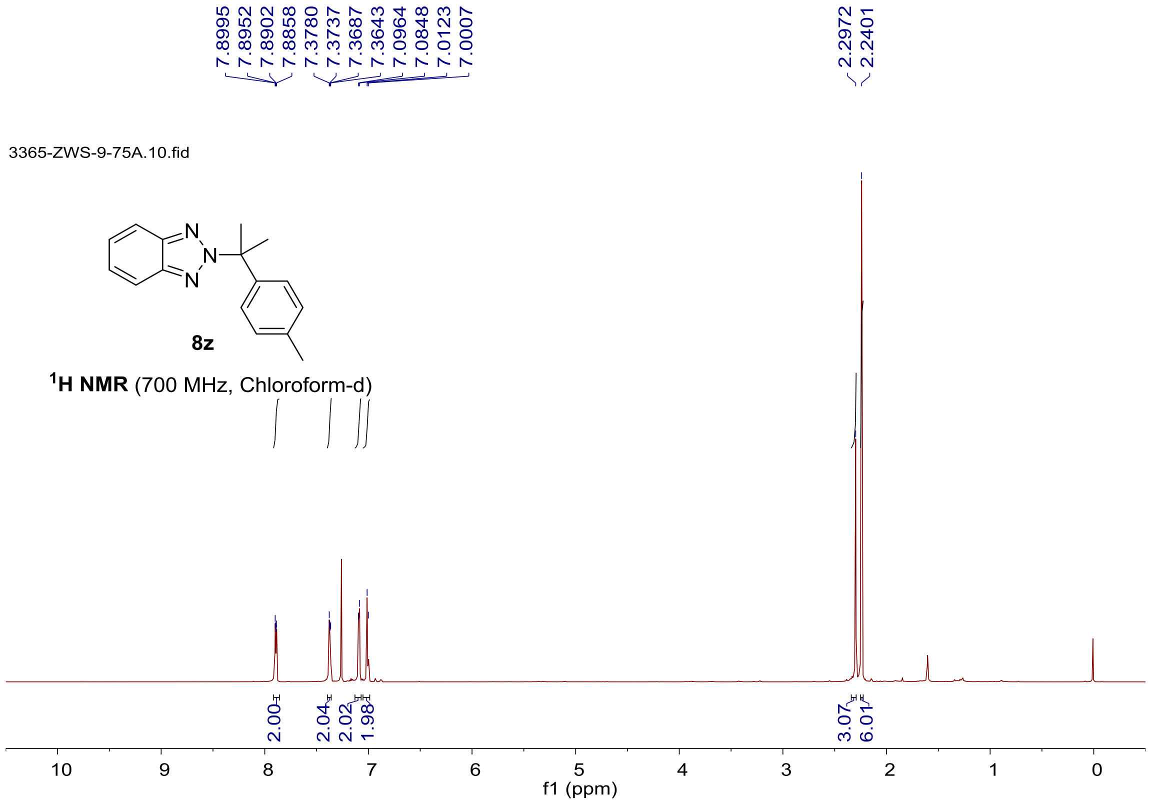
7.8995
7.8952
7.8902
7.8858
7.3780
7.3737
7.3687
7.3643
7.0964
7.0848
7.0123
7.0007
2.2972
2.2401
3365-ZWS-9-75A.10.fid
8z
1H NMR (700 MHz, Chloroform-d)
2.00
2.04
2.02
1.98
3.07
6.01
f1 (ppm)

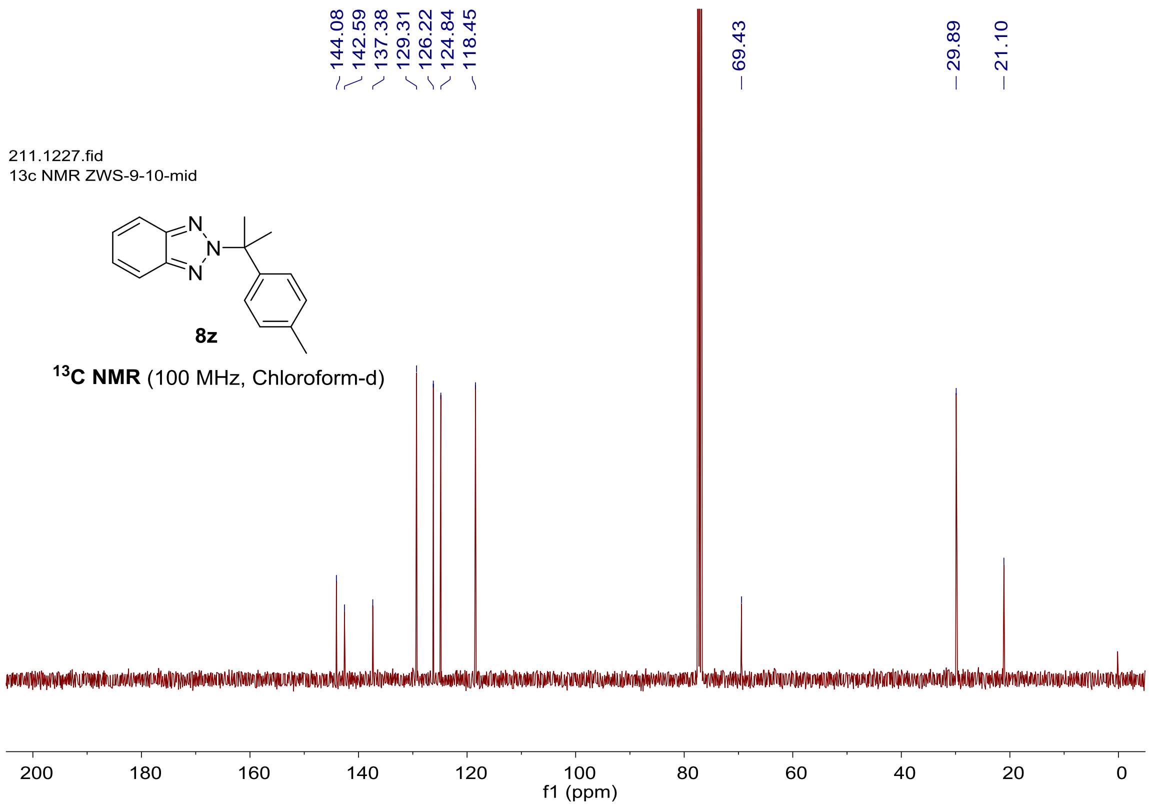
144.08
142.59
137.38
129.31
126.22
124.84
118.45
69.43
29.89
21.10
211.1227.fid
13c NMR ZWS-9-10-mid
8z
13C NMR (100 MHz, Chloroform-d)
f1 (ppm)

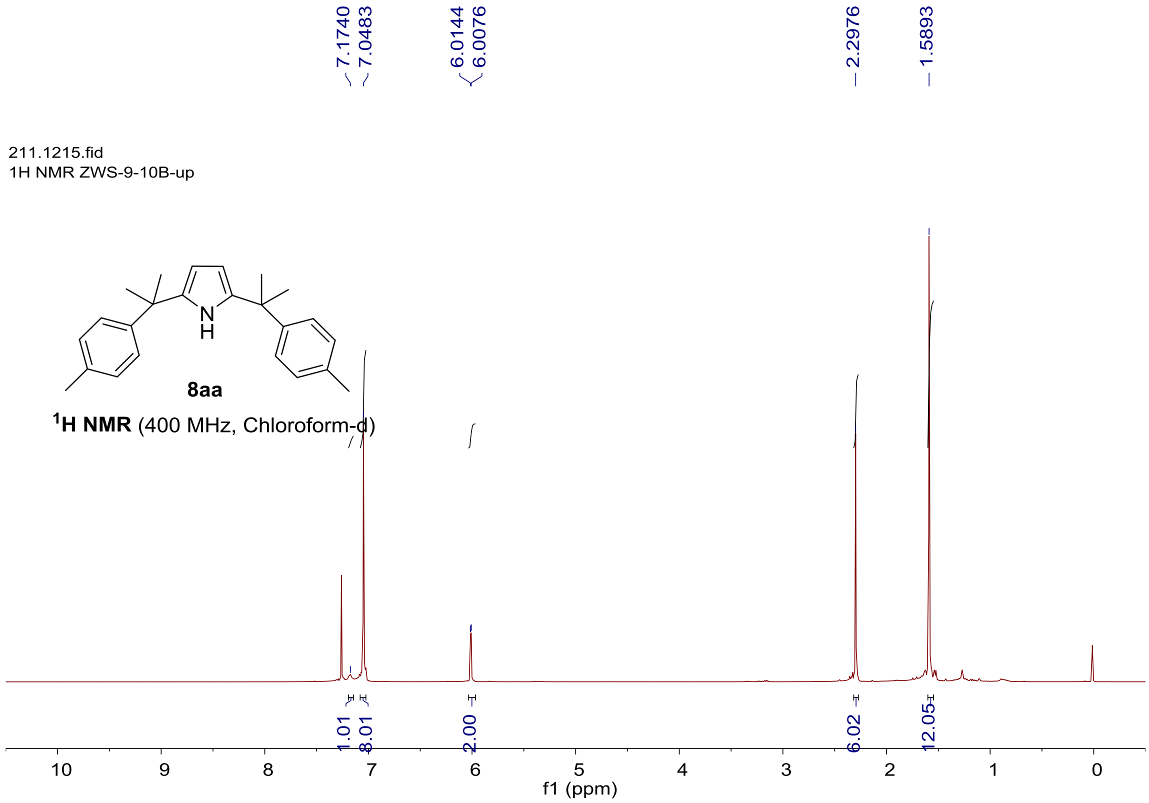

7.1740
7.0483
6.0144
6.0076
2.2976
1.5893
211.1215.fid
1H NMR ZWS-9-10B-up
8aa
1H NMR (400 MHz, Chloroform-d)
1.01
8.01
2.00
6.02
12.05
10
9
8
7
6
5
4
3
2
1
0
f1 (ppm)

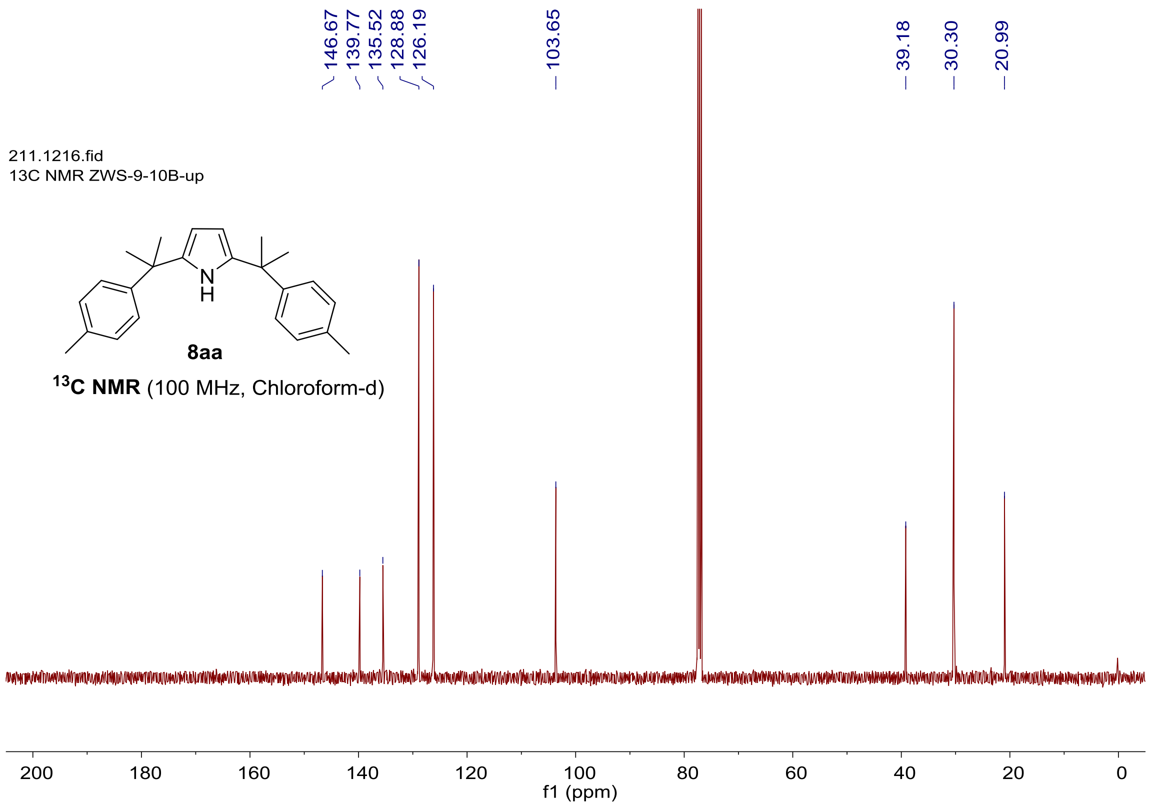

146.67
139.77
135.52
128.88
126.19
103.65
39.18
30.30
20.99
211.1216.fid
13C NMR ZWS-9-10B-up
8aa
13C NMR (100 MHz, Chloroform-d)
200
180
160
140
120
100
80
60
40
20
0
f1 (ppm)

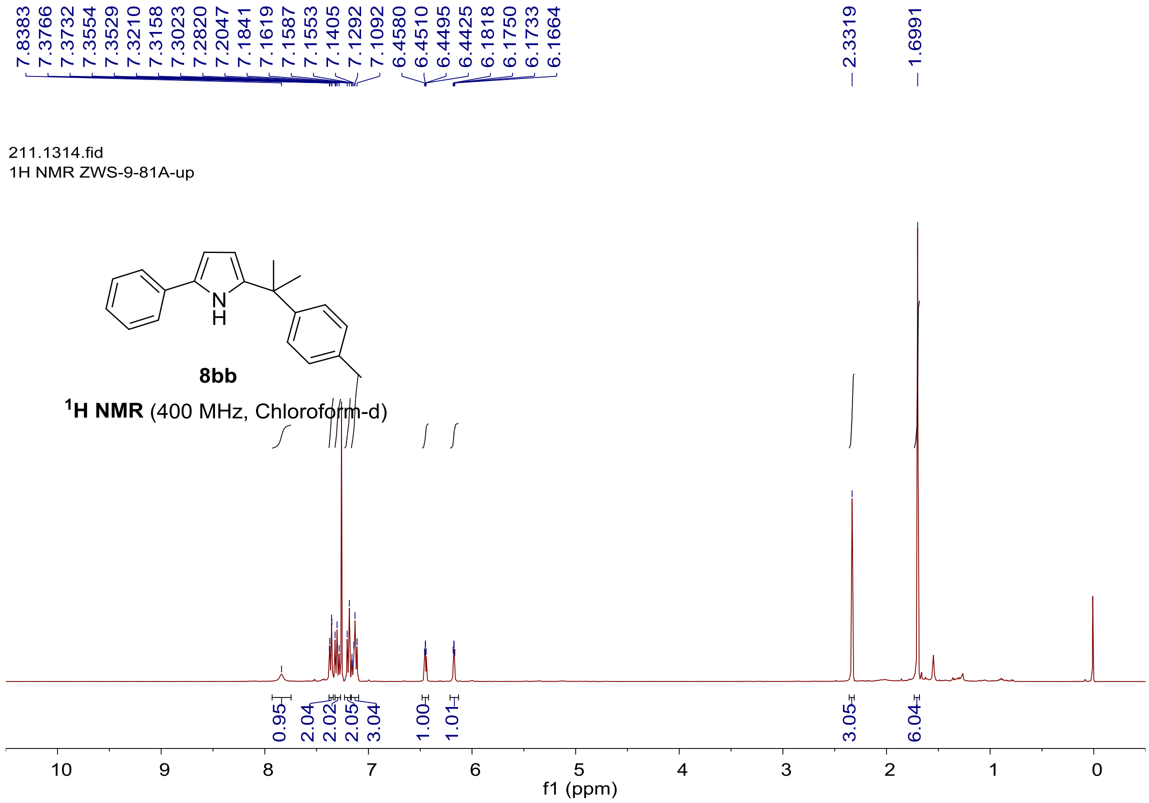
7.8383
7.3766
7.3732
7.3554
7.3529
7.3210
7.3158
7.3023
7.2820
7.2047
7.1841
7.1619
7.1587
7.1553
7.1405
7.1292
7.1092
6.4580
6.4510
6.4495
6.4425
6.1818
6.1750
6.1733
6.1664
2.3319
1.6991
211.1314.fid
1H NMR ZWS-9-81A-up
N
H
8bb
1H NMR (400 MHz, Chloroform-d)
0.95
2.04
2.02
2.05
3.04
1.00
1.01
3.05
6.04
10
9
8
7
6
5
4
3
2
1
0
f1 (ppm)

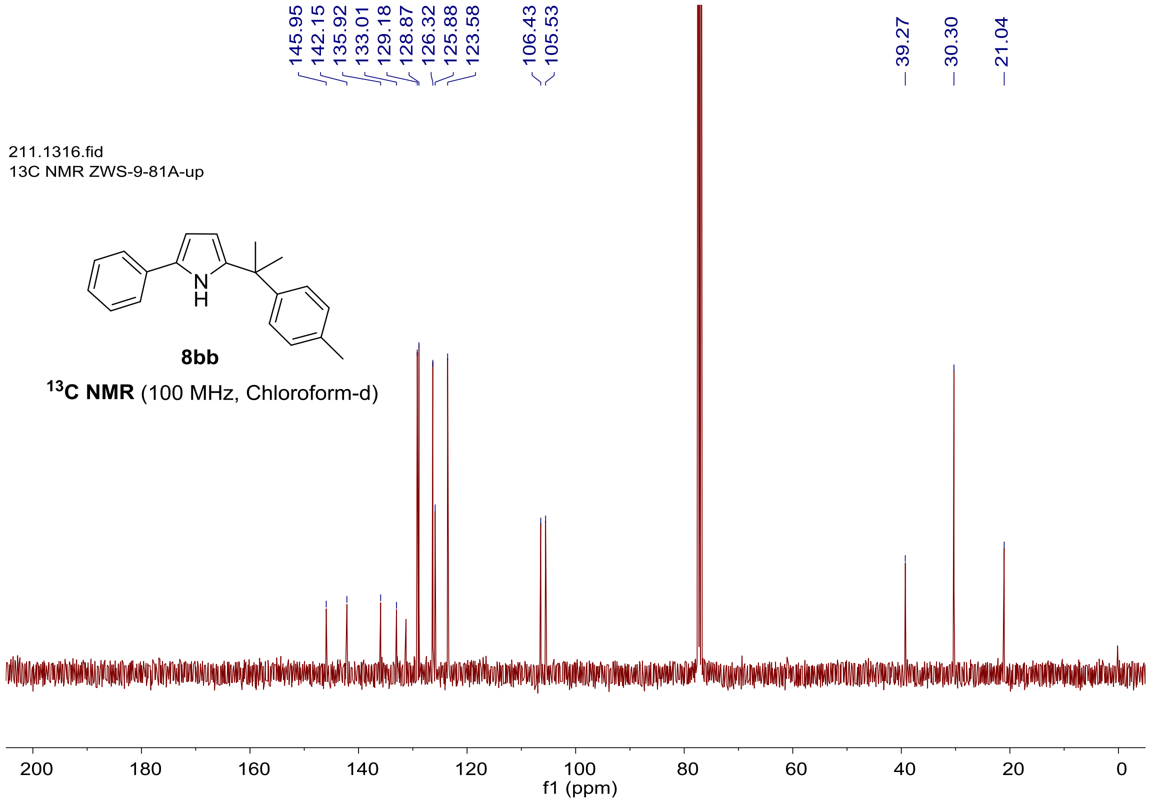
145.95
142.15
135.92
133.01
129.18
128.87
126.32
125.88
123.58
106.43
105.53
39.27
30.30
21.04
211.1316.fid
13C NMR ZWS-9-81A-up
N
H
8bb
13C NMR (100 MHz, Chloroform-d)
200
180
160
140
120
100
80
60
40
20
0
f1 (ppm)

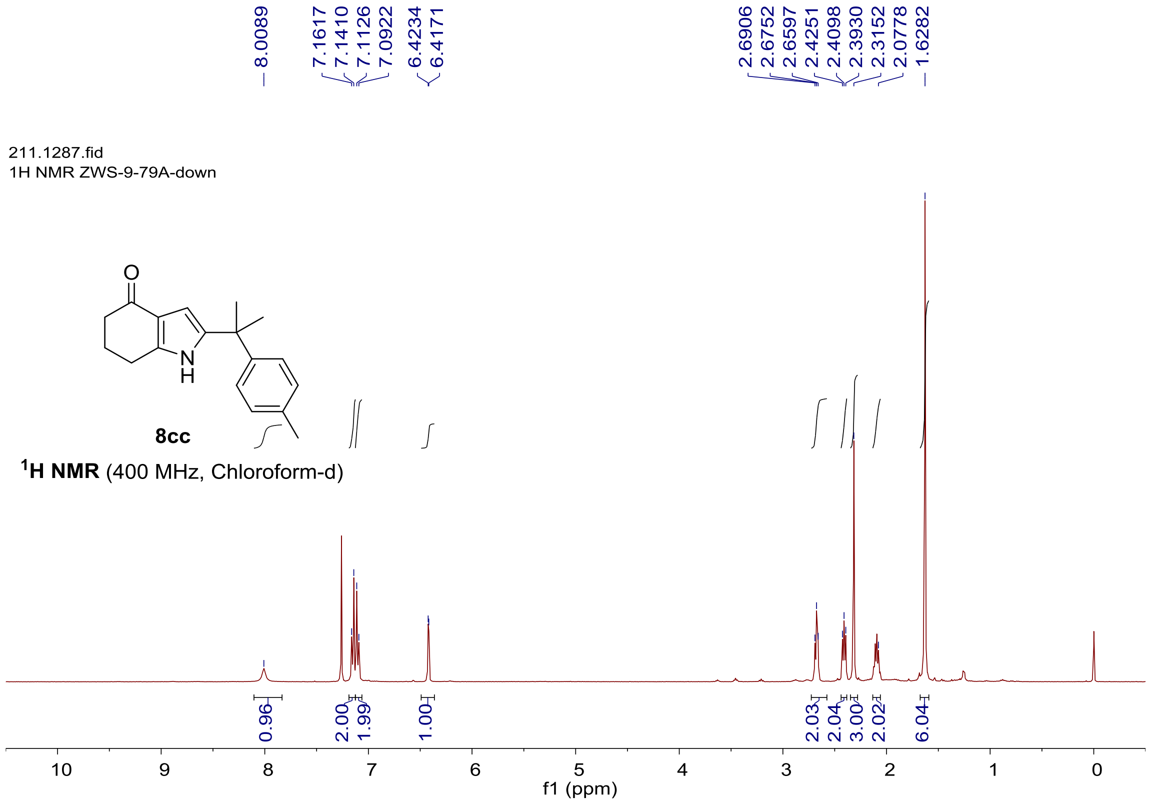
8.0089
7.1617
7.1410
7.1126
7.0922
6.4234
6.4171
2.6906
2.6752
2.6597
2.4251
2.4098
2.3930
2.3152
2.0778
1.6282
211.1287.fid
1H NMR ZWS-9-79A-down
O
N
H
8cc
1H NMR (400 MHz, Chloroform-d)
0.96
2.00
1.99
1.00
2.03
2.04
3.00
2.02
6.04
f1 (ppm)

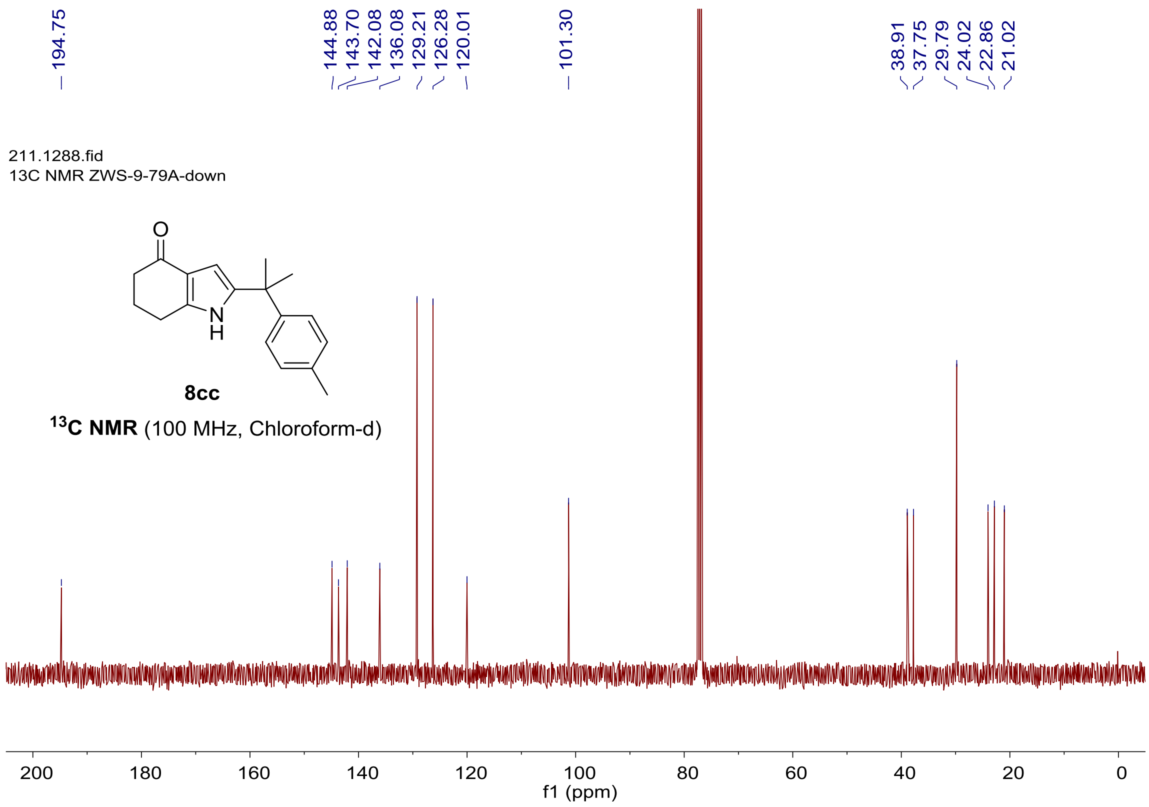
194.75
144.88
143.70
142.08
136.08
129.21
126.28
120.01
101.30
38.91
37.75
29.79
24.02
22.86
21.02
211.1288.fid
13C NMR ZWS-9-79A-down
O
N
H
8cc
13C NMR (100 MHz, Chloroform-d)
f1 (ppm)

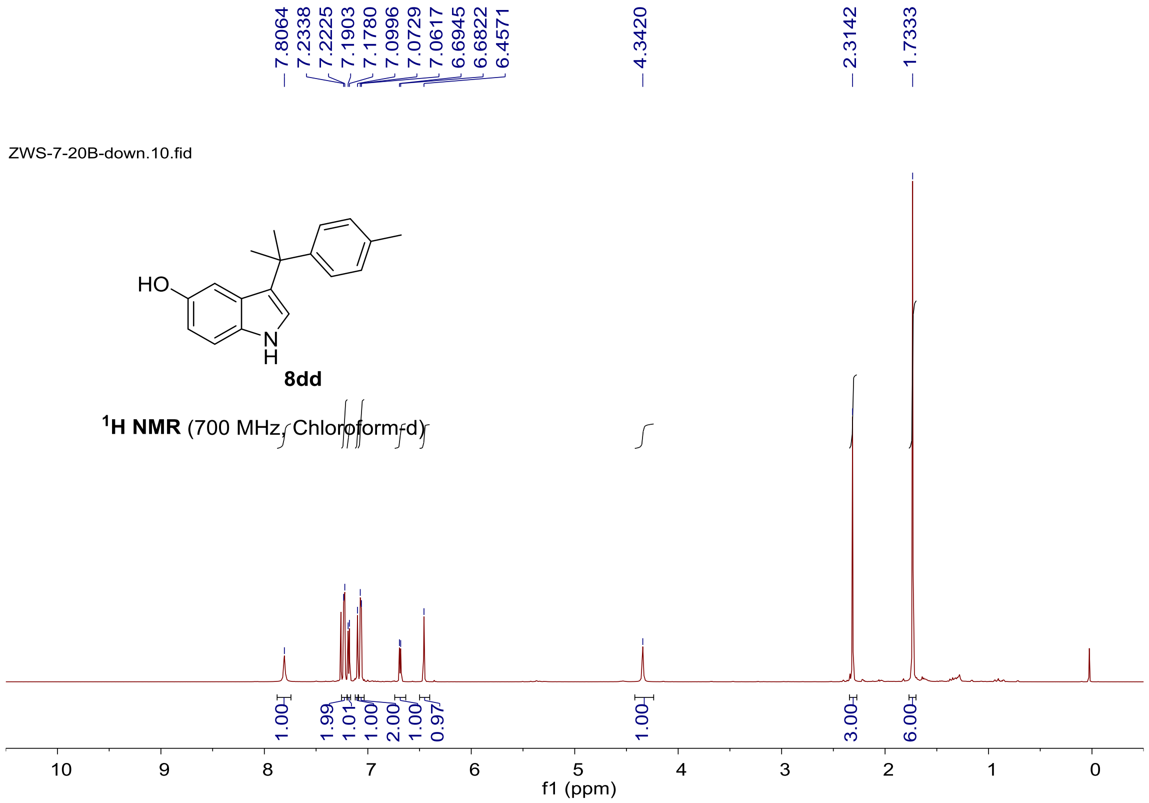
7.8064
7.2338
7.2225
7.1903
7.1780
7.0996
7.0729
7.0617
6.6945
6.6822
6.4571
4.3420
2.3142
1.7333
ZWS-7-20B-down.10.fid
HO
N
H
8dd
1H NMR (700 MHz, Chloroform-d)
1.00
1.99
1.01
1.00
2.00
1.00
0.97
1.00
3.00
6.00
10
9
8
7
6
5
4
3
2
1
0
f1 (ppm)

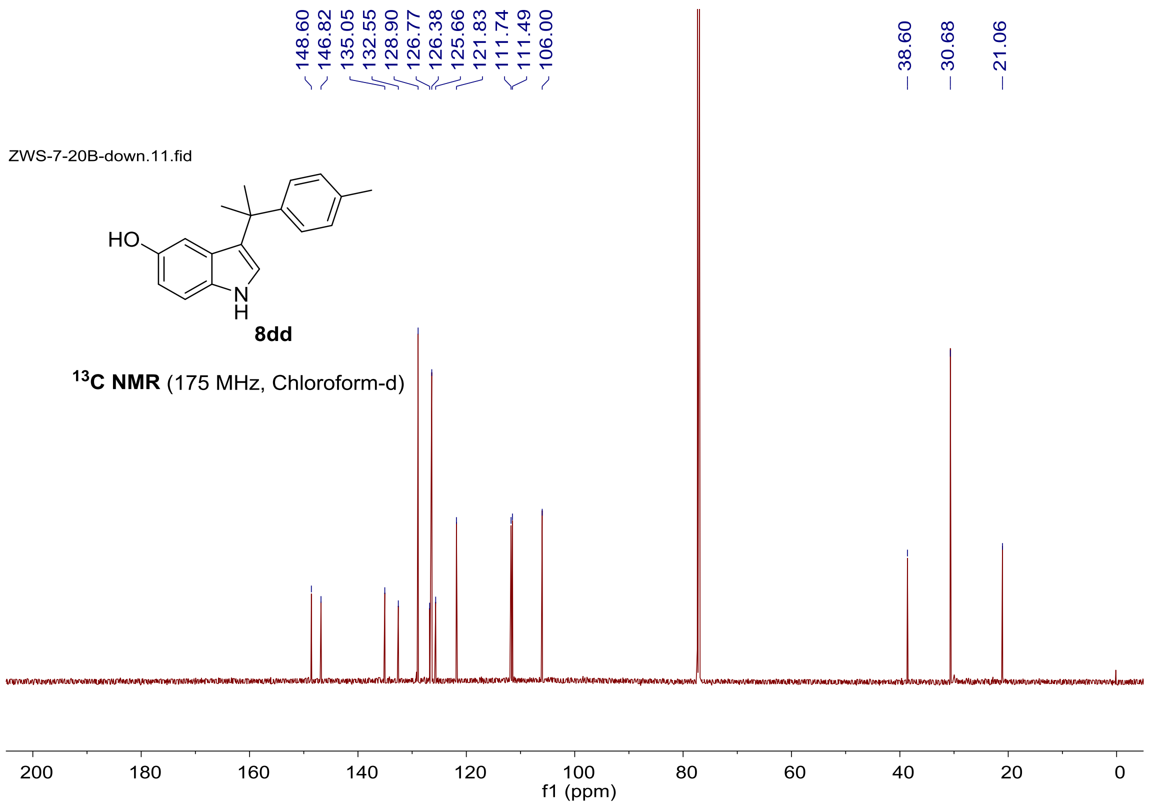
148.60
146.82
135.05
132.55
128.90
126.77
126.38
125.66
121.83
111.74
111.49
106.00
38.60
30.68
21.06
ZWS-7-20B-down.11.fid
HO
N
H
8dd
13C NMR (175 MHz, Chloroform-d)
200
180
160
140
120
100
80
60
40
20
0
f1 (ppm)

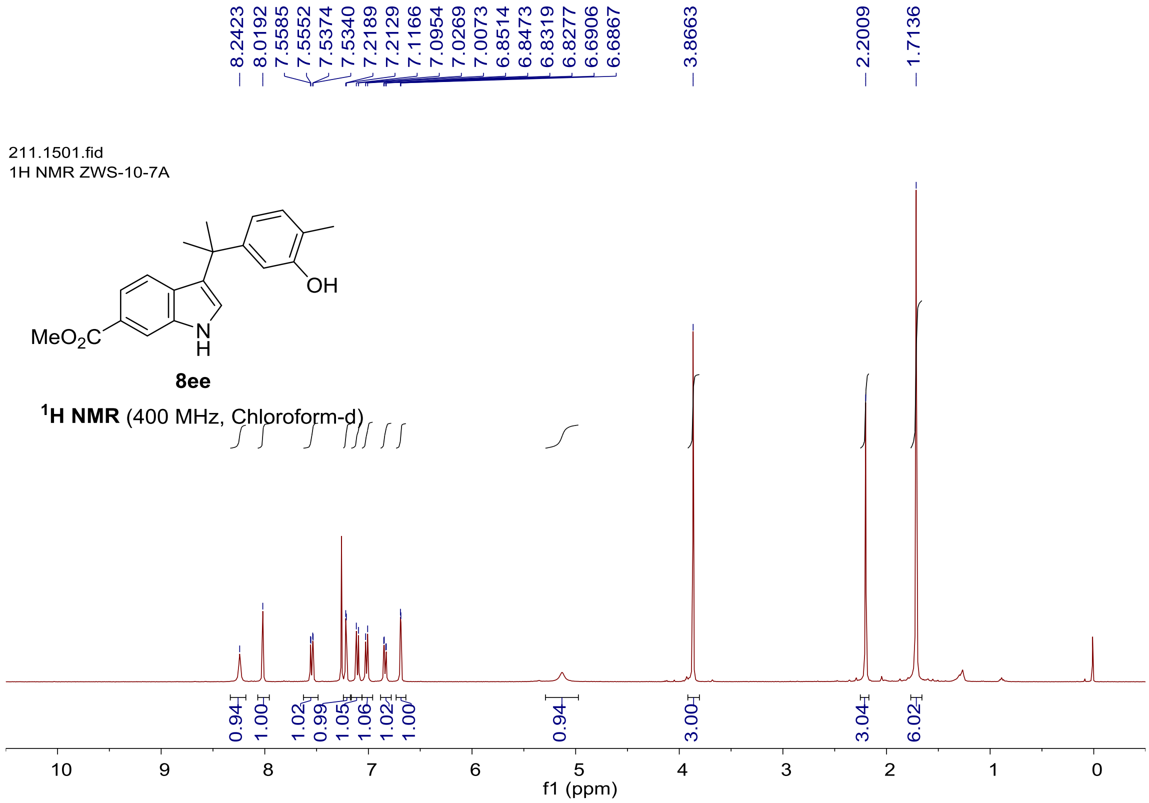
8.2423
8.0192
7.5585
7.5552
7.5374
7.5340
7.2189
7.2129
7.1166
7.0954
7.0269
7.0073
6.8514
6.8473
6.8319
6.8277
6.6906
6.6867
3.8663
2.2009
1.7136
211.1501.fid
1H NMR ZWS-10-7A
OH
MeO2C
N
H
8ee
1H NMR (400 MHz, Chloroform-d)
0.94
1.00
1.02
0.99
1.05
1.06
1.02
1.00
0.94
3.00
3.04
6.02
10
9
8
7
6
5
4
3
2
1
0
f1 (ppm)

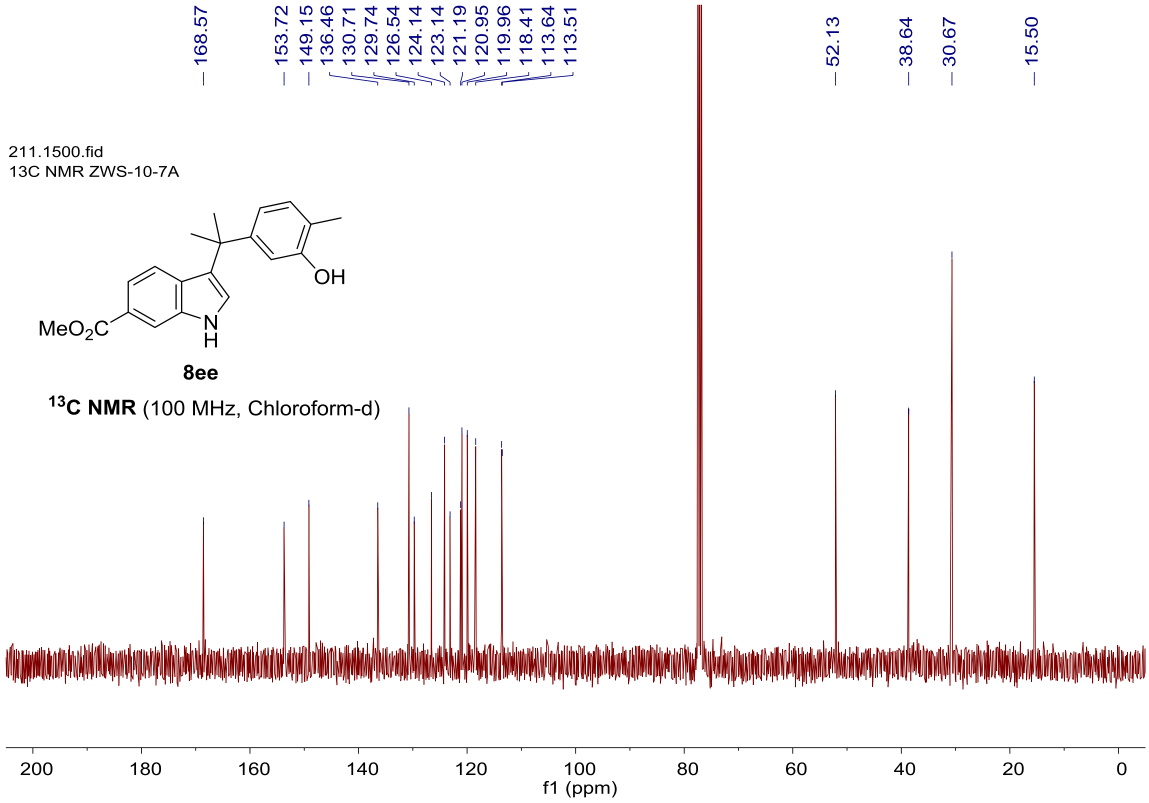
168.57
153.72
149.15
136.46
130.71
129.74
126.54
124.14
123.14
121.19
120.95
119.96
118.41
113.64
113.51
52.13
38.64
30.67
15.50
211.1500.fid
13C NMR ZWS-10-7A
OH
MeO2C
N
H
8ee
13C NMR (100 MHz, Chloroform-d)
200
180
160
140
120
100
80
60
40
20
0
f1 (ppm)

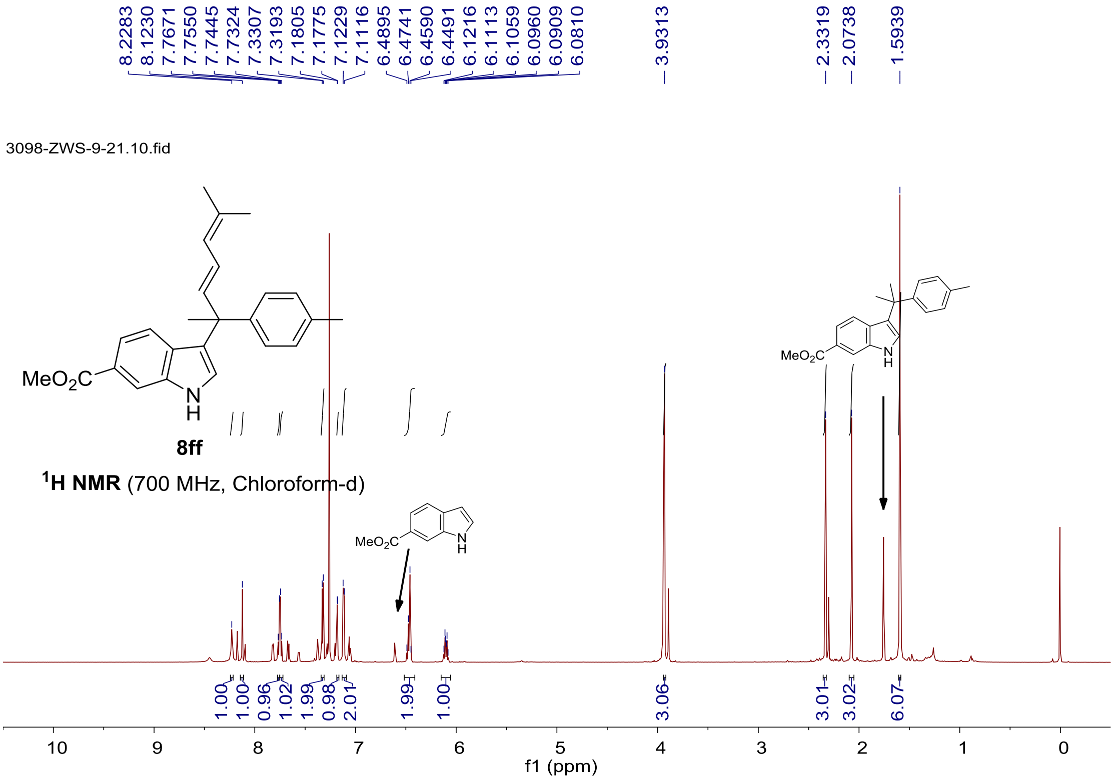
8.2283
8.1230
7.7671
7.7550
7.7445
7.7324
7.3307
7.3193
7.1805
7.1775
7.1229
7.1116
6.4895
6.4741
6.4590
6.4491
6.1216
6.1113
6.1059
6.0960
6.0909
6.0810
3.9313
2.3319
2.0738
1.5939
3098-ZWS-9-21.10.fid
MeO2C
N
H
8ff
1H NMR (700 MHz, Chloroform-d)
1.00
1.00
0.96
1.02
1.99
0.98
2.01
1.99
1.00
3.06
3.01
3.02
6.07
10
9
8
7
6
5
4
3
2
1
0
f1 (ppm)

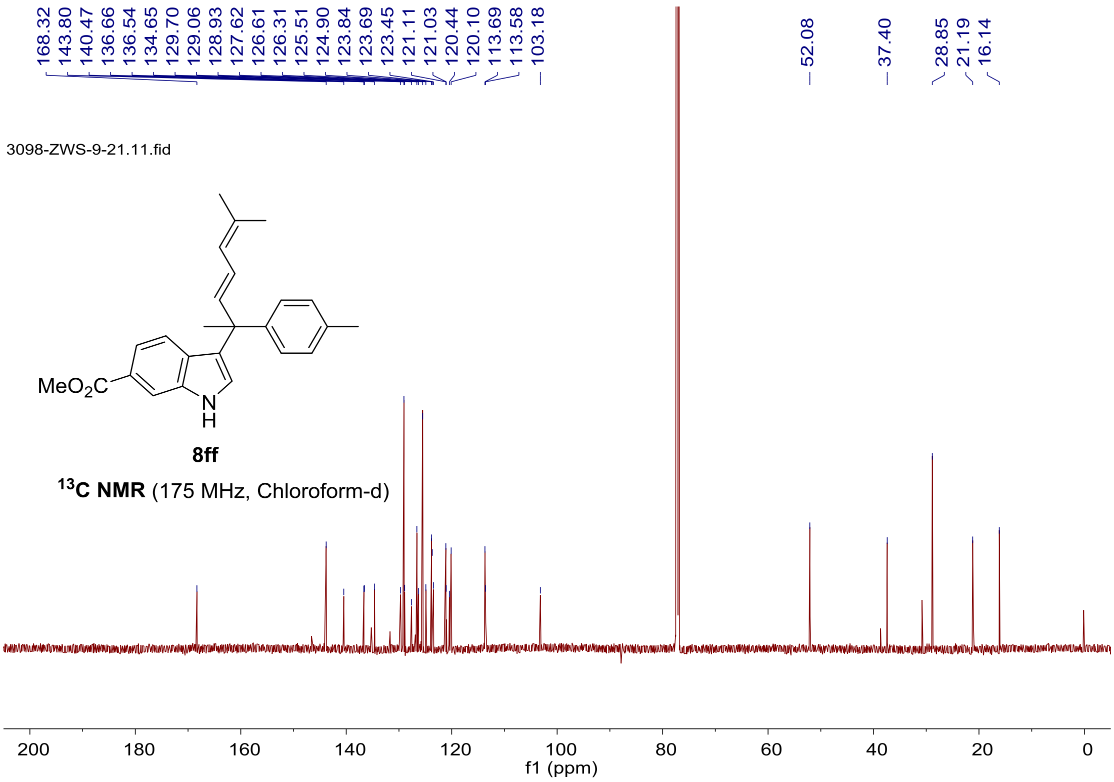
168.32
143.80
140.47
136.66
136.54
134.65
129.70
129.06
128.93
127.62
126.61
126.31
125.51
124.90
123.84
123.69
123.45
121.11
121.03
120.44
120.10
113.69
113.58
103.18
52.08
37.40
28.85
21.19
16.14
3098-ZWS-9-21.11.fid
MeO2C
N
H
8ff
13C NMR (175 MHz, Chloroform-d)
200
180
160
140
120
100
80
60
40
20
0
f1 (ppm)

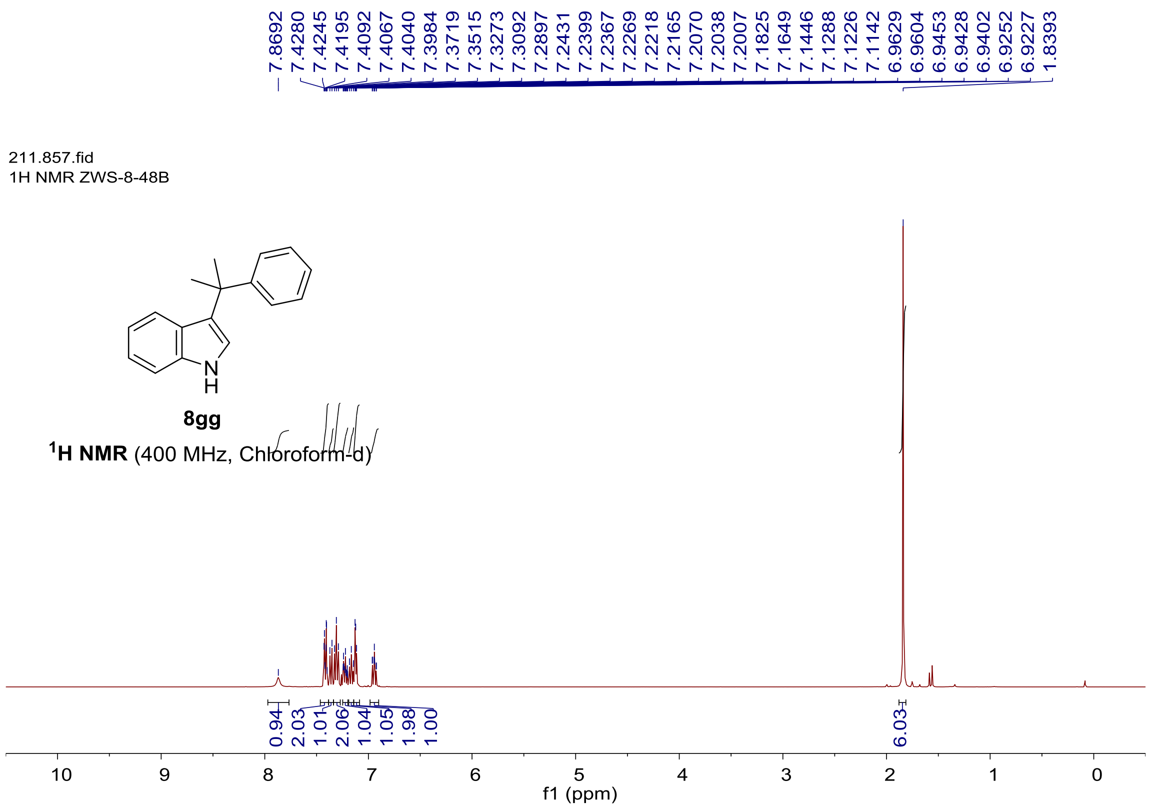

7.8692
7.4280
7.4245
7.4195
7.4092
7.4067
7.4040
7.3984
7.3719
7.3515
7.3273
7.3092
7.2897
7.2431
7.2399
7.2367
7.2269
7.2218
7.2165
7.2070
7.2038
7.2007
7.1825
7.1649
7.1446
7.1288
7.1226
7.1142
6.9629
6.9604
6.9453
6.9428
6.9402
6.9252
6.9227
1.8393
211.857.fid
1H NMR ZWS-8-48B
8gg
1H NMR (400 MHz, Chloroform-d)
0.94
2.03
1.01
2.06
1.04
1.05
1.98
1.00
6.03
f1 (ppm)

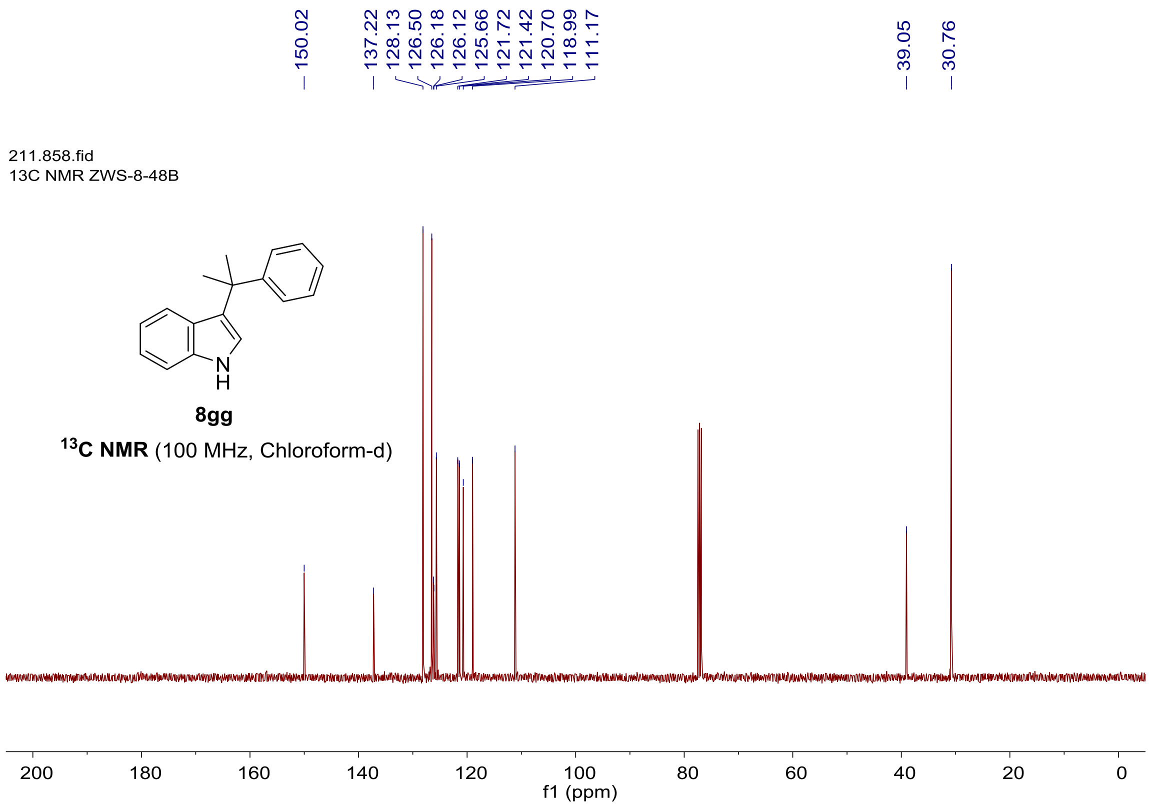

150.02
137.22
128.13
126.50
126.18
126.12
125.66
121.72
121.42
120.70
118.99
111.17
39.05
30.76
211.858.fid
13C NMR ZWS-8-48B
8gg
13C NMR (100 MHz, Chloroform-d)
f1 (ppm)

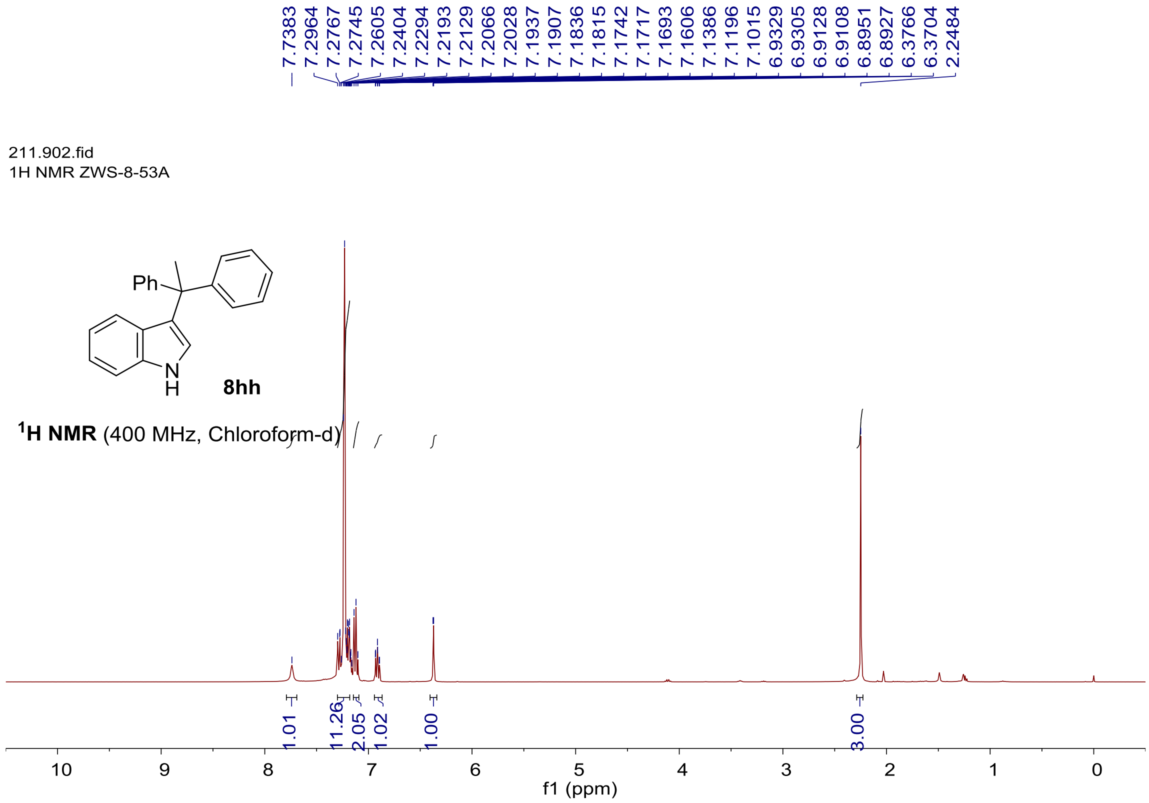
7.7383
7.2964
7.2767
7.2745
7.2605
7.2404
7.2294
7.2193
7.2129
7.2066
7.2028
7.1937
7.1907
7.1836
7.1815
7.1742
7.1717
7.1693
7.1606
7.1386
7.1196
7.1015
6.9329
6.9305
6.9128
6.9108
6.8951
6.8927
6.3766
6.3704
2.2484
211.902.fid
1H NMR ZWS-8-53A
Ph
N
H
8hh
1H NMR (400 MHz, Chloroform-d)
1.01
11.26
2.05
1.02
1.00
3.00
f1 (ppm)

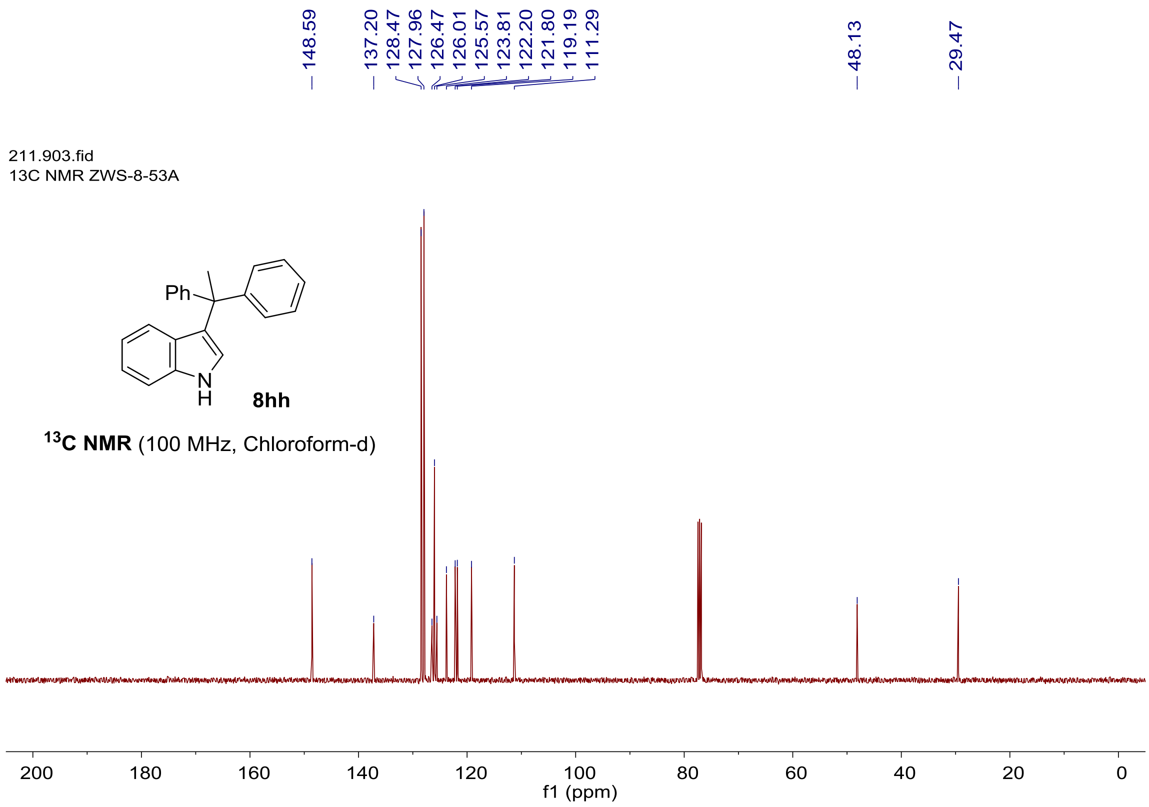
148.59
137.20
128.47
127.96
126.47
126.01
125.57
123.81
122.20
121.80
119.19
111.29
48.13
29.47
211.903.fid
13C NMR ZWS-8-53A
Ph
N
H
8hh
13C NMR (100 MHz, Chloroform-d)
f1 (ppm)

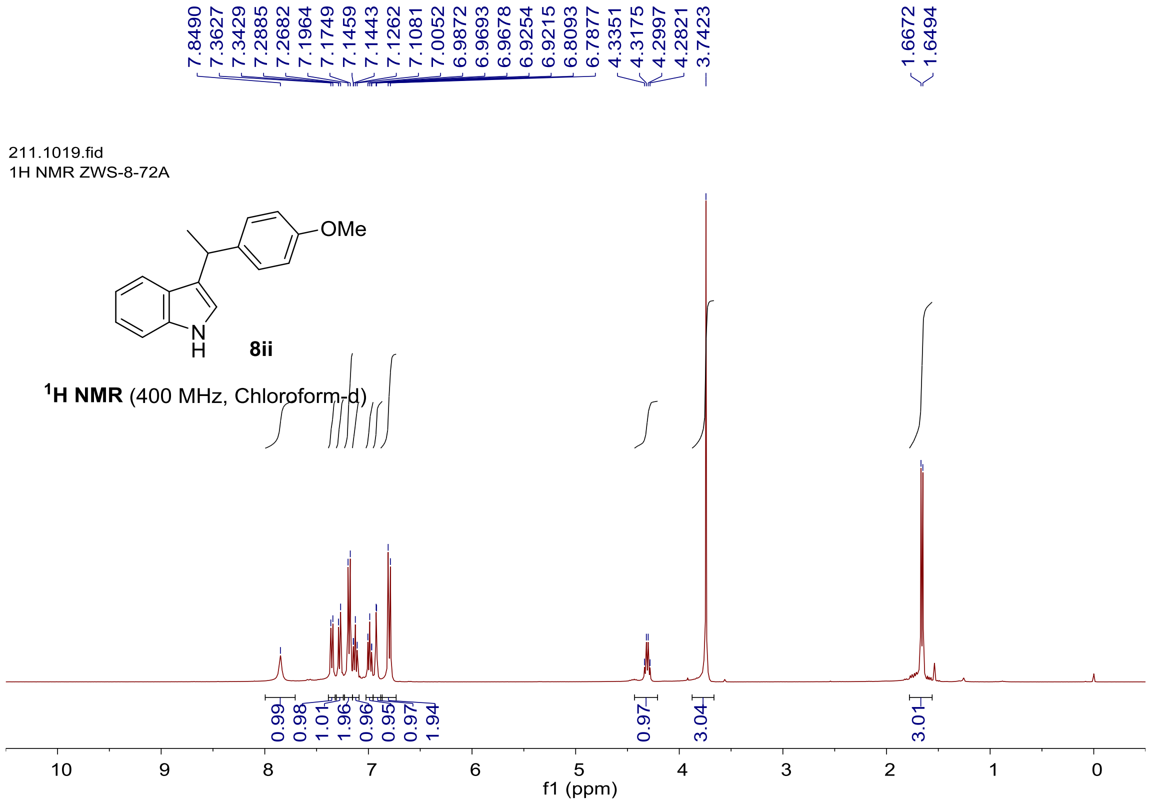
7.8490
7.3627
7.3429
7.2885
7.2682
7.1964
7.1749
7.1459
7.1443
7.1262
7.1081
7.0052
6.9872
6.9693
6.9678
6.9254
6.9215
6.8093
6.7877
4.3351
4.3175
4.2997
4.2821
3.7423
1.6672
1.6494
211.1019.fid
1H NMR ZWS-8-72A
OMe
N
H
8ii
1H NMR (400 MHz, Chloroform-d)
0.99
0.98
1.01
1.96
0.96
0.95
0.97
1.94
0.97
3.04
3.01
f1 (ppm)

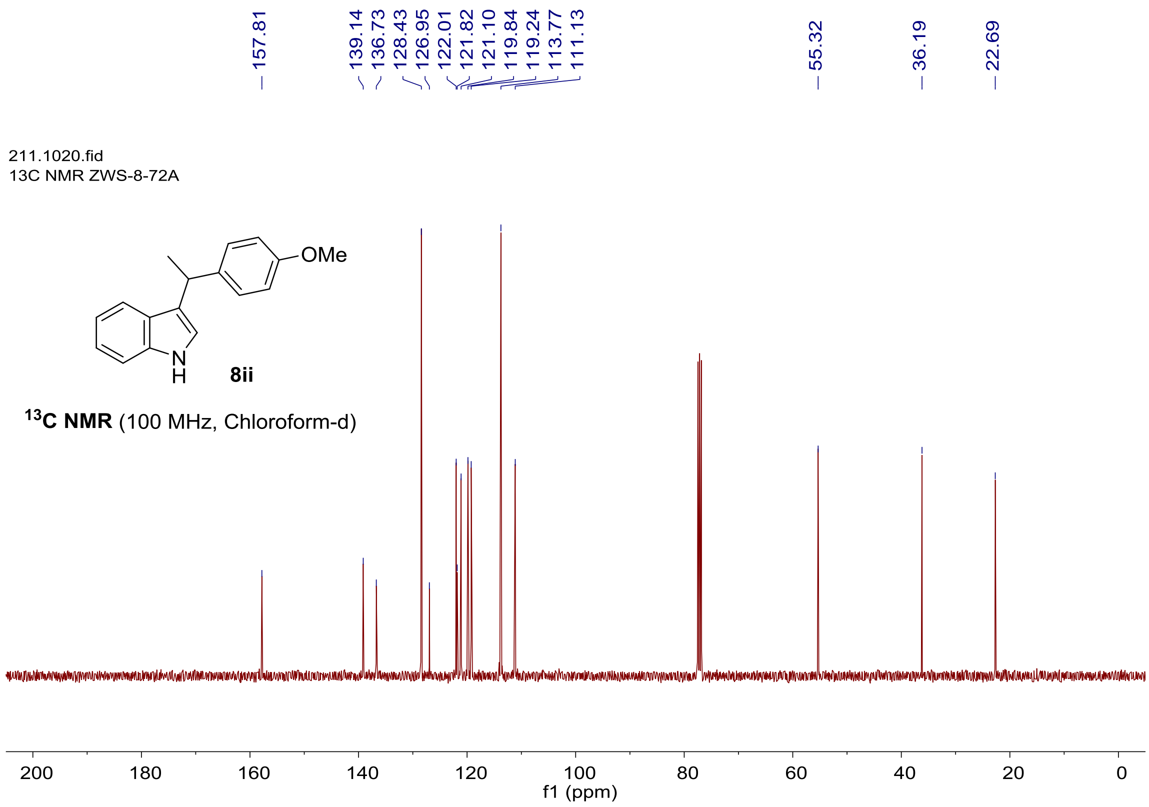
157.81
139.14
136.73
128.43
126.95
122.01
121.82
121.10
119.84
119.24
113.77
111.13
55.32
36.19
22.69
211.1020.fid
13C NMR ZWS-8-72A
OMe
N
H
8ii
13C NMR (100 MHz, Chloroform-d)
f1 (ppm)

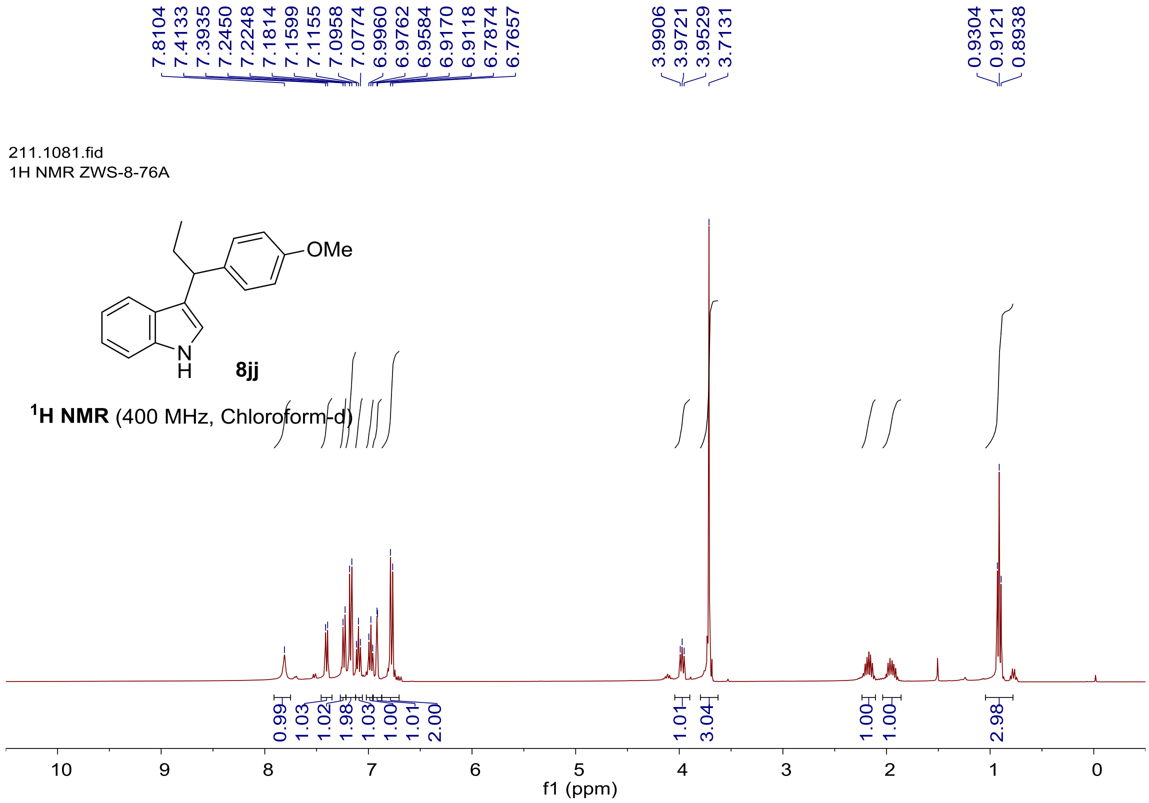
7.8104
7.4133
7.3935
7.2450
7.2248
7.1814
7.1599
7.1155
7.0958
7.0774
6.9960
6.9762
6.9584
6.9170
6.9118
6.7874
6.7657
3.9906
3.9721
3.9529
3.7131
0.9304
0.9121
0.8938
211.1081.fid
1H NMR ZWS-8-76A
OMe
N
H
8jj
1H NMR (400 MHz, Chloroform-d)
0.99
1.03
1.02
1.98
1.03
1.00
1.01
2.00
1.01
3.04
1.00
1.00
2.98
10
9
8
7
6
5
4
3
2
1
0
f1 (ppm)

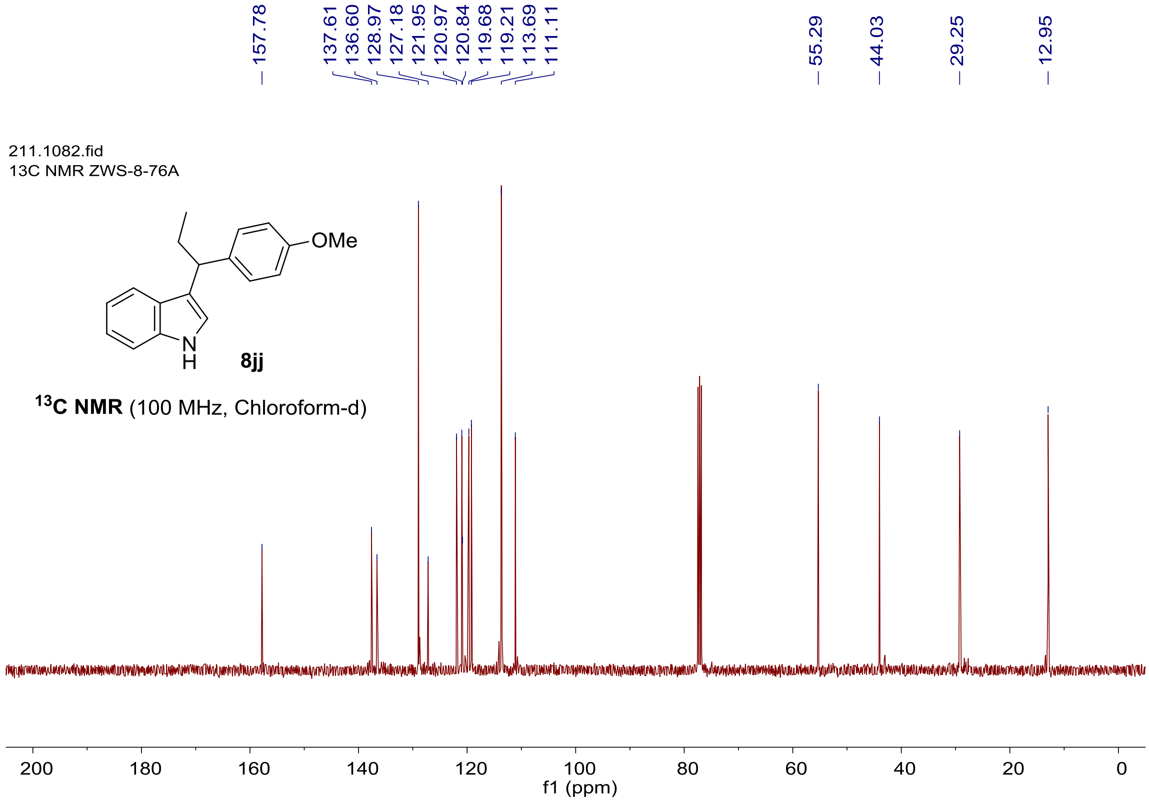
157.78
137.61
136.60
128.97
127.18
121.95
120.97
120.84
119.68
119.21
113.69
111.11
55.29
44.03
29.25
12.95
211.1082.fid
13C NMR ZWS-8-76A
OMe
N
H
8jj
13C NMR (100 MHz, Chloroform-d)
200
180
160
140
120
100
80
60
40
20
0
f1 (ppm)

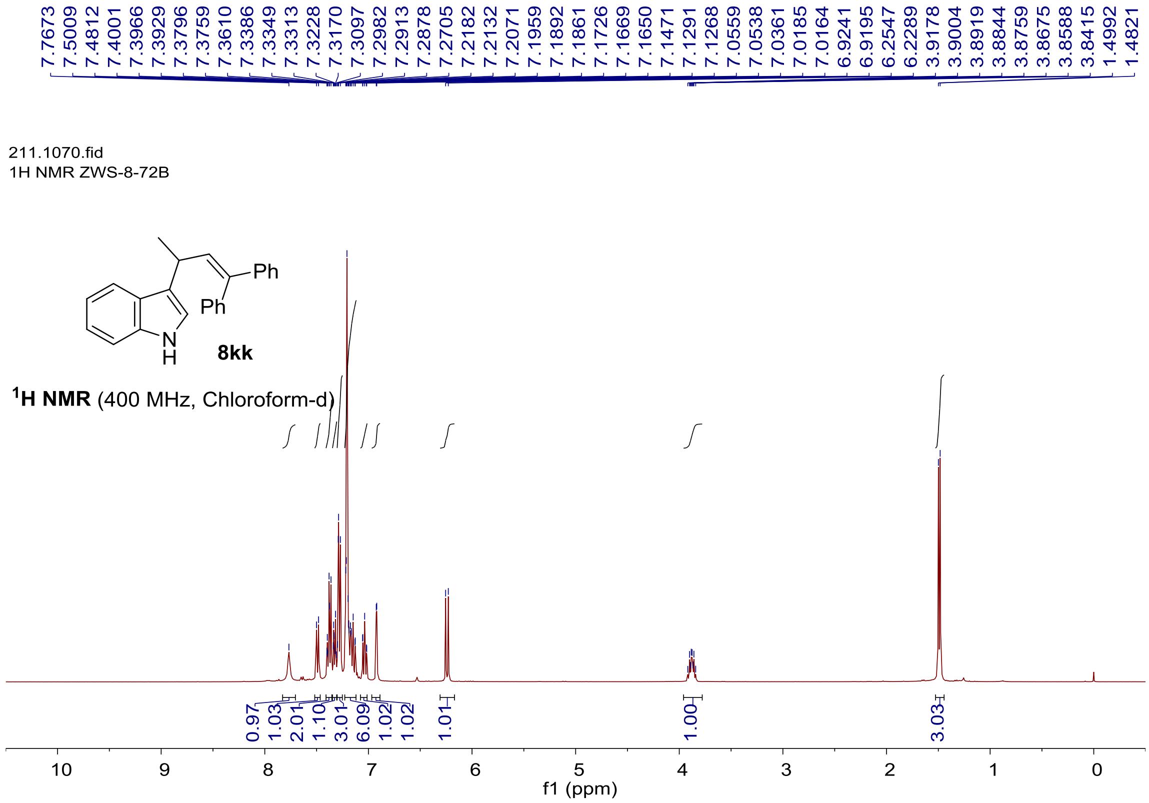
7.7673 7.5009 7.4812 7.4001 7.3966 7.3929 7.3796 7.3759 7.3610 7.3386 7.3349 7.3313 7.3228 7.3170 7.3097 7.2982 7.2913 7.2878 7.2705 7.2182 7.2132 7.2071 7.1959 7.1892 7.1861 7.1726 7.1669 7.1650 7.1471 7.1291 7.1268 7.0559 7.0538 7.0361 7.0185 7.0164 6.9241 6.9195 6.2547 6.2289 3.9178 3.9004 3.8919 3.8844 3.8759 3.8675 3.8588 3.8415 1.4992 1.4821
211.1070.fid
1H NMR ZWS-8-72B
Ph
Ph
N
H
8kk
1H NMR (400 MHz, Chloroform-d)
0.97 1.03 2.01 1.10 3.01 6.09 1.02 1.02 1.01 1.00 3.03
10 9 8 7 6 5 4 3 2 1 0
f1 (ppm)

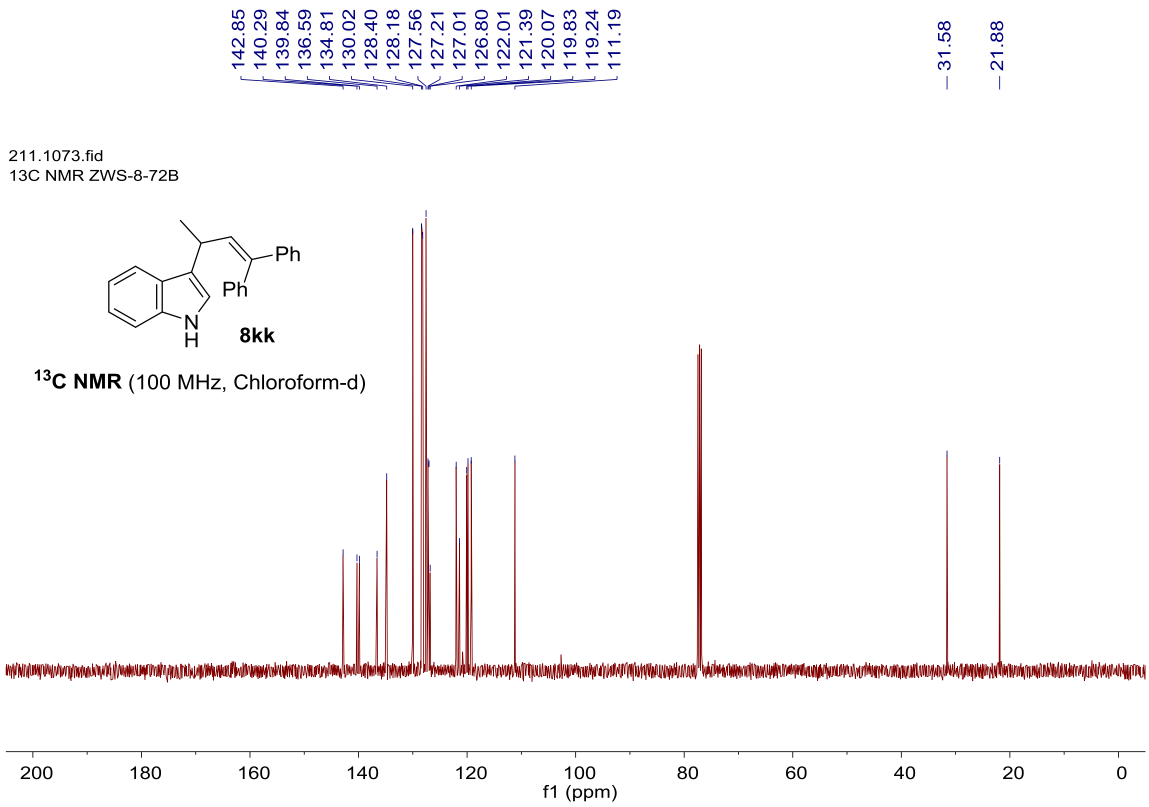
142.85 140.29 139.84 136.59 134.81 130.02 128.40 128.18 127.56 127.21 127.01 126.80 122.01 121.39 120.07 119.83 119.24 111.19
31.58
21.88
211.1073.fid
13C NMR ZWS-8-72B
Ph
Ph
N
H
8kk
13C NMR (100 MHz, Chloroform-d)
200 180 160 140 120 100 80 60 40 20 0
f1 (ppm)

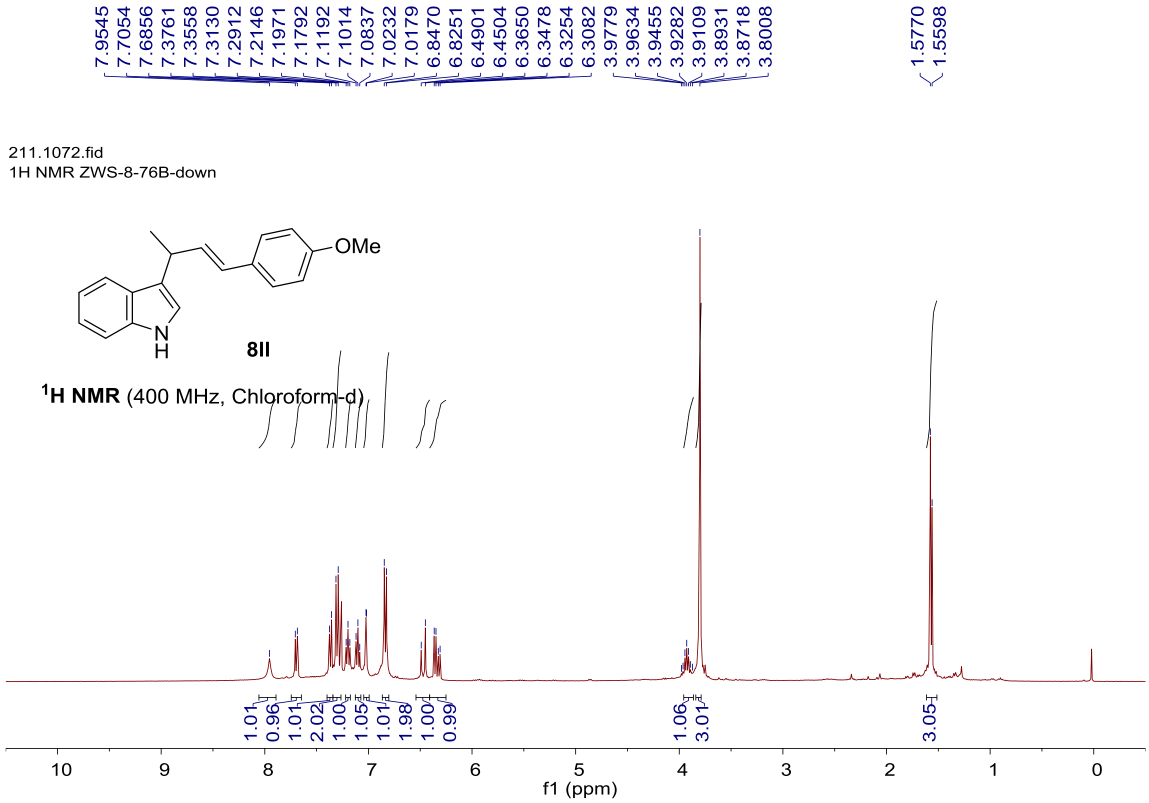
7.9545
7.7054
7.6856
7.3761
7.3558
7.3130
7.2912
7.2146
7.1971
7.1792
7.1192
7.1014
7.0837
7.0232
7.0179
6.8470
6.8251
6.4901
6.4504
6.3650
6.3478
6.3254
6.3082
3.9779
3.9634
3.9455
3.9282
3.9109
3.8931
3.8718
3.8008
1.5770
1.5598
211.1072.fid
1H NMR ZWS-8-76B-down
OMe
N
H
8ll
¹H NMR (400 MHz, Chloroform-d)
1.01
0.96
1.01
2.02
1.00
1.05
1.01
1.98
1.00
0.99
1.06
3.01
3.05
f1 (ppm)

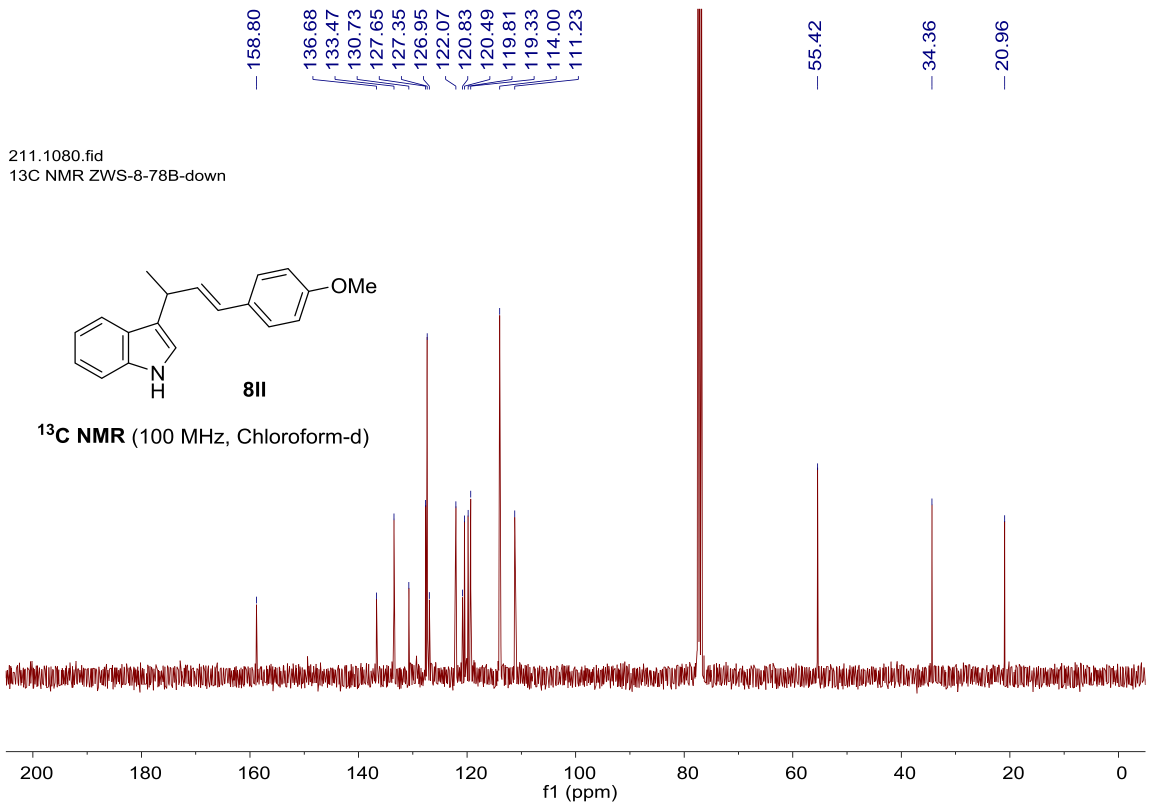
158.80
136.68
133.47
130.73
127.65
127.35
126.95
122.07
120.83
120.49
119.81
119.33
114.00
111.23
55.42
34.36
20.96
211.1080.fid
13C NMR ZWS-8-78B-down
OMe
N
H
8ll
¹³C NMR (100 MHz, Chloroform-d)
f1 (ppm)

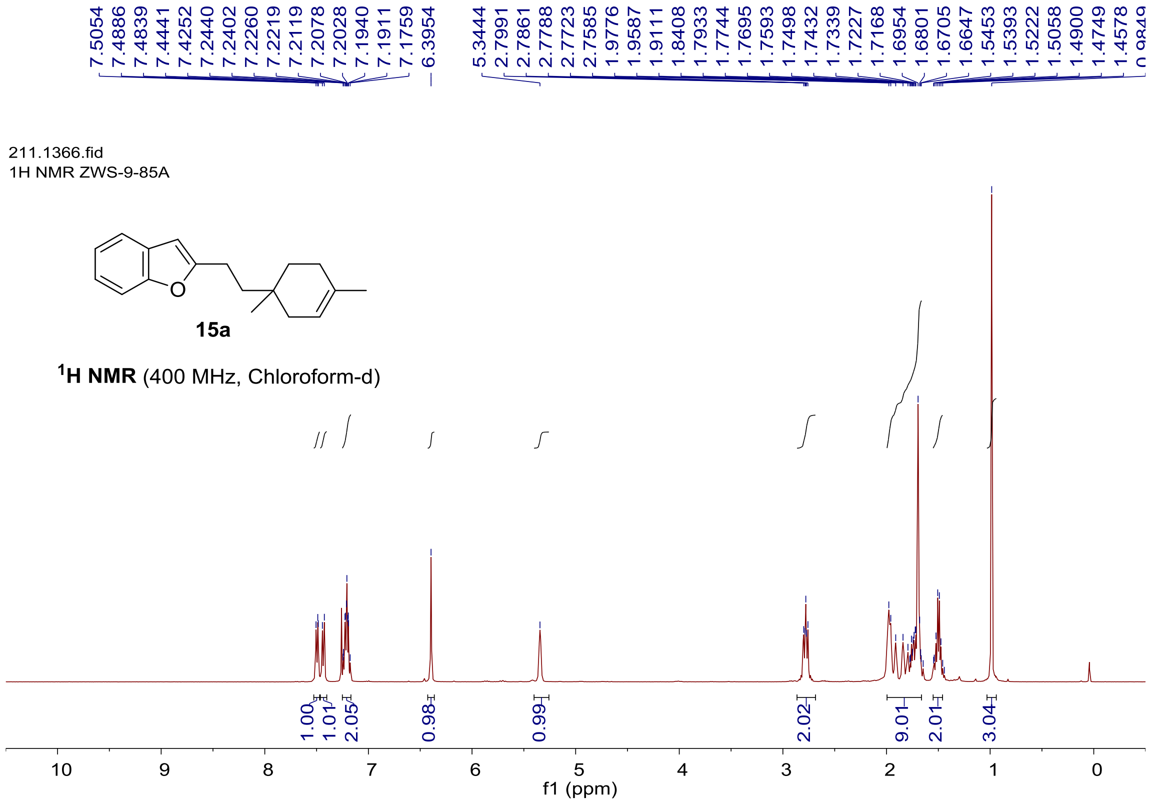
7.5054
7.4886
7.4839
7.4441
7.4252
7.2440
7.2402
7.2260
7.2219
7.2119
7.2078
7.2028
7.1940
7.1911
7.1759
6.3954
5.3444
2.7991
2.7861
2.7788
2.7723
2.7585
1.9776
1.9587
1.9111
1.8408
1.7933
1.7744
1.7695
1.7593
1.7498
1.7432
1.7339
1.7227
1.7168
1.6954
1.6801
1.6705
1.6647
1.5453
1.5393
1.5222
1.5058
1.4900
1.4749
1.4578
211.1366.fid
1H NMR ZWS-9-85A
O
15a
1H NMR (400 MHz, Chloroform-d)
1.00
1.01
2.05
0.98
0.99
2.02
9.01
2.01
3.04
10
9
8
7
6
5
4
3
2
1
0
f1 (ppm)

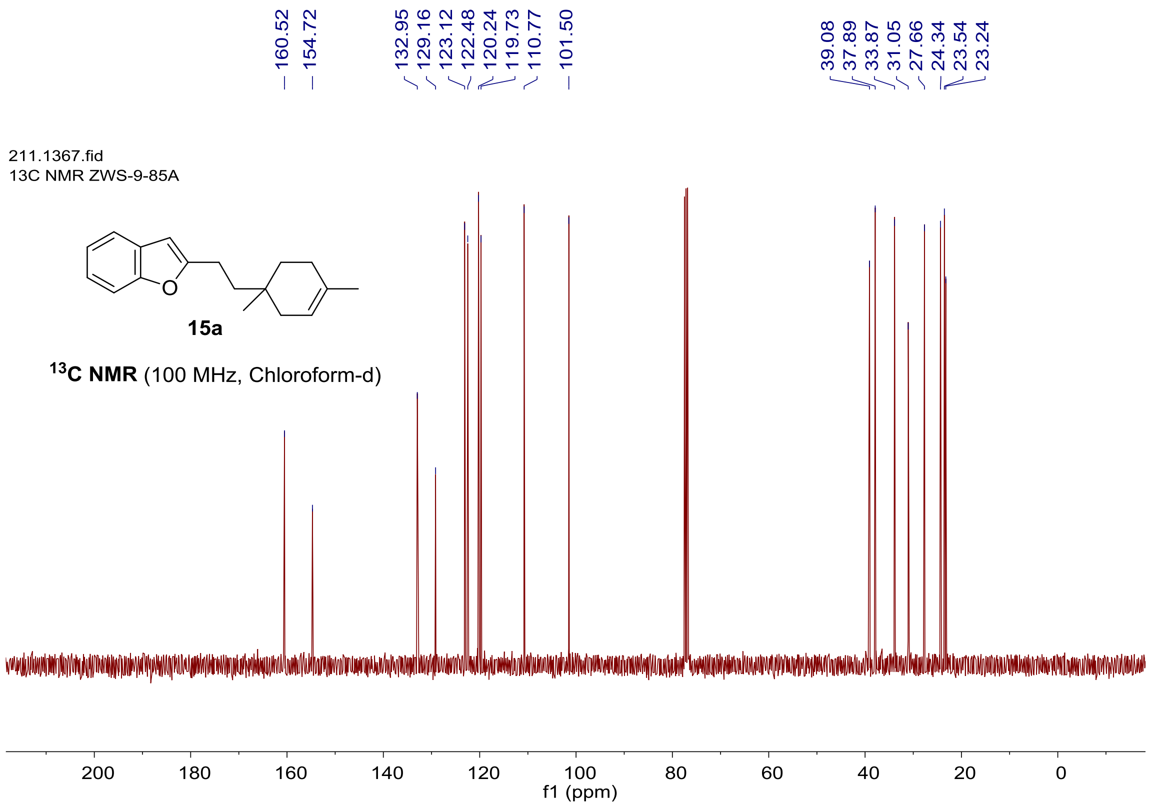
160.52
154.72
132.95
129.16
123.12
122.48
120.24
119.73
110.77
101.50
39.08
37.89
33.87
31.05
27.66
24.34
23.54
23.24
211.1367.fid
13C NMR ZWS-9-85A
O
15a
13C NMR (100 MHz, Chloroform-d)
200
180
160
140
120
100
80
60
40
20
0
f1 (ppm)

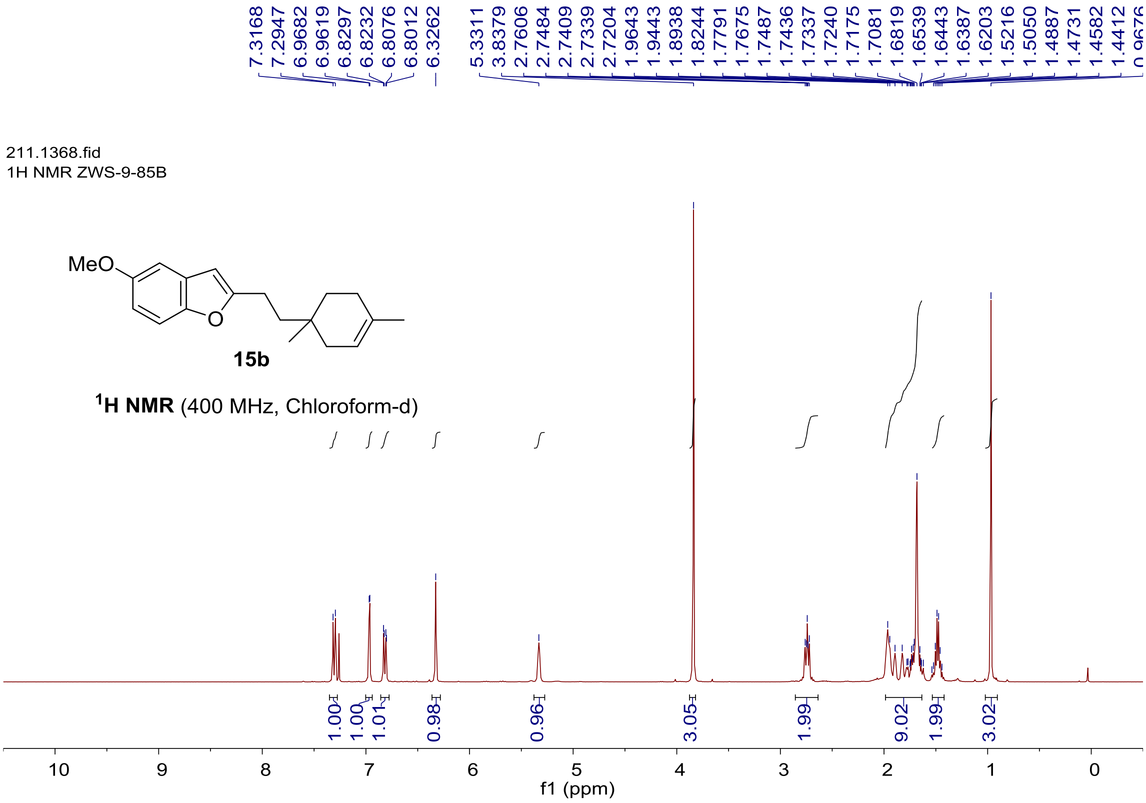
7.3168
7.2947
6.9682
6.9619
6.8297
6.8232
6.8076
6.8012
6.3262
5.3311
3.8379
2.7606
2.7484
2.7409
2.7339
2.7204
1.9643
1.9443
1.8938
1.8244
1.7791
1.7675
1.7487
1.7436
1.7337
1.7240
1.7175
1.7081
1.6819
1.6539
1.6443
1.6387
1.6203
1.5216
1.5050
1.4887
1.4731
1.4582
1.4412
211.1368.fid
1H NMR ZWS-9-85B
MeO
O
15b
¹H NMR (400 MHz, Chloroform-d)
1.00
1.00
1.01
0.98
0.96
3.05
1.99
9.02
1.99
3.02
10
9
8
7
6
5
4
3
2
1
0
f1 (ppm)

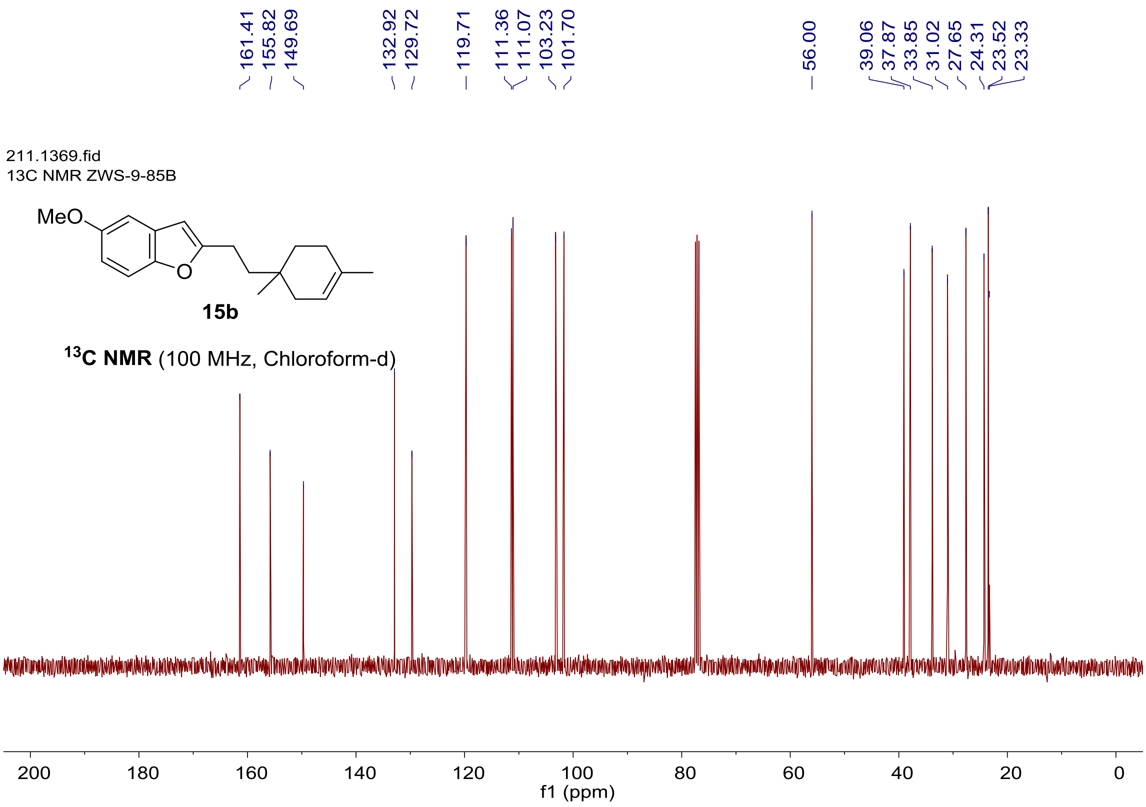
161.41
155.82
149.69
132.92
129.72
119.71
111.36
111.07
103.23
101.70
56.00
39.06
37.87
33.85
31.02
27.65
24.31
23.52
23.33
211.1369.fid
13C NMR ZWS-9-85B
MeO
O
15b
¹³C NMR (100 MHz, Chloroform-d)
200
180
160
140
120
100
80
60
40
20
0
f1 (ppm)

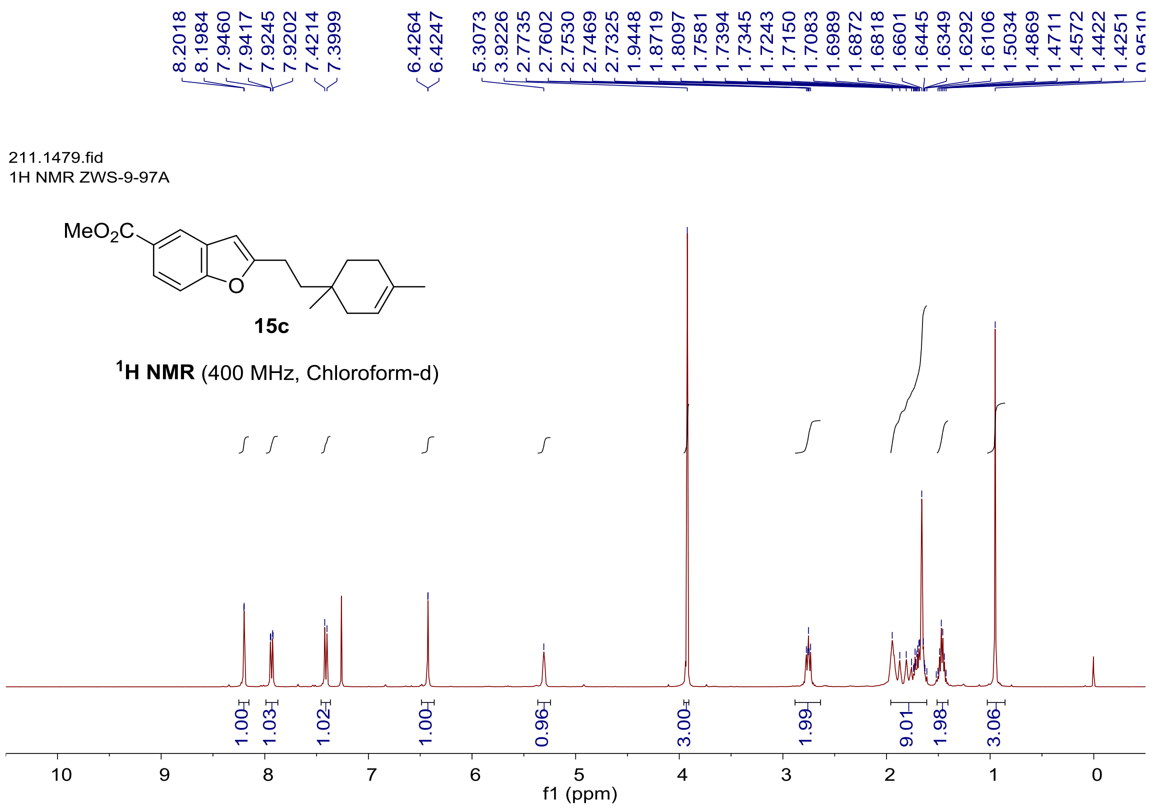
8.2018
8.1984
7.9460
7.9417
7.9245
7.9202
7.4214
7.3999
6.4264
6.4247
5.3073
3.9226
2.7735
2.7602
2.7530
2.7469
2.7325
1.9448
1.8719
1.8097
1.7581
1.7394
1.7345
1.7243
1.7150
1.7083
1.6989
1.6872
1.6818
1.6601
1.6445
1.6349
1.6292
1.6106
1.5034
1.4869
1.4711
1.4572
1.4422
1.4251
211.1479.fid
1H NMR ZWS-9-97A
MeO_2C
O
15c
^{1}H NMR (400 MHz, Chloroform-d)
1.00
1.03
1.02
1.00
0.96
3.00
1.99
9.01
1.98
3.06
10
9
8
7
6
5
4
3
2
1
0
f1 (ppm)

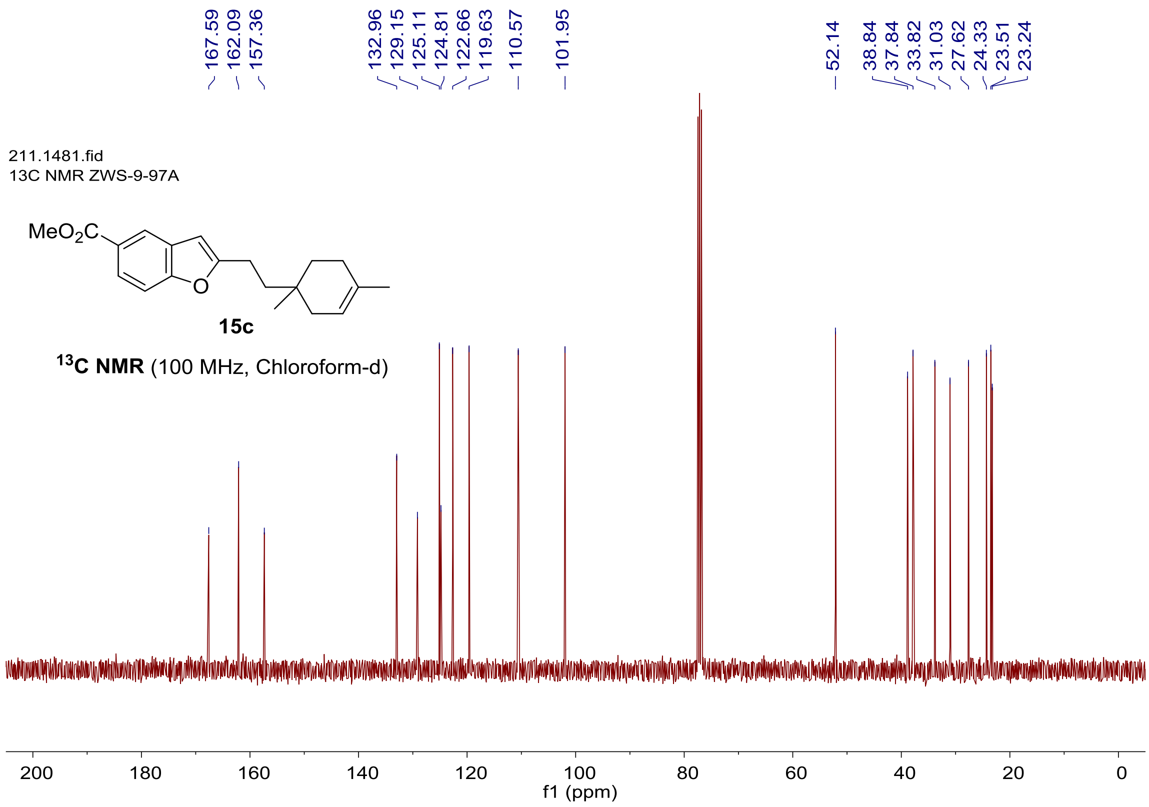
167.59
162.09
157.36
132.96
129.15
125.11
124.81
122.66
119.63
110.57
101.95
52.14
38.84
37.84
33.82
31.03
27.62
24.33
23.51
23.24
211.1481.fid
13C NMR ZWS-9-97A
MeO_2C
O
15c
^{13}C NMR (100 MHz, Chloroform-d)
200
180
160
140
120
100
80
60
40
20
0
f1 (ppm)

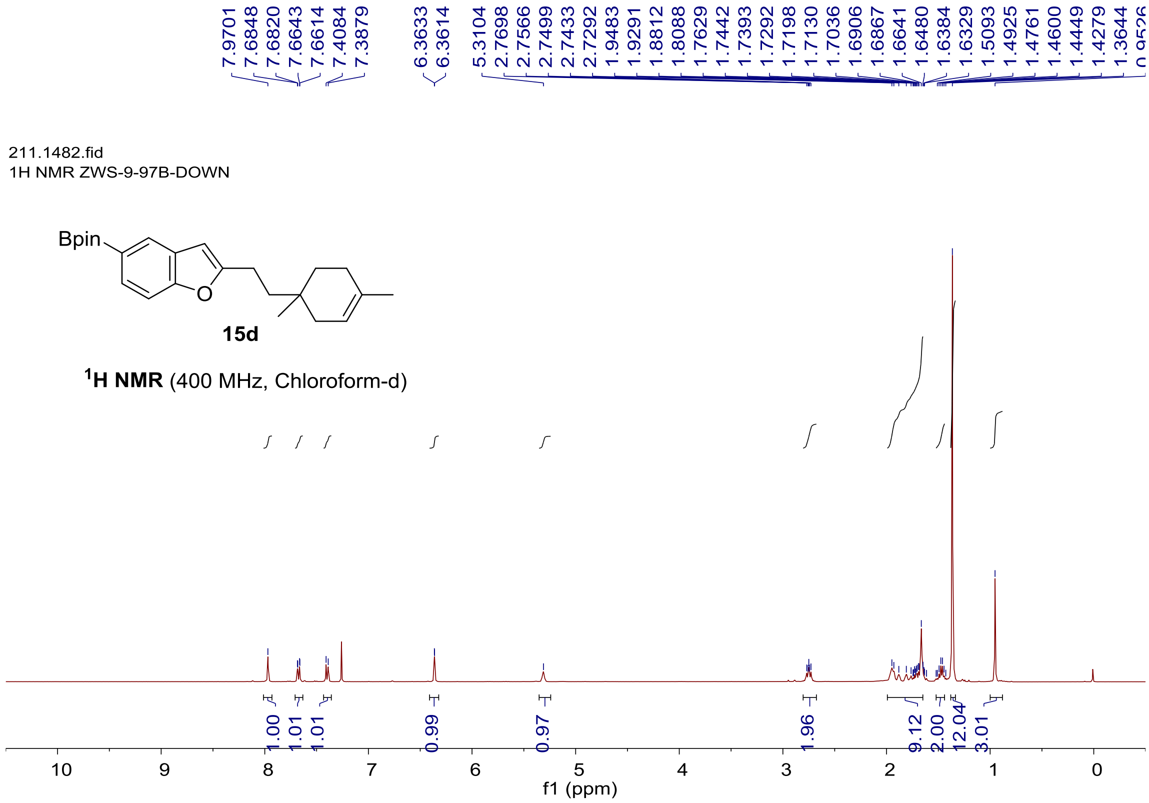
7.9701
7.6848
7.6820
7.6643
7.6614
7.4084
7.3879
6.3633
6.3614
5.3104
2.7698
2.7566
2.7499
2.7433
2.7292
1.9483
1.9291
1.8812
1.8088
1.7629
1.7442
1.7393
1.7292
1.7198
1.7130
1.7036
1.6906
1.6867
1.6641
1.6480
1.6384
1.6329
1.5093
1.4925
1.4761
1.4600
1.4449
1.4279
1.3644
211.1482.fid
1H NMR ZWS-9-97B-DOWN
Bpin
O
15d
1H NMR (400 MHz, Chloroform-d)
1.00
1.01
1.01
0.99
0.97
1.96
9.12
2.00
12.04
3.01
10
9
8
7
6
5
4
3
2
1
0
f1 (ppm)

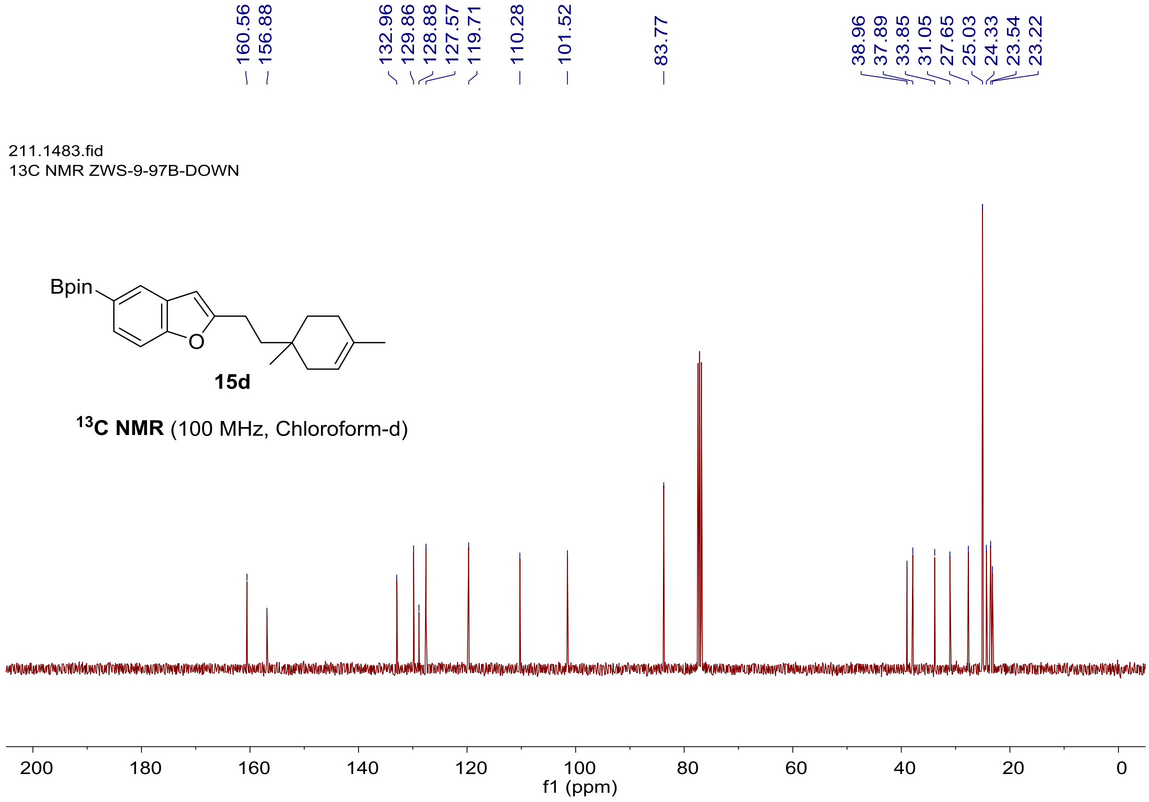
160.56
156.88
132.96
129.86
128.88
127.57
119.71
110.28
101.52
83.77
38.96
37.89
33.85
31.05
27.65
25.03
24.33
23.54
23.22
211.1483.fid
13C NMR ZWS-9-97B-DOWN
Bpin
O
15d
13C NMR (100 MHz, Chloroform-d)
200
180
160
140
120
100
80
60
40
20
0
f1 (ppm)

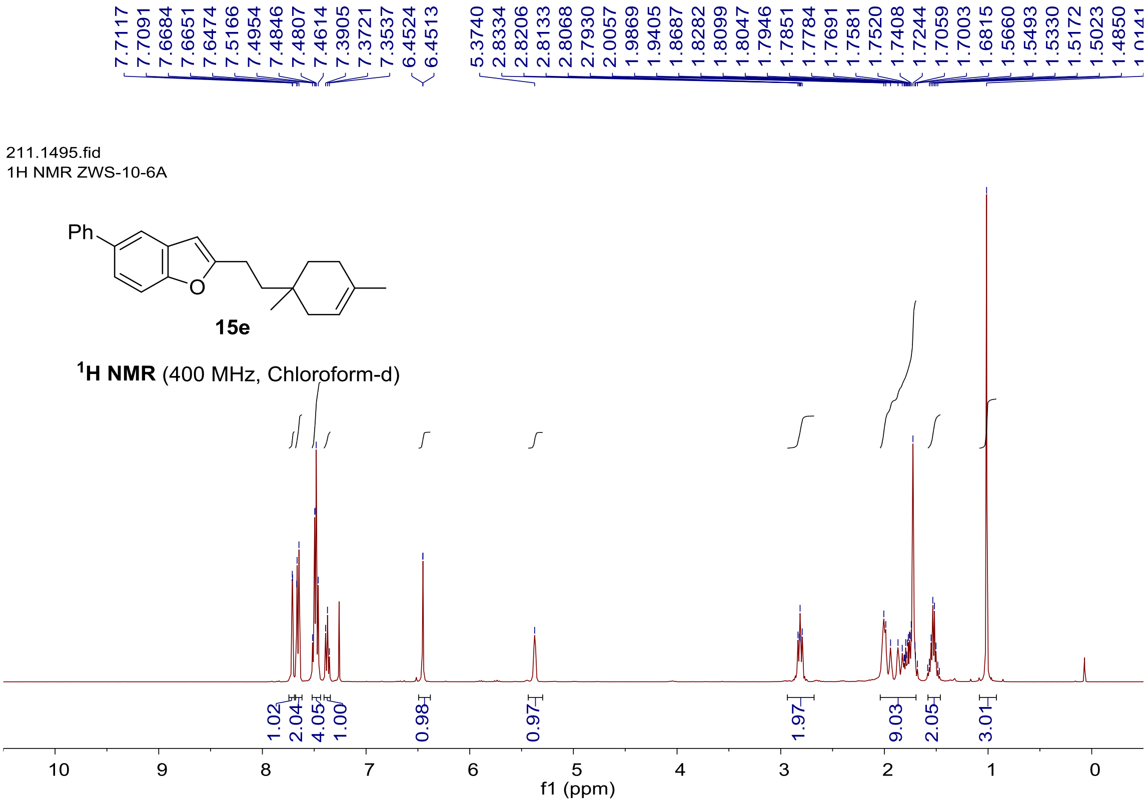
7.7117
7.7091
7.6684
7.6651
7.6474
7.5166
7.4954
7.4846
7.4807
7.4614
7.3905
7.3721
7.3537
6.4524
6.4513
5.3740
2.8334
2.8206
2.8133
2.8068
2.7930
2.0057
1.9869
1.9405
1.8687
1.8282
1.8099
1.8047
1.7946
1.7851
1.7784
1.7691
1.7581
1.7520
1.7408
1.7244
1.7059
1.7003
1.6815
1.5660
1.5493
1.5330
1.5172
1.5023
1.4850
211.1495.fid
1H NMR ZWS-10-6A
Ph
O
15e
1H NMR (400 MHz, Chloroform-d)
1.02
2.04
4.05
1.00
0.98
0.97
1.97
9.03
2.05
3.01
f1 (ppm)

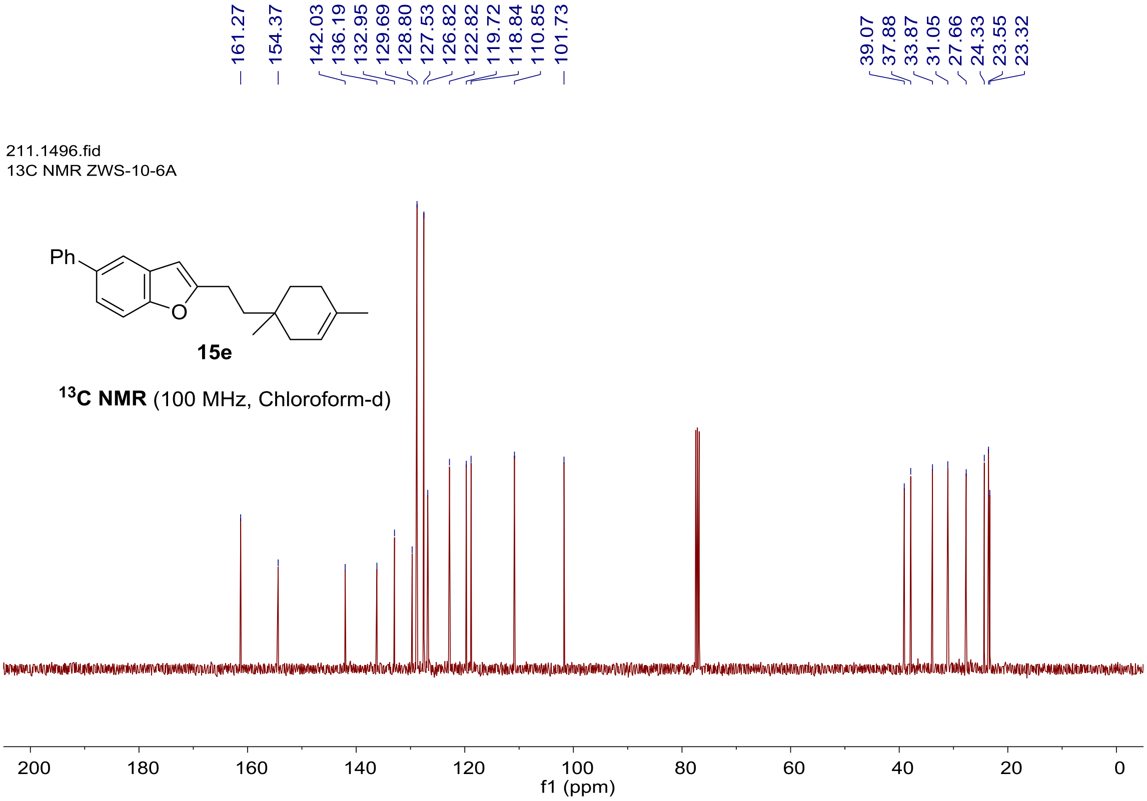
161.27
154.37
142.03
136.19
132.95
129.69
128.80
127.53
126.82
122.82
119.72
118.84
110.85
101.73
39.07
37.88
33.87
31.05
27.66
24.33
23.55
23.32
211.1496.fid
13C NMR ZWS-10-6A
Ph
O
15e
13C NMR (100 MHz, Chloroform-d)
f1 (ppm)

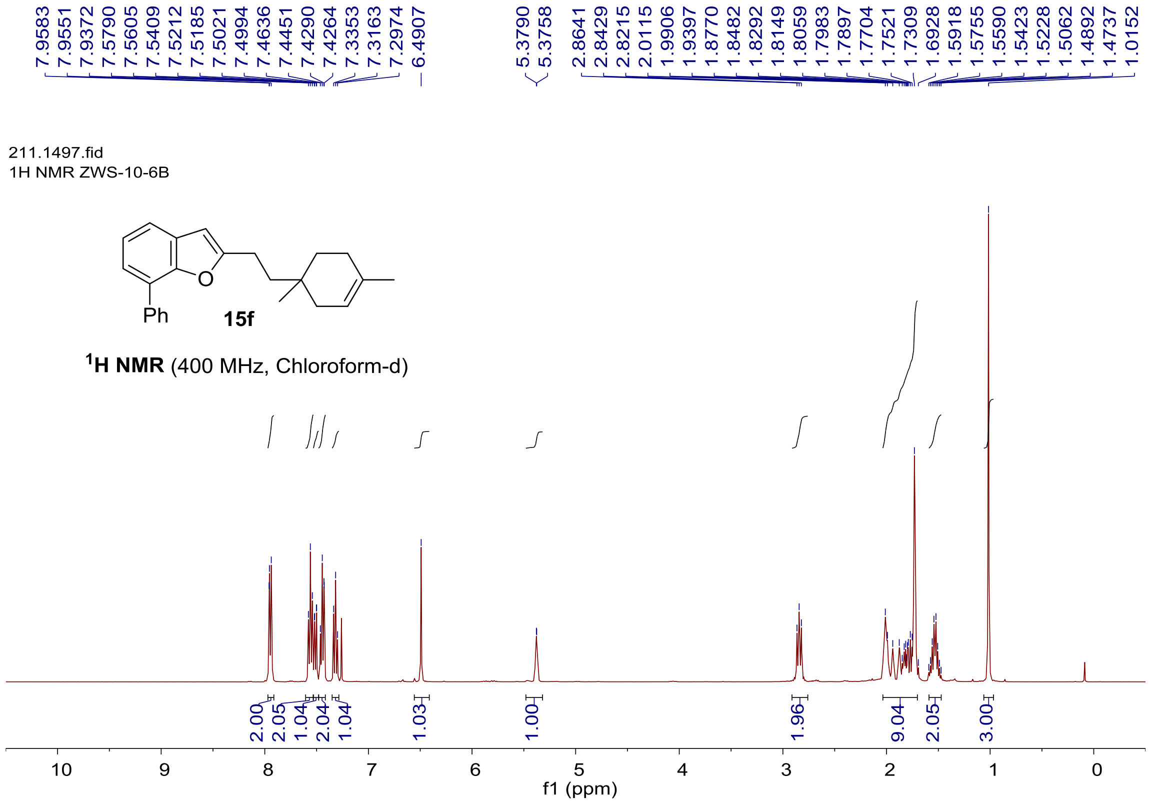

7.9583
7.9551
7.9372
7.5790
7.5605
7.5409
7.5212
7.5185
7.5021
7.4994
7.4636
7.4451
7.4290
7.4264
7.3353
7.3163
7.2974
6.4907
5.3790
5.3758
2.8641
2.8429
2.8215
2.0115
1.9906
1.9397
1.8770
1.8482
1.8292
1.8149
1.8059
1.7983
1.7897
1.7704
1.7521
1.7309
1.6928
1.5918
1.5755
1.5590
1.5423
1.5228
1.5062
1.4892
1.4737
1.0152
211.1497.fid
1H NMR ZWS-10-6B
O
Ph
15f
1H NMR (400 MHz, Chloroform-d)
2.00
2.05
1.04
2.04
1.04
1.03
1.00
1.96
9.04
2.05
3.00
f1 (ppm)

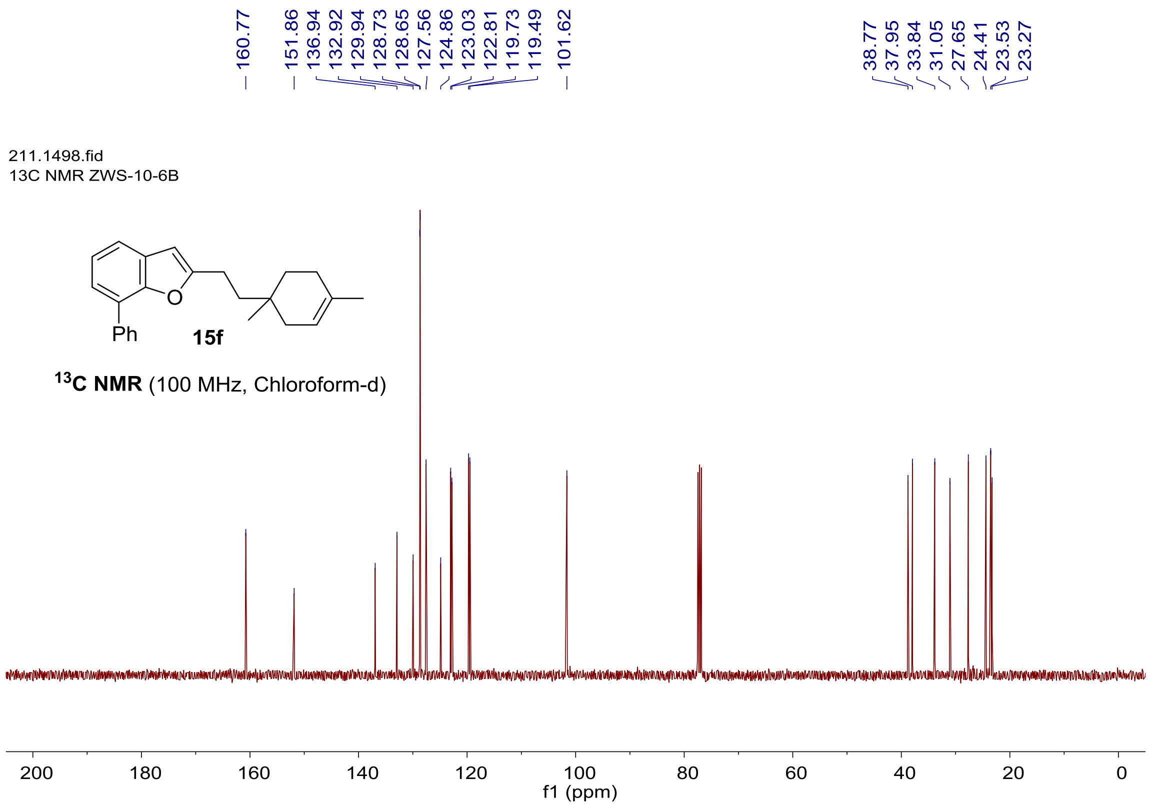

160.77
151.86
136.94
132.92
129.94
128.73
128.65
127.56
124.86
123.03
122.81
119.73
119.49
101.62
38.77
37.95
33.84
31.05
27.65
24.41
23.53
23.27
211.1498.fid
13C NMR ZWS-10-6B
O
Ph
15f
13C NMR (100 MHz, Chloroform-d)
f1 (ppm)

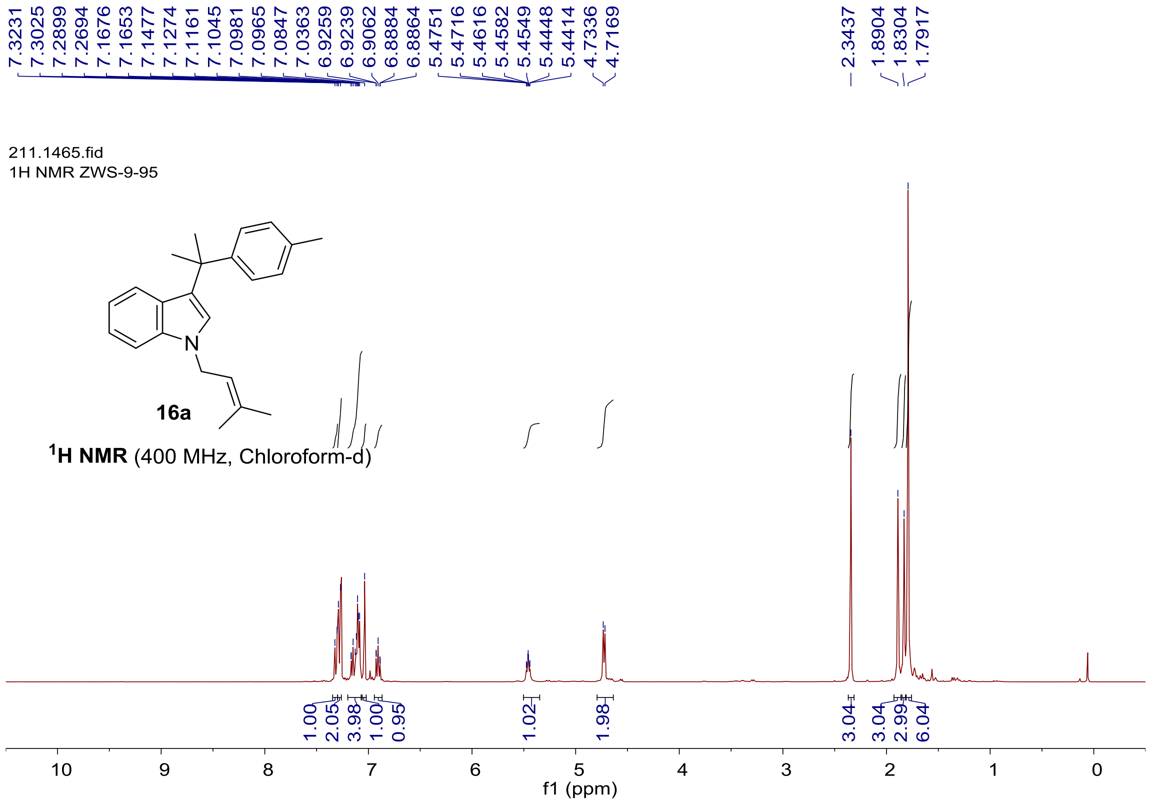

7.3231
7.3025
7.2899
7.2694
7.1676
7.1653
7.1477
7.1274
7.1161
7.1045
7.0981
7.0965
7.0847
7.0363
6.9259
6.9239
6.9062
6.8884
6.8864
5.4751
5.4716
5.4616
5.4582
5.4549
5.4448
5.4414
4.7336
4.7169
2.3437
1.8904
1.8304
1.7917
211.1465.fid
1H NMR ZWS-9-95
N
16a
1H NMR (400 MHz, Chloroform-d)
1.00
2.05
3.98
1.00
0.95
1.02
1.98
3.04
3.04
2.99
6.04
f1 (ppm)

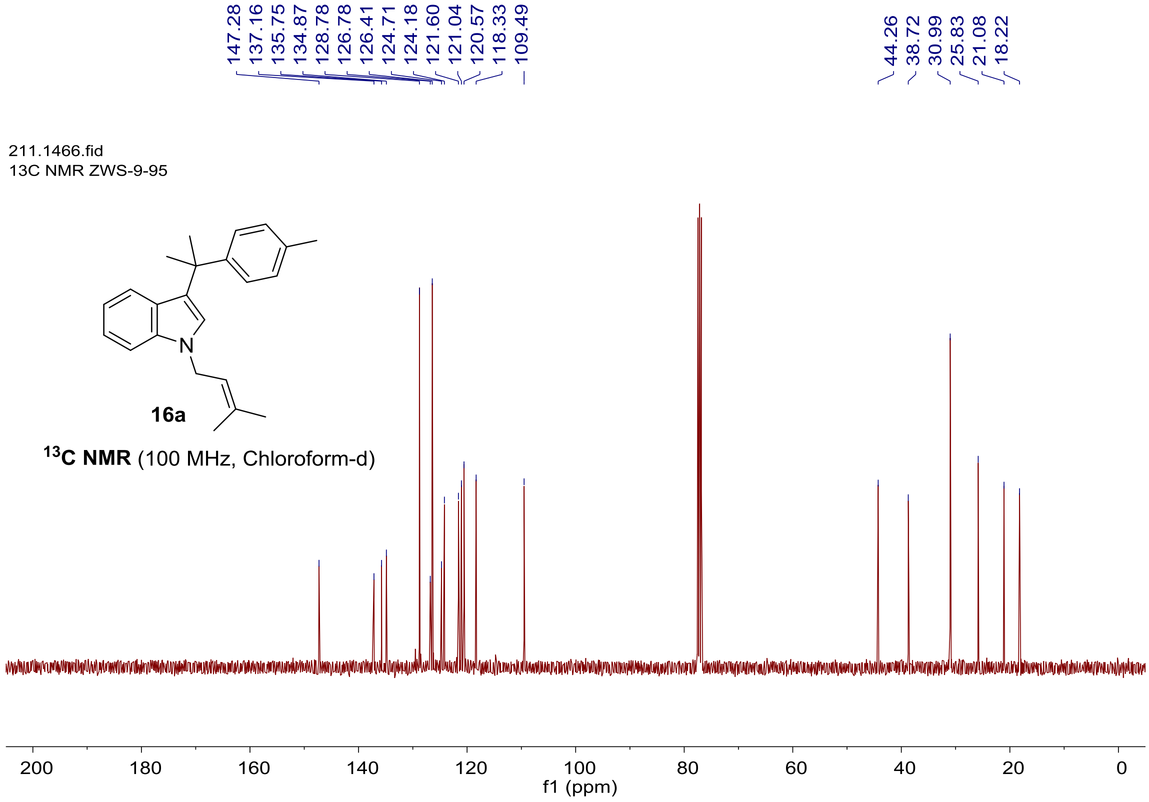

147.28
137.16
135.75
134.87
128.78
126.78
126.41
124.71
124.18
121.60
121.04
120.57
118.33
109.49
44.26
38.72
30.99
25.83
21.08
18.22
211.1466.fid
13C NMR ZWS-9-95
N
16a
13C NMR (100 MHz, Chloroform-d)
f1 (ppm)

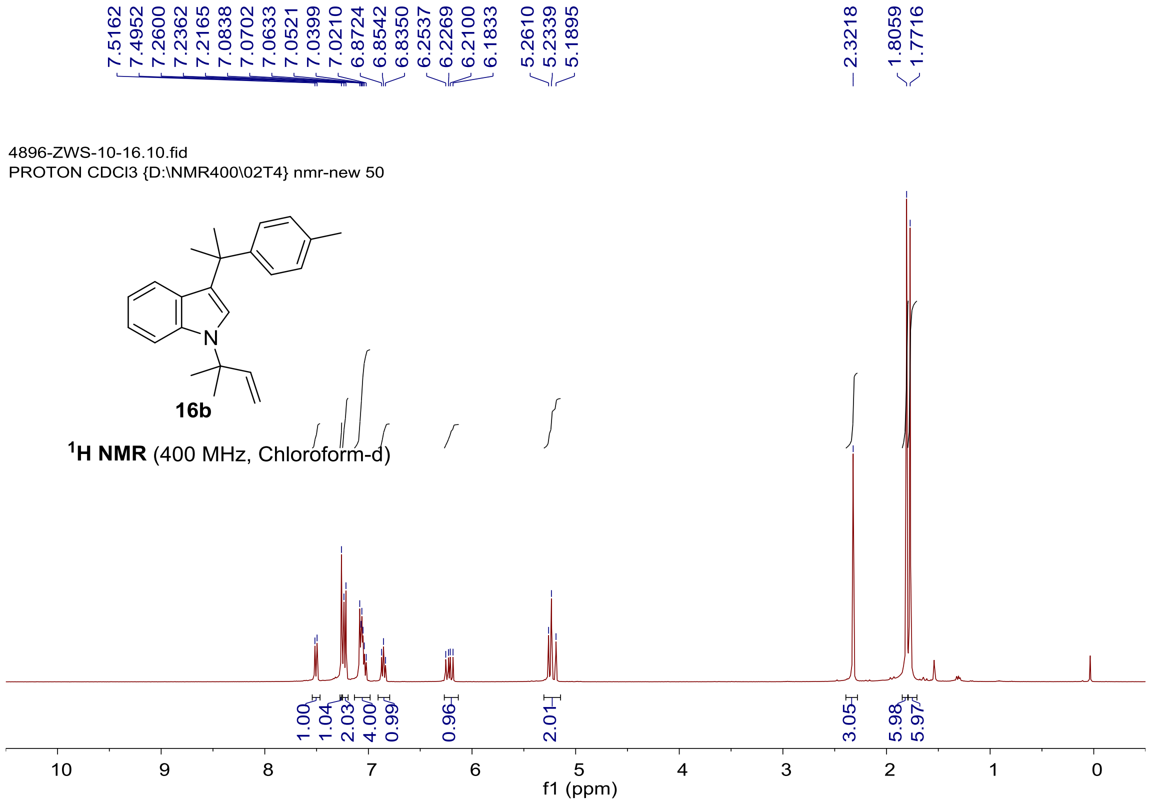
7.5162
7.4952
7.2600
7.2362
7.2165
7.0838
7.0702
7.0633
7.0521
7.0399
7.0210
6.8724
6.8542
6.8350
6.2537
6.2269
6.2100
6.1833
5.2610
5.2339
5.1895
2.3218
1.8059
1.7716
4896-ZWS-10-16.10.fid
PROTON CDCl3 {D:\NMR400\02T4} nmr-new 50
N
16b
1H NMR (400 MHz, Chloroform-d)
1.00
1.04
2.03
4.00
0.99
0.96
2.01
3.05
5.98
5.97
f1 (ppm)

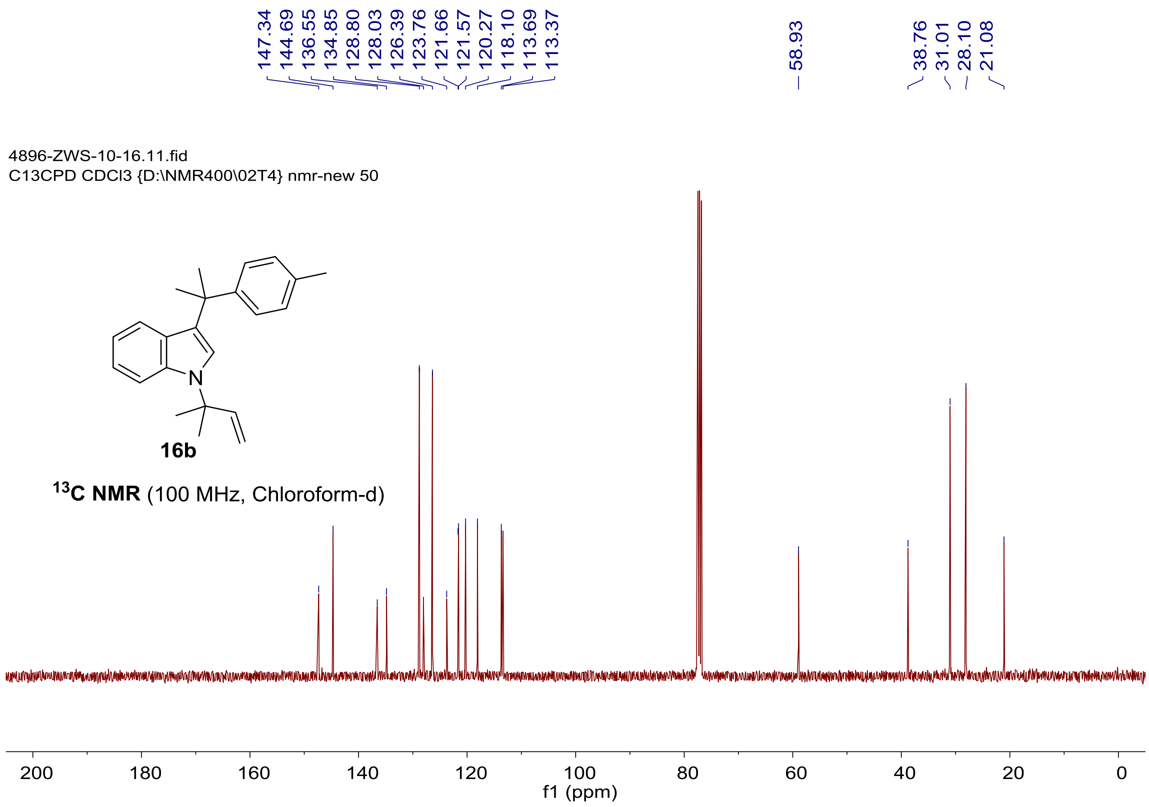
147.34
144.69
136.55
134.85
128.80
128.03
126.39
123.76
121.66
121.57
120.27
118.10
113.69
113.37
58.93
38.76
31.01
28.10
21.08
4896-ZWS-10-16.11.fid
C13CPD CDCl3 {D:\NMR400\02T4} nmr-new 50
N
16b
13C NMR (100 MHz, Chloroform-d)
f1 (ppm)

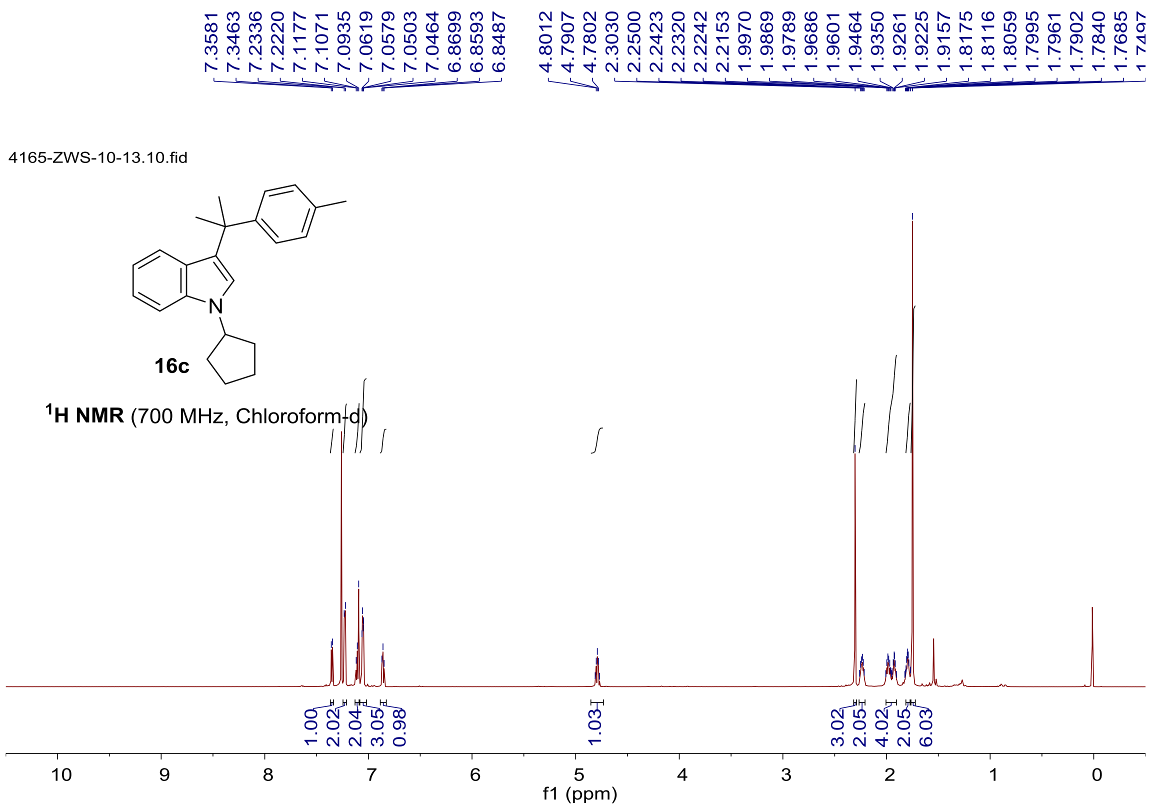
7.3581
7.3463
7.2336
7.2220
7.1177
7.1071
7.0935
7.0619
7.0579
7.0503
7.0464
6.8699
6.8593
6.8487
4.8012
4.7907
4.7802
2.3030
2.2500
2.2423
2.2320
2.2242
2.2153
1.9970
1.9869
1.9789
1.9686
1.9601
1.9464
1.9350
1.9261
1.9225
1.9157
1.8175
1.8116
1.8059
1.7995
1.7961
1.7902
1.7840
1.7685
1.7497
4165-ZWS-10-13.10.fid
16c
N
^{1}H NMR (700 MHz, Chloroform-d)
1.00
2.02
2.04
3.05
0.98
1.03
3.02
2.05
4.02
2.05
6.03
10
9
8
7
6
5
4
3
2
1
0
f1 (ppm)

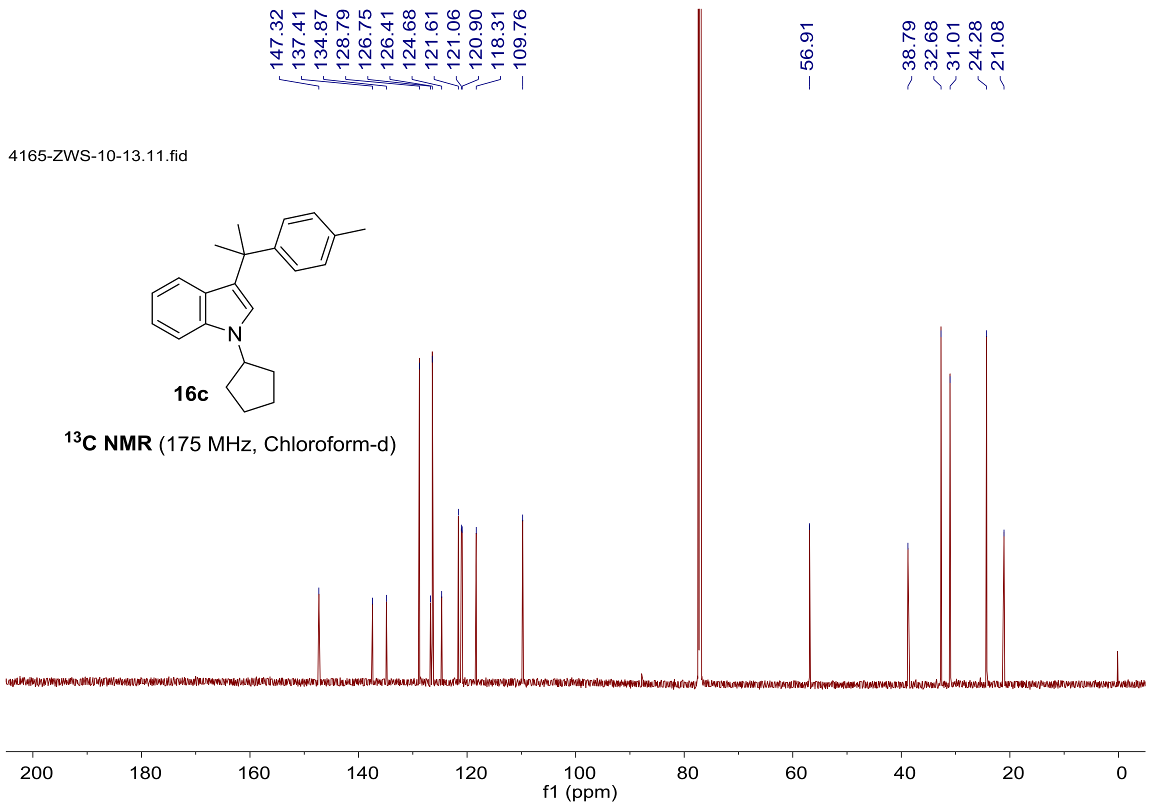
147.32
137.41
134.87
128.79
126.75
126.41
124.68
121.61
121.06
120.90
118.31
109.76
56.91
38.79
32.68
31.01
24.28
21.08
4165-ZWS-10-13.11.fid
N
16c
^{13}C NMR (175 MHz, Chloroform-d)
200
180
160
140
120
100
80
60
40
20
0
f1 (ppm)

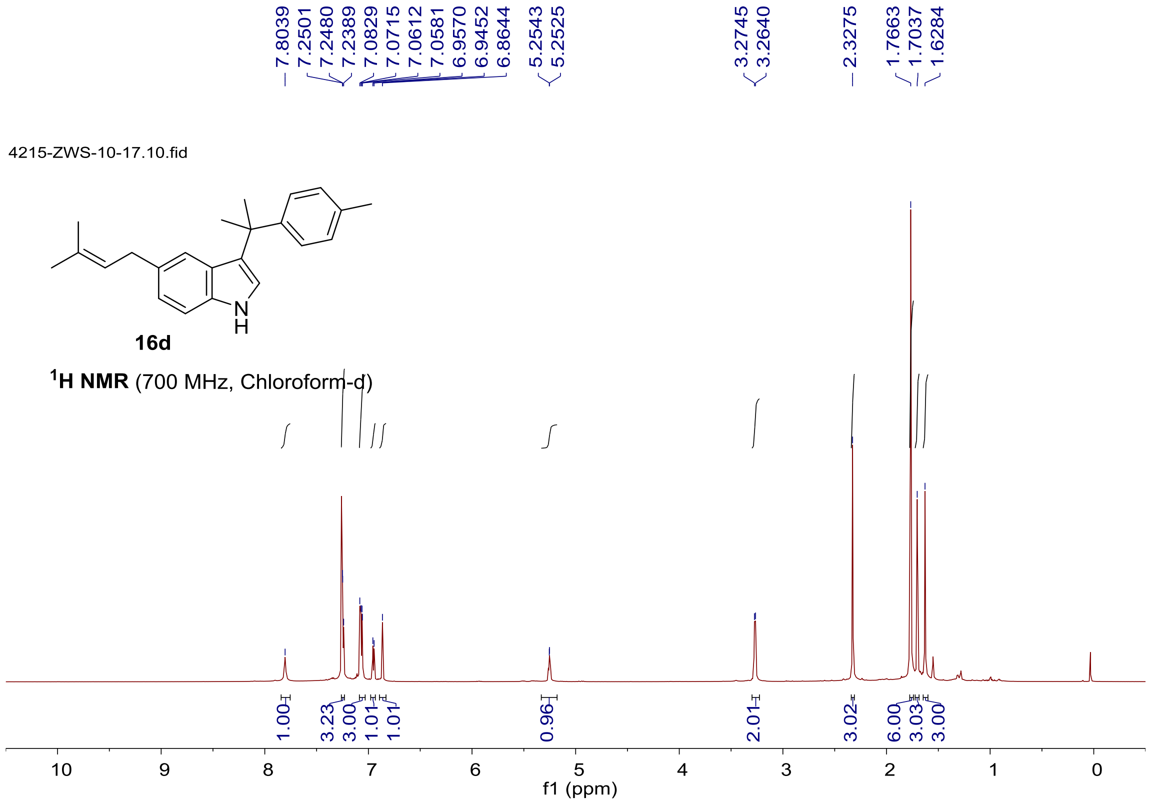
7.8039
7.2501
7.2480
7.2389
7.0829
7.0715
7.0612
7.0581
6.9570
6.9452
6.8644
5.2543
5.2525
3.2745
3.2640
2.3275
1.7663
1.7037
1.6284
4215-ZWS-10-17.10.fid
16d
^{1}H NMR (700 MHz, Chloroform-d)
1.00
3.23
3.00
1.01
1.01
0.96
2.01
3.02
6.00
3.03
3.00
f1 (ppm)

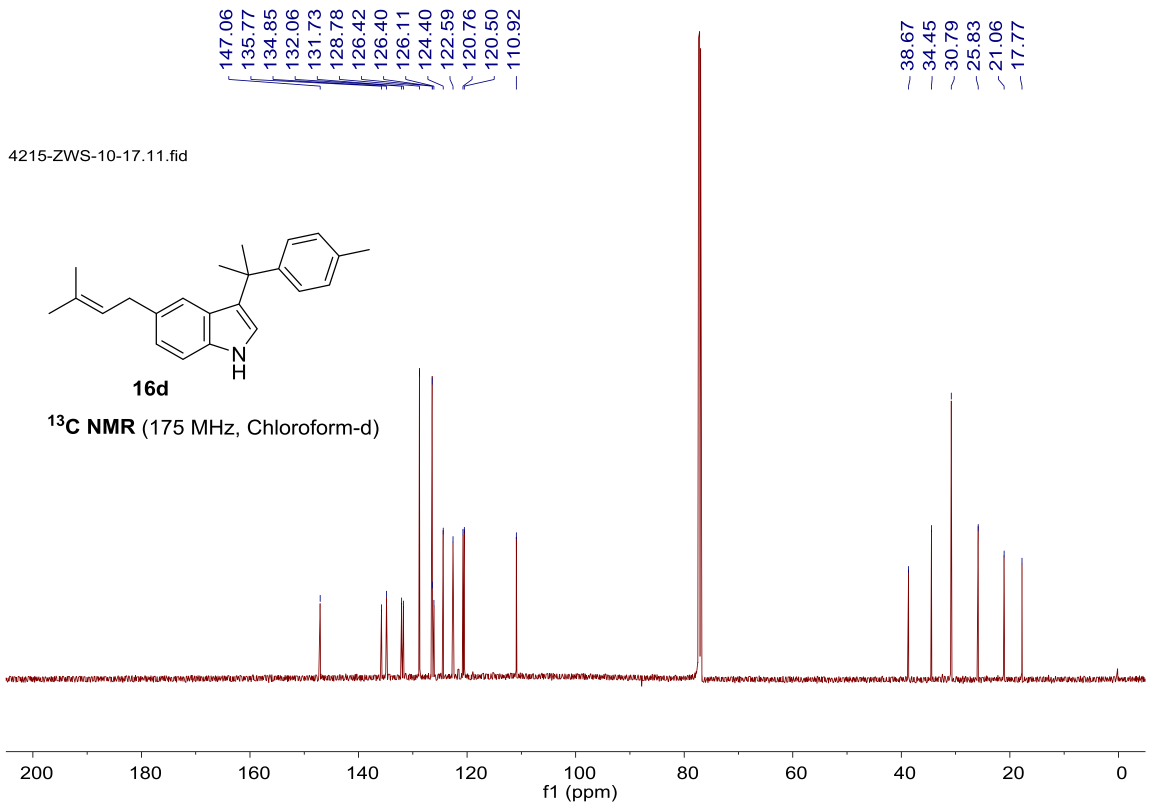
147.06
135.77
134.85
132.06
131.73
128.78
126.42
126.40
126.11
124.40
122.59
120.76
120.50
110.92
38.67
34.45
30.79
25.83
21.06
17.77
4215-ZWS-10-17.11.fid
16d
^{13}C NMR (175 MHz, Chloroform-d)
f1 (ppm)

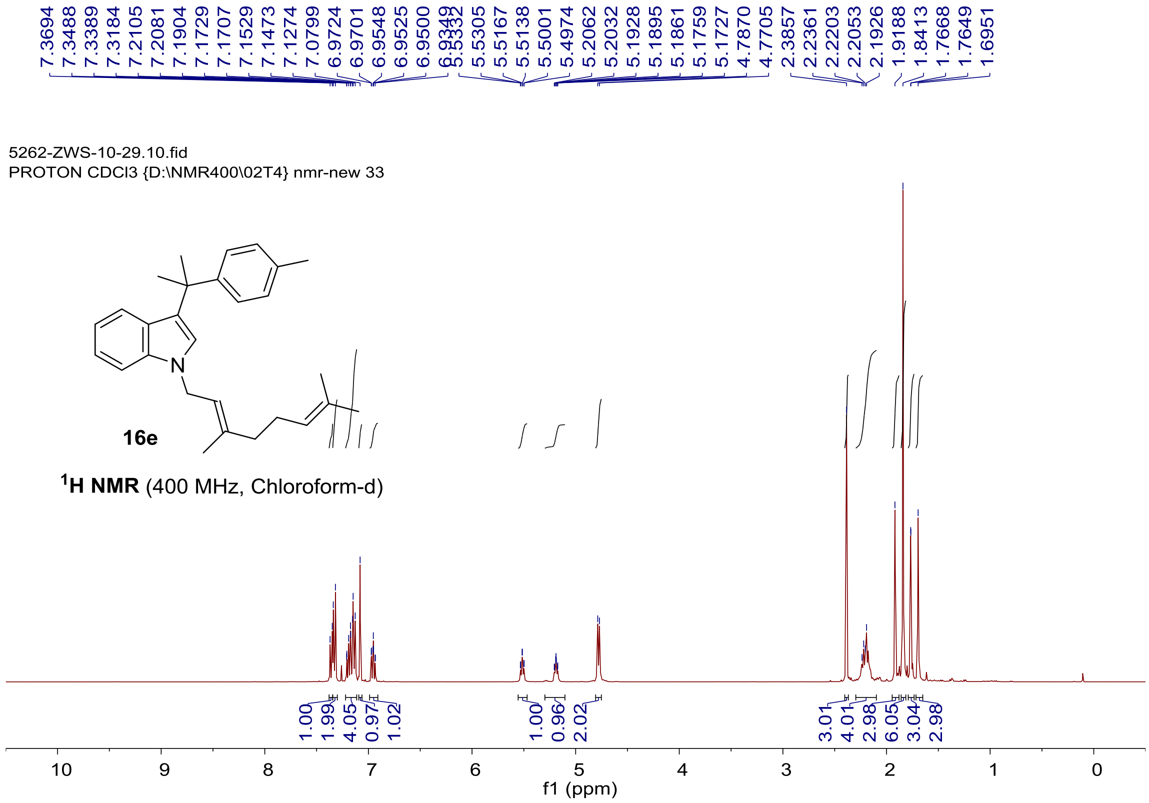
7.3694 7.3488 7.3389 7.3184 7.2105 7.2081 7.1904 7.1729 7.1707 7.1529 7.1473 7.1274 7.0799 6.9724 6.9701 6.9548 6.9525 6.9500 6.9349 5.5332 5.5305 5.5167 5.5138 5.5001 5.4974 5.2062 5.2032 5.1928 5.1895 5.1861 5.1759 5.1727 4.7870 4.7705 2.3857 2.2361 2.2203 2.2053 2.1926 1.9188 1.8413 1.7668 1.7649 1.6951
5262-ZWS-10-29.10.fid
PROTON CDCl3 {D:\NMR400\02T4} nmr-new 33
N
16e
1H NMR (400 MHz, Chloroform-d)
1.00 1.99 4.05 0.97 1.02 1.00 0.96 2.02 3.01 4.01 2.98 6.05 3.04 2.98
10 9 8 7 6 5 4 3 2 1 0
f1 (ppm)

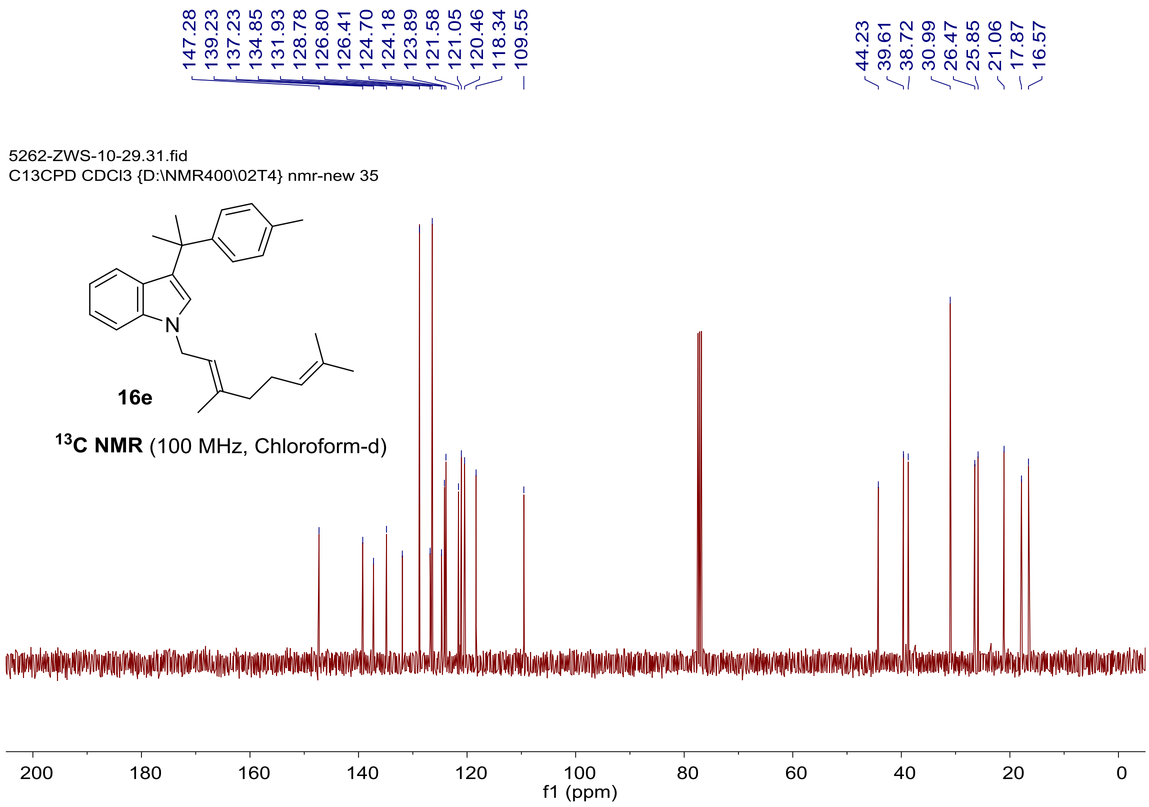
147.28 139.23 137.23 134.85 131.93 128.78 126.80 126.41 124.70 124.18 123.89 121.58 121.05 120.46 118.34 109.55
44.23 39.61 38.72 30.99 26.47 25.85 21.06 17.87 16.57
5262-ZWS-10-29.31.fid
C13CPD CDCl3 {D:\NMR400\02T4} nmr-new 35
N
16e
13C NMR (100 MHz, Chloroform-d)
200 180 160 140 120 100 80 60 40 20 0
f1 (ppm)

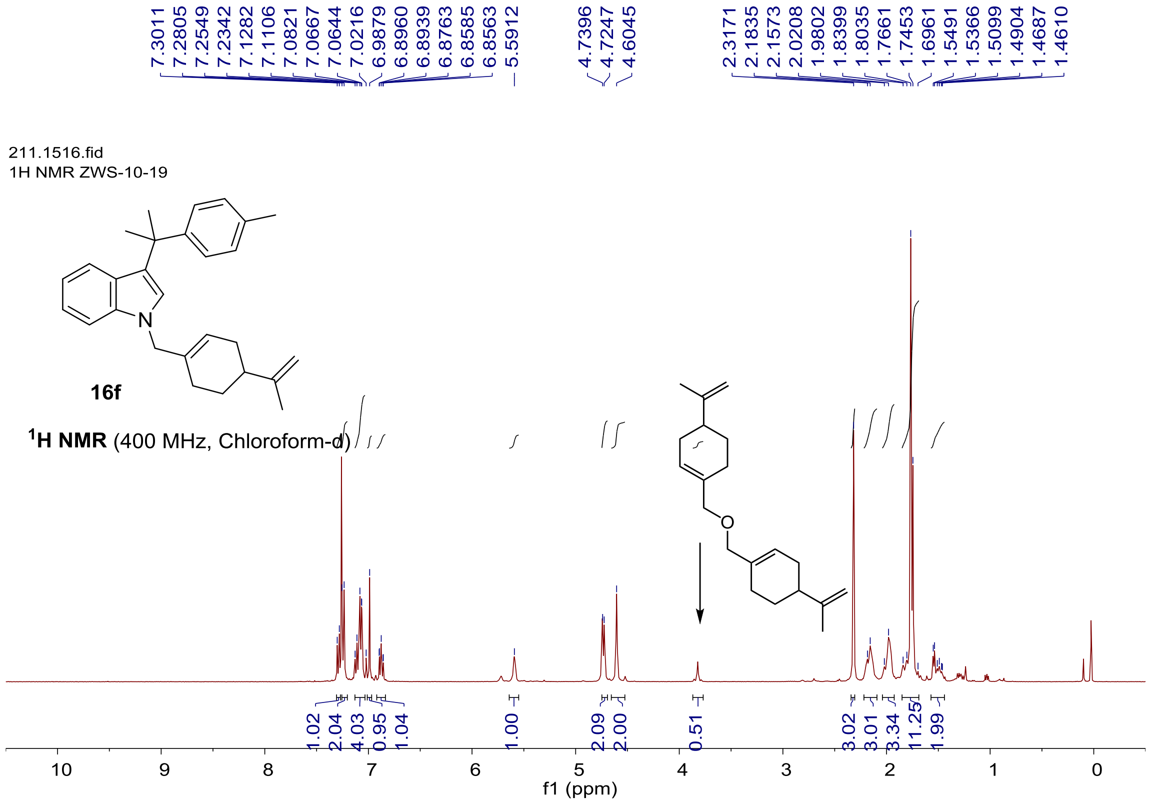
7.3011
7.2805
7.2549
7.2342
7.1282
7.1106
7.0821
7.0667
7.0644
7.0216
6.9879
6.8960
6.8939
6.8763
6.8585
6.8563
5.5912
4.7396
4.7247
4.6045
2.3171
2.1835
2.1573
2.0208
1.9802
1.8399
1.8035
1.7661
1.7453
1.6961
1.5491
1.5366
1.5099
1.4904
1.4687
1.4610
211.1516.fid
1H NMR ZWS-10-19
N
16f
1H NMR (400 MHz, Chloroform-d)
O
1.02
2.04
4.03
0.95
1.04
1.00
2.09
2.00
0.51
3.02
3.01
3.34
11.25
1.99
10
9
8
7
6
5
4
3
2
1
0
f1 (ppm)

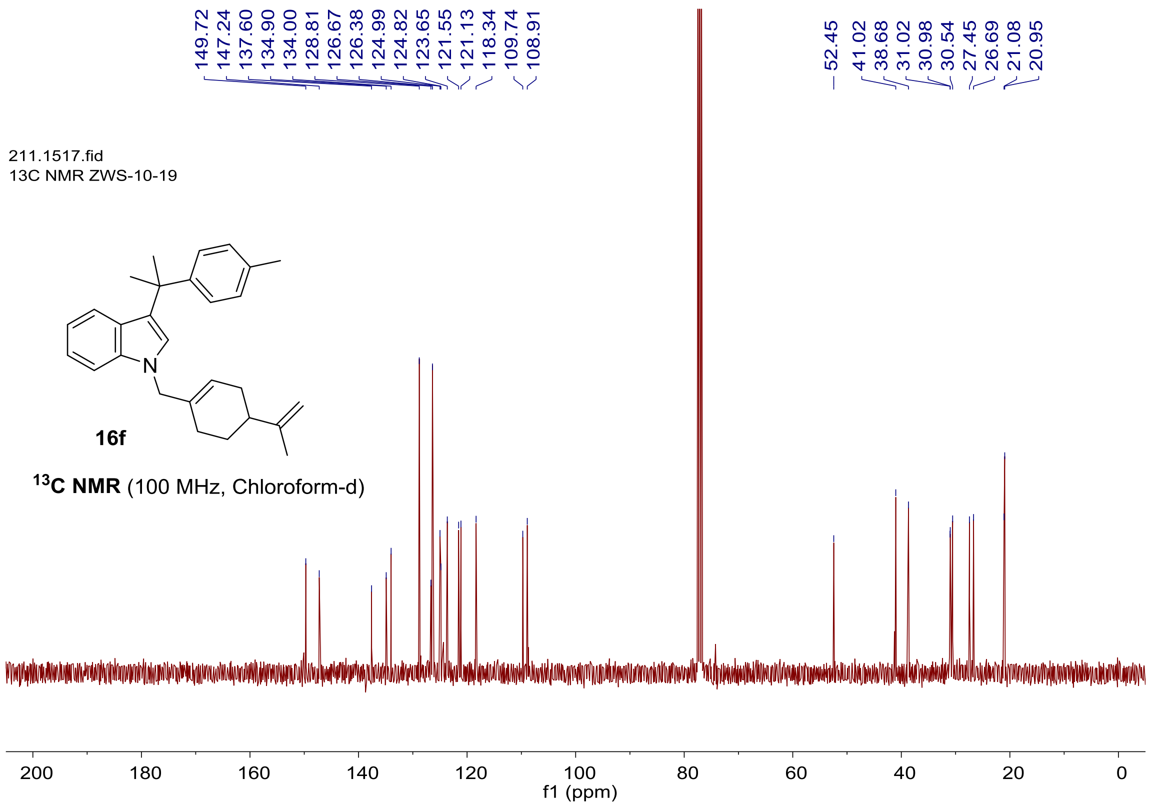
149.72
147.24
137.60
134.90
134.00
128.81
126.67
126.38
124.99
124.82
123.65
121.55
121.13
118.34
109.74
108.91
52.45
41.02
38.68
31.02
30.98
30.54
27.45
26.69
21.08
20.95
211.1517.fid
13C NMR ZWS-10-19
N
16f
13C NMR (100 MHz, Chloroform-d)
200
180
160
140
120
100
80
60
40
20
0
f1 (ppm)

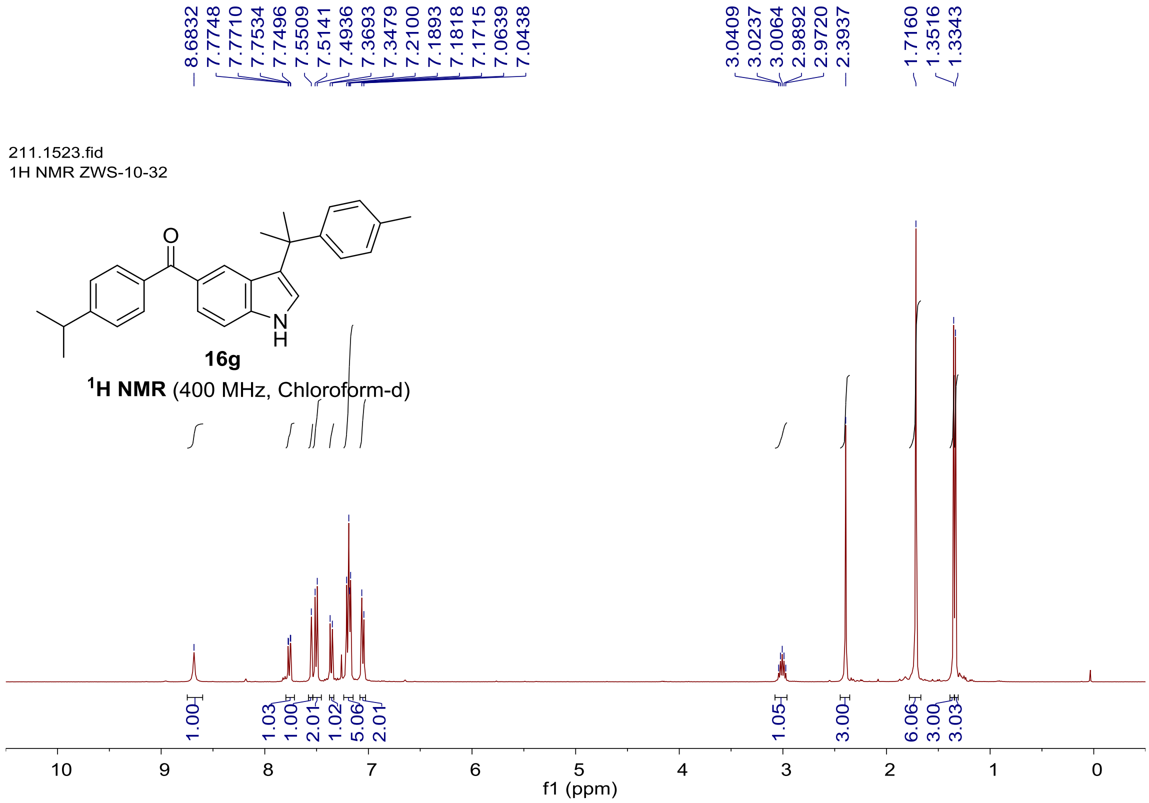
8.6832
7.7748
7.7710
7.7534
7.7496
7.5509
7.5141
7.4936
7.3693
7.3479
7.2100
7.1893
7.1818
7.1715
7.0639
7.0438
3.0409
3.0237
3.0064
2.9892
2.9720
2.3937
1.7160
1.3516
1.3343
211.1523.fid
1H NMR ZWS-10-32
O
N
H
16g
^{1}H NMR (400 MHz, Chloroform-d)
1.00
1.03
1.00
2.01
1.02
5.06
2.01
1.05
3.00
6.06
3.00
3.03
10
9
8
7
6
5
4
3
2
1
0
f1 (ppm)

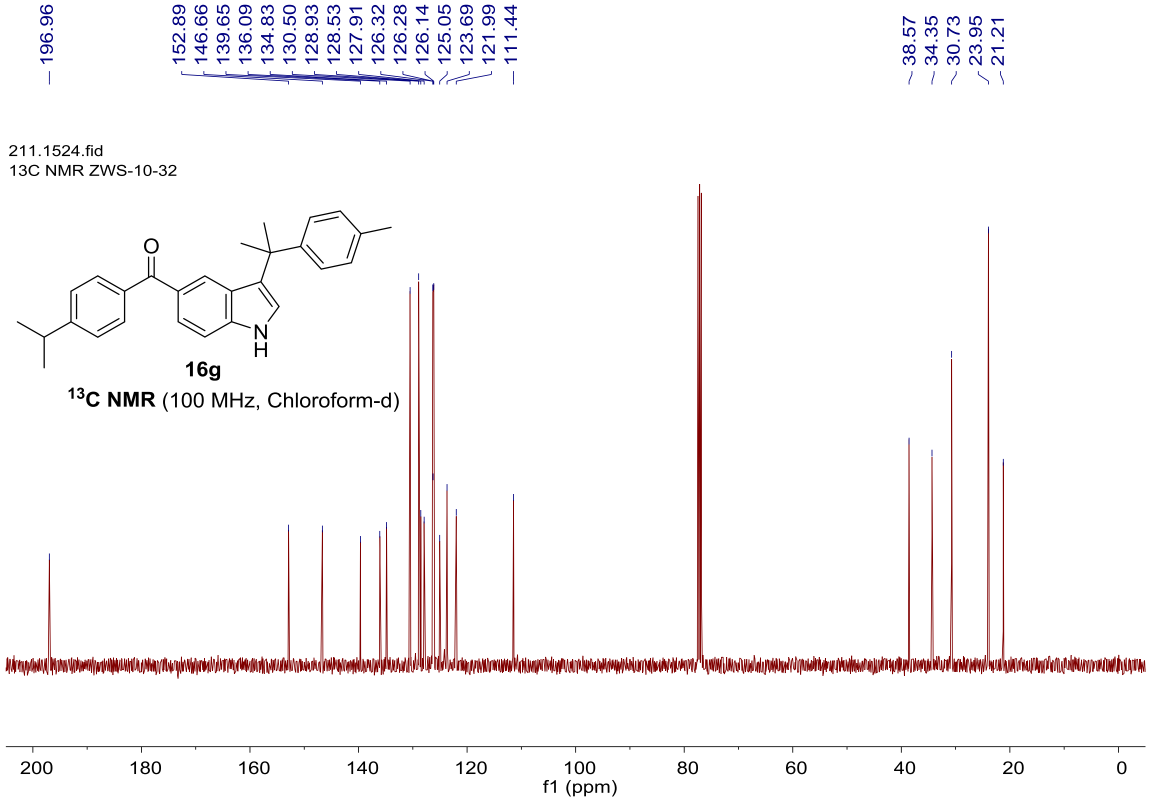
196.96
152.89
146.66
139.65
136.09
134.83
130.50
128.93
128.53
127.91
126.32
126.28
126.14
125.05
123.69
121.99
111.44
38.57
34.35
30.73
23.95
21.21
211.1524.fid
13C NMR ZWS-10-32
O
N
H
16g
^{13}C NMR (100 MHz, Chloroform-d)
200
180
160
140
120
100
80
60
40
20
0
f1 (ppm)

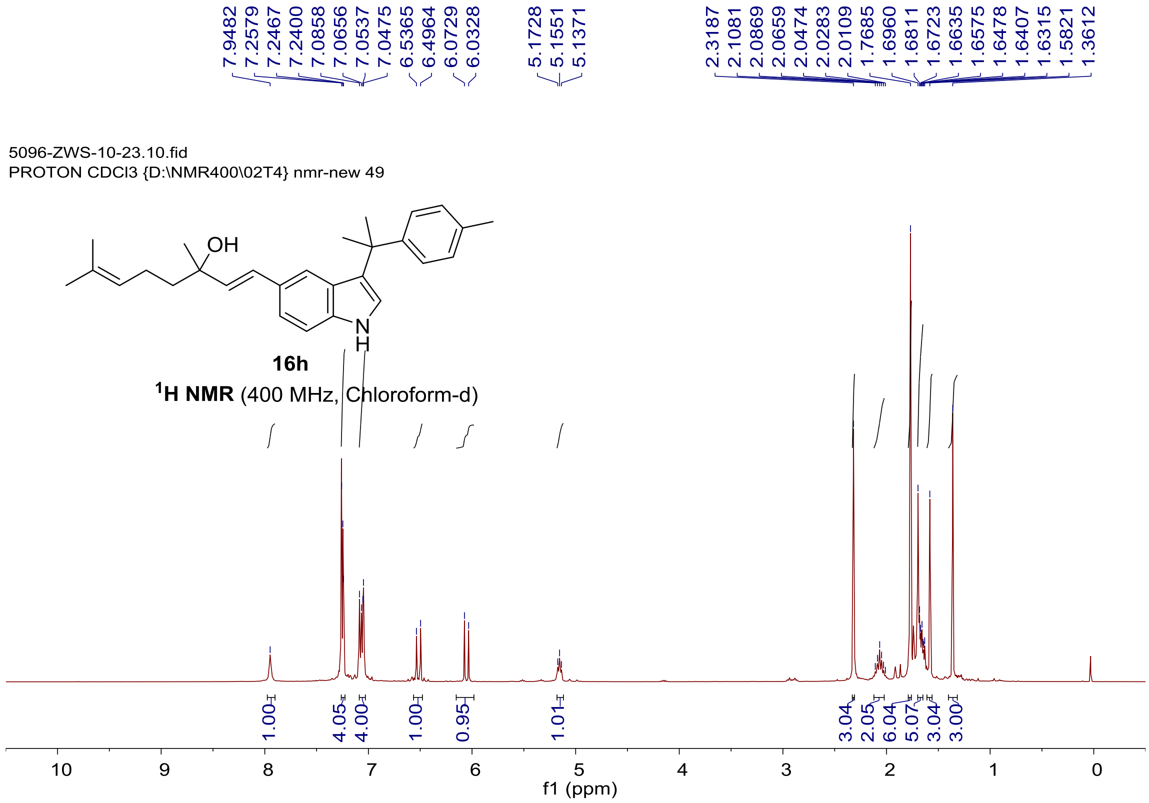
7.9482
7.2579
7.2467
7.2400
7.0858
7.0656
7.0537
7.0475
6.5365
6.4964
6.0729
6.0328
5.1728
5.1551
5.1371
2.3187
2.1081
2.0869
2.0659
2.0474
2.0283
2.0109
1.7685
1.6960
1.6811
1.6723
1.6635
1.6575
1.6478
1.6407
1.6315
1.5821
1.3612
5096-ZWS-10-23.10.fid
PROTON CDCl3 {D:\NMR400\02T4} nmr-new 49
OH
N
H
16h
1H NMR (400 MHz, Chloroform-d)
1.00
4.05
4.00
1.00
0.95
1.01
3.04
2.05
6.04
5.07
3.04
3.00
10
9
8
7
6
5
4
3
2
1
0
f1 (ppm)

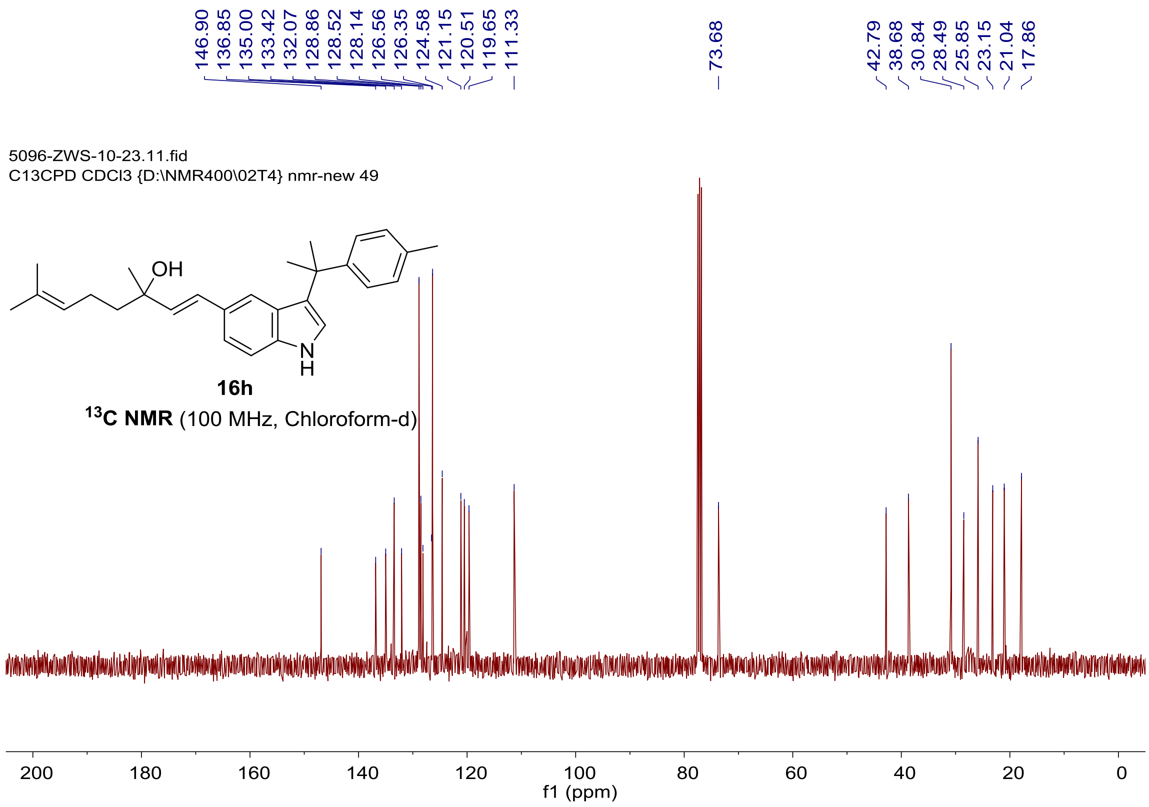
146.90
136.85
135.00
133.42
132.07
128.86
128.52
128.14
126.56
126.35
124.58
121.15
120.51
119.65
111.33
73.68
42.79
38.68
30.84
28.49
25.85
23.15
21.04
17.86
5096-ZWS-10-23.11.fid
C13CPD CDCl3 {D:\NMR400\02T4} nmr-new 49
OH
N
H
16h
13C NMR (100 MHz, Chloroform-d)
200
180
160
140
120
100
80
60
40
20
0
f1 (ppm)

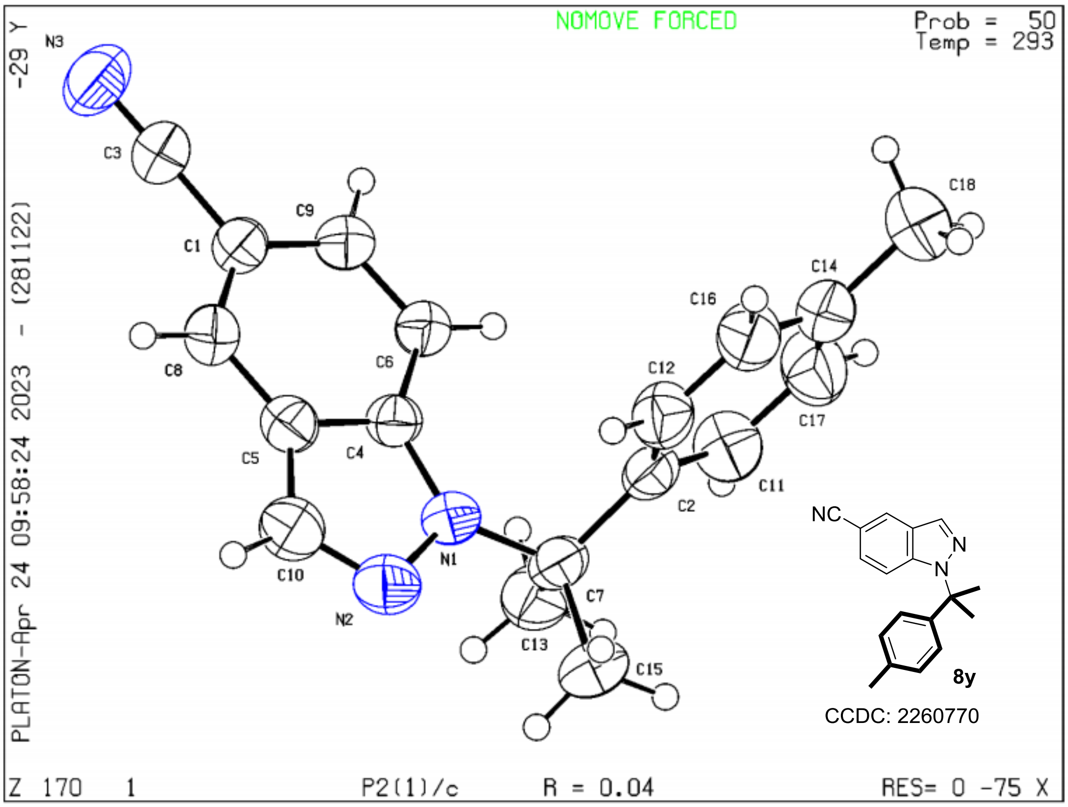
NOMOVE FORCED
Prob = 50
Temp = 293
-29 Y
PLATON-Apr 24 09:58:24 2023 - (281122)
N3
C3
C1
C9
C8
C6
C5
C4
C10
N2
N1
C7
C13
C15
C18
C14
C16
C12
C17
C2
C11
NC
N
N
8y
CCDC: 2260770
Z 170 1
P2(1)/c
R = 0.04
RES= 0 -75 X

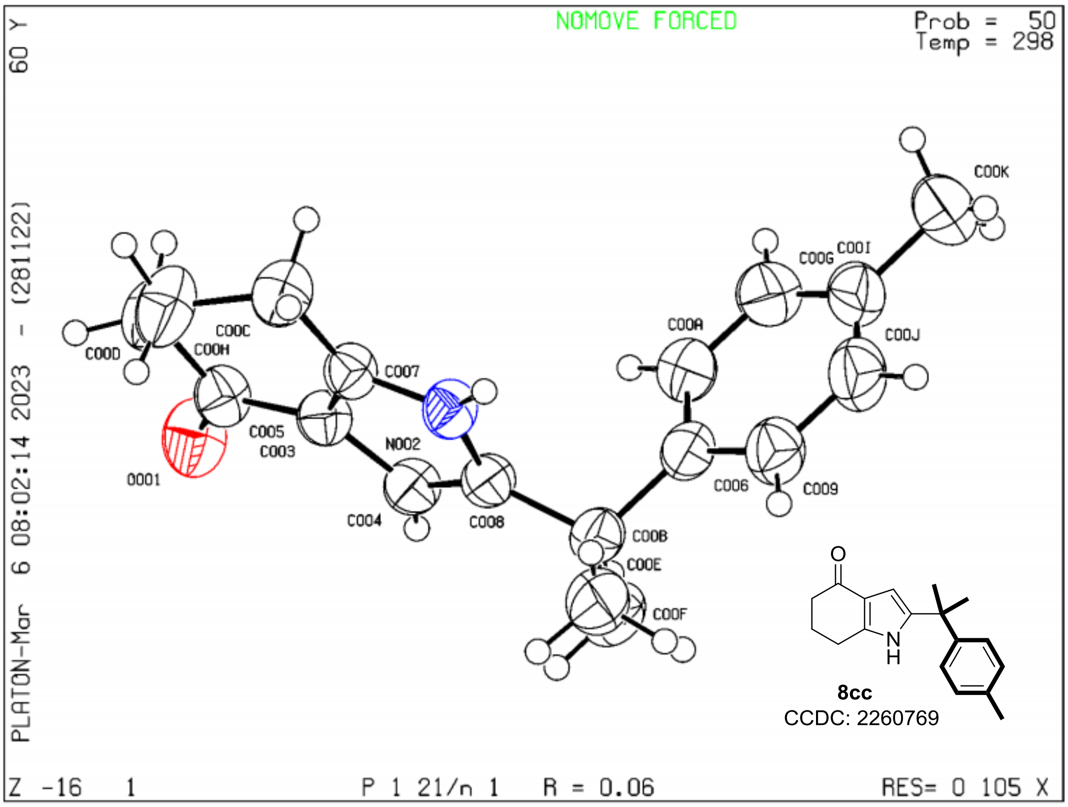
NOMOVE FORCED
Prob = 50
Temp = 298
60 Y
PLATON-Mar 6 08:02:14 2023 - (281122)
C00D
C00H
C00C
C007
O001
C005
C003
N002
C004
C008
C00B
C00E
C00F
C00A
C00G
C00I
C00K
C00J
C006
C009
O
H
N
8cc
CCDC: 2260769
Z -16 1
P 1 21/n 1
R = 0.06
RES= 0 105 X

7. Supplementary References

1 Song, H. *et al.* HFIP as protonation reagent and solvent for regioselective alkylation of indoles with all-carbon centers. *J. Org. Chem.* **87**, 1086-1097 (2022).

2 Esezobor, O. Z., Zeng, W., Niederegger, L., Grubel, M. & Hess, C. R. Co-mabiq flies solo: light-driven Markovnikov-selective C- and N-alkylation of indoles and indazoles without a cocatalyst. *J. Am. Chem. Soc.* **144**, 2994-3004 (2022).

3 Wang, M. Z., Wong, M. K. & Che, C. M. Gold (I)-catalyzed intermolecular hydroarylation of alkenes with indoles under thermal and microwave-assisted conditions. *Chem. Eur. J.* **14**, 8353-8364 (2008).

4 Comelli, N. A., Ponzi, E. N. & Ponzi, M. I. Isomerization of a-Pinene, Limonene, a-Terpinene, and Terpinolene on Sulfated Zirconia. *J. Am. Oil Chem. Soc.* **82**, 531-535 (2005).

5 Gassman, P. G., Bonser, S. M. & Mlinaric-Majerski, K. Molecules with twist bent bonds. The synthesis, properties, and reactions of trans-bicyclo[4.1.0]hept-3-ene and certain methylated derivatives. *J. Am. Chem. Soc.* **111**, 2652-2662 (1989).

6 Kawahara, T. *et al.* Ring-opening reactions of α- and β-pinenes in pressurized hot water in the absence of any additive. *Org. Process Res. Dev.* **17**, 1485-1491 (2013).

7 Hu, Y. C., Ji, D. W., Zhao, C. Y., Zheng, H. & Chen, Q. A. Catalytic prenylation and reverse prenylation of indoles with isoprene: regioselectivity manipulation through choice of metal hydride. *Angew. Chem. Int. Ed.* **58**, 5438-5442 (2019).

8 Luzung, M. R., Lewis, C. A. & Baran, P. S. Direct, chemoselective N-tert-prenylation of indoles by C-H functionalization. *Angew. Chem. Int. Ed.* **48**, 7025-7029 (2009).

9 Wang, Z., Zeng, H. & Li, C.-J. Dearomatization–rearomatization strategy for reductive cross-coupling of indoles with ketones in water. *Org. Lett.* **21**, 2302-2306 (2019).

10 Yang, Y. & Buchwald, S. L. Ligand-controlled palladium-catalyzed regiodivergent Suzuki–Miyaura cross-coupling of allylboronates and aryl halides. *J. Am. Chem. Soc.* **135**, 10642-10645 (2013).

11 Pei, M., Wang, A., Xie, X., Hu, X. & Liu, Y. Gold-catalyzed cyclization of ynones involving cis-hydrofunctionalizations: rapid assembly of C-, O-, or S-functionalized pyrroles by a single methodology. *Org. Lett.* **24**, 1541-1545 (2022).

12 Vandavasi, J. K., Hua, X., Halima, H. B. & Newman, S. G. A nickel-catalyzed carbonyl-Heck reaction. *Angew. Chem. Int. Ed.* **56**, 15441-15445 (2017).

13 Yokoyama, Y. *et al.* Chemoselective palladium-catalyzed reaction in aqueous media: selectivity in the reaction of haloanilines with 1,1-dimethylallyl alcohol. *Adv. Synth. Catal.* **349**, 662-668 (2007).
